# Association between sleep-related phenotypes and gut microbiota: a two-sample bidirectional Mendelian randomization study

**DOI:** 10.3389/fmicb.2024.1341643

**Published:** 2024-02-02

**Authors:** Xiaoqiu Wang, Chi Wang, Kai Liu, Qingyun Wan, Wenzhong Wu, Chengyong Liu

**Affiliations:** Jiangsu Province Hospital of Chinese Medicine, Affiliated Hospital of Nanjing University of Chinese Medicine, Nanjing, Jiangsu, China

**Keywords:** sleep-related phenotypes, gut microbiota, causal effect, Mendelian randomization, insomnia

## Abstract

**Background:**

An increasing body of evidence suggests a profound interrelation between the microbiome and sleep-related concerns. Nevertheless, current observational studies can merely establish their correlation, leaving causality unexplored.

**Study objectives:**

To ascertain whether specific gut microbiota are causally linked to seven sleep-related characteristics and propose potential strategies for insomnia prevention.

**Methods:**

The study employed an extensive dataset of gut microbiota genetic variations from the MiBioGen alliance, encompassing 18,340 individuals. Taxonomic classification was conducted, identifying 131 genera and 196 bacterial taxa for analysis. Sleep-related phenotype (SRP) data were sourced from the IEU OpenGWAS project, covering traits such as insomnia, chronotype, and snoring. Instrumental variables (IVs) were selected based on specific criteria, including locus-wide significance, linkage disequilibrium calculations, and allele frequency thresholds. Statistical methods were employed to explore causal relationships, including inverse variance weighted (IVW), MR-Egger, weighted median, and weighted Mode. Sensitivity analyses, pleiotropy assessments, and Bonferroni corrections ensured result validity. Reverse causality analysis and adherence to STROBE-MR guidelines were conducted to bolster the study’s rigor.

**Results:**

Bidirectional Mendelian randomization (MR) analysis reveals a causative interplay between selected gut microbiota and sleep-related phenotypes. Notably, outcomes from the rigorously Bonferroni-corrected examination illuminate profound correlations amid precise compositions of the intestinal microbiome and slumber-associated parameters. Elevated abundance within the taxonomic ranks of class Negativicutes and order Selenomonadales was markedly associated with heightened susceptibility to severe insomnia (OR = 1.03, 95% CI: 1.02–1.05, *p* = 0.0001). Conversely, the augmented representation of the phylum Lentisphaerae stands in concord with protracted sleep duration (OR = 1.02, 95% CI: 1.01–1.04, *p* = 0.0005). Furthermore, heightened exposure to the genus *Senegalimassilia* exhibits the potential to ameliorate the manifestation of snoring symptoms (OR = 0.98, 95% CI: 0.96–0.99, *p* = 0.0001).

**Conclusion:**

This study has unveiled the causal relationship between gut microbiota and SRPs, bestowing significant latent value upon future endeavors in both foundational research and clinical therapy.

## 1 Introduction

In recent years, insomnia disorders have gradually emerged as a burden on public health (Glick et al., 2023). Recent studies indicate that the overall pooled prevalence of post-COVID sleep disturbances is 28.98% (Linh et al., 2023). These patients commonly experience challenges in initiating sleep, easy arousal, shortened sleep duration, early awakening, and disruptive snoring. Subsequently, daytime drowsiness becomes evident, presenting an air of lethargy. Sleep deprivation or inadequate sleep is correlated with elevated blood pressure (Makarem et al., 2021), weight augmentation (Nielsen et al., 2011; Ogilvie and Patel, 2017), heightened susceptibility to diabetes, cardiovascular ailments (He et al., 2017; Ogilvie and Patel, 2018), and compromised immune system functionality (Besedovsky et al., 2019). Managing sleep-related issues in a clinical setting remains inherently challenging. While the temporary alleviation of insomnia disorder symptoms can be achieved through the use of hypnotic medications, it fails to address the fundamental causes, resulting in patients excessively relying on such medications without resolving the underlying issues (Rocha et al., 2023). Sleep cognitive-behavioral therapy stands out as a primary therapeutic modality for ameliorating sleep problems. Unlike hypnotic drugs, it avoids side effects such as daytime somnolence, dizziness, weight gain, and addiction. Nevertheless, it is not without drawbacks, including discontinuation risks (Nam et al., 2023), relatively low accessibility (Koffel et al., 2018), and a time-consuming, intricate nature (Chung et al., 2023). The widespread occurrence of insomnia, coupled with negative clinical consequences and the lack of effective preventive and treatment strategies, emphasizes the need for comprehensive research to enhance understanding, investigate pathophysiology, analyze potential risk factors, and develop innovative approaches for prevention and therapy.

The pathophysiology of insomnia is exceedingly intricate, which is influenced by factors such as sleep environment, dietary habits, endocrine disruptions, psychological issues, and circadian rhythm disturbances, all of which can impact sleep. Among these, the gut microbiota (GM) engages in reciprocal interaction with brain function through the brain–gut microbiota axis (BGMA), constituting a complex and extensive bidirectional communication network. Previous research suggests that the maintenance of normal sleep physiology is inseparable from the assistance of GM, with the richness and diversity of GM fluctuating in accordance with alterations in sleep patterns, duration, and other related sleep features. Studies have identified *Lachnospira* and *Bacteroides* as signature bacteria distinguishing acute insomnia patients from healthy controls, while *Faecalibacterium* and Blautia serve as signature bacteria for distinguishing chronic insomnia patients from healthy controls (Li et al., 2020). This underscores the potential of gut microbiota as a vital indicator for the ancillary diagnosis of insomnia, offering potential novel therapeutic targets within the realm of insomnia disorders. Scholars have delved into specific genus differences between insomnia patients and healthy individuals, demonstrating the capability of addressing disrupted GM homeostasis and ameliorating insomnia, symptoms, as well as enhancing sleep quality through the administration of *Lactobacillus brevis* DL1-11 (Yu et al., 2020). Additionally, existing animal experiments validate that *Lactobacillus brevis*-fermented γ-aminobutyric acid improves sleep behaviors in fruit flies and rodent models (Jeong et al., 2021).

However, in observational studies, the association between intestinal microbiota and insomnia is easily affected by confounding factors such as age, environment, diet pattern, and lifestyle, which limits the confirmation of the causal relationship between intestinal microbiota and insomnia. Most antecedent investigations were configured as cross-sectional studies, posing challenges in ascertaining the duration of exposure and definitive outcomes. As an illustration, Grosicki et al. (2020) discerned that *Blautia* and *Ruminococcus* exhibited abundance in individuals attesting superior sleep quality, juxtaposed with *Prevotella*, which displayed diminished abundance, and a concomitant elevation in the α diversity of the gut microbiota. In addition, the results of some animal experiments are also inconsistent. For example, Poroyko et al. (2016) found that *Firmicutes* increased and *Bacteroidetes* decreased in mice with sleep fragmentation, while Maki et al. (2020) found that during the period of chronic sleep deprivation, *Bacteroidetes* decreased as compared with the control group. The abundance, F:B ratio, and α diversity of Firmicutes were decreased.

In this context, Mendelian randomization (MR) is a novel way to explore the causal relationship between the gut microbiota and SRPs. Mendelian randomization is an analytical technique in epidemiology that leverages genetic variation as an instrumental variable for exposure to explore a causal association between exposure factors and outcome events (Yavorska and Burgess, 2017). The advantage of MR is that because genetic variation is random, the analysis process is less influenced by environmental factors and lifestyle, which minimizes bias due to residual confounding (Haycock et al., 2016). Moreover, since the genetic variation is not altered by the disease state, this avoids the possibility of reverse causality in the analysis. MR has been widely used to explore the causal relationship between the gut microbiome and diseases, including metabolic diseases (Sanna et al., 2019), pregnancy-related diseases (Li P. et al., 2022; Li C. et al., 2022), autoimmune diseases (Xu et al., 2021), cardiovascular diseases (Zhang Y. et al., 2022), chronic kidney disease (Li N. et al., 2023), and rheumatoid arthritis (Inamo, 2021). In this study, we aimed to use pooled statistics from genome-wide association studies (GWAS) from MiBioGen and the UKB Consortium to elucidate potential causal relationships between gut microbiota and SRPs using a two-sample MR approach. Identifying the relationship between them will help promote the prediction and prevention of related diseases and the further development of precision medicine. This will have a positive impact on population health and healthcare spending.

## 2 Methods

### 2.1 Data sources

The genetic variations within the gut microbiota were obtained through the most extensive genomic compilation of gut microbiota composition to date, which were carried out by the MiBioGen alliance (Kurilshikov et al., 2021). This study focused on the V4, V3–V4, and V1–V2 variable regions of the 16S rRNA gene, encompassing 18,340 individuals from 24 cohorts, with a predominant European ancestry (*n* = 13,266). A total of 211 gut microbiota profiles and 122,110 associated single nucleotide polymorphisms (SNPs) were recorded, and a taxonomic classification was conducted using direct taxonomic classification methods. Host genetic variations related to bacterial taxa abundance within the gut microbiota were identified through microbiota quantitative trait locus (mbQTL) mapping analysis. A total of 131 genera with an average abundance exceeding 1% were identified, including 12 unknown genera. Consequently, this study encompassed 119 genus-level taxonomic groups for analysis, comprising 196 bacterial taxa.

The summary statistics for GWAS of Sleep-Related Phenotypes (SRPs) were sourced from the IEU OpenGWAS project, encompassing traits such as sleeplessness/insomnia, chronotype (morning/evening preference), sleep duration, morning wakefulness, daytime napping, narcolepsy, and snoring. The GWAS ID, sample size, and SNP count for these traits are presented in Supplementary Table S1. Trait definitions utilized in this study are presented in Supplementary Table S2. Information pertaining to gut microbiota relevant to the study is presented in Supplementary Table S3.

### 2.2 The selection of instrumental variables

The following criteria guided IV selection as follows: (1) SNPs linked to each genus at a significance level of *p* < 1.0 × 10^−5^ were chosen as potential IVs (Li P. et al., 2022); (2) The linkage disequilibrium (LD) between SNPs was calculated using 1,000 Genomes Project European sample data as the reference panel, and among those with R^2^ < 0.001 (using a clumping window size of 10,000 kb), only the SNPs with the most significant *p*-values were retained; (3) SNPs with a minor allele frequency (MAF) ≤ 0.01 were excluded; (4) In cases of palindromic SNPs, the forward strand alleles were determined based on allele frequency information.

### 2.3 Statistical analysis

In this study, we employed a range of statistical methods to investigate the causal relationship between exposure factors and the study’s outcomes. Specifically, we used inverse variance weighted (IVW), MR-Egger, weighted median, and weighted mode methods. IVW, a classic technique, merged Wald ratio estimates from individual instrumental variables in a meta-analysis, essentially implementing a weighted linear regression relating instrumental variables to the outcome. IVW is advantageous as it provides unbiased estimates without horizontal pleiotropy. MR-Egger, based on the InSIDE assumption, primarily reflects the dose–response relationship between instrumental variables and outcomes, acknowledging some level of pleiotropy (Bowden et al., 2015). The weighted median method reduced class 1 errors and accommodated potentially invalid genetic variants. The weighted mode approach remained credible when most instrumental variables yielded consistent causal estimates, even if some did not meet MR method requirements. The primary method utilized is the IVW methods with a random effect assumption, assuming all SNPs to be valid instrumental variables or balanced horizontal pleiotropy, providing the most precise and unbiased estimates.

To ensure uniformity in effect alleles, we harmonized summary statistics and removed SNPs with unclear strands. We excluded palindromic SNPs to prevent allele effects on gut microbiota taxa and SRP causality. Horizontal pleiotropy and outliers were assessed using MR-Egger and MR Pleiotropy RESidual Sum and Outlier (MR-PRESSO) tests. Cochrane’s Q test was used to test heterogeneity among instrumental variables. Leave-one-out sensitivity analysis tested outliers and result stability. To establish causality rigorously, we applied Bonferroni corrections based on the number of bacteria attributes (genera, families, orders, classes, and phyla).

A reverse causality analysis was conducted, and *p*-values within the range of the corrected values to 0.05 were considered nominally indicative of causation. The study was adhered to the STROBE-MR guidelines (Skrivankova et al., 2021), and statistical analyses were conducted using R software version 4.3.1, facilitated by the R package TwoSampleMR (version 0.5.6).

In summary, various statistical techniques were employed to comprehensively examine the causal relationship between exposure factors and outcomes, with IVW being the primary method. Sensitivity analyses and rigorous statistical corrections were performed to enhance the robustness and validity of the results. Additionally, reverse causality was considered.

## 3 Results

### 3.1 Selection of instrumental variables

We meticulously curated instrumental variables for 211 bacterial taxa. Those instrumental variables, which met the genome-wide significance threshold (*p* < 1 × 10^−5^) and had the confounding effects of specific taxonomic groups removed through linkage disequilibrium calculations, are presented in Supplementary Tables S4–S17. Upon rigorous examination, we observed an absence of weak instrumental variables, as all instrumental variables exhibited an F-statistic exceeding 10 (Supplementary Tables S32–S45).

### 3.2 Bidirectional two-sample MR analysis

#### 3.2.1 Daytime dozing/sleeping (narcolepsy)

**Figure 1:**
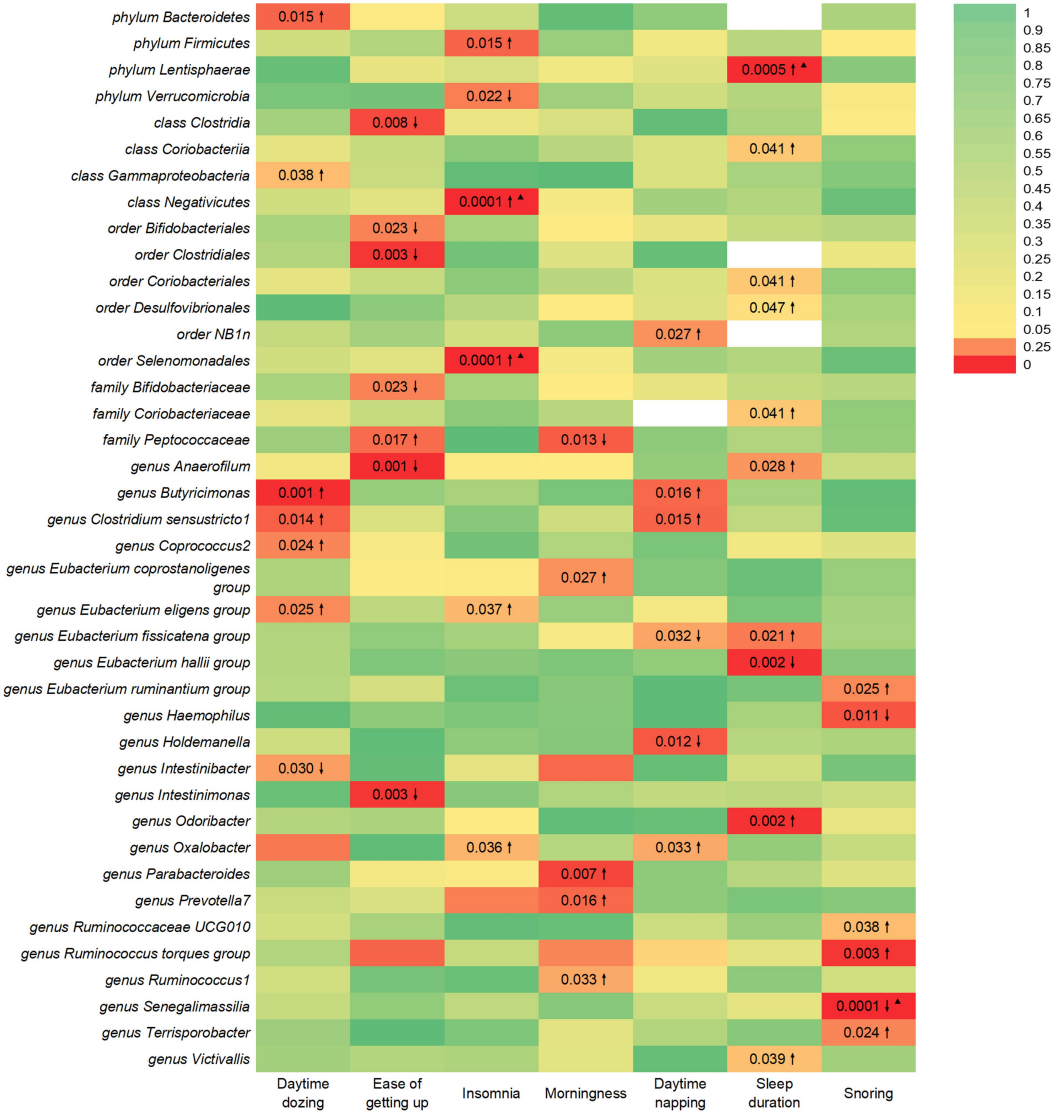
IVW estimates from gut microbiota on SRPs. The color of each block represents the IVW-derived *p*-values of every MR analysis. *p*-values of <0.05 were shown in red and *p*-values of >0.05 were shown in yellow or green. *p*-value <0.05 is set as nominal significant, whereas ^▲^ represents significant [genera: 0.05/131 (3.81 × 10^−4^), families: 0.05/35 (1.4 × 10^−3^), orders: 0.05/20 (2.5 × 10^−3^), classes: 0.05/16 (3.1 × 10^−3^), and phyla: 0.05/9 (5.5 × 10^−3^)]. ↑ and ↓ represent, respectively, an increased and decreased risk of developing sleep-related traits in the gut microbiota.

This study identified seven causal relationships between the gut microbiota and the risk of developing daytime dozing (Figure 1). A higher genetically predicted class Gammaproteobacteria, phylum Bacteroidetes, genus *Butyricimonas*, genus *Clostridium sensustricto1*, genus *Coprococcus2*, and genus *Eubacterium eligens group* were associated with a higher risk of daytime dozing. Differently, genus *Intestinibacter* was associated with a lower risk.

In the reverse study, nine causal relationships were identified between daytime dozing and the risk of changes in gut microbiota. Daytime dozing may lead to a higher rate of *genus Flavonifractor*, *genus Anaerofilum, class Deltaproteobacteria,* and *genus Eubacterium eligens* group and a lower rate of *genus Fusicatenibacter, genus Oxalobacter, genus Slackia, genus Butyricicoccus,* and *genus Dorea*.

#### 3.2.2 Getting up in the morning

This study identified seven causal relationships between the gut microbiota and the risk of getting up in the morning (Figure 1). A higher genetically predicted family Peptococcaceae was associated with a higher risk of getting up in the morning. Differently, genus *Intestinimonas,* order Bifidobacteriales, order Clostridiales, family Bifidobacteriaceae, genus *Anaerofilum,* and class Clostridia were associated with a lower risk.

In the reverse study, seven causal relationships were identified between getting up in the morning and the risk of changes in gut microbiota. Getting up in the morning may lead to a higher rate of genus *Terrisporobacter,* order Bacteroidales, phylum Bacteroidetes, class Bacteroidia, and genus *Clostridium innocuum group* and a lower rate of genus *Intestinimonas and* genus *Slackia*.

#### 3.2.3 Sleeplessness/insomnia

This study identified six causal relationships between the gut microbiota and the risk of developing insomnia (Figure 1). A higher genetically predicted genus *Oxalobacter,* class Negativicutes, order Selenomonadales, phylum Firmicutes, and genus *Eubacterium eligens group* were associated with a higher risk of insomnia. Differently, phylum Verrucomicrobia was associated with a lower risk.

In the reverse study, 12 causal relationships were identified between insomnia and the risk of changes in gut microbiota. Insomnia may lead to a higher rate of genus *Oxalobacter,* family Clostridiaceae1, and family Oxalobacteraceae and a lower rate of genus *Ruminococcaceae UCG013*, order Erysipelotrichales, order Rhodospirillales, class Alphaproteobacteria, family Erysipelotrichaceae, and family Lachnospiraceae.

#### 3.2.4 Morning/evening person (chronotype)

This study identified seven causal relationships between the gut microbiota and the risk of developing being a morning/evening person (Figure 1). A higher genetically predicted genus *Parabacteroides*, genus *Prevotella7,* genus *Ruminococcus1,* and genus *Eubacterium coprostanoligenes group* were associated with a higher risk of being morning/evening person. Differently, family Peptococcaceae was associated with a lower risk.

In the reverse study, eight causal relationships were identified between being a morning/evening person and the risk of changes in gut microbiota. Being a morning/evening person may lead to a higher rate of *genus Ruminococcaceae UCG010*, family Porphyromonadaceae, and genus Butyricicoccus and a lower rate of *genus Streptococcus*, order Lactobacillales, class Bacilli, family Peptostreptococcaceae, and family Streptococcaceae.

#### 3.2.5 Nap during day

This study identified six causal relationships between the gut microbiota and the risk of developing daytime napping (Figure 1). A higher genetically predicted genus *Oxalobacter*, order NB1n, genus *Butyricimonas,* and genus *Clostridium sensustricto1* were associated with a higher risk of daytime napping. Differently, genus *Holdemanella* and genus *Eubacterium fissicatena group* were associated with a lower risk.

**Figure 2:**
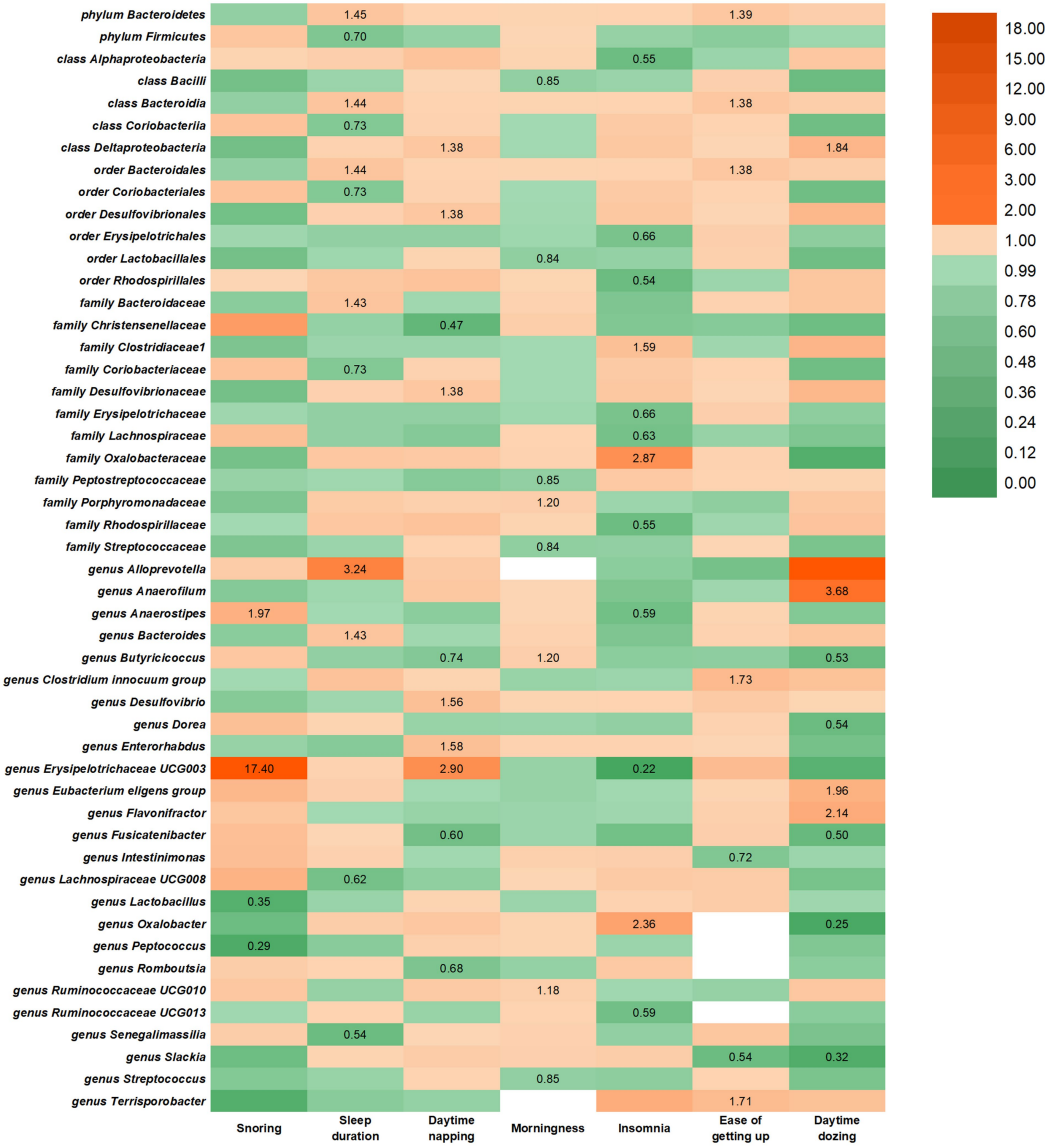
IVW estimates from SRPs on gut microbiota. The color of each block represents the IVW-derived odds ratio of every MR analysis. Odds ratio of <1.00 were shown in green and odds ratio of >1.00 were shown in yellow or orange.

In the reverse study, 10 causal relationships were identified between daytime napping and the risk of changes in gut microbiota (Figure 2). Daytime napping may lead to a higher rate of family Desulfovibrionaceae, class Deltaproteobacteria, genus *Desulfovibrio*, *genus Enterorhabdus,* and *genus Erysipelotrichaceae UCG003* and a lower rate of *genus Fusicatenibacter, genus Romboutsia,* order Desulfovibrionales, family Christensenellaceae, and *genus Butyricicoccus*.

#### 3.2.6 Sleep duration

This study identified seven causal relationships between the gut microbiota and sleep duration (Figure 1). A higher genetically predicted genus *Odoribacter*, genus *Victivallis*, order Coriobacteriales, order Desulfovibrionales, phylum Lentisphaerae, family Coriobacteriaceae, genus *Anaerofilum*, class Coriobacteriia, and genus *Eubacterium fissicatena group* were associated with a higher risk of longer sleep duration. Differently, genus *Eubacterium hallii group* was associated with a higher risk of shorter sleep duration.

In the reverse study, seven causal relationships were identified between sleep duration and the risk of changes in gut microbiota. Longer sleep duration may lead to a higher rate of *or*der Bacteroidales, family Bacteroidaceae, phylum Bacteroidetes, class Bacteroidia, genus *Alloprevotella,* and genus *Bacteroides* and a lower rate of genus *Lachnospiraceae UCG008,* genus *Senegalimassilia,* order Coriobacteriales, phylum Firmicutes, family Coriobacteriaceae and class Coriobacteriia.

#### 3.2.7 Snoring

This study identified six causal relationships between the gut microbiota and the risk of developing snoring (Figure 1). A higher genetically predicted genus *Ruminococcaceae UCG010,* genus *Ruminococcus torques group,* genus *Terrisporobacter,* genus *Eubacterium ruminantium group* were associated with a higher risk of snoring. Differently, genus *Haemophilus* and genus *Senegalimassilia* were associated with a lower risk.

In the reverse study, four causal relationships were identified between snoring and the risk of changes in gut microbiota. Getting up in the morning may lead to a higher rate of genus *Anaerostipes* and genus *Erysipelotrichaceae UCG003* and a lower rate of genus *Lactobacillus* and genus *Peptococcus.*

### 3.3 Bonferroni-corrected test, sensitivity analysis, and reverse analysis

The results from the Bonferroni-corrected test revealed that a higher level of class Negativicutes retains a strong causal relationship with more severe insomnia (OR = 1.03, 95% CI: 1.02 to 1.05, *p* = 0.0001), whereas a higher level of order Selenomonadales retains a strong causal relationship with more severe insomnia (OR = 1.03, 95% CI: 1.02 to 1.05, p = 0.0001). At the same time, it turns out that a higher level of phylum Lentisphaerae leads to longer sleep duration (OR = 1.02, 95% CI: 1.01 to 1.04, *p* = 0.0005). In addition, more exposure to the *genus Senegalimassilia* has the potential to lead to improved symptoms of snoring (OR = 0.98, 95% CI: 0.96 to 0.99, *p* = 0.0001).

In Figure 1, we illustrate the causal links between intestinal flora and various outcomes. Notably, the F-statistics of our IVs ranged from 20.35 to 27.31, effectively mitigating any bias associated with weak IVs. Cochran’s IVW Q test results revealed no significant heterogeneity among these IVs (Supplementary Tables S32–S38). Additionally, our MR-Egger regression intercept analysis showed no significant directional horizontal pleiotropy (Supplementary Tables S32–S38). Although potential IV outliers were observed upon visual inspection in scatter plots (Supplementary Figures 61–107), further analysis using MR-PRESSO did not identify any significant outliers. Consequently, there is insufficient evidence to support horizontal pleiotropy in the association between these bacteria and SRPs. Notably, our risk estimations for genetically predicted outcomes remained stable in the leave-one-out analysis, indicating that the causal relationship was not driven by specific SNPs (Supplementary Figures 61–107).

Reverse MR analysis revealed a suggestive association between seven SRPs and 50 intestinal flora. However, Bonferroni-corrected testing indicated that only nominal significant associations were found between SRPs and other gut microbiota, as shown in Figure 2. Notably, Cochran’s IVW Q test showed no significant heterogeneity among SRP IVs (Supplementary Tables S39–S45), and both MR-Egger regression intercept analysis and MR-PRESSO analysis failed to detect significant horizontal pleiotropy (Supplementary Tables S39–S45). Similar to forward analysis, our leave-one-out analysis (Supplementary Figures 1–60) demonstrated no significant changes in risk estimates for genetic predictions, confirming that causality was not driven by specific SNPs.

## 4 Discussion

### 4.1 Sleeplessness/insomnia

A multitude of preceding studies underscores a keen interest in unraveling the potential molecular mechanisms linking the intestinal microbiota to the onset of insomnia. Disruption of the gut microbiome can precipitate intestinal inflammation and neural inflammation, thereby becoming embroiled in the pathogenesis of insomnia (de Theije et al., 2014; Sun and Shen, 2018). We have ascertained a positive correlation between the genus *Oxalobacter*, order Selenomonadales, phylum Firmicutes and the genus *Eubacterium eligens group*, with the propensity for insomnia. The order Selenomonadales, phylum Firmicutes, and the genus *Eubacterium eligens group* can generate butyrate (Louis and Flint, 2017; Gasaly et al., 2021), a metabolite intimately associated with inflammation. It is conceivable that butyrate may modulate inflammatory responses in the brain, employing microglial cells as target cells. An investigation suggests that butyrate, serving as a signaling molecule in the gut–brain axis, may ameliorate the cognitive impairments induced by sleep deprivation (Wang et al., 2023). Conversely, a lower association is established between insomnia and phylum Verrucomicrobia. An observational study corroborates our findings, demonstrating amelioration in sleep quality and a higher proportion of phylum Verrucomicrobia associated therewith. The assessment of sleep quality is conducted through the employment of the PSQI, underscoring that an augmentation in phylum Verrucomicrobia can engender an improvement in insomnia.

Insomnia exerts a multifaceted impact on the gut microbiota. Insomnia may lead to a reduction in the abundance of *genus Ruminococcaceae UCG013*, order Erysipelotrichales, order Rhodospirillales, and family Erysipelotrichaceae. In a study investigating sleep deprivation and inflammatory responses in mice (Zhang M. et al., 2023), a substantial increase in the abundance of *Rhodospirillales* and *Ruminococcaceae UCG013* was observed among sleep-deprived mice. Another study (Benedict et al., 2016), conducted on nine healthy adult males subjected to partial sleep deprivation, also noted a significant increase in the family Erysipelotrichaceae compared with baseline. These findings diverge from our research, potentially implying that experimentally induced sleep deprivation and subjective insomnia are not entirely congruent phenomena.

Furthermore, our investigation has unveiled that insomnia augments the abundance of the genus *Oxalobacter*, and the elevation of genus *Oxalobacter*, in turn, escalates the risk of insomnia. There exists a bidirectional causative relationship between these two factors, thus fostering a pernicious cycle of insomnia. However, it is worth noting that as of now, no study has definitively substantiated the causal link between these factors. Consequently, further research aimed at elucidating the causal relationship between them that may serve as a prospective avenue for future inquiries.

### 4.2 Sleep duration

In the investigation of the causal relationship between gut microbiota and sleep duration, we have discerned an association between the genus *Odoribacter* and an increased risk of prolonged sleep. A study concerning sleep disturbances in traumatic brain injury patients revealed a relative increase in the abundance of the genus *Odoribacter* within the intestinal microbiota of individuals who were suffering from post-traumatic sleep disturbances (Zhanfeng et al., 2022). This elevation may be correlated with excessively prolonged sleep following cranial trauma. Conversely, there exists a correlation between the genus *Eubacterium hallii group* and the risk of shortened sleep duration. Qiu et al. (2022) identified *Eubacterium hallii* as a microbial marker for sleep disturbances in individuals both before and after exercise interventions. The augmentation of *Eubacterium hallii* is associated with healthy sleep patterns. Based on the findings from MR analysis, it is conceivable that the genus *Eubacterium hallii group* may ameliorate sleep quality by modulating excessively extended sleep durations.

Regarding the study of the causal relationship between sleep duration and the risk of changes in the gut microbiota, it has been observed that longer sleep durations may lead to a diminished abundance of order Coriobacteriales, family Coriobacteriaceae, and class Coriobacteriia. In a cross-sectional investigation (Zhang et al., 2021), researchers found a negative correlation, which was statistically significant, between the relative abundance of *Coriobacteriia* and *Coriobacteriales* in individuals with type 1 narcolepsy and their total sleep duration, which aligns with the outcomes of our MR analysis.

Conversely, higher levels of order Coriobacteriales, family Coriobacteriaceae, and class Coriobacteriia may engender an increased risk of prolonged sleep duration. Lin et al. (2021) demonstrated that the probiotic *Lactobacillus PS150* can ameliorate sleep disturbances induced by the “first night effect” in mice, leading to an extension of sleep duration. They also noted a substantial increase in *Coriobacteriia* in the fecal samples of mice treated with *Lactobacillus PS150*. Therefore, in conjunction with the findings from our MR study, we have reasonable grounds to surmise that *Lactobacillus PS150* may indirectly contribute to the extension of sleep duration by reshaping the microbial composition, specifically with regard to *Coriobacteriia*.

Therefore, it is evident that *Coriobacteriia* plays a pivotal role in the bidirectional regulation of sleep duration. When considering therapeutic interventions for issues of excessive or insufficient sleep duration, it may be prudent to contemplate strategies that target this particular microbial community.

### 4.3 Easiness of getting up in the morning

This investigation unveils that the presence of the genus *Anaerofilum* mitigates the propensity for morning reluctance. A systematic review concerning the gut microbiota in individuals with depression has highlighted the heightened abundance of the genus *Anaerofilum* in this cohort. Previous studies (Kripke, 2016) have illustrated an inverse association between ease of early rising and depressive tendencies. Hence, the heightened abundance of the genus *Anaerofilum* in individuals with depression precipitates the challenge of early morning awakening.

Simultaneously, research has indicated that the use of sertraline, a commonly prescribed antidepressant, elevates the difficulty of morning arousal (Sun et al., 2017). This indirectly corroborates our findings. Furthermore, we have ascertained a bidirectional causal relationship between an increased abundance of the genus *Intestinimonas* and a reduction in the risk of morning reluctance. However, current evidence does not substantiate a definitive causative link between these factors. Thus, further investigations are imperative to ascertain the existence of a causal relationship between them.

### 4.4 Chronotype of morning/evening person

Previous investigations have elucidated diurnal fluctuations in the functionality and composition of the gut microbiota, which are intricately linked to circadian rhythms (Thaiss et al., 2014; Leone et al., 2015). Disruptions in the ecological equilibrium of the gut can lead to temporal misalignment (Wang et al., 2022). In our study, we have discerned that augmentation in the genus *Ruminococcus1* and the genus *Eubacterium coprostanoligenes group* escalates the risk of manifesting the chronotype of a morning or evening person. Carasso et al. (2021) have established a positive correlation between heightened fruit consumption and levels of the *genus Ruminococcus* within the microbiota, while increased intake of fruits and vegetables is associated with morning chronotypes. The gut microbiota plays a pivotal role in facilitating significant alterations in the expression patterns of circadian clock genes (Choi et al., 2021). For instance, in a study conducted on mice, it was observed that microbial populations, such as the genus *Eubacterium coprostanoligenes group*, generate butyrate, which modulates the expression of hepatic circadian clock genes (Leone et al., 2015). These findings align harmoniously with the outcomes of our investigation.

Similarly, perturbations in the host’s circadian rhythms can impact the gut microbiota. Alterations in clock genes (Bmal1 or Per1/2) can influence the rhythmic oscillations of the gut microbiota. Long-term disruptions in circadian rhythms induced by changes in light exposure, jet lag, and shift work also exert an influence on the oscillatory patterns of the gut microbiota (Thaiss et al., 2014; Liang et al., 2015). However, the specific impact of circadian rhythm alterations on distinct microbial populations within the gut remains unclear. Currently, there is a growing interest in understanding how circadian rhythms can be harnessed for the prevention and treatment of psychiatric disorders. Future research endeavors may delve deeper into the intricate mechanisms through which circadian rhythms affect specific microbial communities within the gut, restore physiological circadian patterns, consider potential microbial transformations, and devise optimal intervention strategies (Zhang P. et al., 2023).

### 4.5 Daytime napping and daytime dozing

Our investigation reveals a bidirectional causal relationship between the gut microbiota and both daytime napping and daytime dozing, with considerable overlap in the specific microbial populations involved. For instance, an elevation in the levels of the genus *Butyricimonas* and genus *Clostridium sensustricto1* is associated with an increased susceptibility to daytime napping and daytime dozing. Conversely, engaging in daytime napping and daytime dozing results in an elevated proportion of class Deltaproteobacteria, accompanied by a decrease in the levels of the genus *Fusicatenibacter* and genus *Butyricicoccus*.

Previous research have uncovered a negative correlation between *genus Butyricicoccus* and the severity of rapid eye movement (REM) sleep, and in patients afflicted with Parkinson’s disease (PD), a reduction in the abundance of *genus Butyricicoccus* is noted (Zhang Q. et al., 2023). This decrease may be linked to the heightened frequency of daytime dozing observed in PD patients. Furthermore, previous research (Zhang Q. et al., 2023) has indicated that individuals with Cushing’s syndrome exhibit a significantly lower relative abundance of the genus *Eubacterium eligens group*, which correlates negatively with cortisol levels, systolic blood pressure (SBP), and diastolic blood pressure (DBP). Consequently, excess of the genus *Eubacterium eligens* group may lead to decreased daytime cortisol levels, thereby inducing daytime dozing, which, in turn, contributes to nocturnal difficulties in falling asleep. The underlying mechanisms driving the elevation in the abundance of the genus *Eubacterium eligens group* in response to daytime dozing remain a subject of exploration.

### 4.6 Snoring

Research concerning the interplay between the gut microbiota and snoring remains limited, while investigations into obstructive sleep apnea (OSA) have garnered considerable attention in recent years. Snoring is recognized as a hallmark of OSA (Campos et al., 2020). Further exploration of the relationship between snoring and the gut microbiota holds promise for future advancements in OSA treatments. An escalation in the levels of the genus *Ruminococcaceae UCG010* and genus *Ruminococcus torques group* is associated with an elevated risk of snoring. The study by Zhang C. et al. (2022) unveiled potential compensatory mechanisms in the alterations of the gut microbiota in the pathophysiology of OSA, proposing an association between OSA and the increased presence of *Ruminococcus_1* and *the Ruminococcus torques group* in patients. These findings are in remarkable congruence with our results.

Conversely, snoring also exerts an impact on the composition of the gut microbiota. We have observed that snoring leads to augmentation of the genus *Anaerostipes*. However, Li Q. et al. (2023) have reported a decrease in the genus Anaerostipes among individuals with severe OSA, presenting a contradiction with our findings. This incongruity may be attributed to differences between habitual non-apnoeic benign snorers and individuals with OSA. Additionally, snoring contributes to a reduction in the levels of the genus *Lactobacillus*. Studies (Liu et al., 2019) demonstrate that OSA can affect the gut microbiota of rodents by depleting lactobacilli. This observation may elucidate the decrease in *Lactobacillus*, resulting from snoring.

### 4.7 Clinical and research implications

The decline in sleep quality is considered a crucial catalyzing factor in the occurrence and exacerbation of various diseases, and is associated with an increase in healthcare expenditures. However, due to an incomplete understanding of potential mechanisms and a lack of early intervention measures, there has been a continuous quest for more effective, lower side-effect, and more convenient treatment methods. Our investigation supplements antecedent observational revelations and additionally scrutinizes the causal relationships between the gut microbiota and sleep-related attributes. These findings contribute to unveiling the impact of the gut microbiota on the development of sleep-related issues, paving novel avenues for etiological investigations into sleep-related concerns. We anticipate that the aforementioned research findings can offer potential targets for intervening in insomnia through modulation of the gut microbiota. This, in turn, may serve as a reference for the prevention and treatment of sleep-related issues. For instance, by modulating the equilibrium of the gut microbiota, it may be plausible to mitigate the risk of developing insomnia or ameliorate the severity of these conditions. This could be accomplished through dietary alterations, probiotic supplementation, and analogous modalities. Nevertheless, the research landscape in this domain is still in its embryonic phase, necessitating further studies to corroborate these preliminary findings and ascertain the most effective intervention strategies. Furthermore, intricacy and individual variability of the gut microbiota pose a challenge, potentially requiring personalized therapeutic regimens.

### 4.8 Strengths and limitations

**Figure 3:**
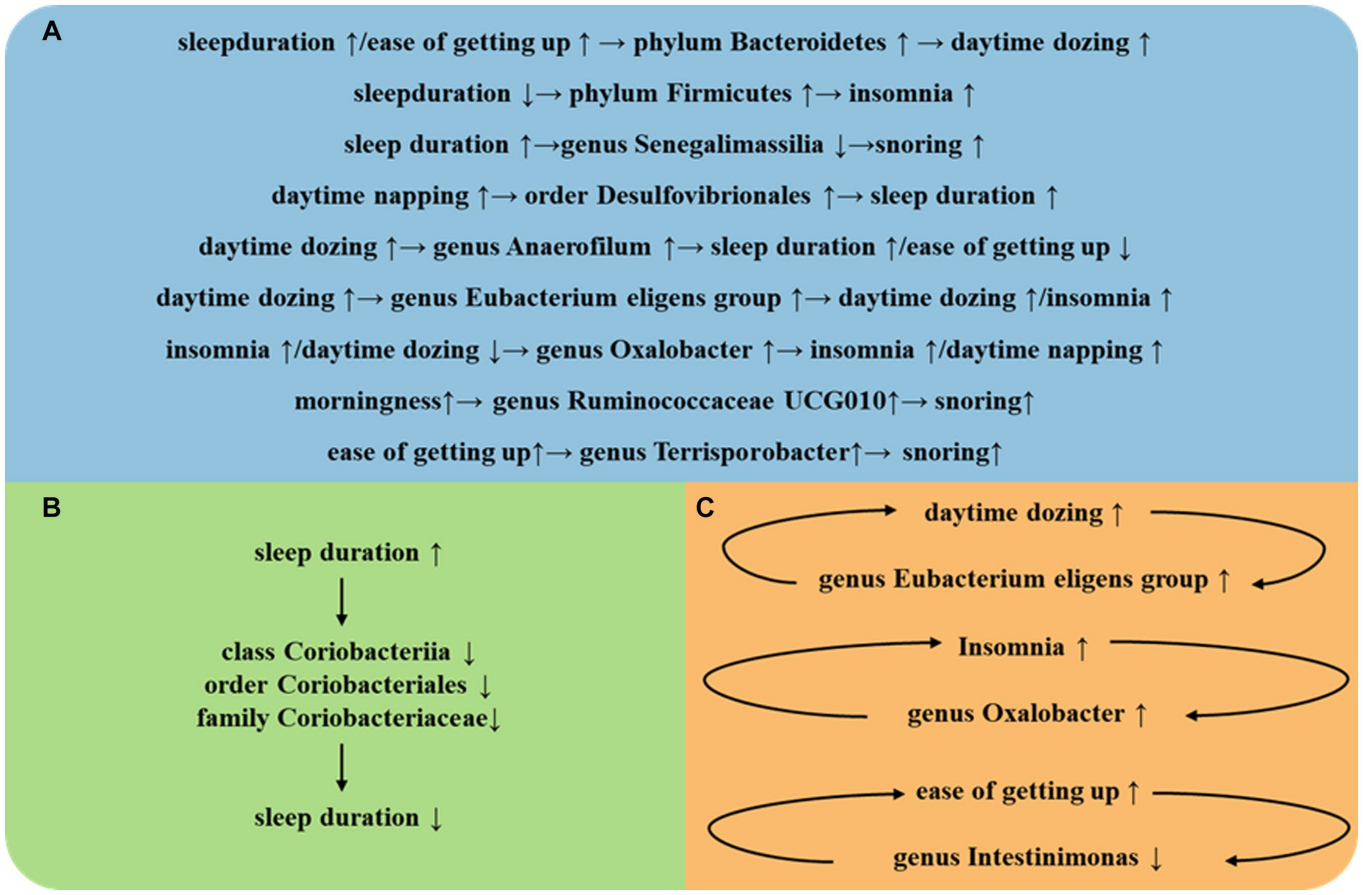
**(A)** Intestinal flora play an important role between different SRPs. **(B)** Intestinal flora play an important role in self-regulation between the same SRPs. **(C)** Intestinal flora play an important role in a vicious cycle between the same SRPs.

The strengths of this study encompass several facets. First, we have employed a multitude of sleep-related phenotypes, refining the scope of our sleep research. Second, the implementation of MR design has bolstered the credibility of causal inferences between the gut microbiota and sleep-related phenotypes by mitigating residual confounding and reverse causality. Third, the causal relationships unearthed in this investigation not only corroborate findings from previous studies but also pave the way for novel approaches in subsequent research, focusing on the targeted manipulation of specific gut bacterial populations to prevent and treat insomnia (Figure 3).

Nonetheless, certain limitations must be acknowledged. The stringent genome-wide statistical significance threshold (5 × 10^−8^) for SNPs has compelled us to integrate SNPs meeting a genome-wide significance level of 1 × 10^−5^ into this study, given their limited availability. Second, a fraction of the gut microbiota data utilized in this study originates from populations of different ethnicities, potentially introducing bias into some of the research outcomes. Finally, we cannot discount the possibility of gene-diet or gene–environment interactions affecting the observed results, as these interactions may exert an influence on the ultimate outcomes under scrutiny.

## 5 Conclusion

This study has unveiled the causal nexus between the gut microbiome and SRPs, thereby endowing prospective clinical therapeutics and foundational inquiries with profound latent worth. On the clinical front, this revelation will substantiate the domain of personalized medicine and the genesis of novel therapeutic modalities, thereby mitigating pharmaceutical dependencies. In basic research, these findings will provide essential insights into the mechanisms behind insomnia disorders, enhancing our understanding of how the gut and brain interact and contribute to sleep difficulties. Furthermore, comprehension of these causal relationships is poised to galvanize the formulation of prophylactic measures, alleviating the burden of associated maladies and fostering interdisciplinary collaborations that burgeon innovative investigations. In summary, this discovery holds the promise of providing assistance to individuals troubled by insomnia disorders.

## Data availability statement

The original contributions presented in the study are included in the article/Supplementary material, further inquiries can be directed to the corresponding authors.

## Ethics statement

All the genes used in the research data are from the public database in the large-scale genome-wide association study (GWAS), so the study does not require ethical approval.

## Author contributions

XW: Formal analysis, Methodology, Software, Writing – original draft. CW: Conceptualization, Formal analysis, Investigation, Methodology, Software, Visualization, Writing – original draft, Writing – review & editing. KL: Writing – original draft. QW: Writing – review & editing. WW: Writing – review & editing. CL: Writing – review & editing.
