# Supplementary material for: Association between sleep-related phenotypes and gut microbiota: a two-sample bidirectional Mendelian randomization study
Source: Front Microbiol. 2024 Feb 2;15:1341643. doi: 10.3389/fmicb.2024.1341643 (PMC10869596; doi:10.3389/fmicb.2024.1341643)
Supplement: Supplementary file 1 [file Data_Sheet_1.docx]

**Supplementary figures 1**

**Sup Fig. 1 to Sup Fig. 8 Scatter plots,** **leave-one-out plots and funnel plots for the causal association between daytime dozing and gut microbiota.**

**Sup Fig. 9 to Sup Fig. 15 Scatter plots,** **leave-one-out plots and funnel plots for the causal association between getting up in morning and gut microbiota.**

**Sup Fig. 16 to Sup Fig. 28 Scatter plots,** **leave-one-out plots and funnel plots for the causal association between insomnia and gut microbiota.**

**Sup Fig. 29 to Sup Fig. 36 Scatter plots,** **leave-one-out plots and funnel plots for the causal association between chronotype and gut microbiota.**

**Sup Fig. 37 to Sup Fig. 45 Scatter plots,** **leave-one-out plots and funnel plots for the causal association between nap during day and gut microbiota.**

**Sup Fig. 46 to Sup Fig. 56 Scatter plots,** **leave-one-out plots and funnel plots for the causal association between sleep duration and gut microbiota.**

**Sup Fig. 57 to Sup Fig. 60 Scatter plots,** **leave-one-out plots and funnel plots for the causal association between snoring and gut microbiota.**

**Sup Fig. 1** **Scatter plot,** **leave-one-out plot and funnel plot for the causal association between daytime dozing and *genus Anaerofilum*.**


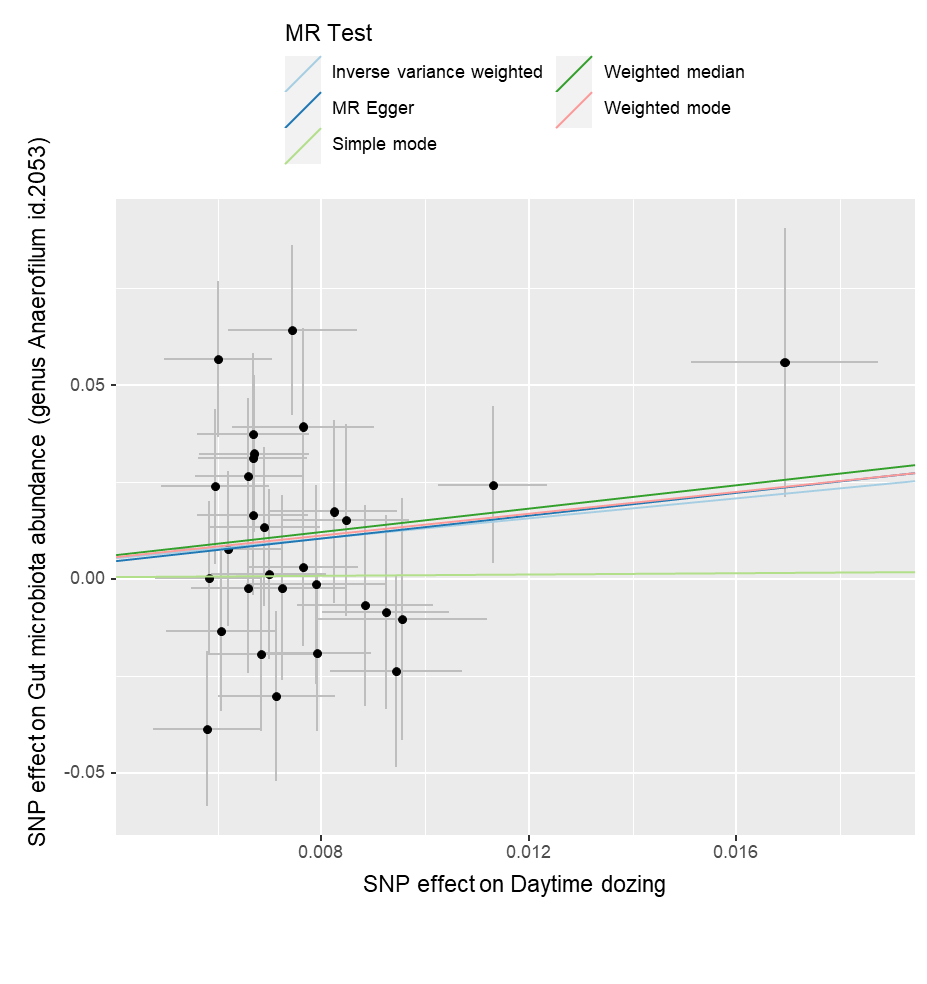

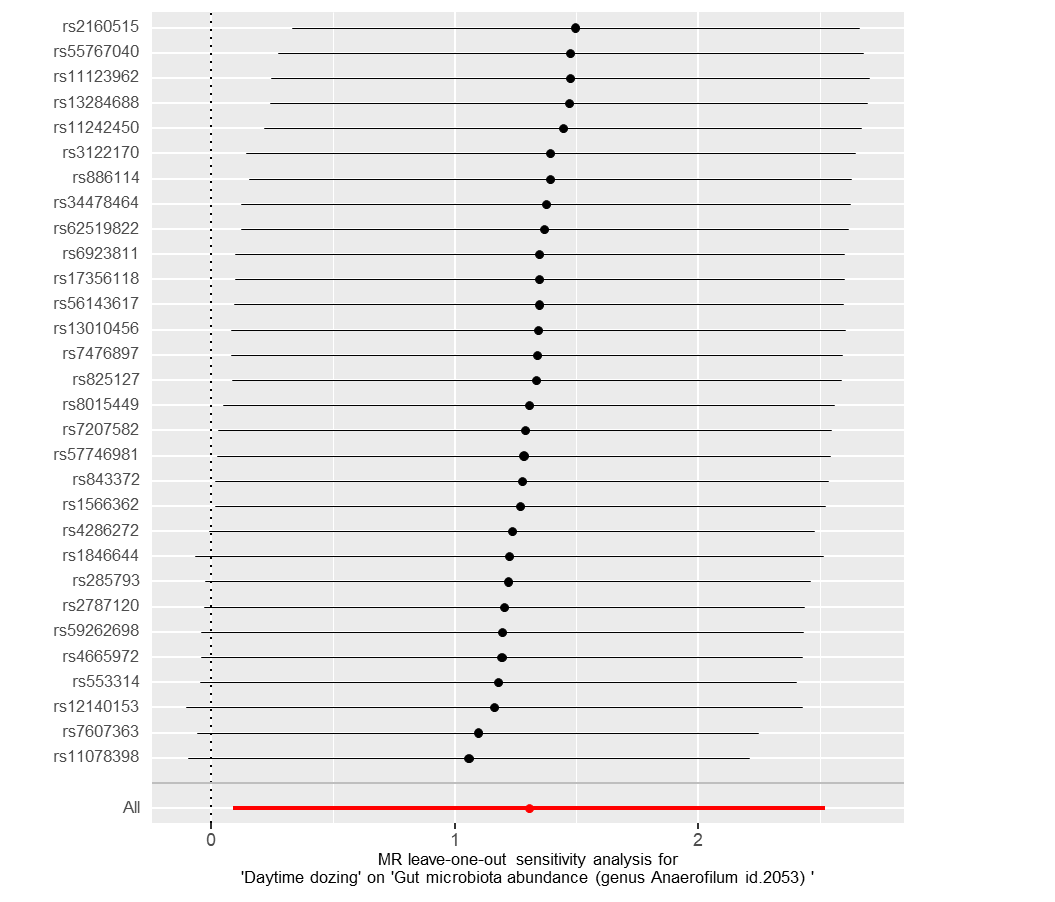

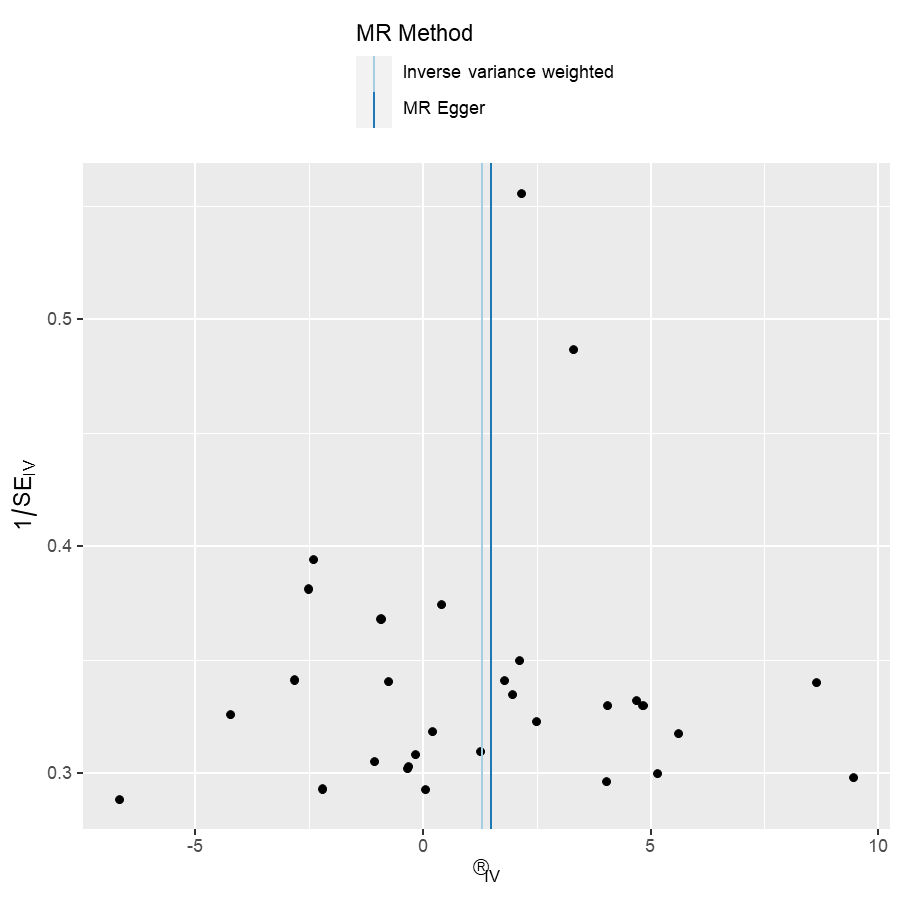


**Sup Fig. 2 Scatter plot,** **leave-one-out plot and funnel plot for the causal association between daytime dozing and *genus Butyricicoccus*.**

**
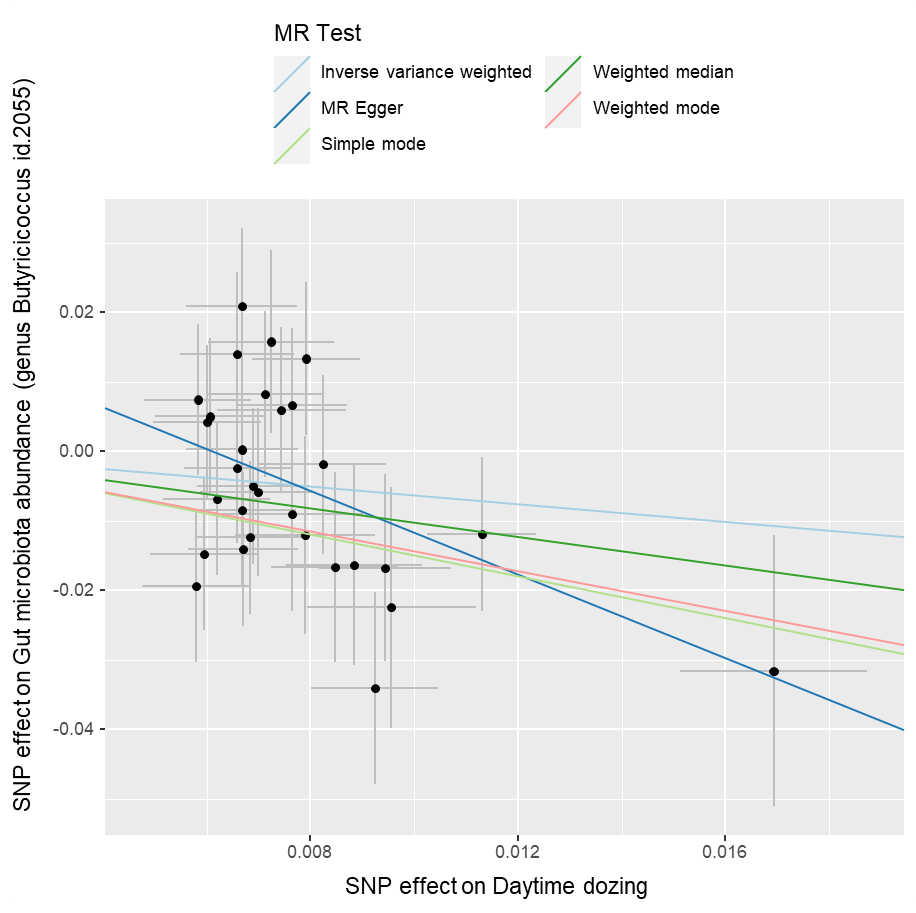

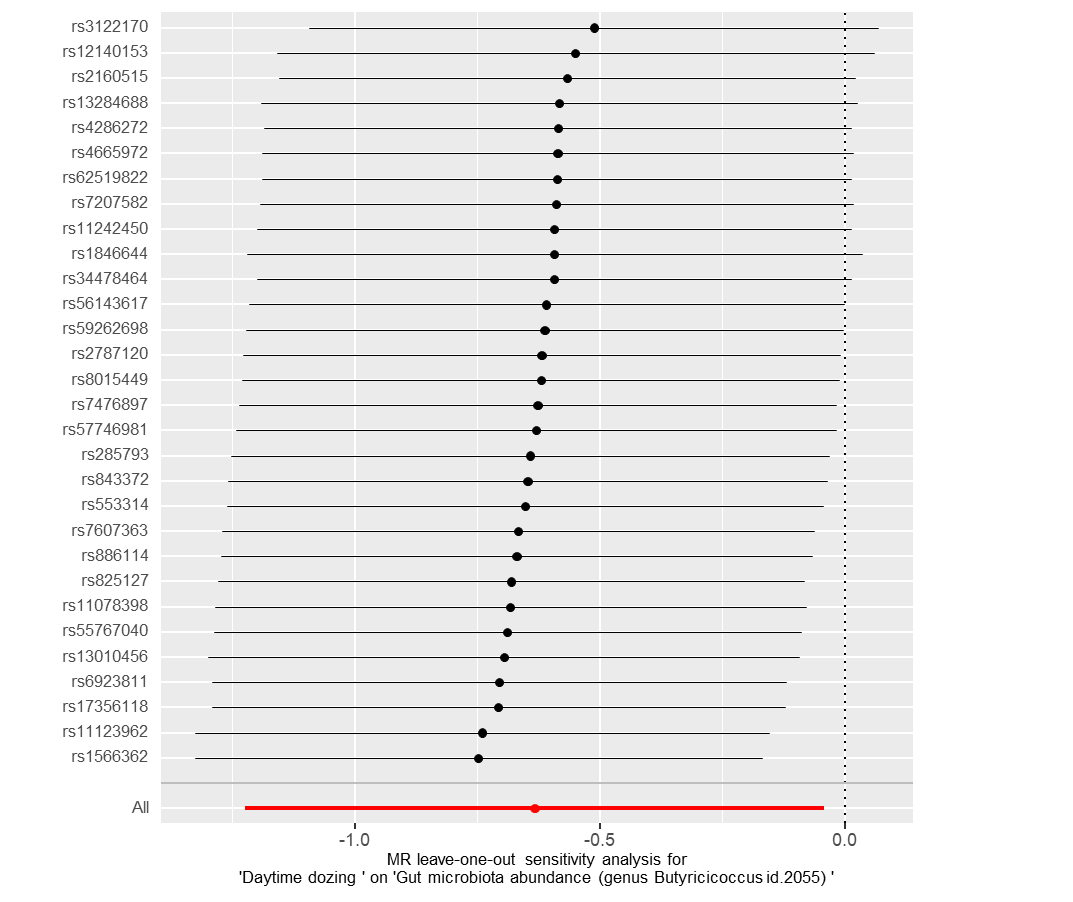

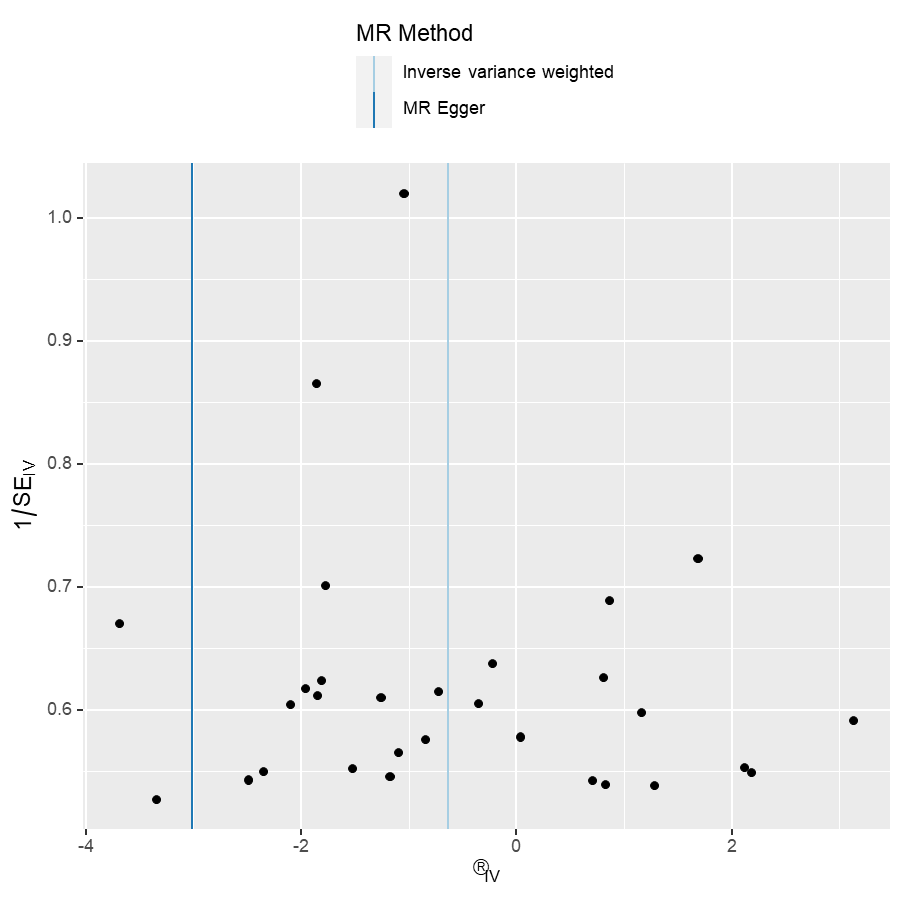
**

**Sup Fig. 3 Scatter plot,** **leave-one-out plot and funnel plot for the causal association between daytime dozing and *genus Dorea*.**

**
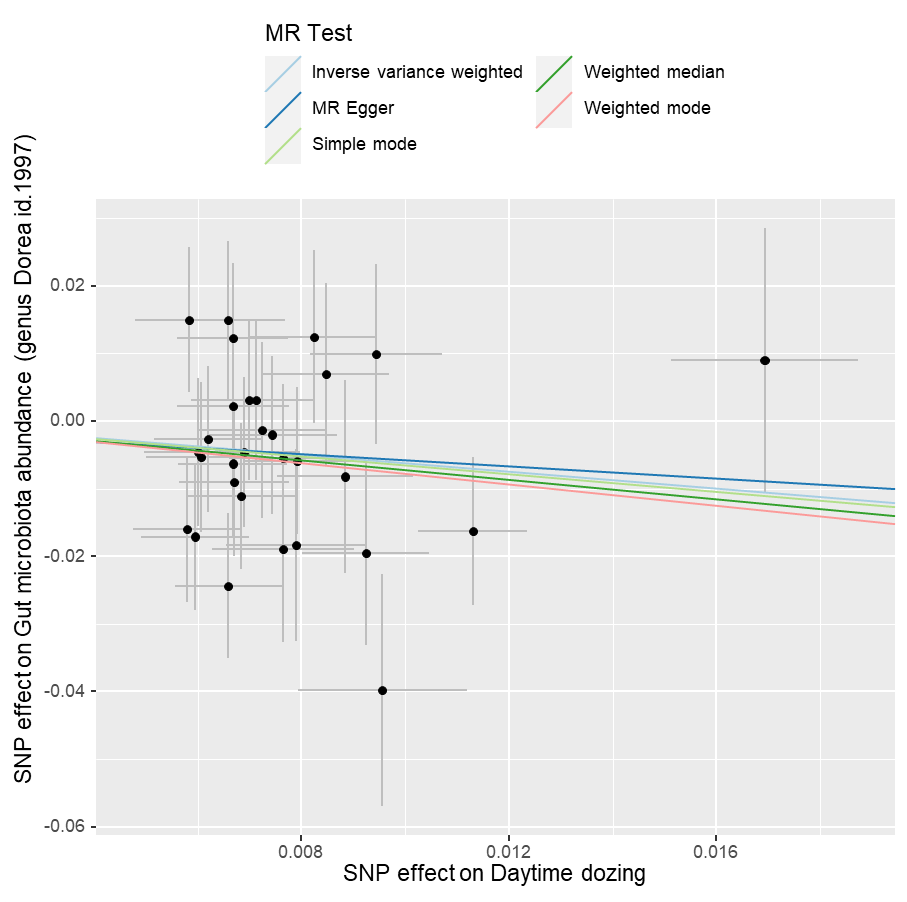

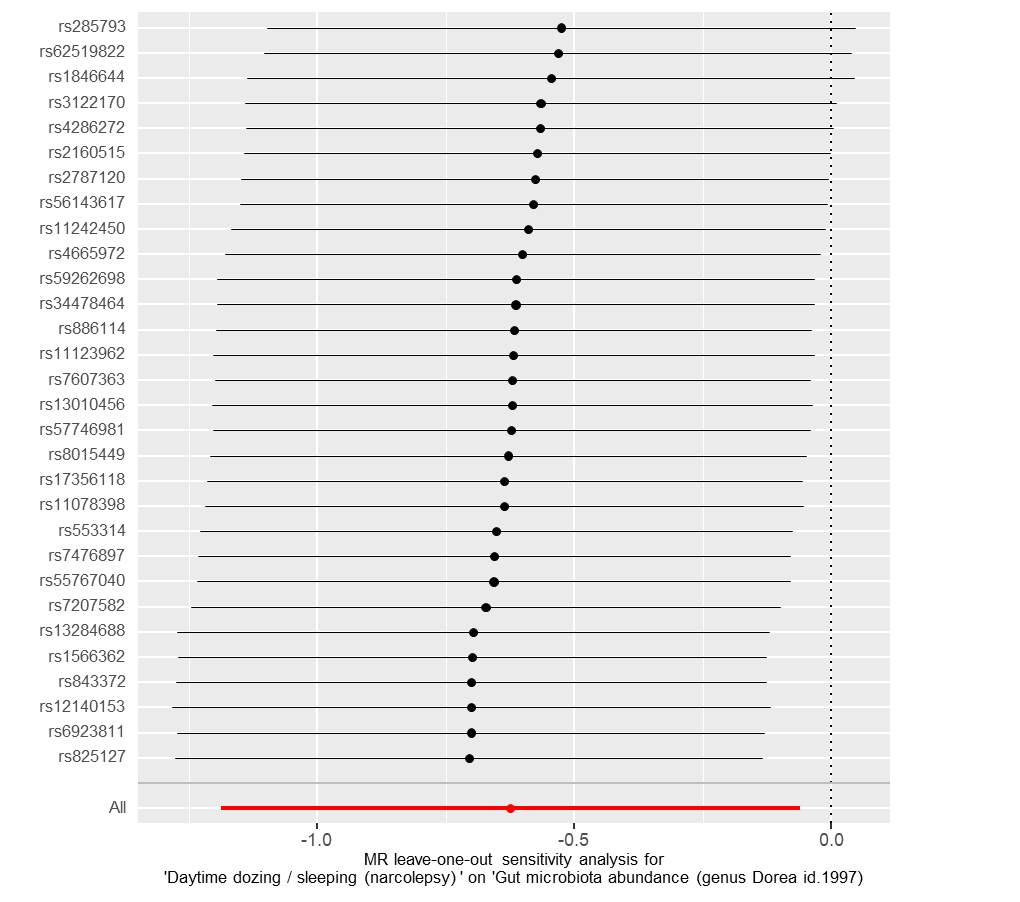

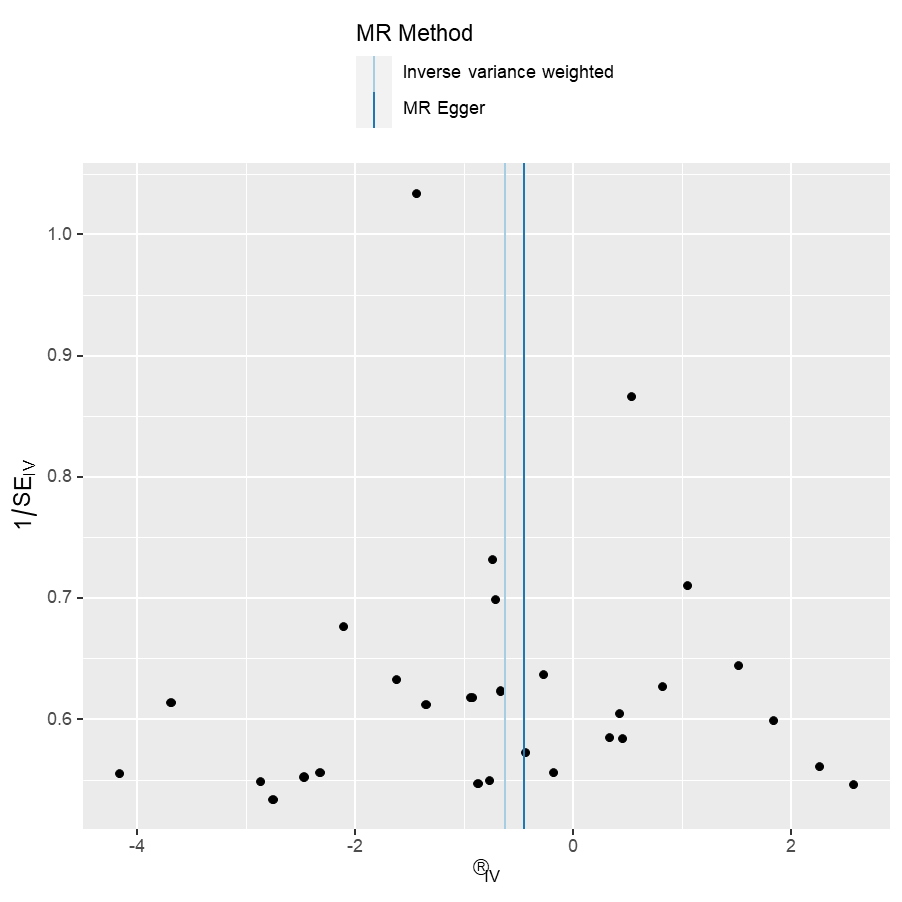
**

**Sup Fig. 4 Scatter plot,** **leave-one-out plot and funnel plot for the causal association between daytime dozing and *genus Eubacterium eligens group*.**

**
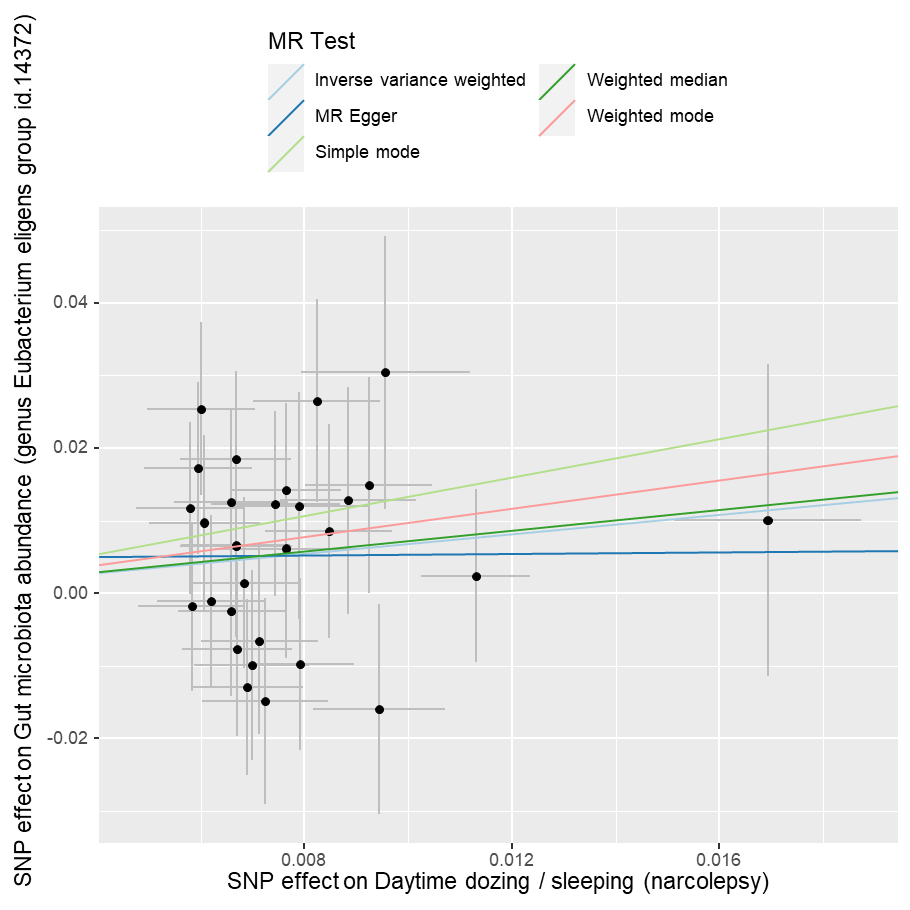

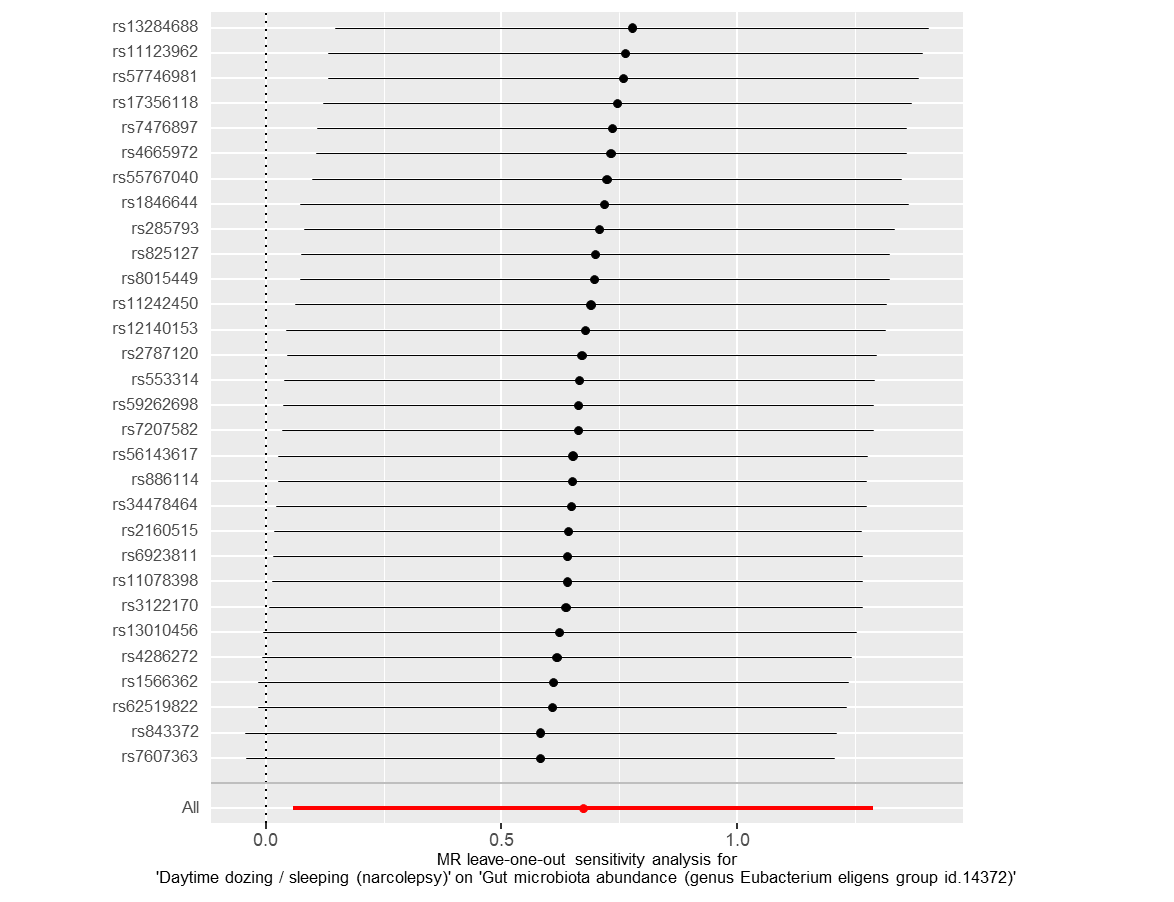

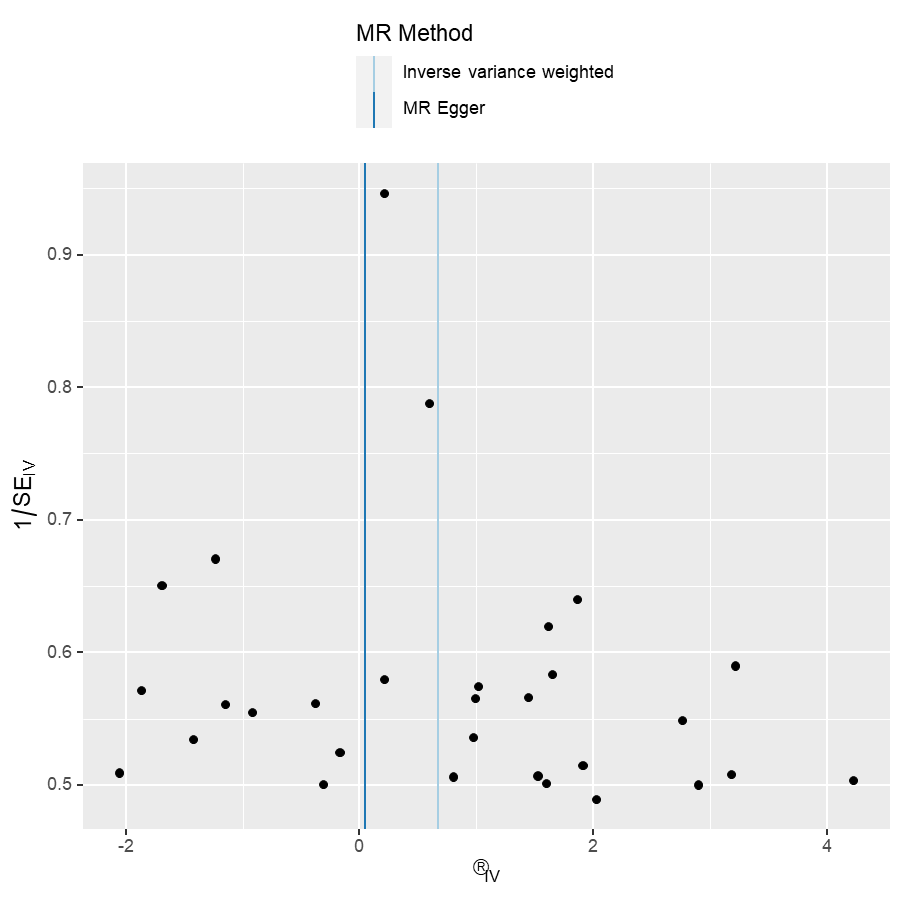
**

**Sup Fig. 5 Scatter plot,** **leave-one-out plot and funnel plot for the causal association between daytime dozing and *genus Flavonifractor*.**

**
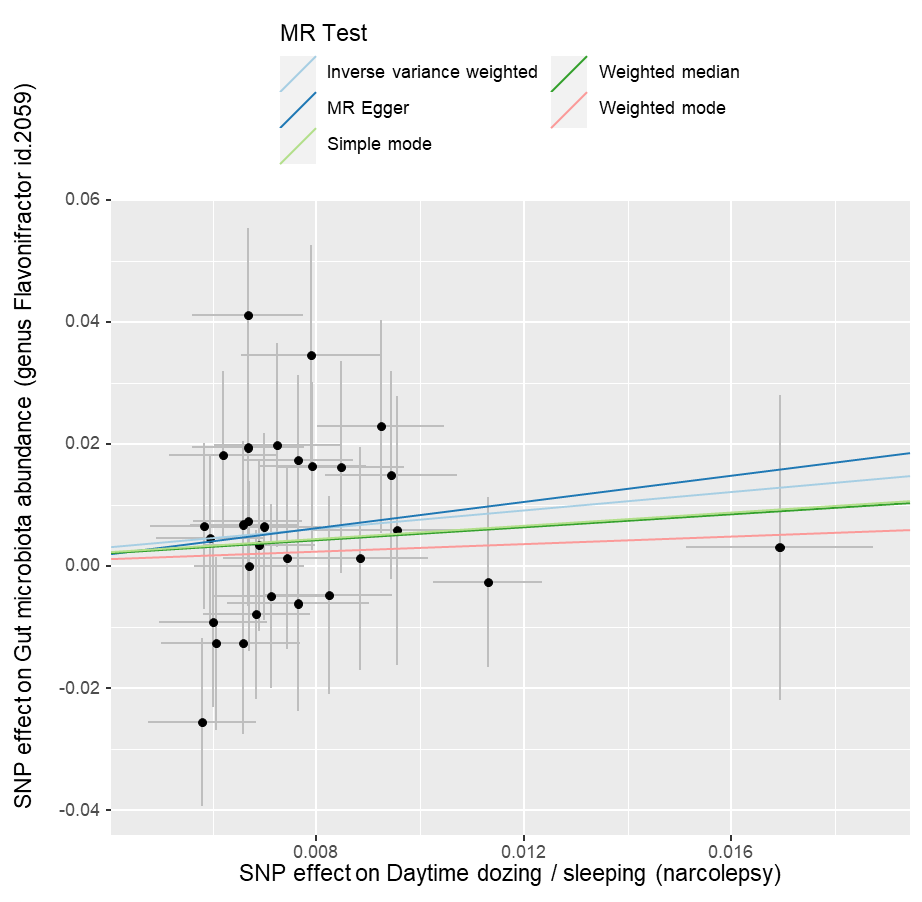

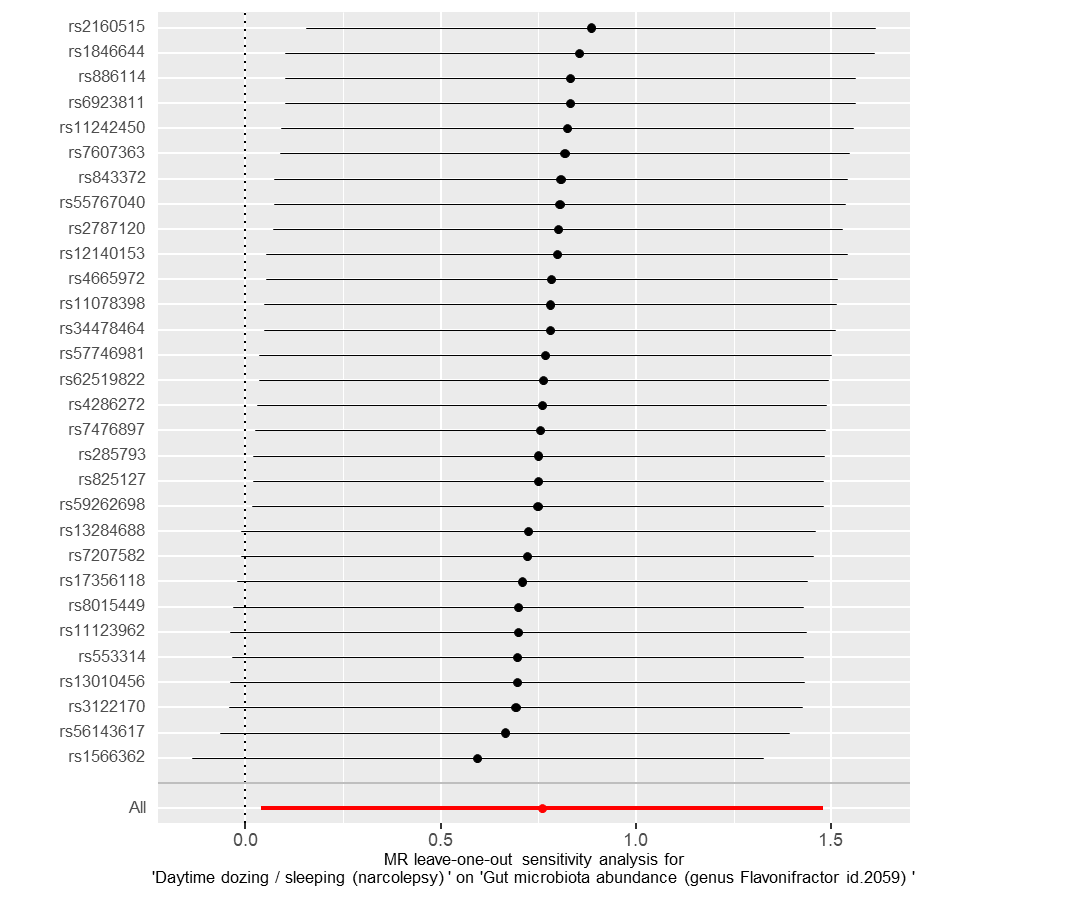

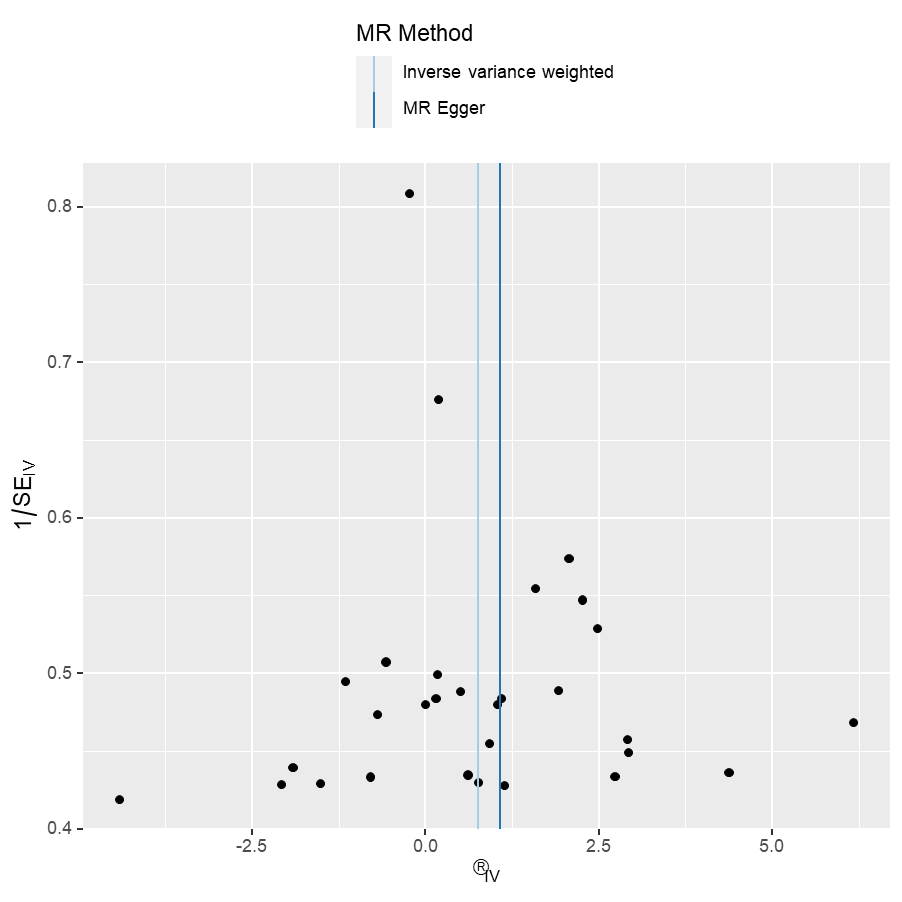
**

**Sup Fig. 6 Scatter plot,** **leave-one-out plot and funnel plot for the causal association between daytime dozing and *genus Fusicatenibacter*.**

**
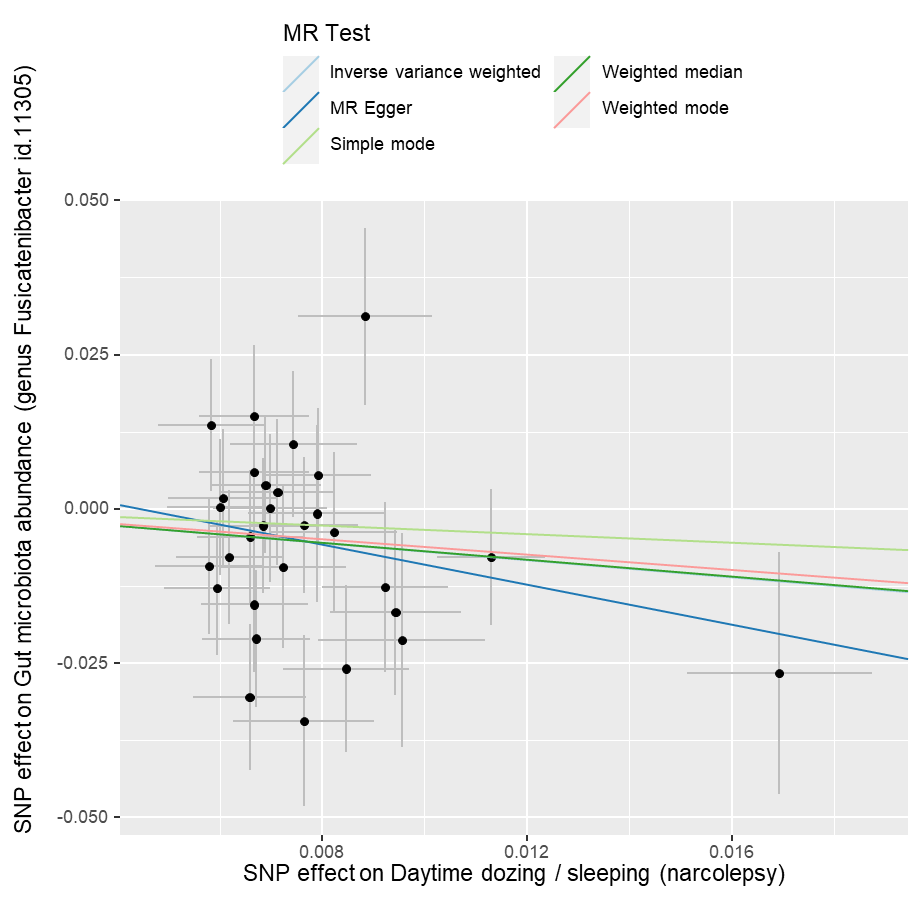

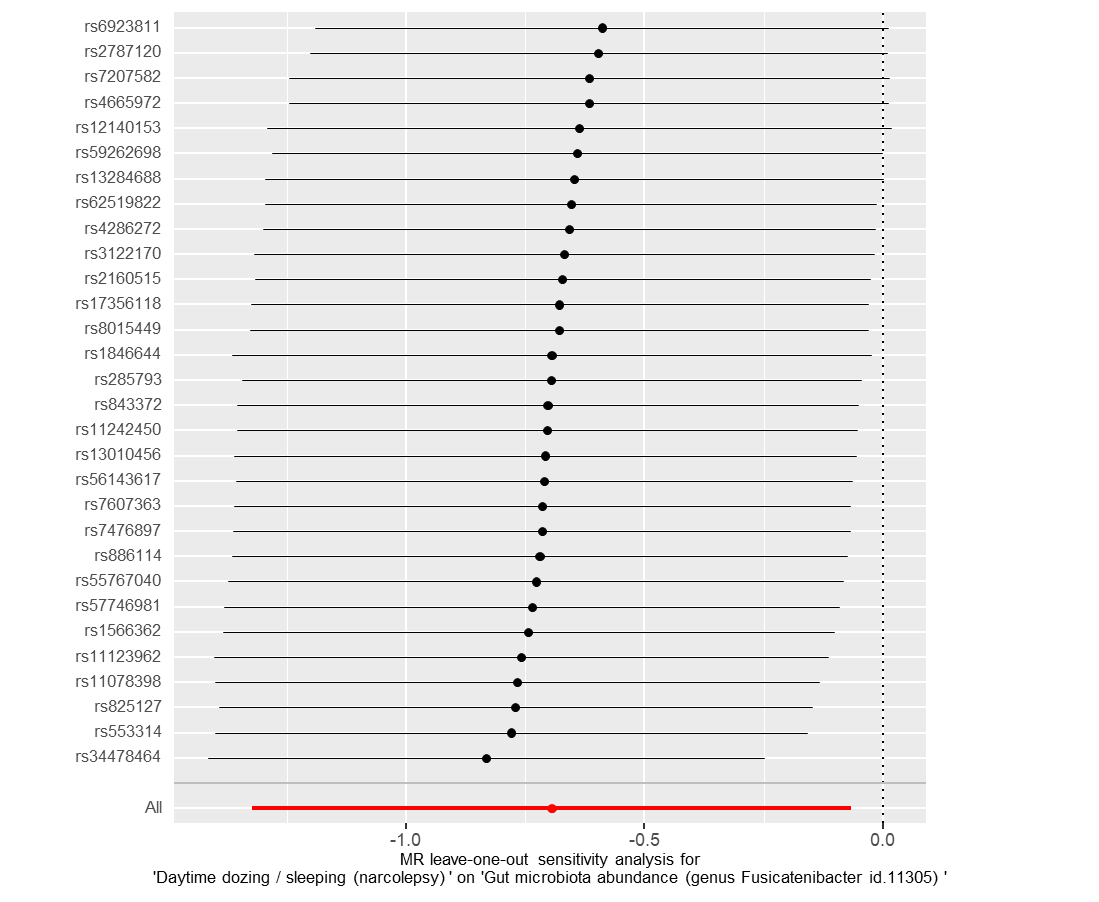

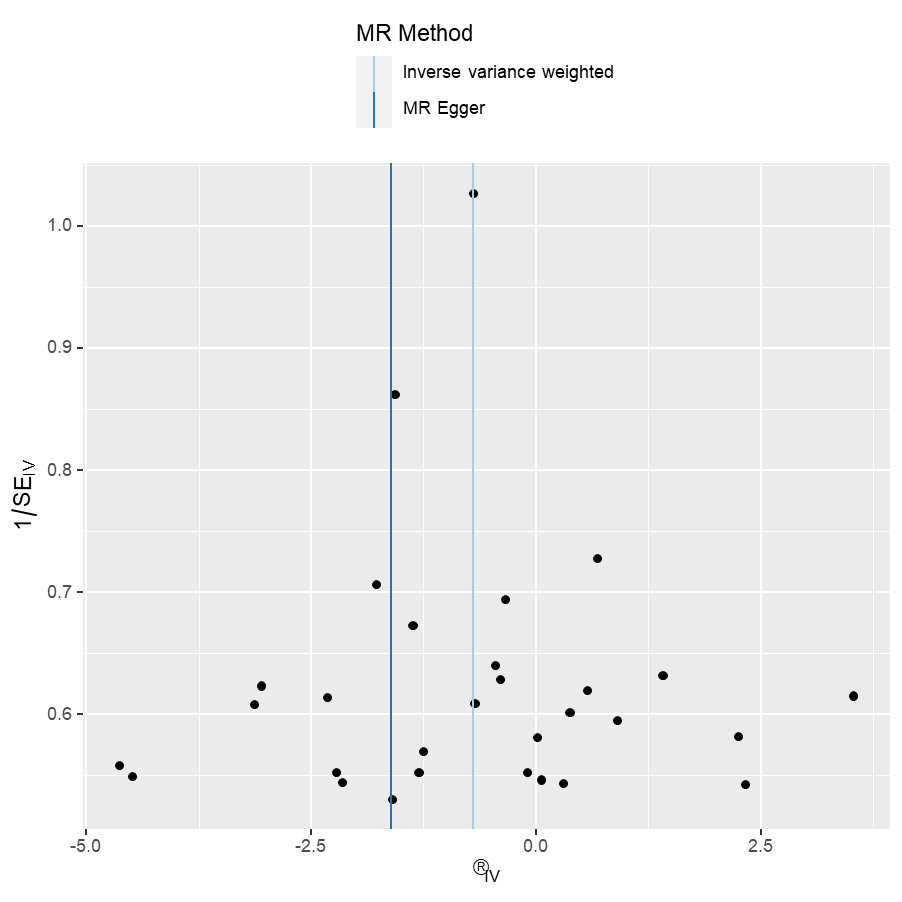
**

**Sup Fig. 7 Scatter plot,** **leave-one-out plot and funnel plot for the causal association between daytime dozing and *genus Oxalobacter*.**

**
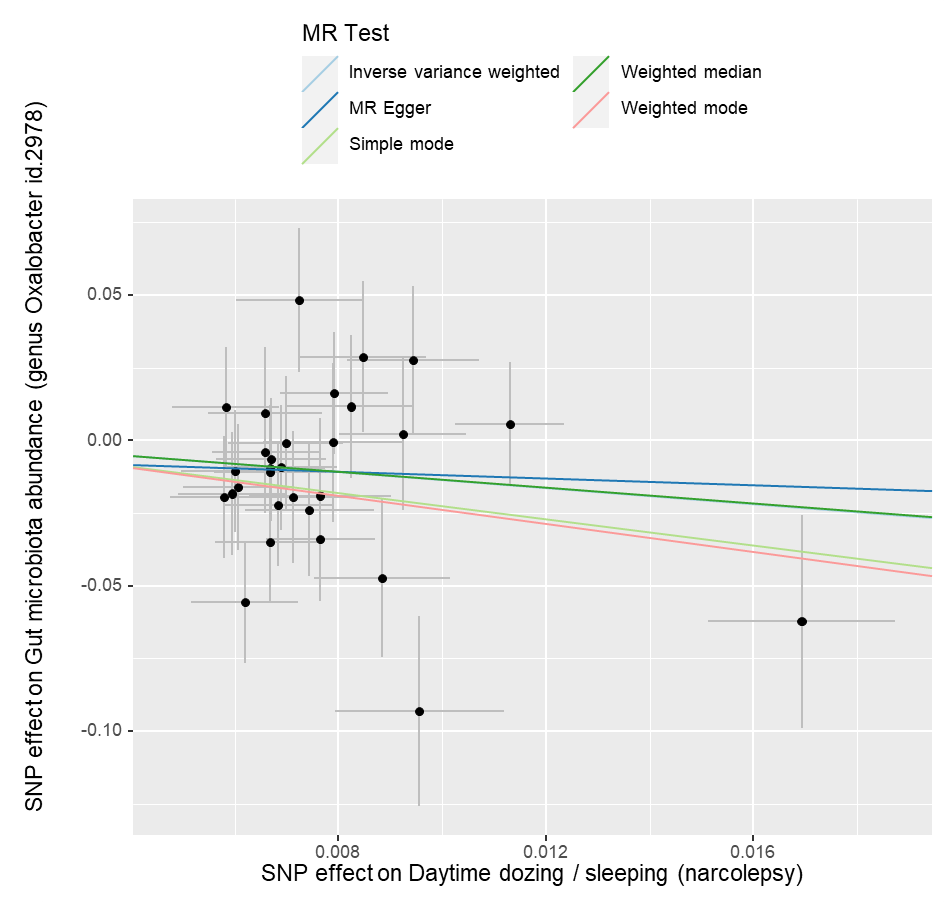

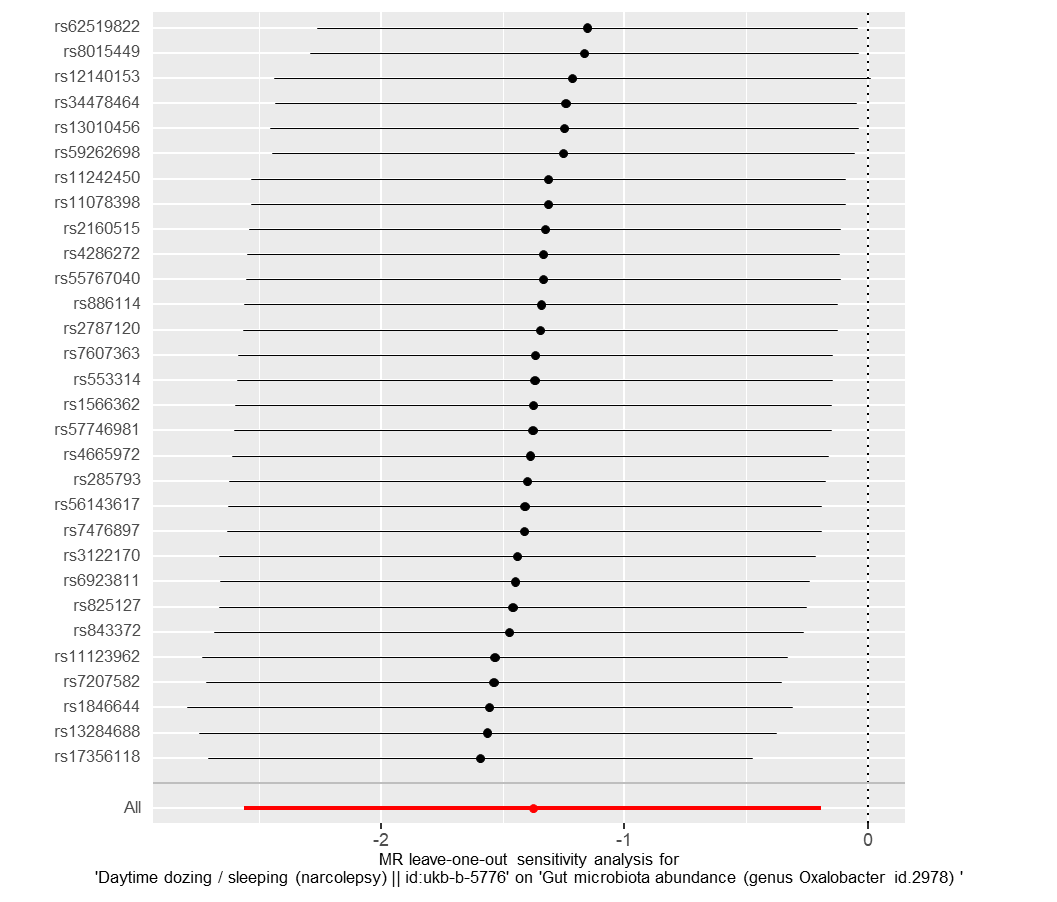

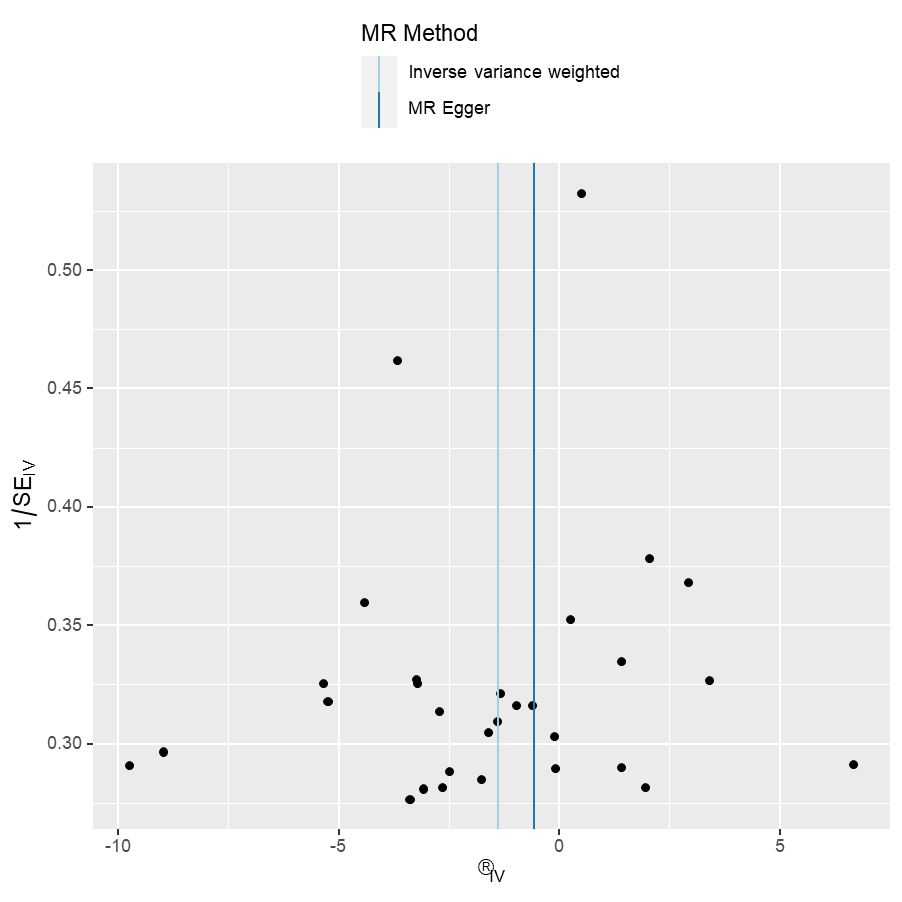
**

**Sup Fig. 8 Scatter plot,** **leave-one-out plot and funnel plot for the causal association between daytime dozing and *genus Slackia*.**


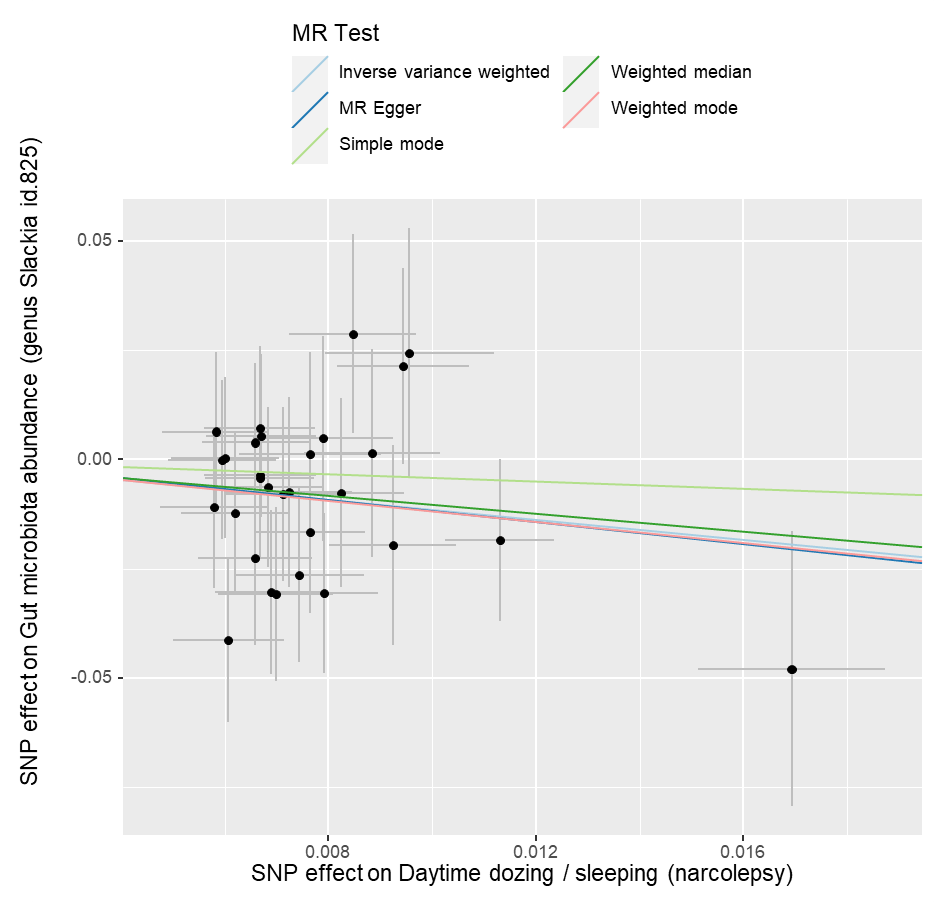

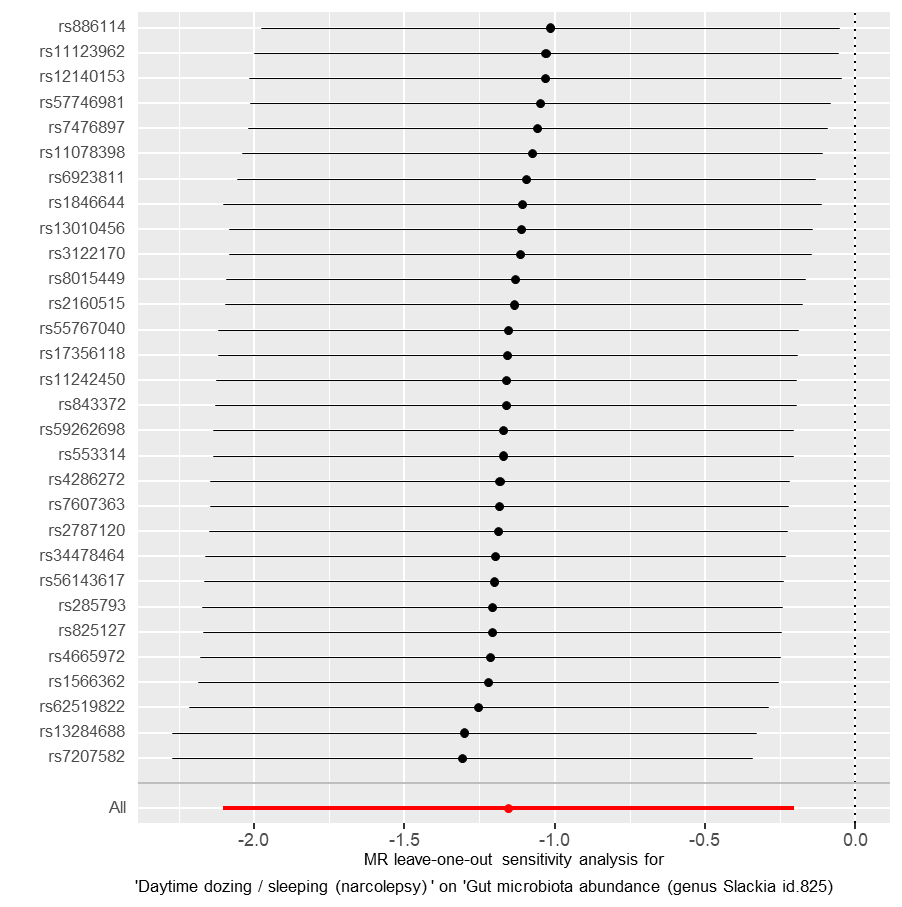

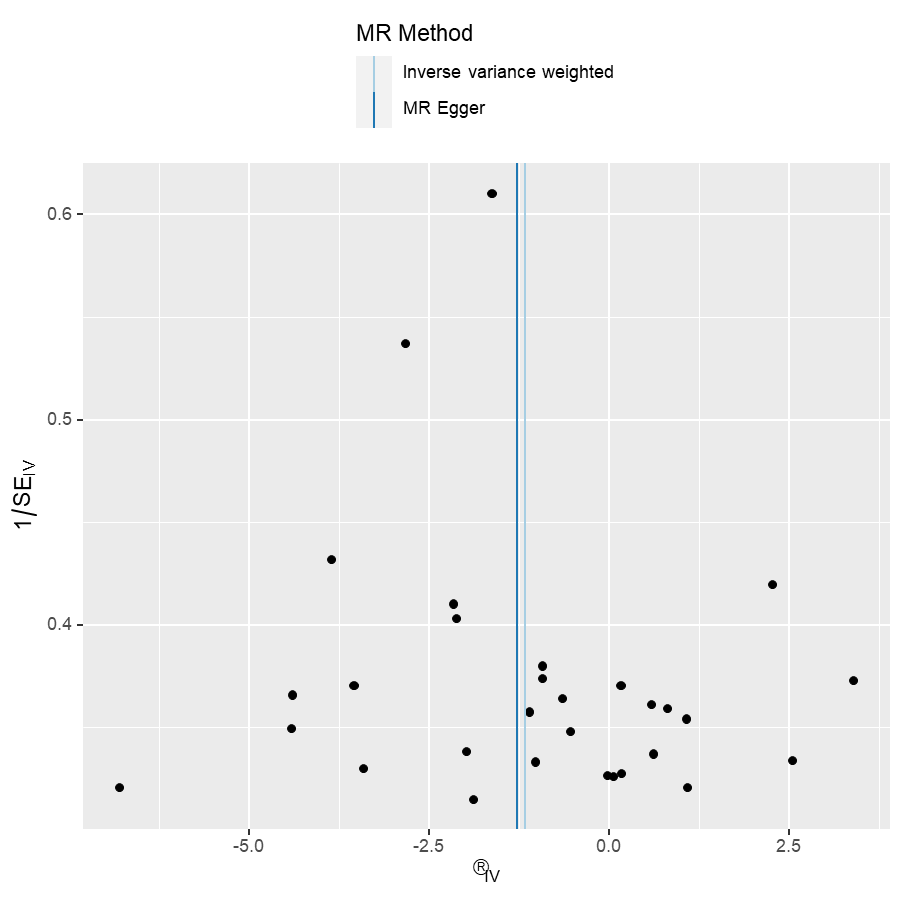


**Sup Fig. 9 Scatter plot,** **leave-one-out plot and funnel plot for the causal association between getting up in morning and *class Bacteroidia*.**


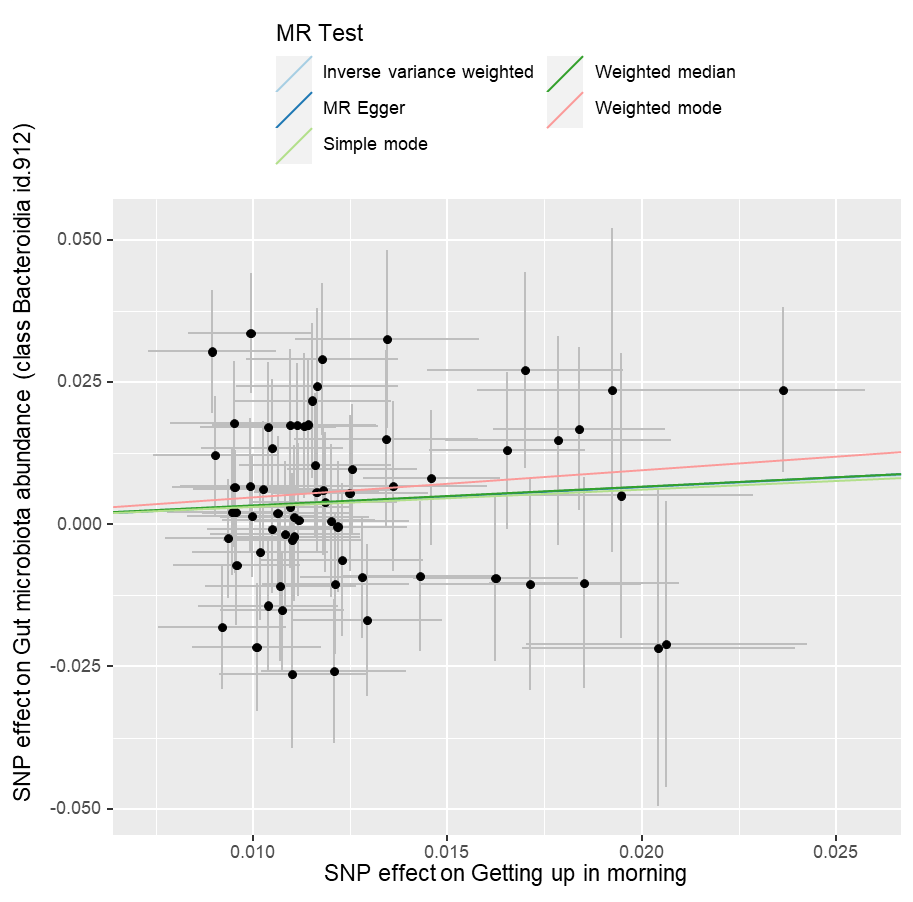

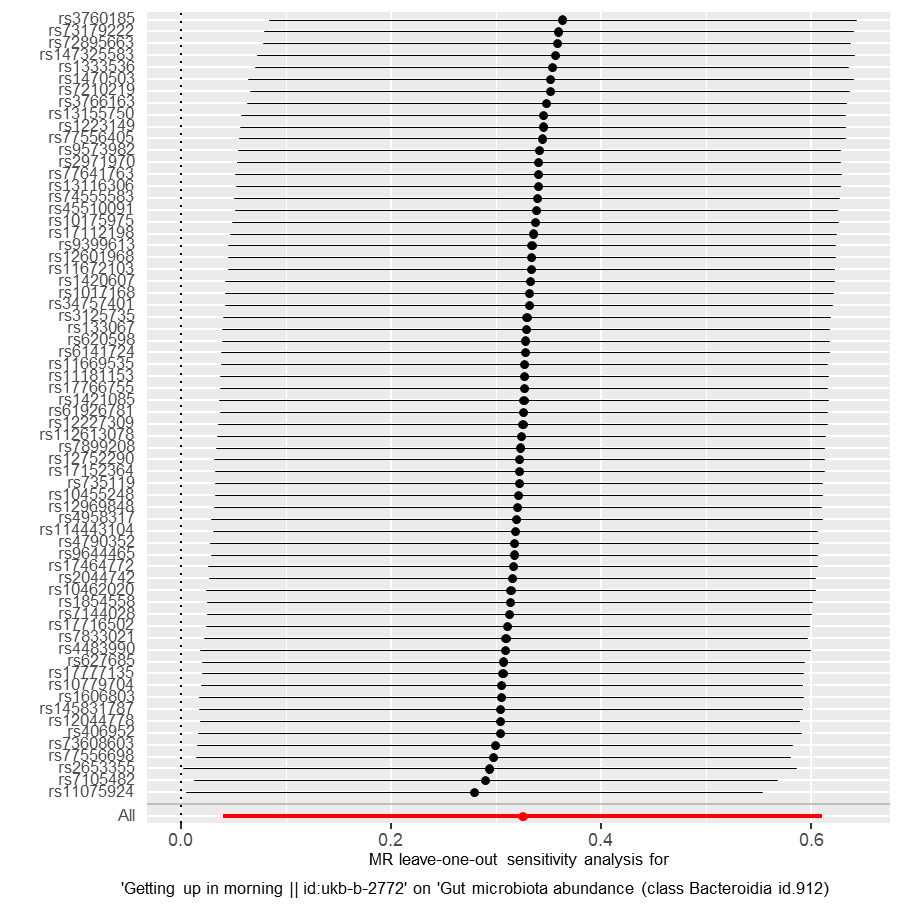

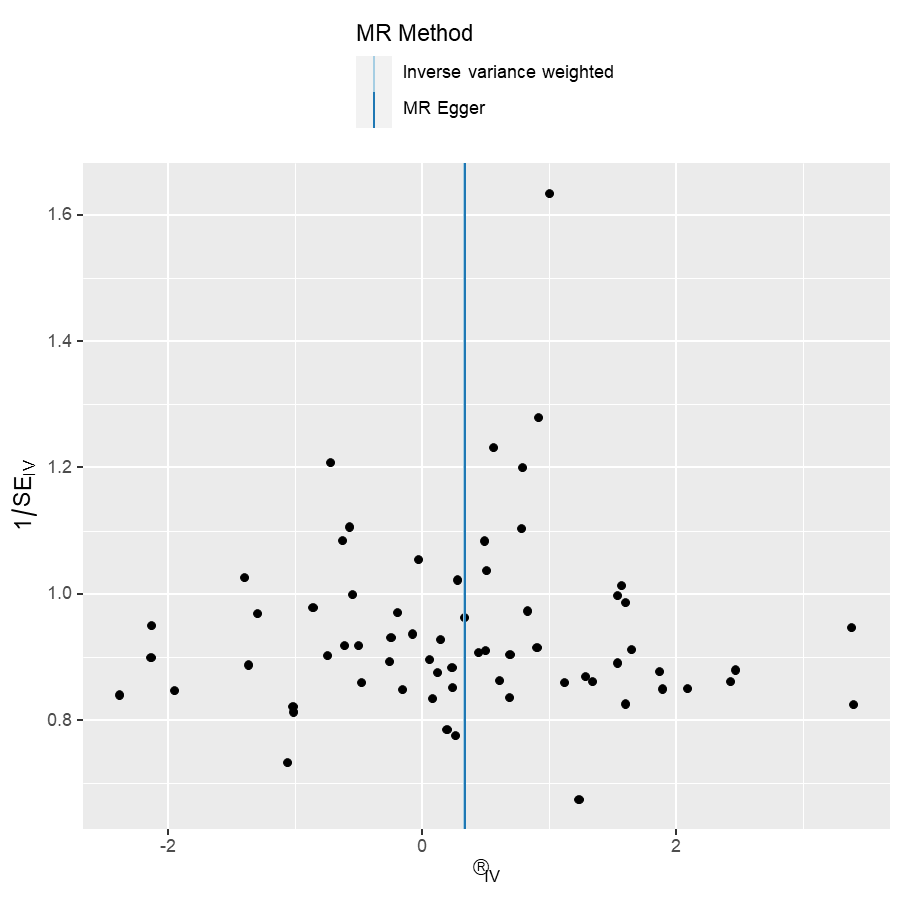


**Sup Fig. 10 Scatter plot,** **leave-one-out plot and funnel plot for the causal association between getting up in morning and *genus Clostridium innocuum group*.**

**
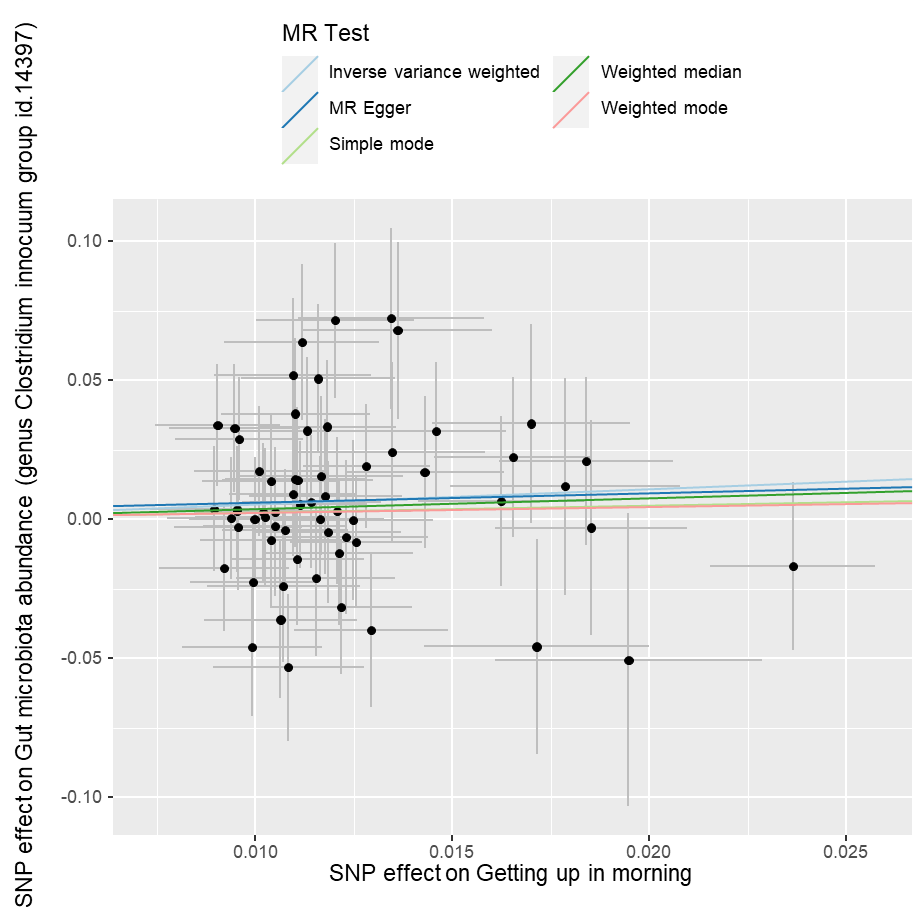

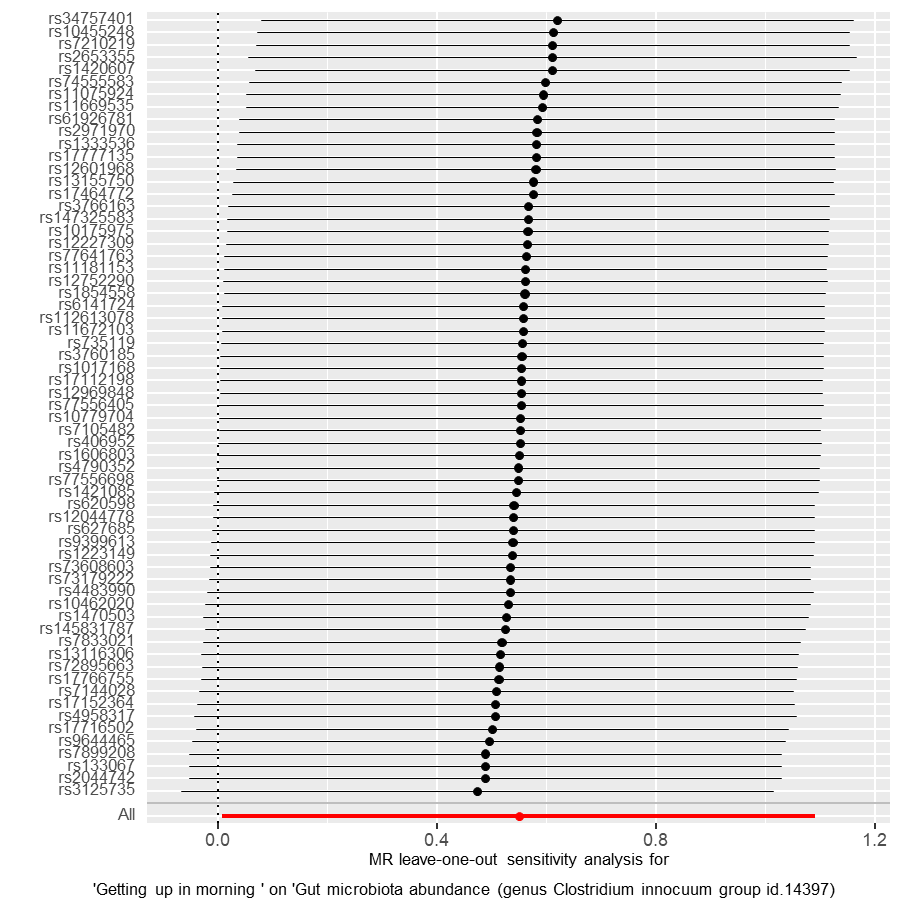

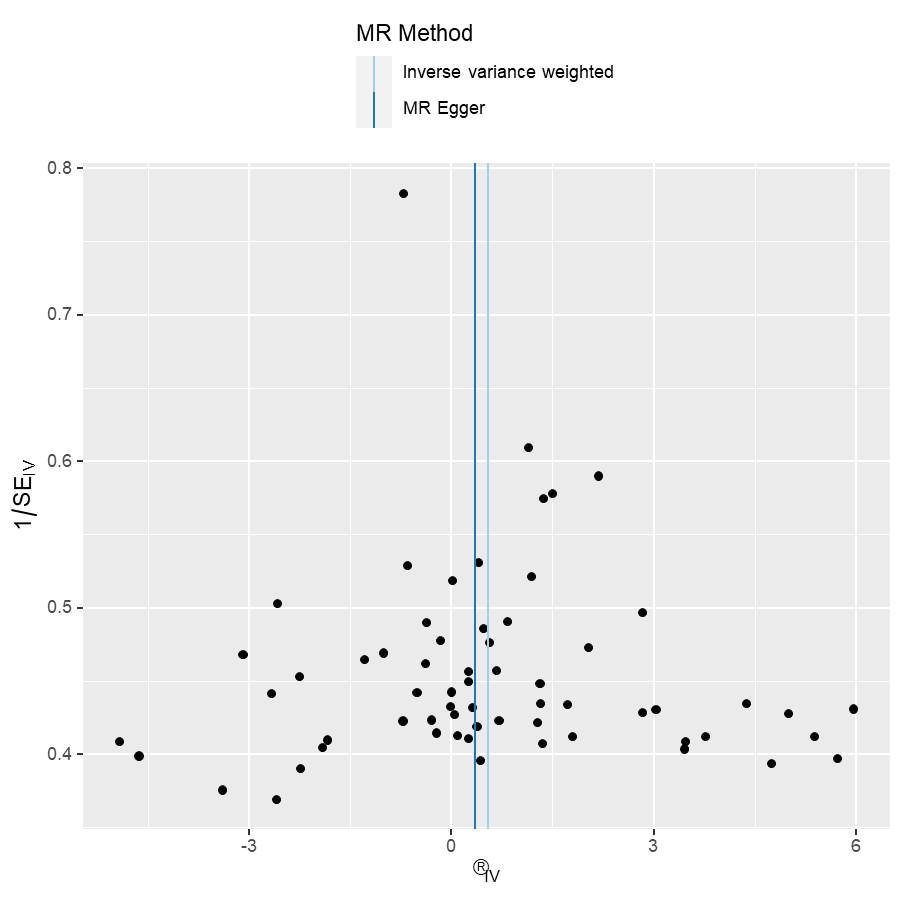
**

**Sup Fig. 11 Scatter plot,** **leave-one-out plot and funnel plot for the causal association between getting up in morning and *genus Intestinimonas*.**

**
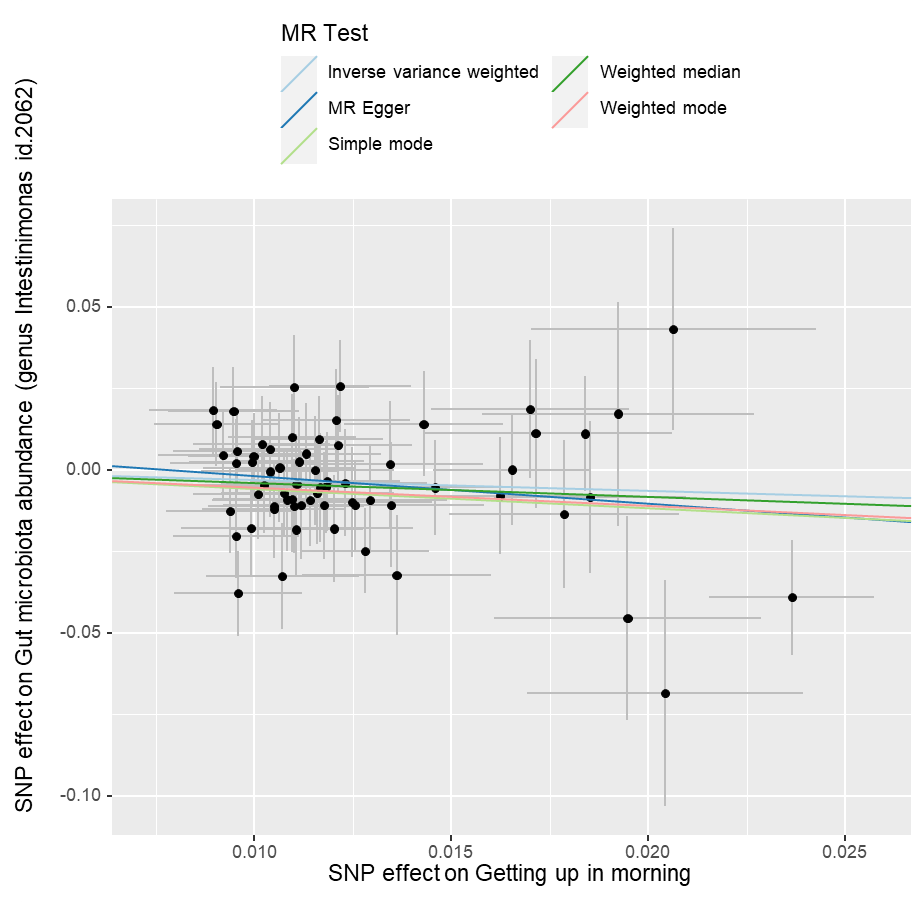

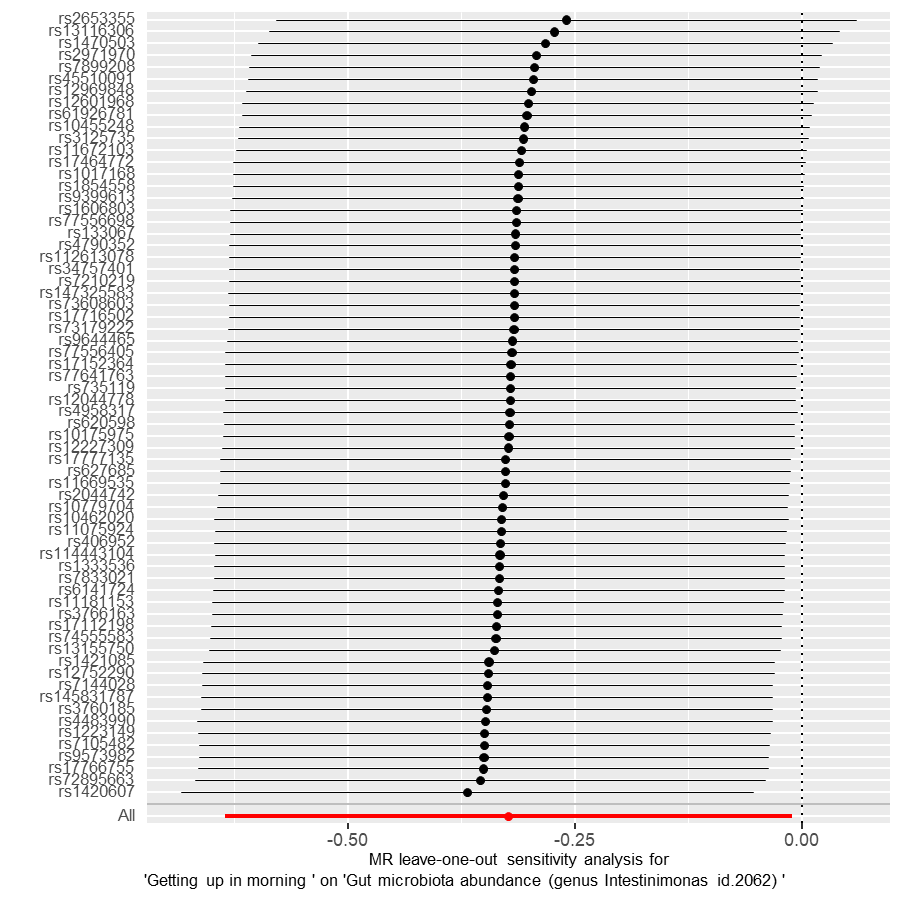

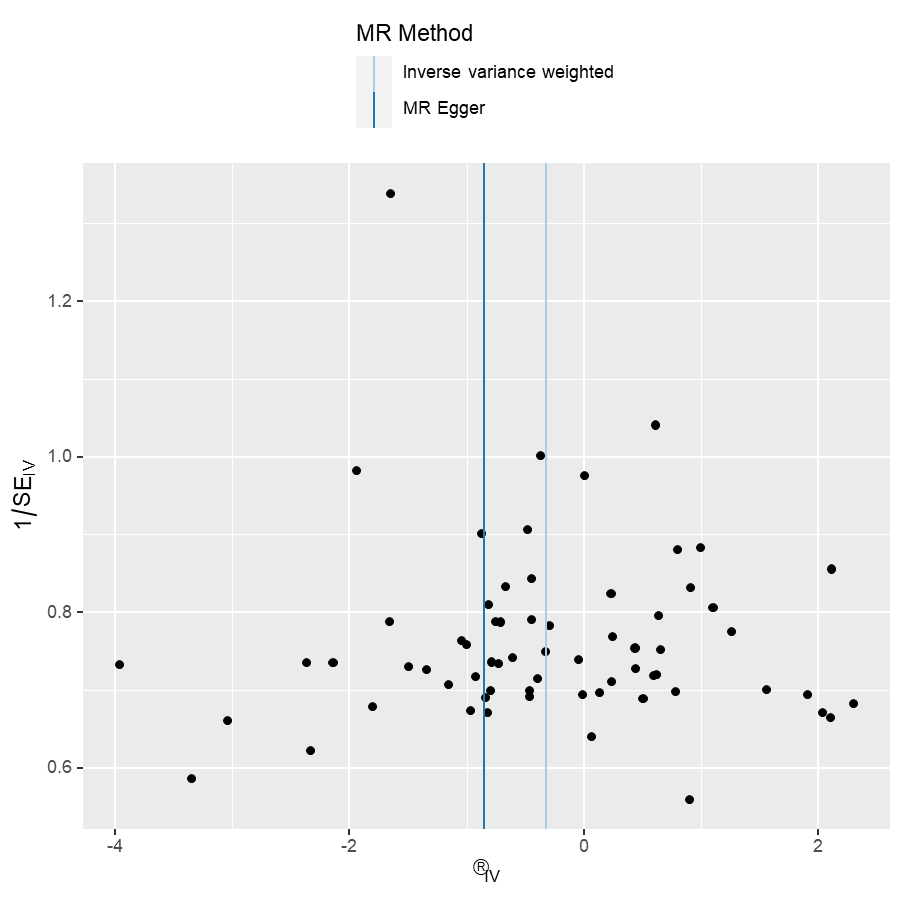
**

**Sup Fig. 12 Scatter plot,** **leave-one-out plot and funnel plot for the causal association between getting up in morning and *genus Slackia*.**

**
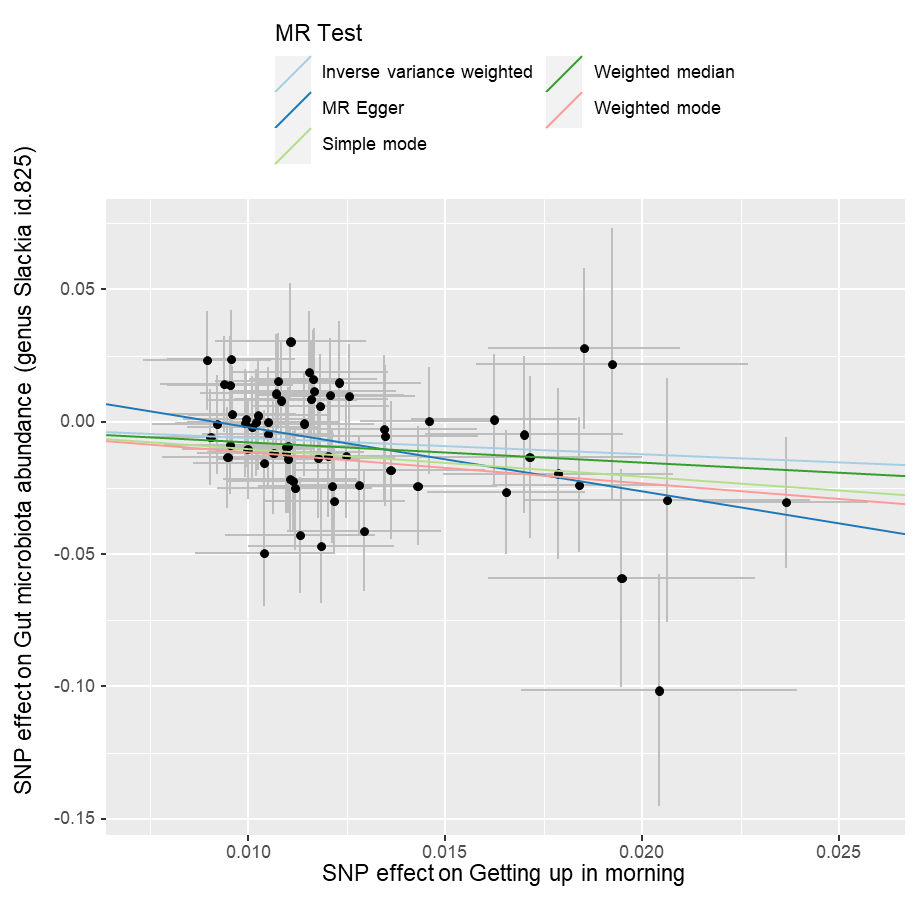

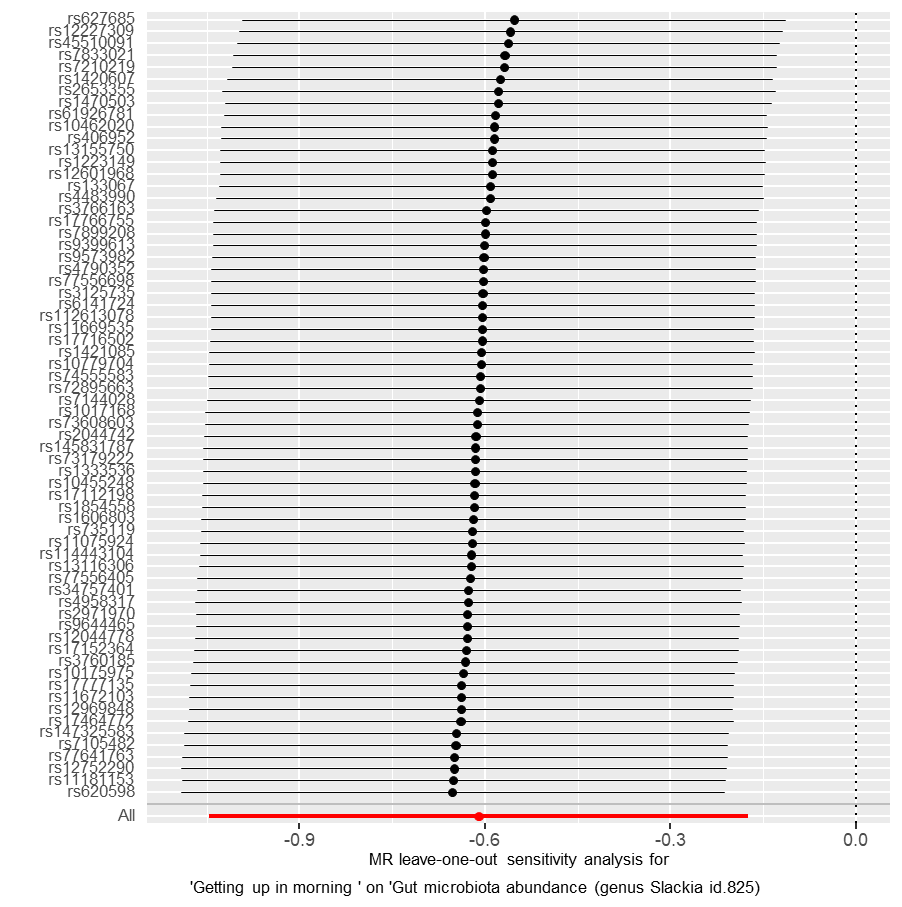

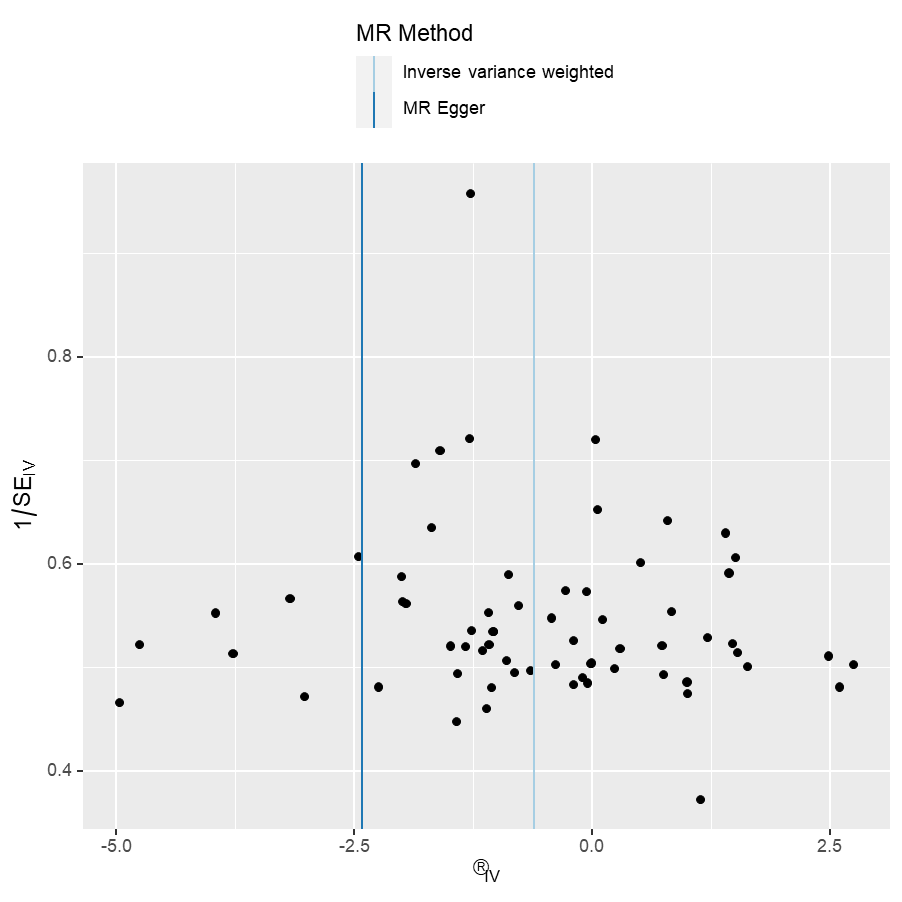
**

**Sup Fig. 13 Scatter plot,** **leave-one-out plot and funnel plot for the causal association between getting up in morning and *genus Terrisporobacter*.**

**
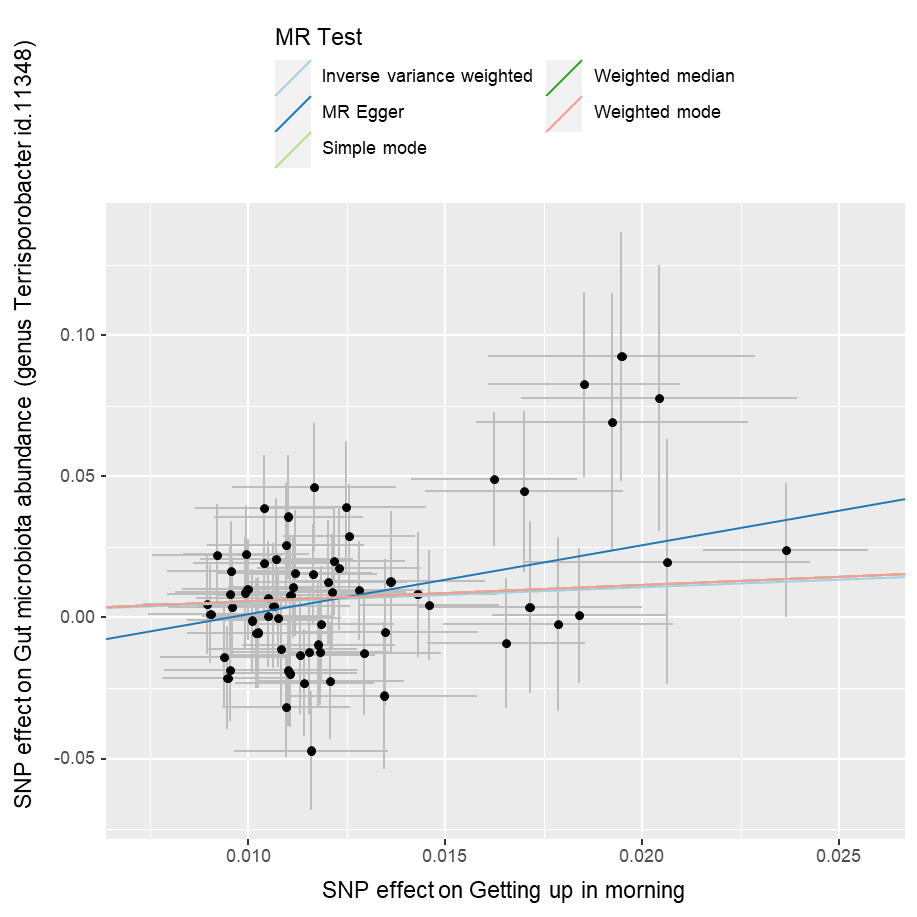

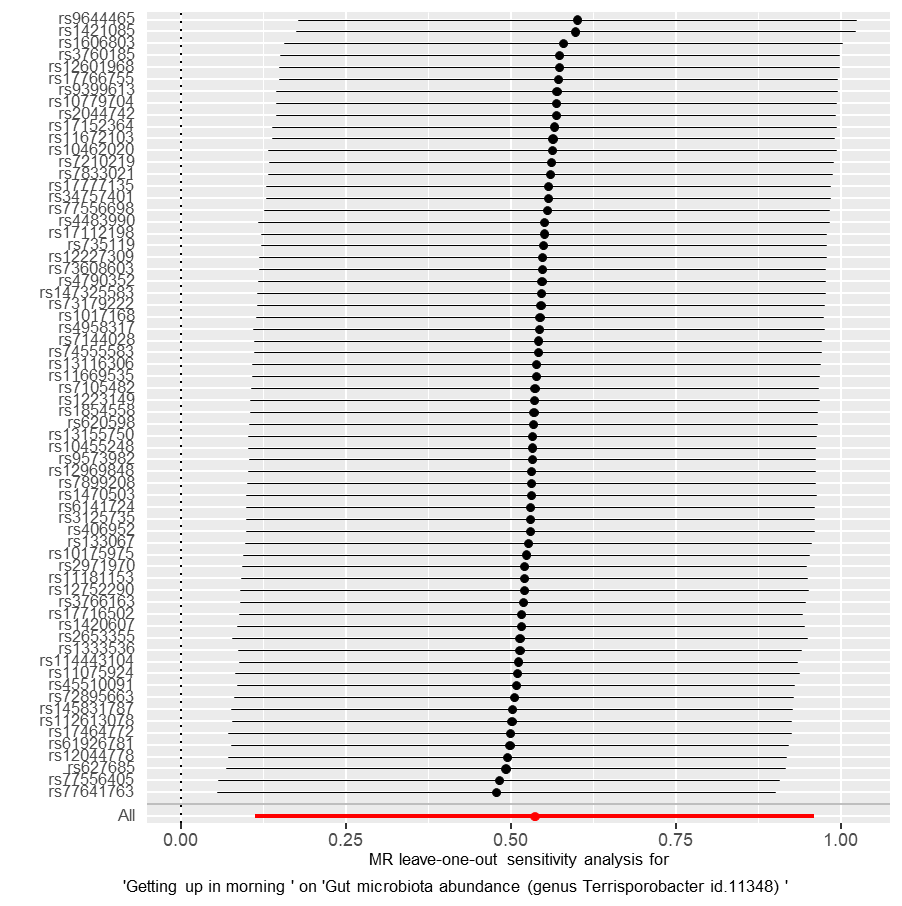

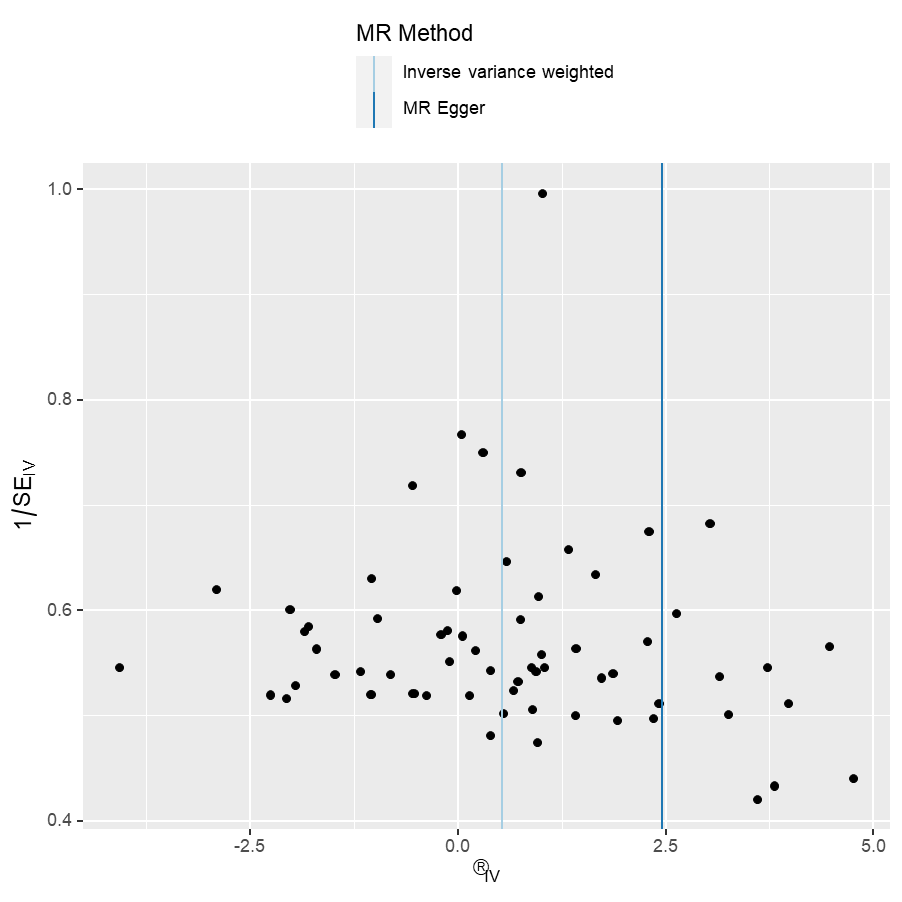
**

**Sup Fig. 14 Scatter plot,** **leave-one-out plot and funnel plot for the causal association between getting up in morning and *order Bacteroidales*.**

**
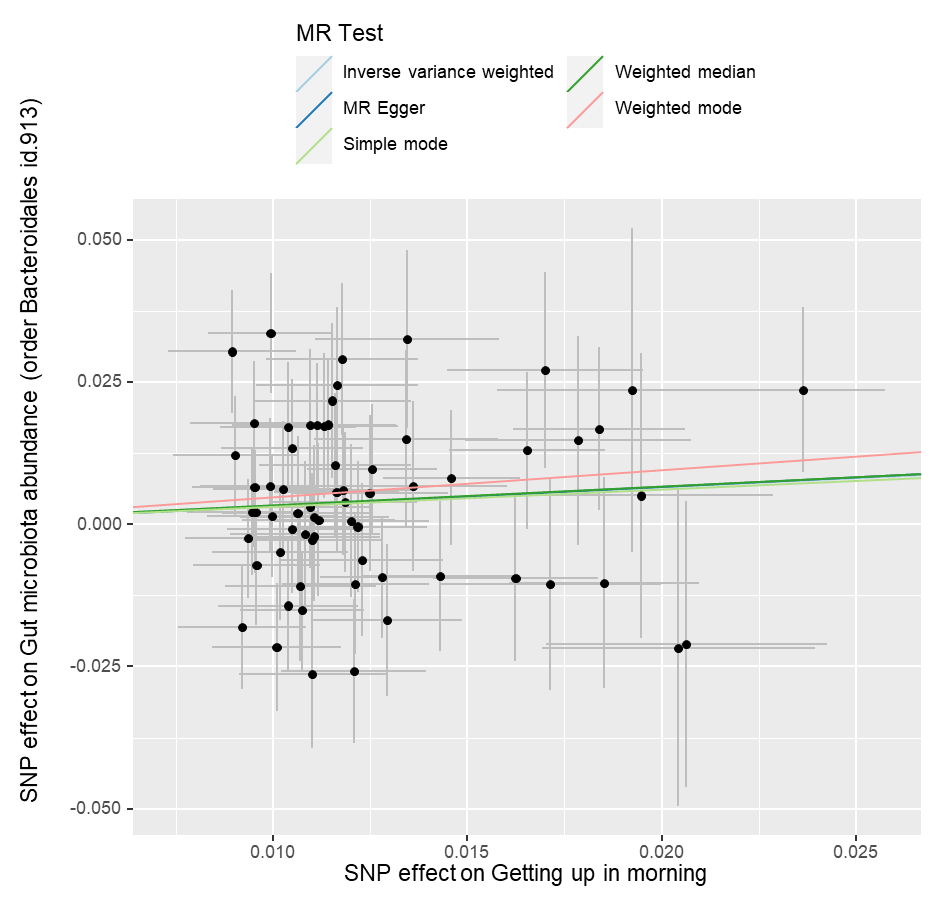

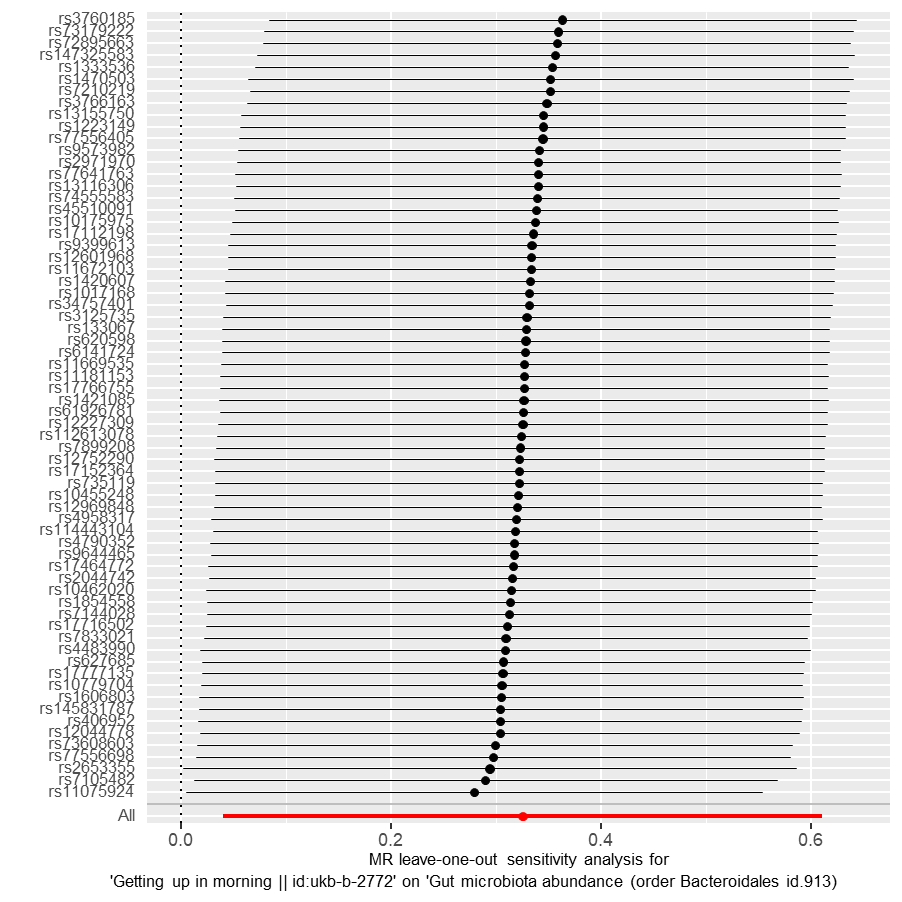

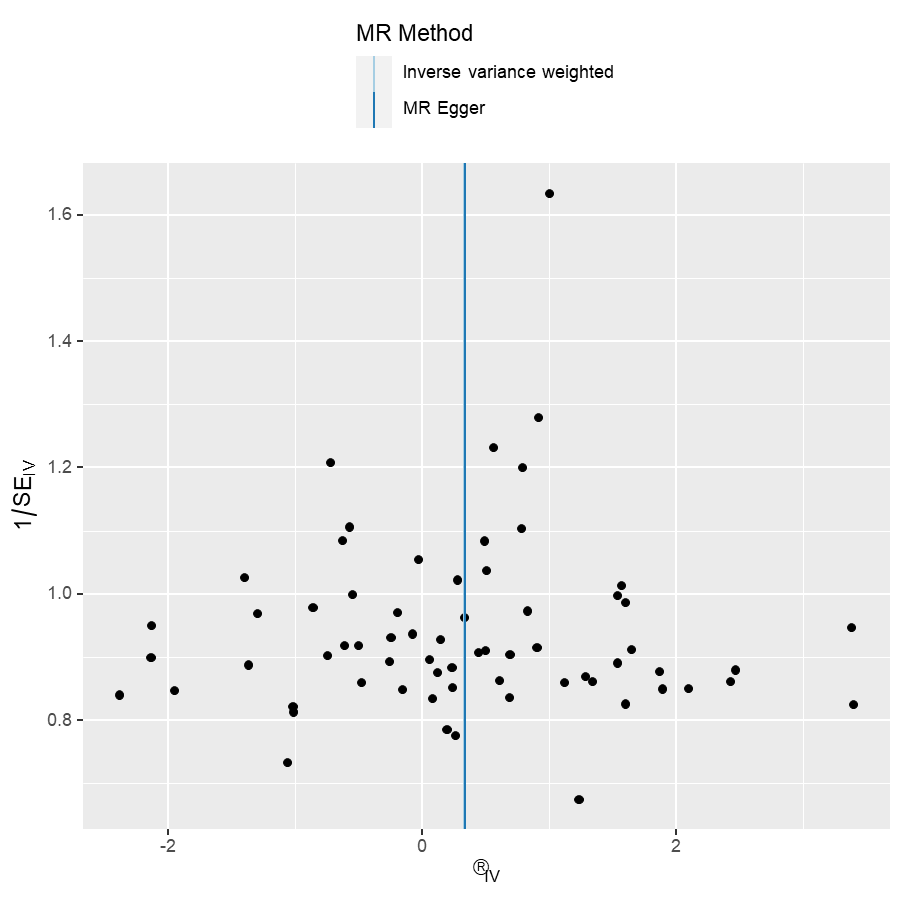
**

**Sup Fig. 15 Scatter plot,** **leave-one-out plot and funnel plot for the causal association between getting up in morning and *phylum Bacteroidetes*.**

**
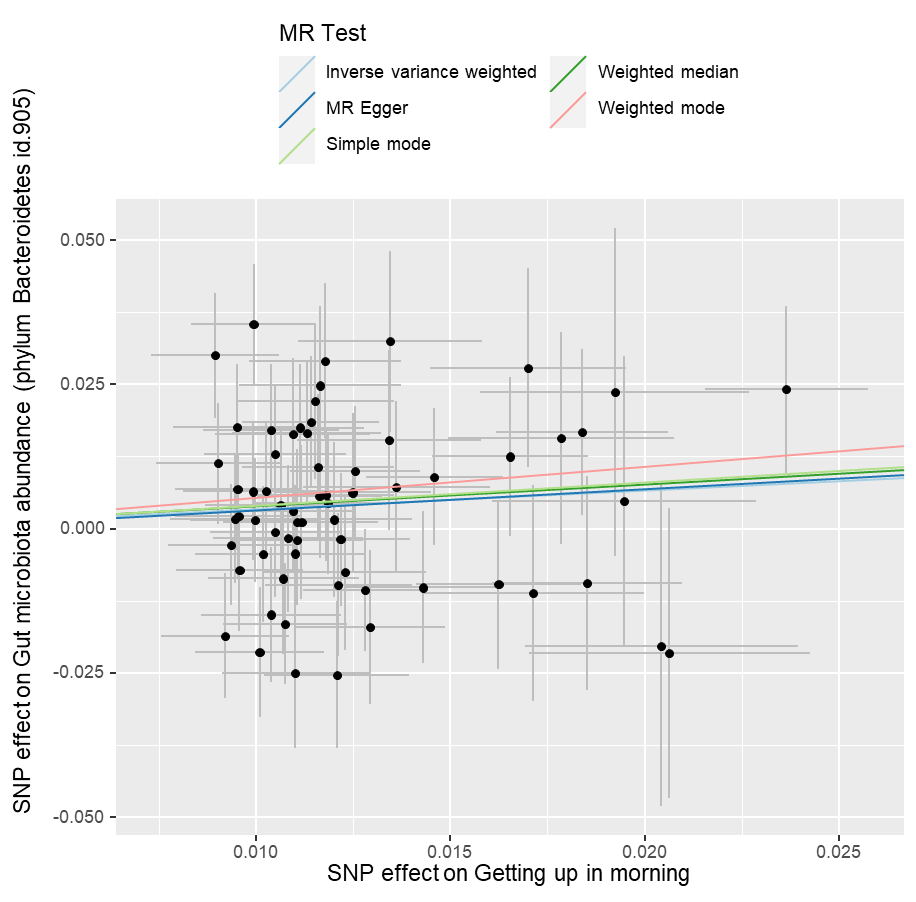

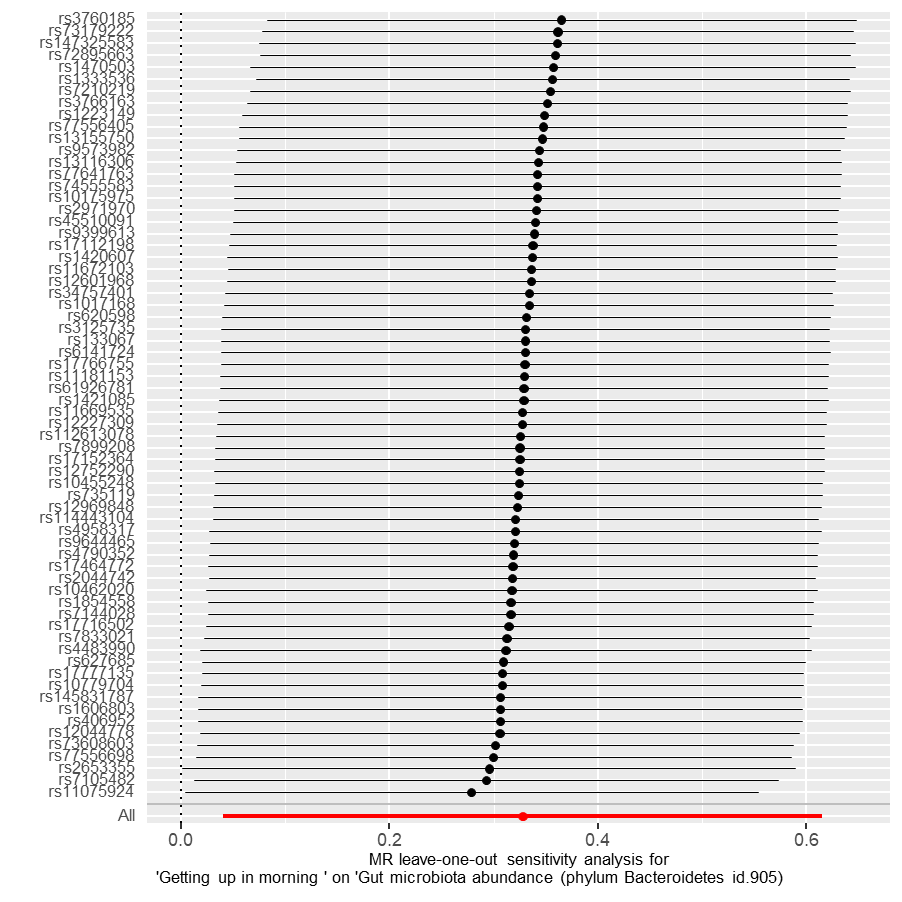

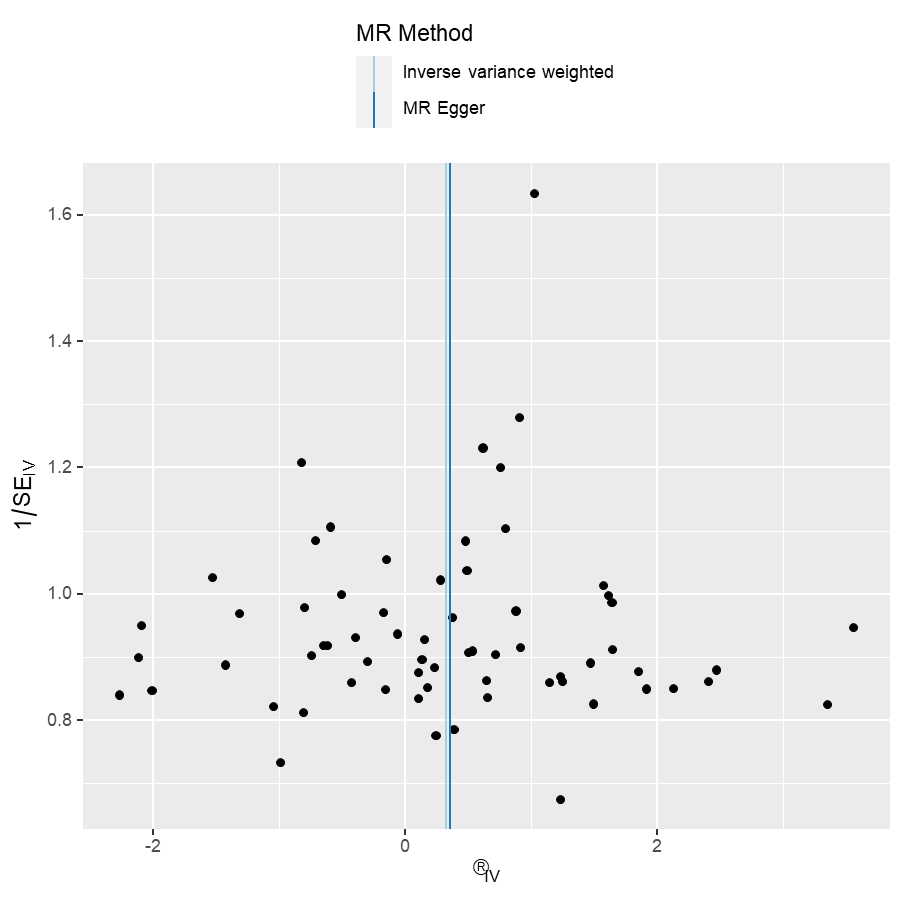
**

**Sup Fig. 16 Scatter plot,** **leave-one-out plot and funnel plot for the causal association between insomnia and *class Alphaproteobacteria*.**


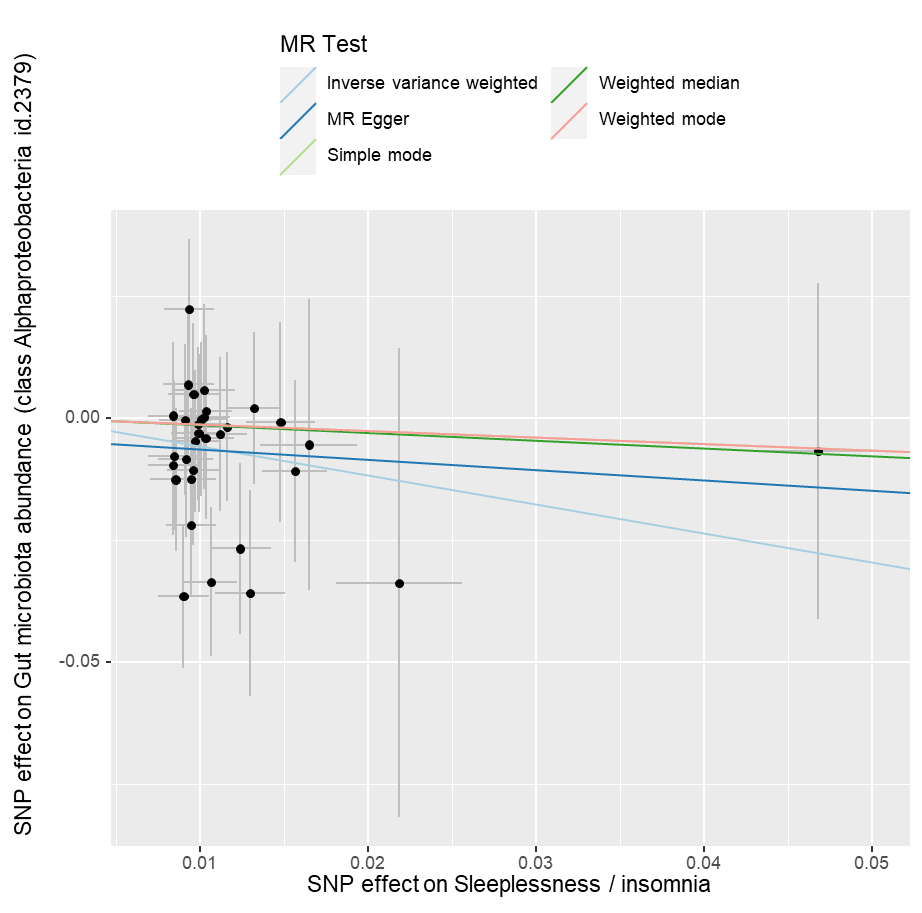

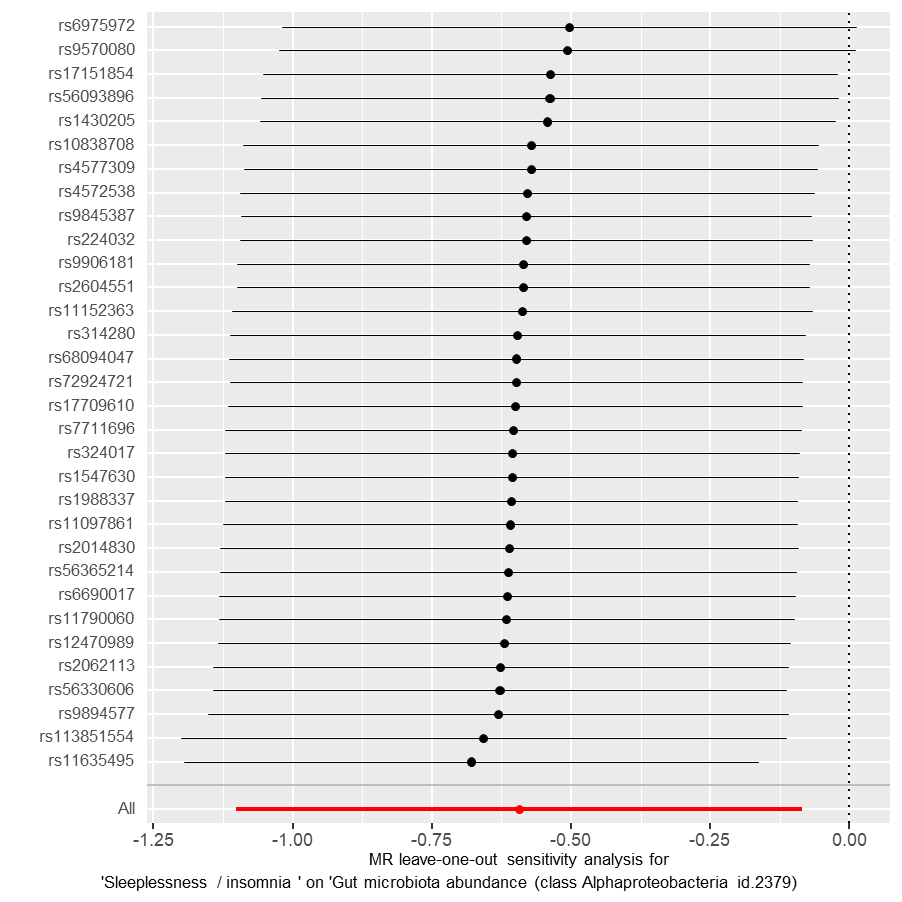

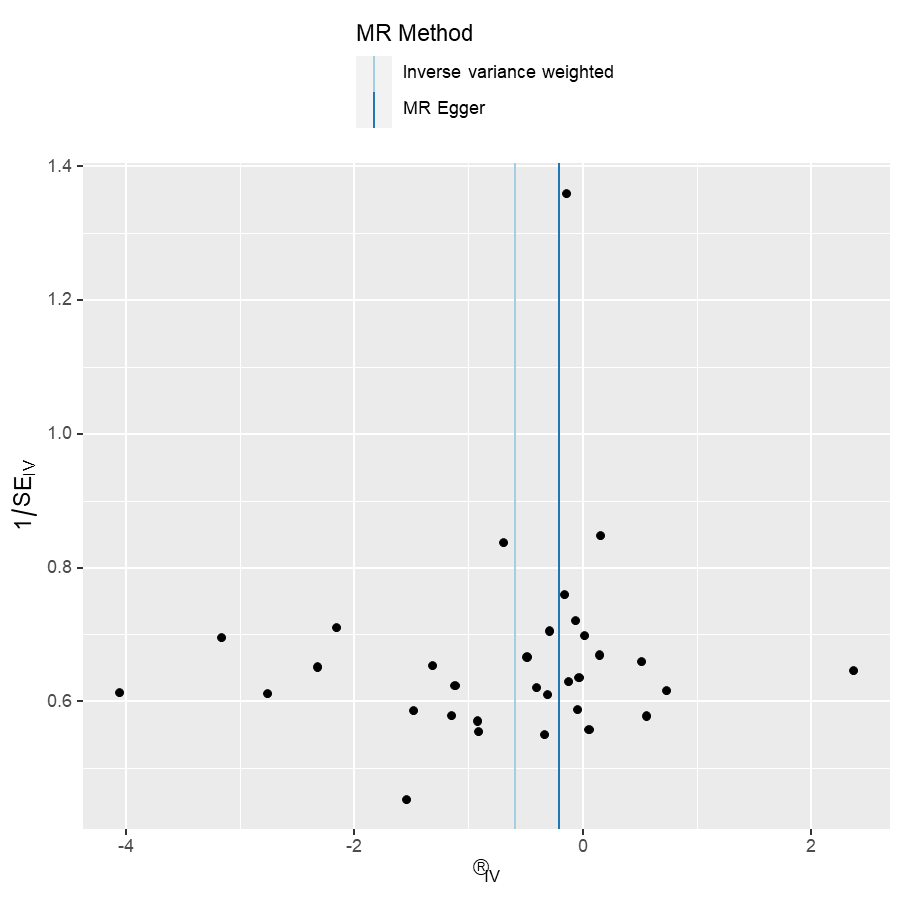


**Sup Fig. 17 Scatter plot,** **leave-one-out plot and funnel plot for the causal association between insomnia and *class Erysipelotrichia*.**

**
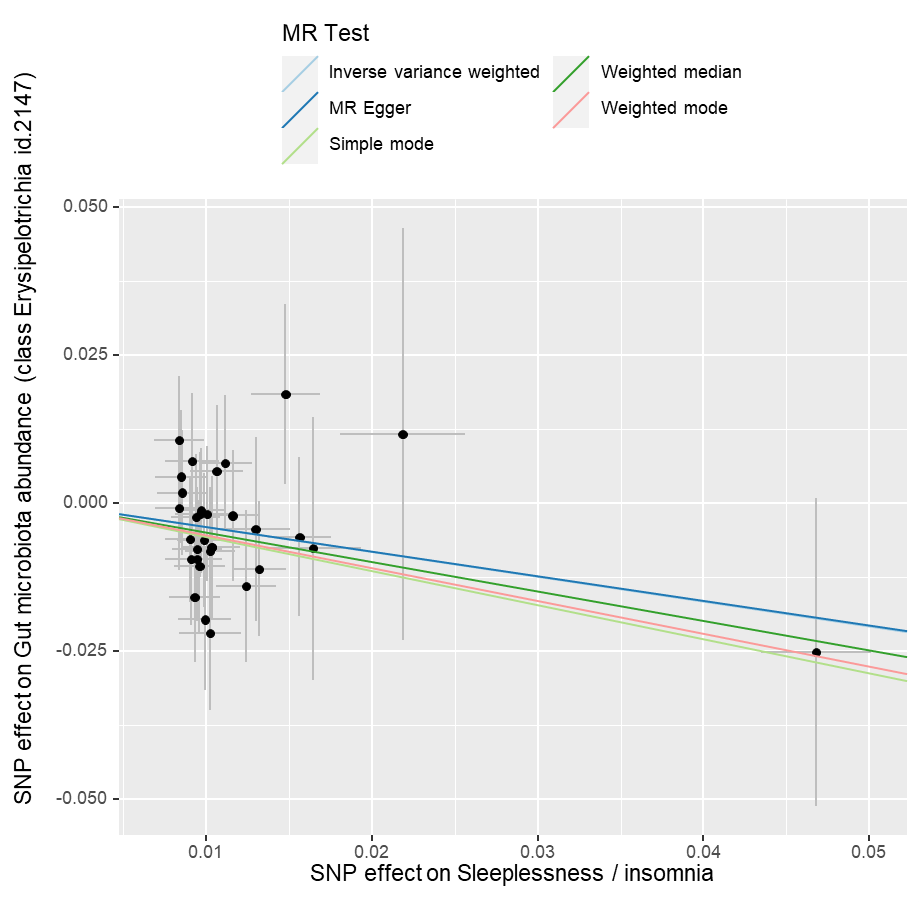

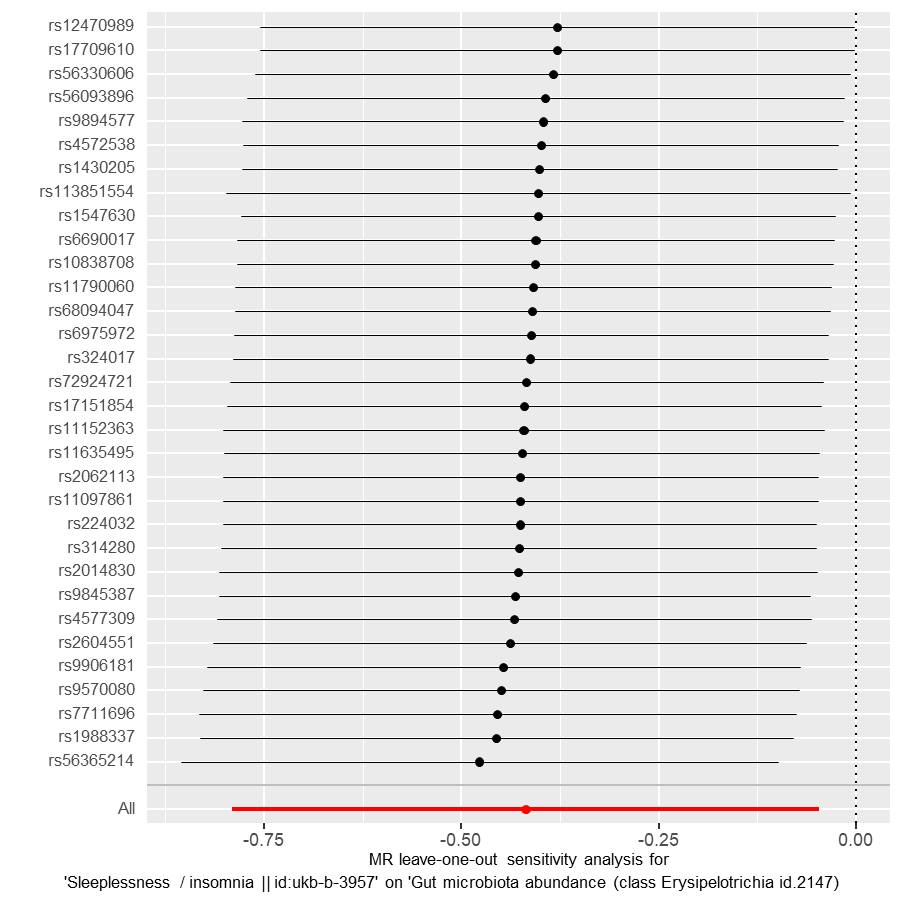

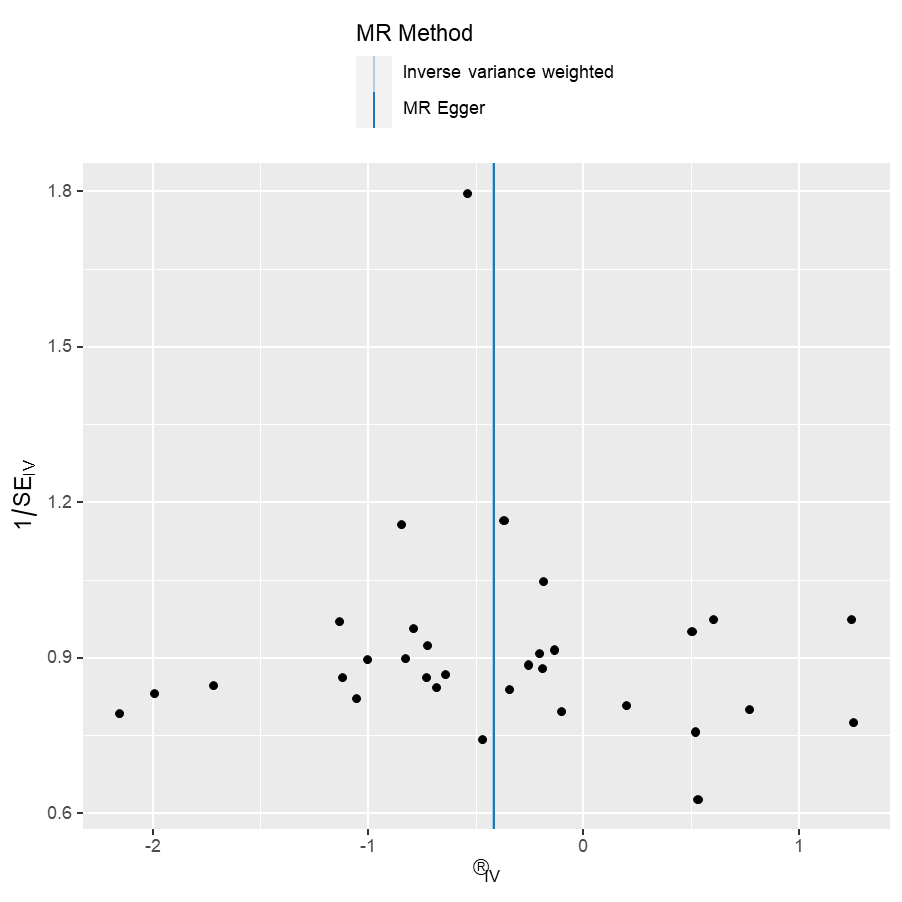
**

**Sup Fig. 18 Scatter plot,** **leave-one-out plot and funnel plot for the causal association between insomnia and *family Clostridiaceae1*.**

**
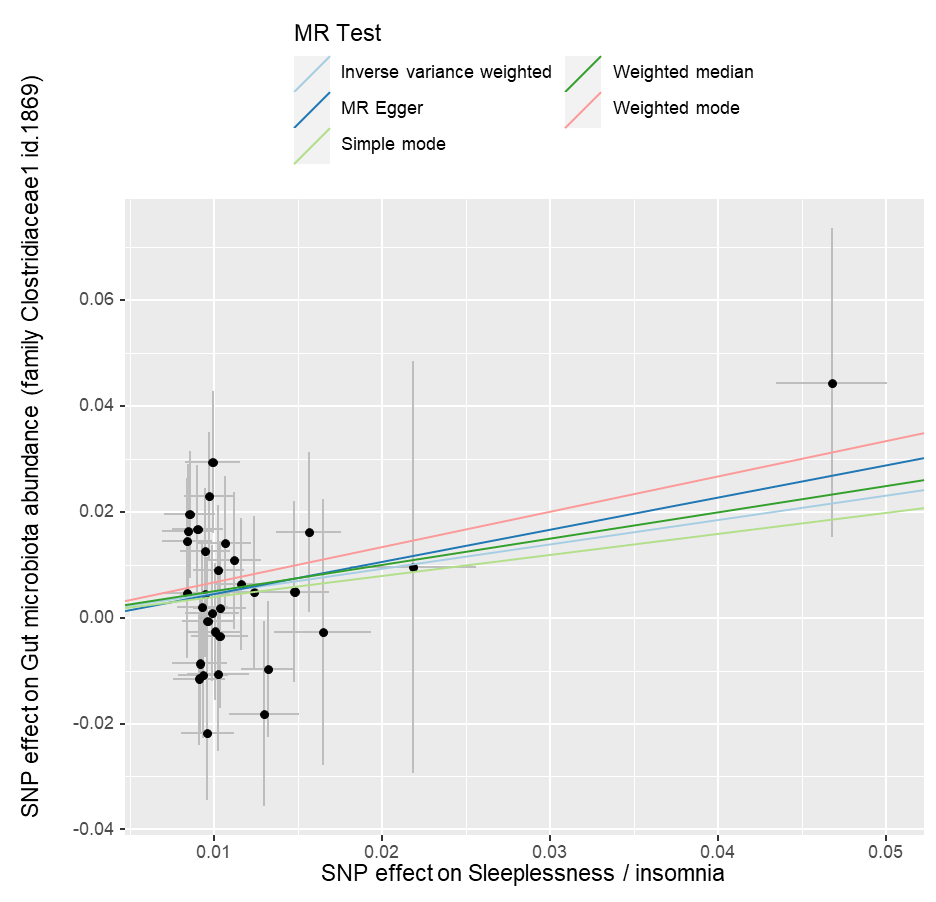

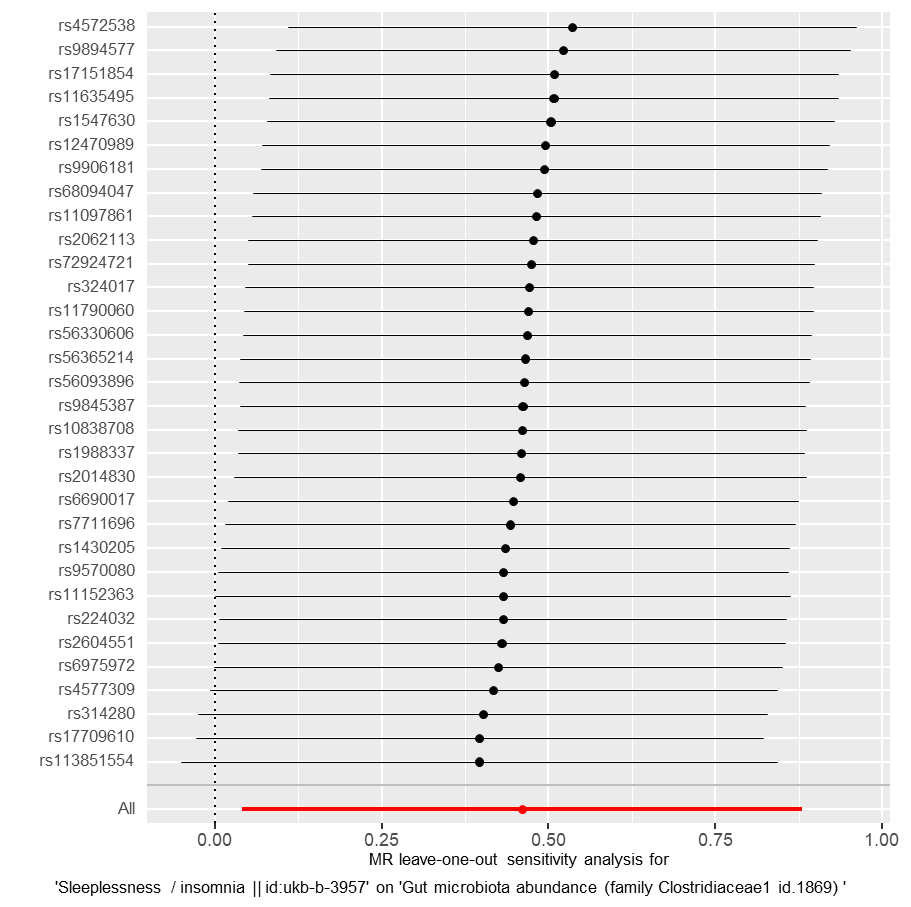

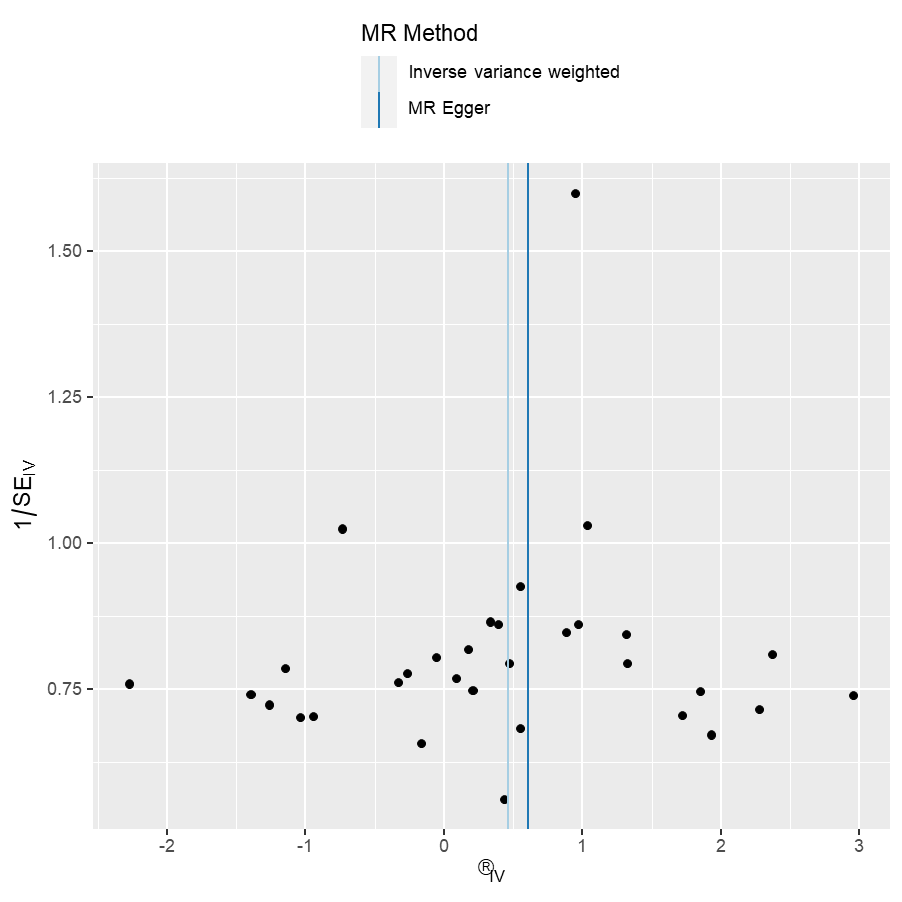
**

**Sup Fig. 19 Scatter plot,** **leave-one-out plot and funnel plot for the causal association between insomnia and *family Erysipelotrichaceae*.**

**
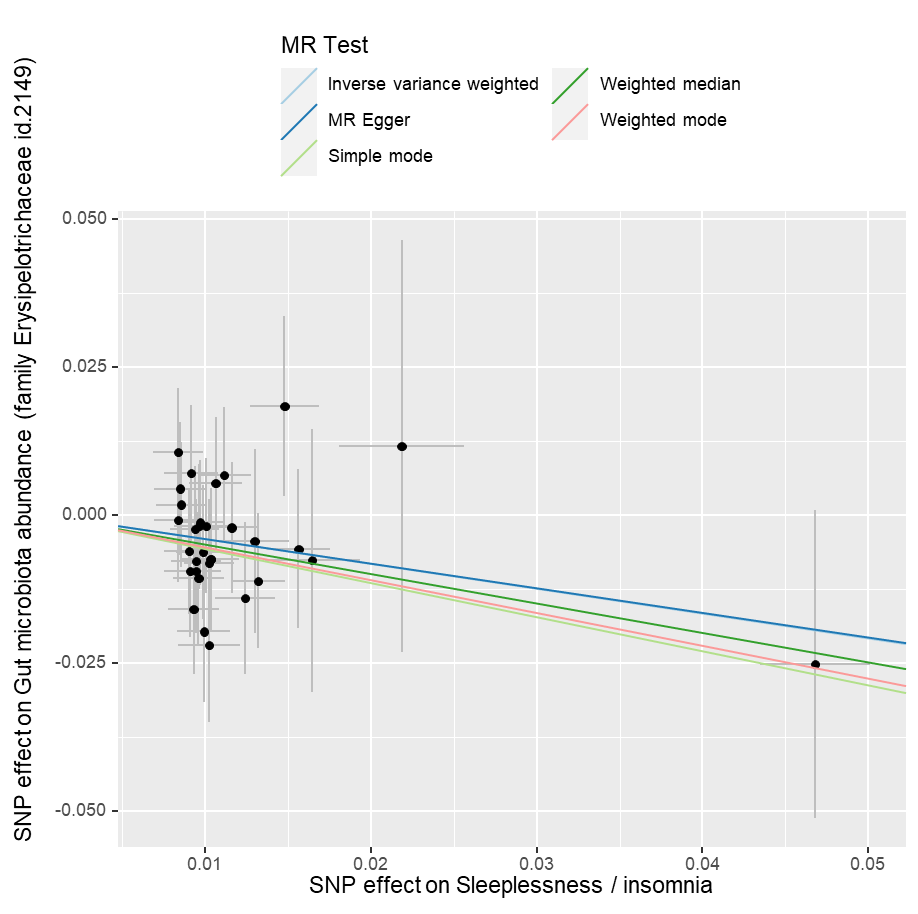

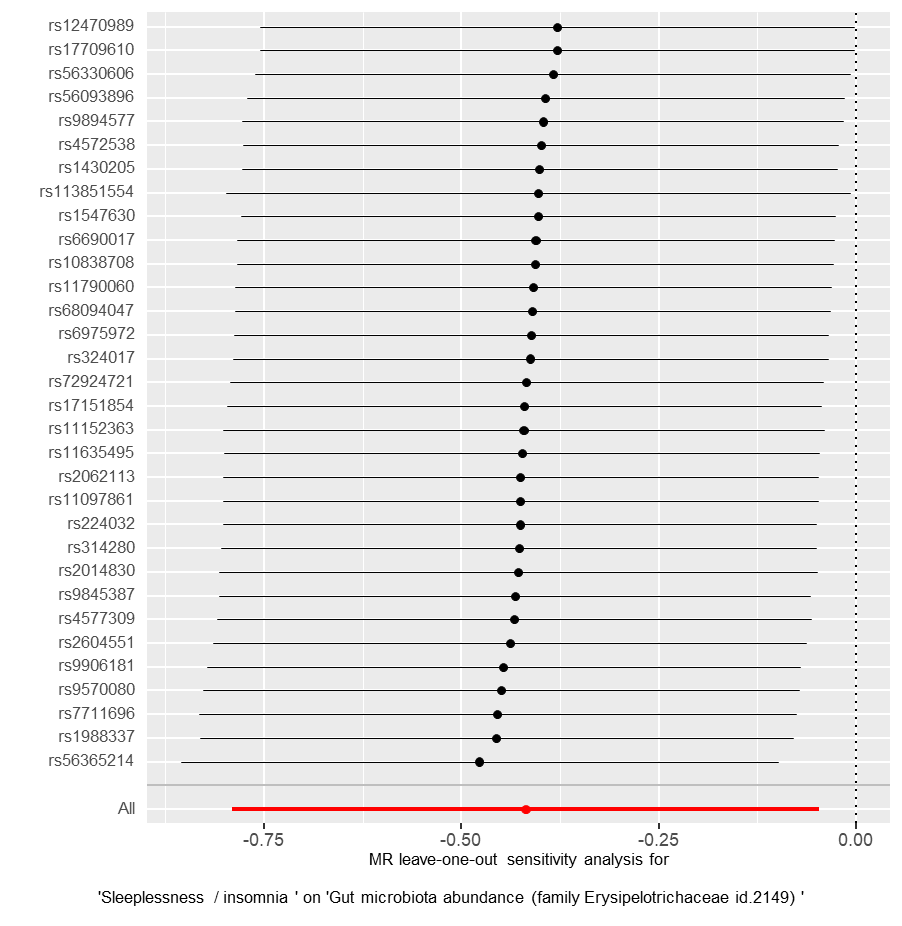

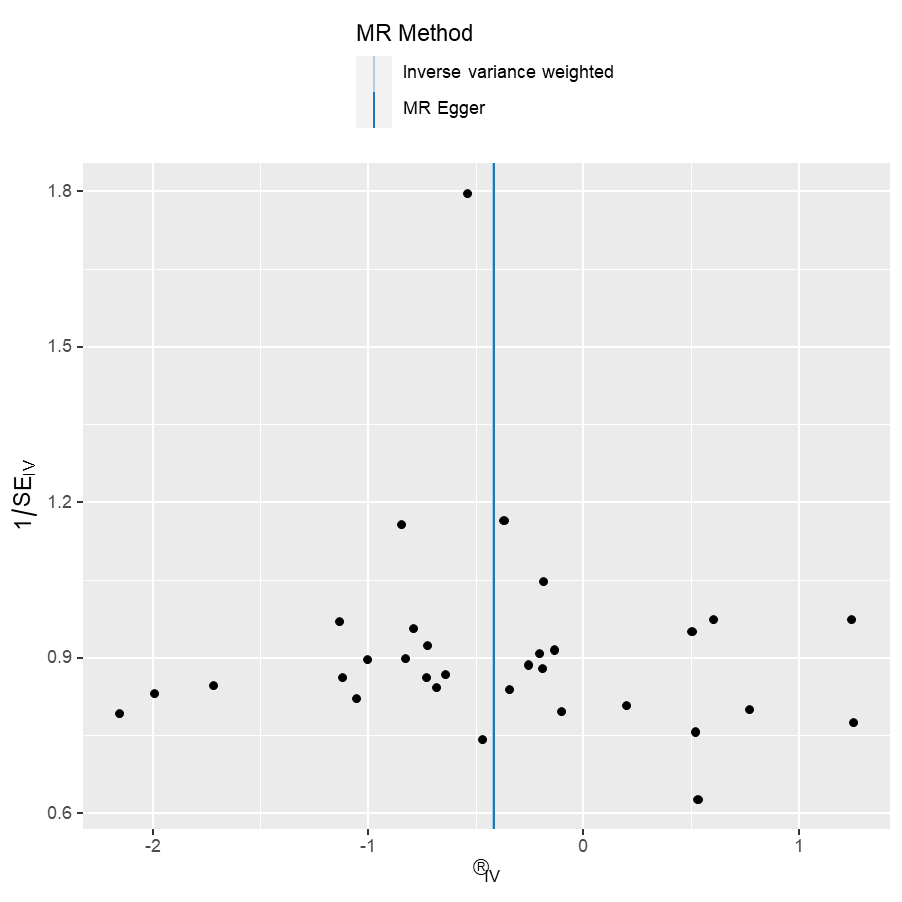
**

**Sup Fig. 20 Scatter plot,** **leave-one-out plot and funnel plot for the causal association between insomnia and *family Lachnospiraceae*.**

**
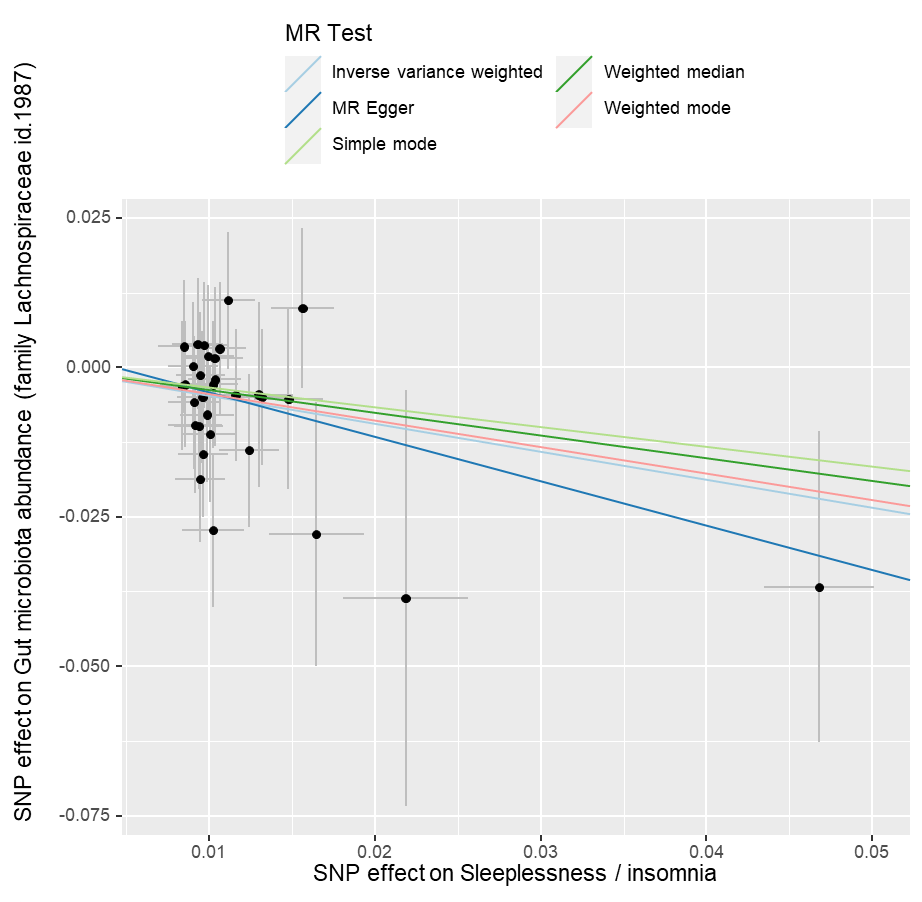

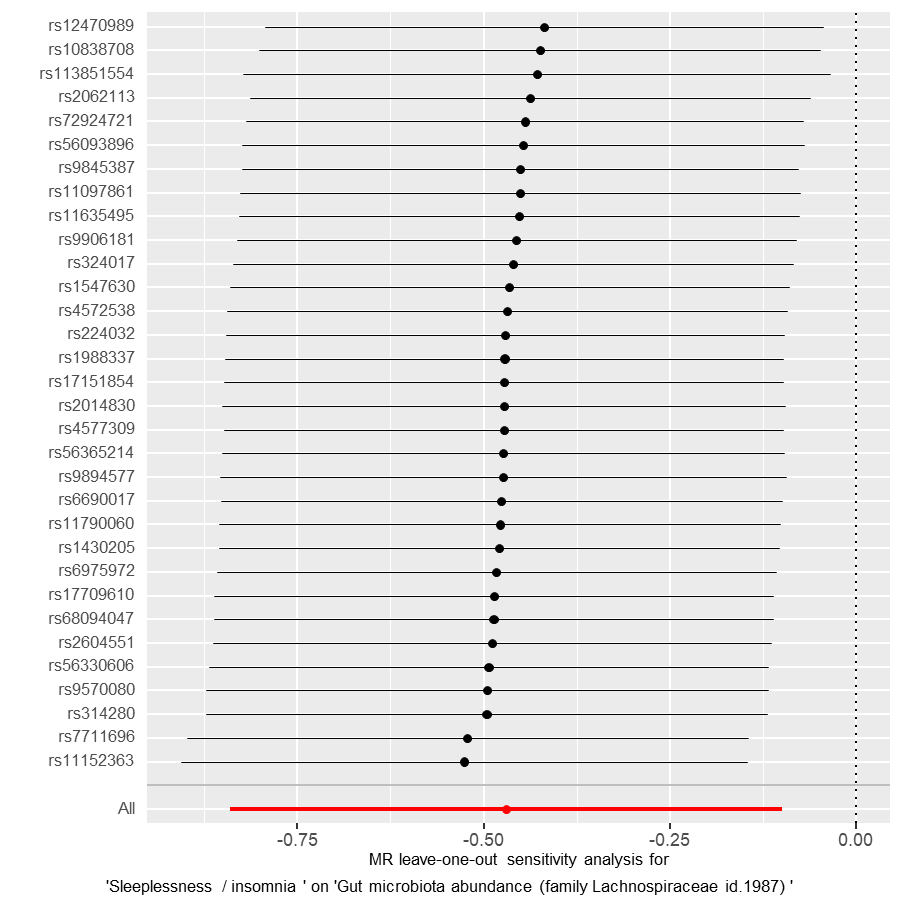

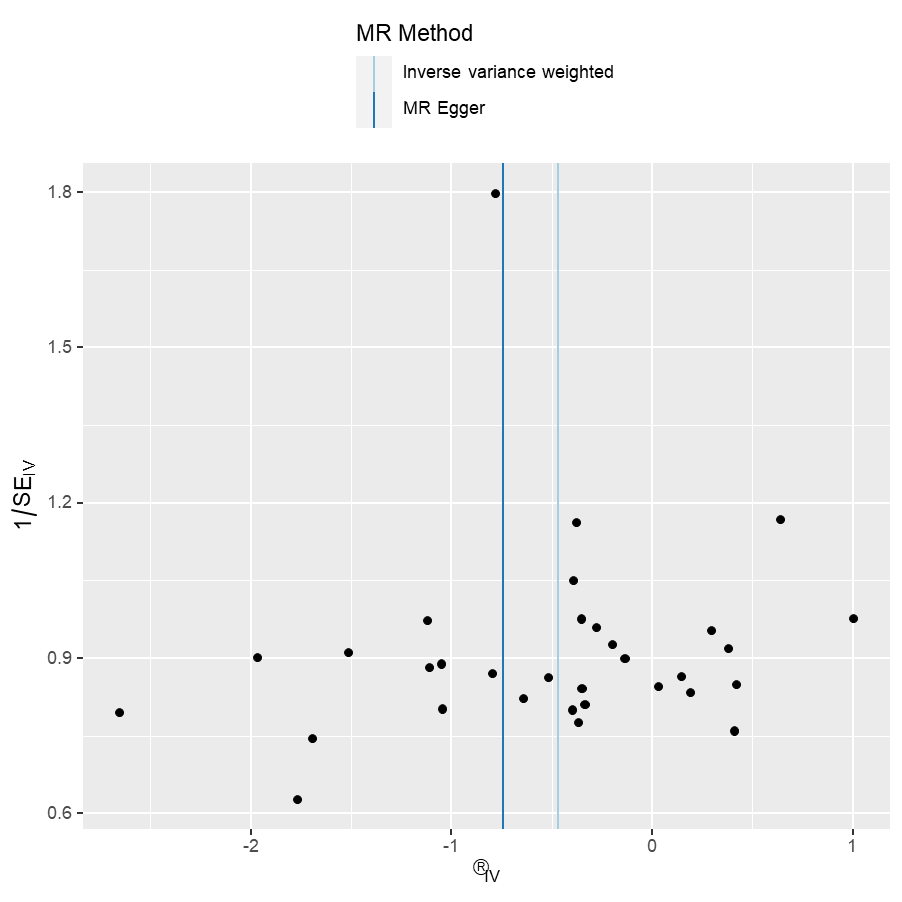
**

**Sup Fig. 21 Scatter plot,** **leave-one-out plot and funnel plot for the causal association between insomnia and *family Oxalobacteraceae*.**

**
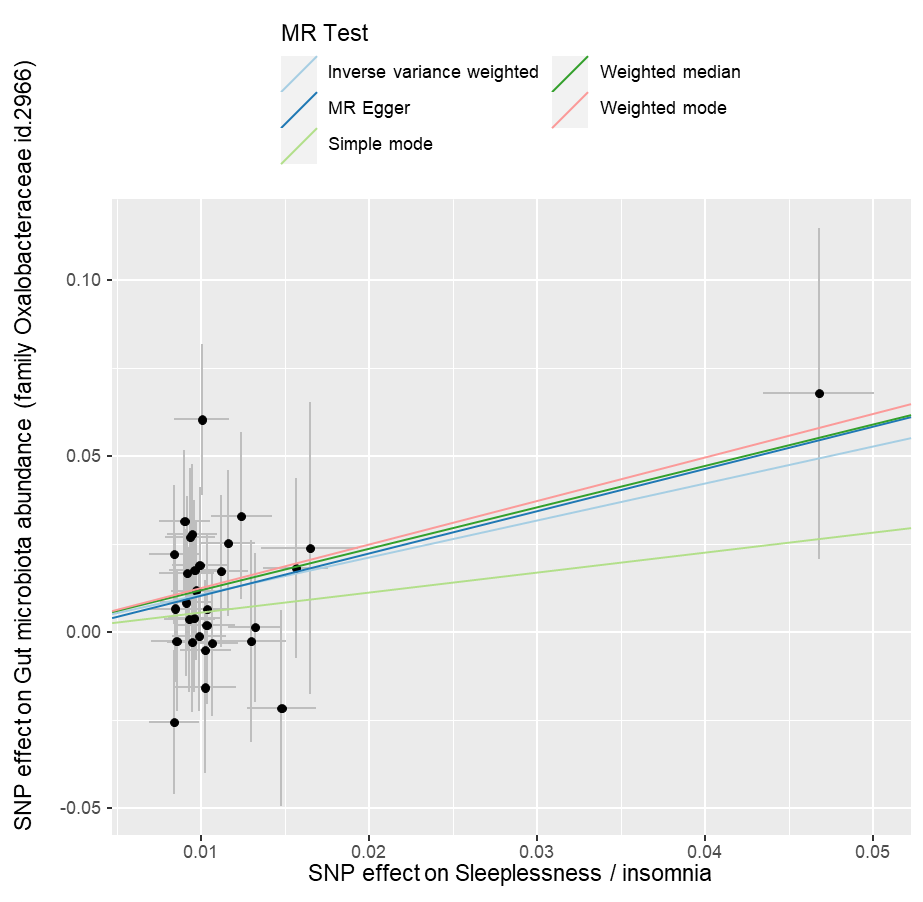

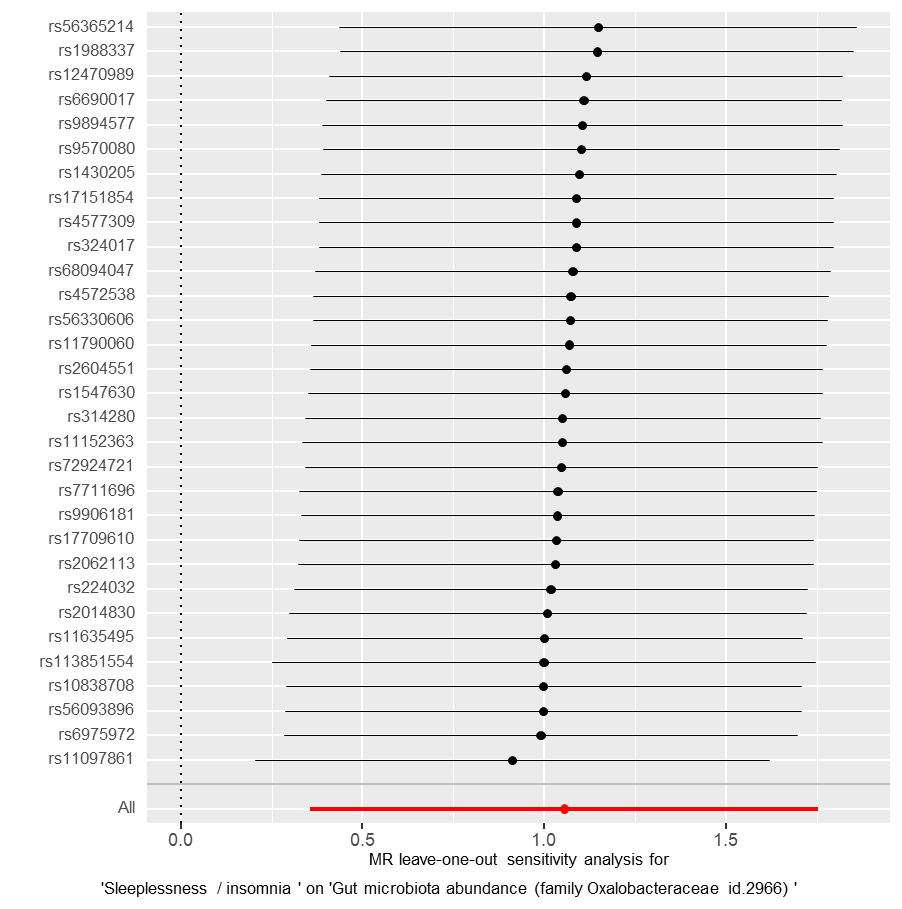

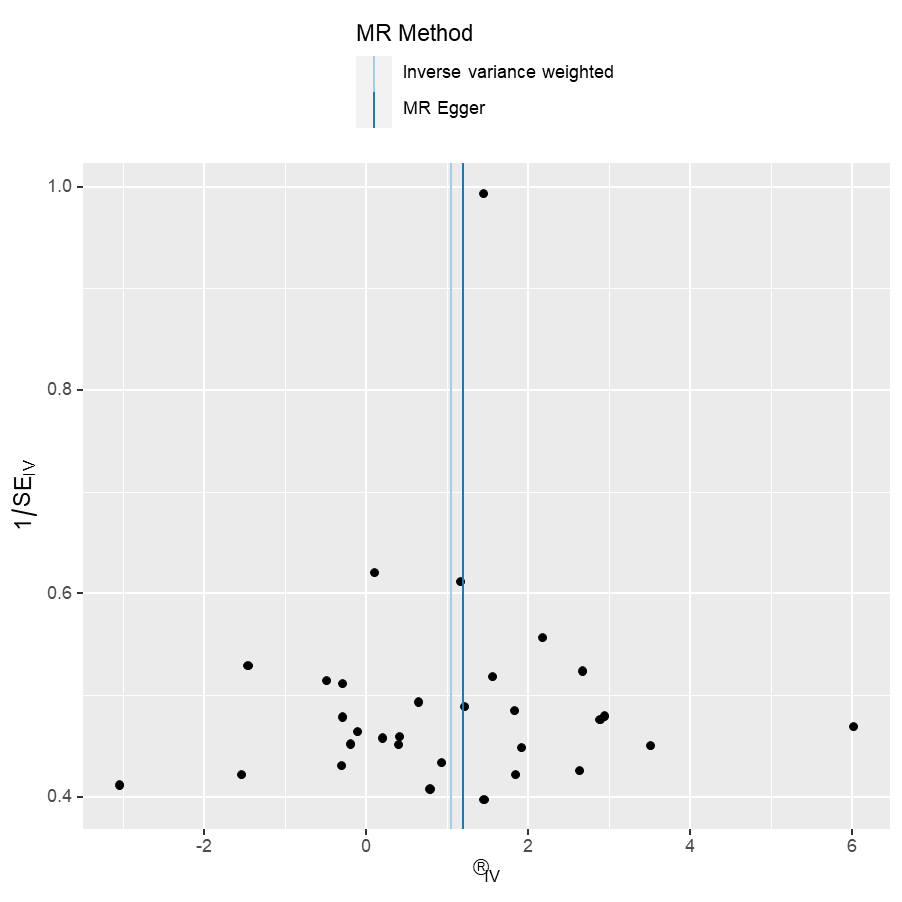
**

**Sup Fig. 22 Scatter plot,** **leave-one-out plot and funnel plot for the causal association between insomnia and *family Rhodospirillaceae*.**

**
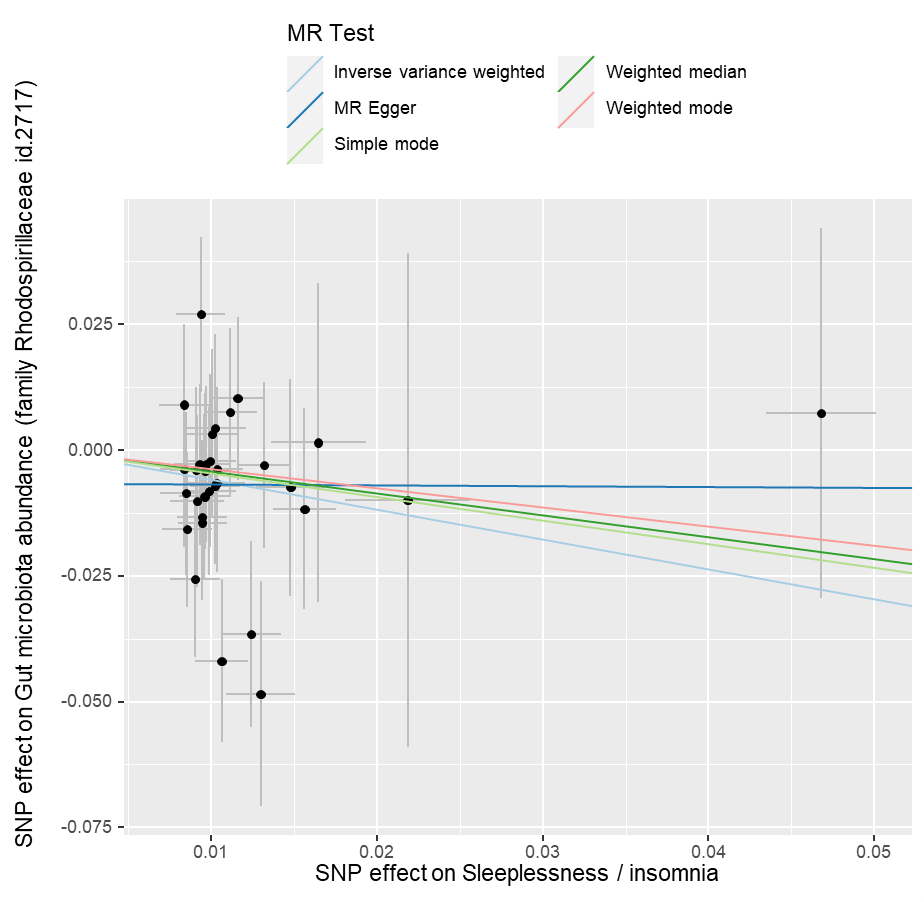

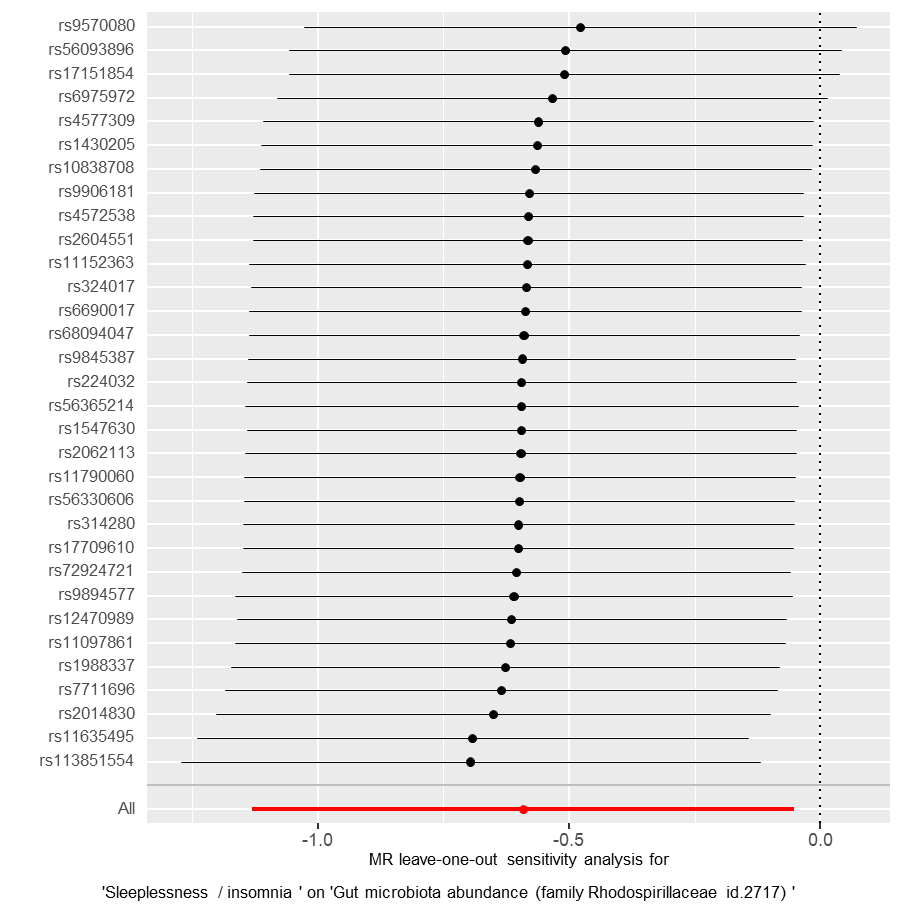

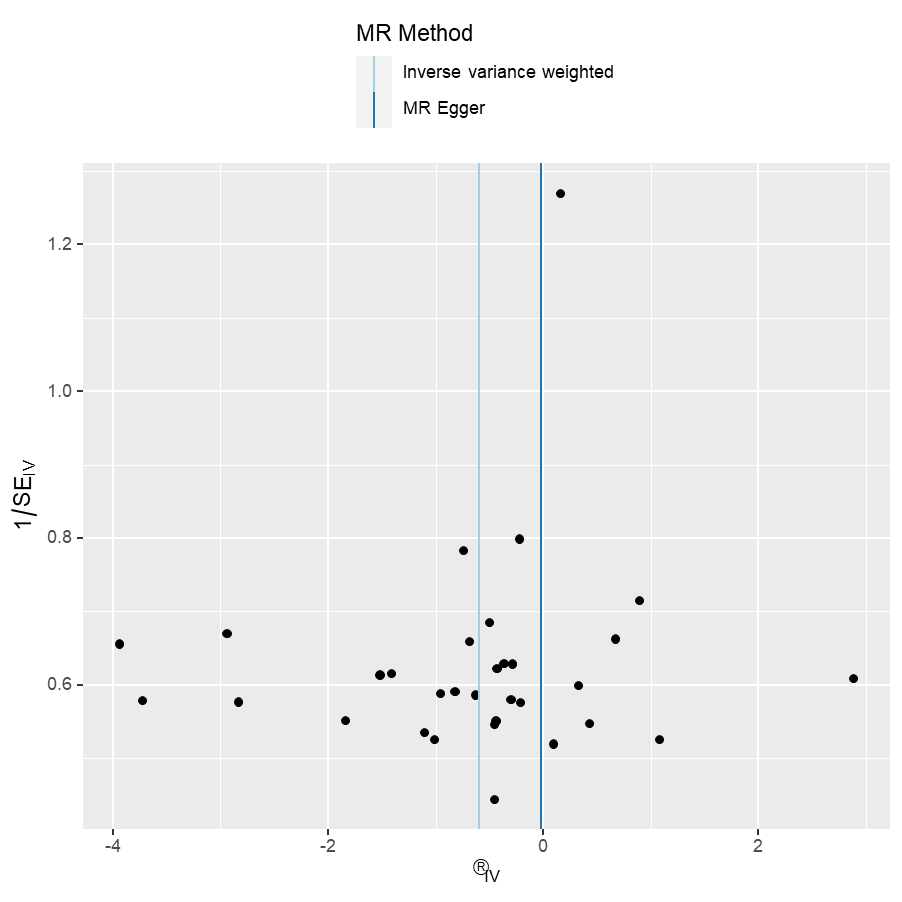
**

**Sup Fig. 23 Scatter plot,** **leave-one-out plot and funnel plot for the causal association between insomnia and *genus Anaerostipes*.**

**
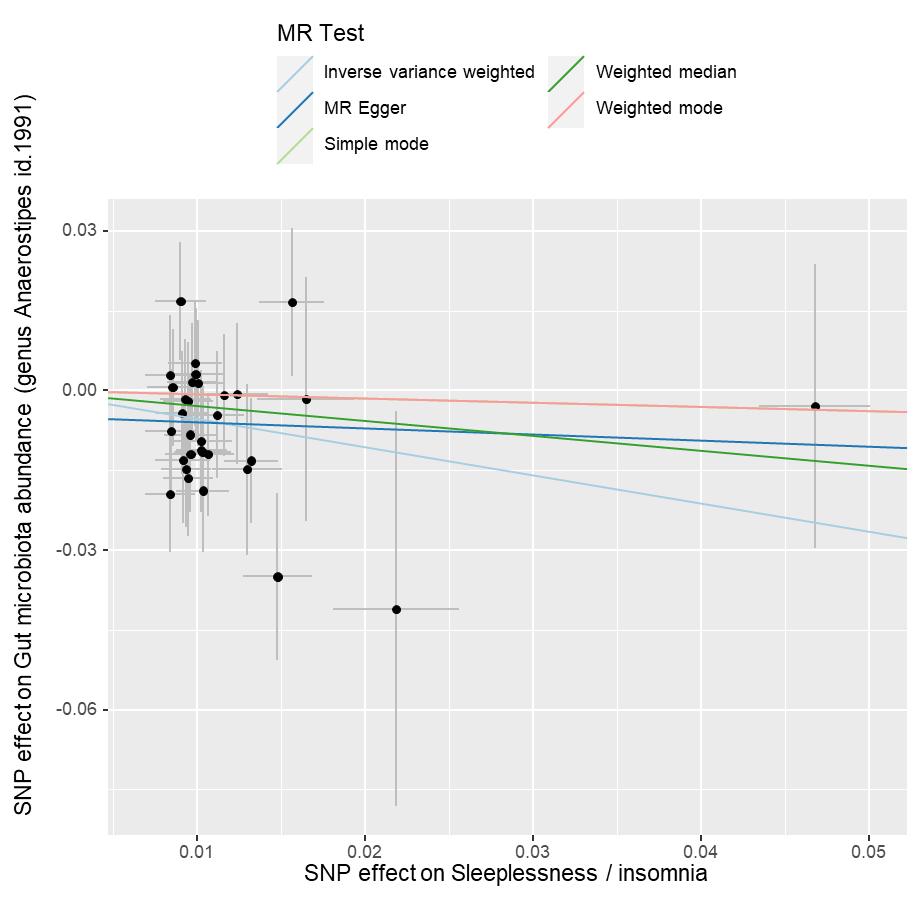

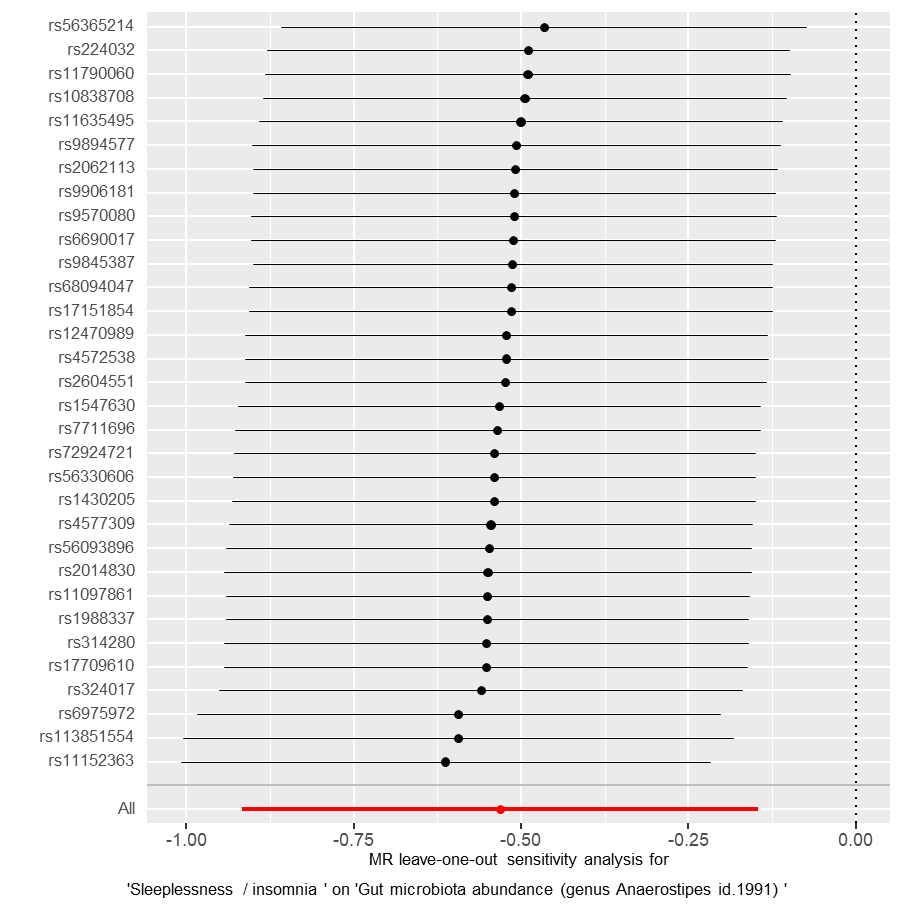

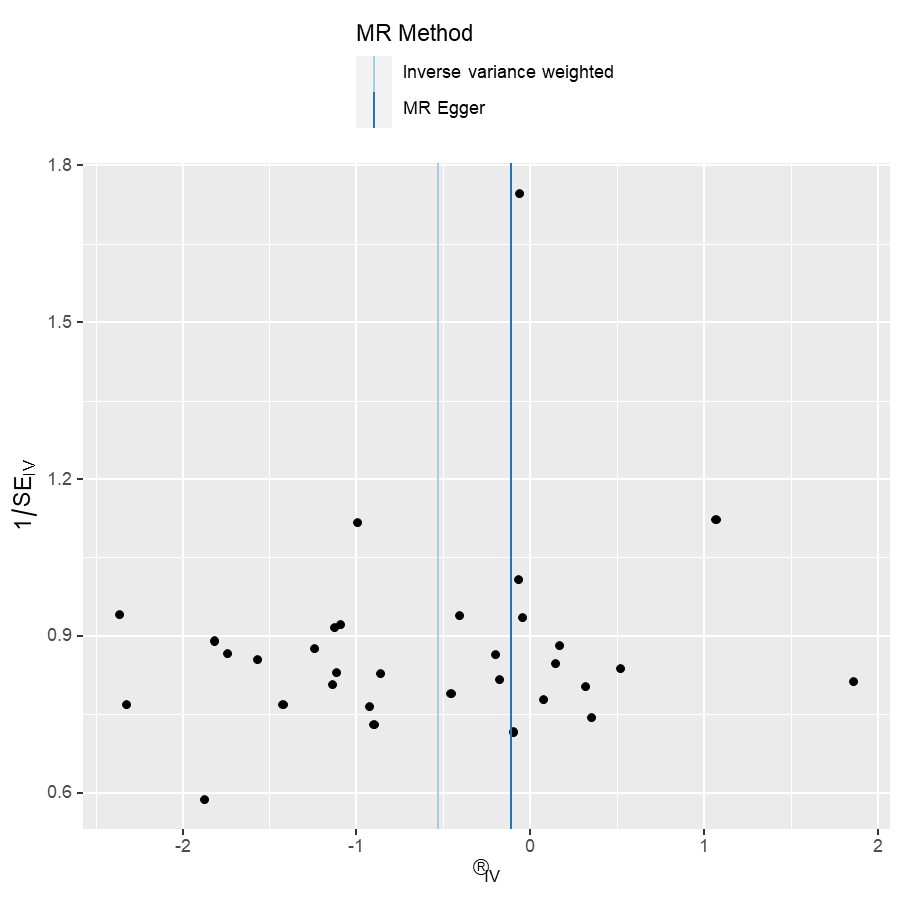
**

**Sup Fig. 24 Scatter plot,** **leave-one-out plot and funnel plot for the causal association between insomnia and *genus Erysipelotrichaceae UCG003*.**

**
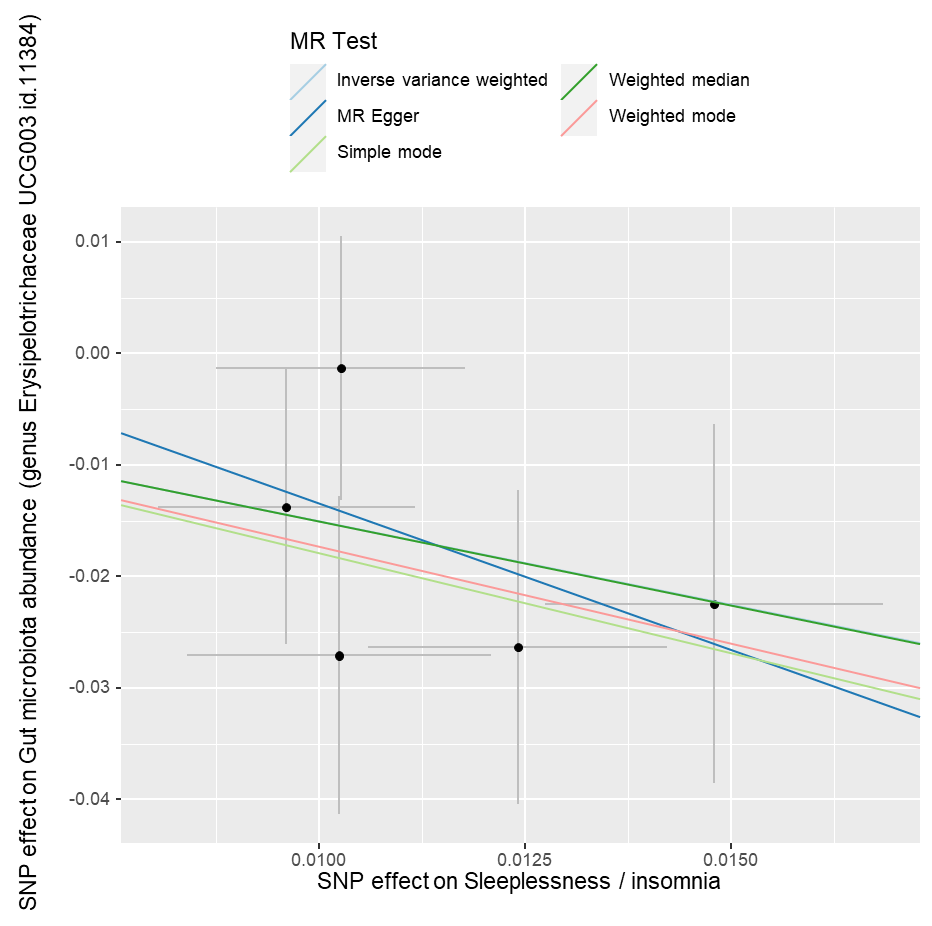

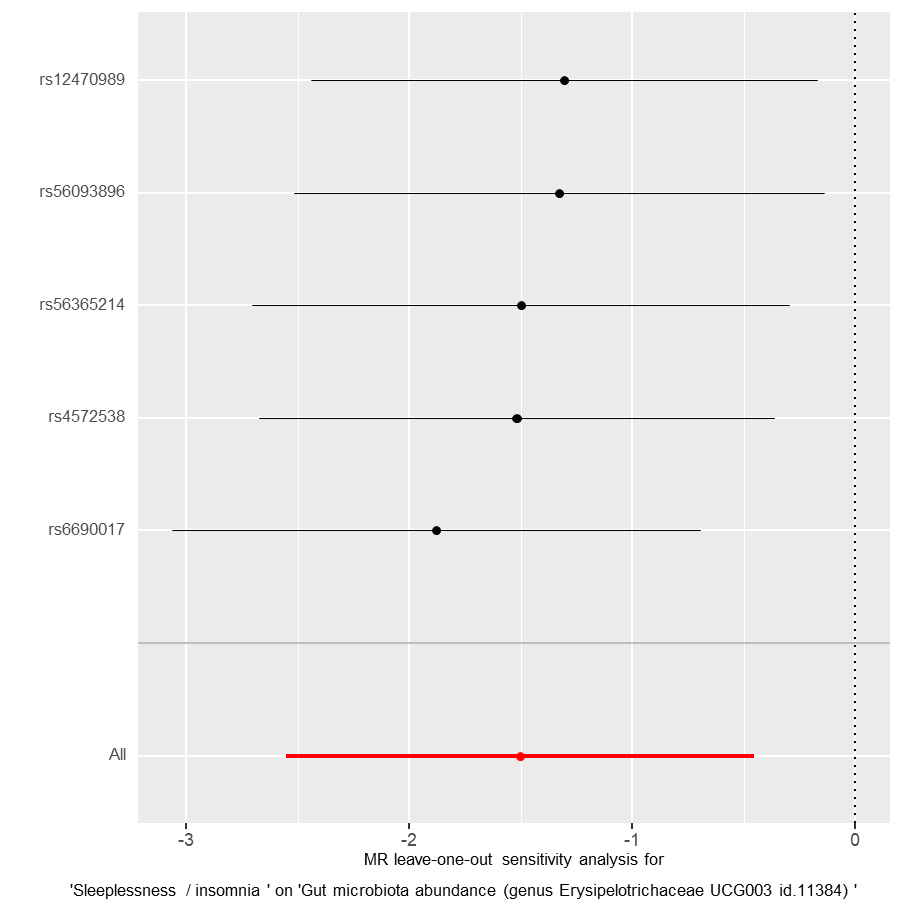

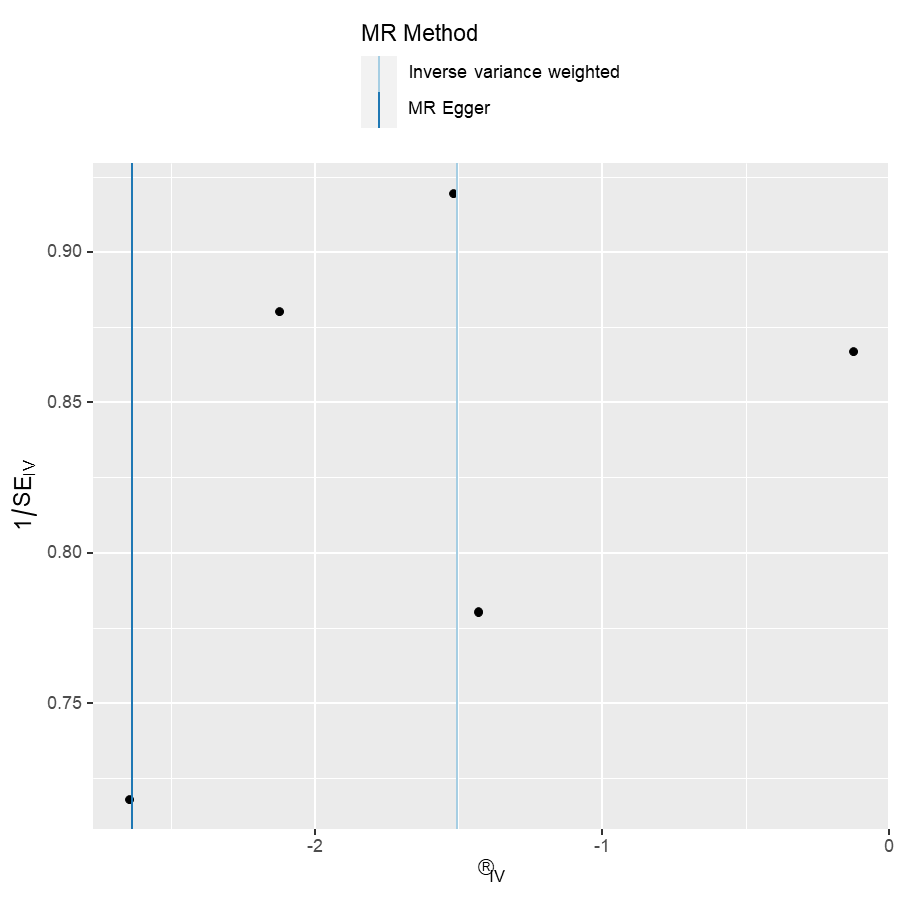
**

**Sup Fig. 25 Scatter plot,** **leave-one-out plot and funnel plot for the causal association between insomnia and *genus Oxalobacter*.**

**
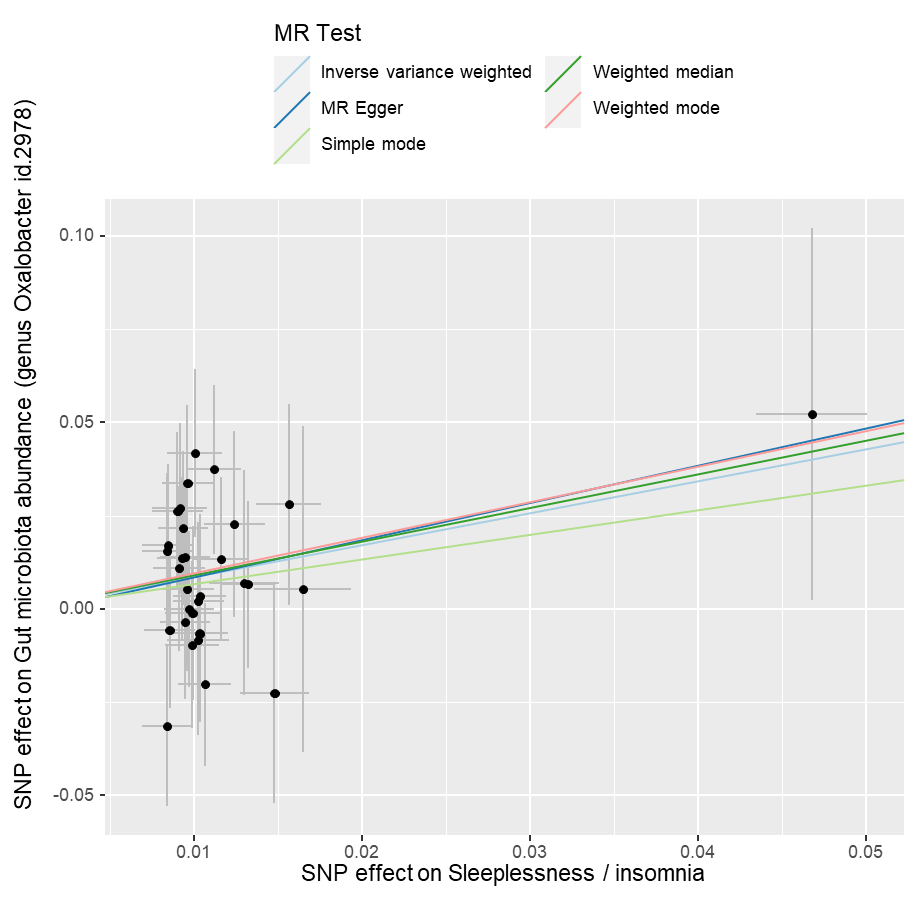

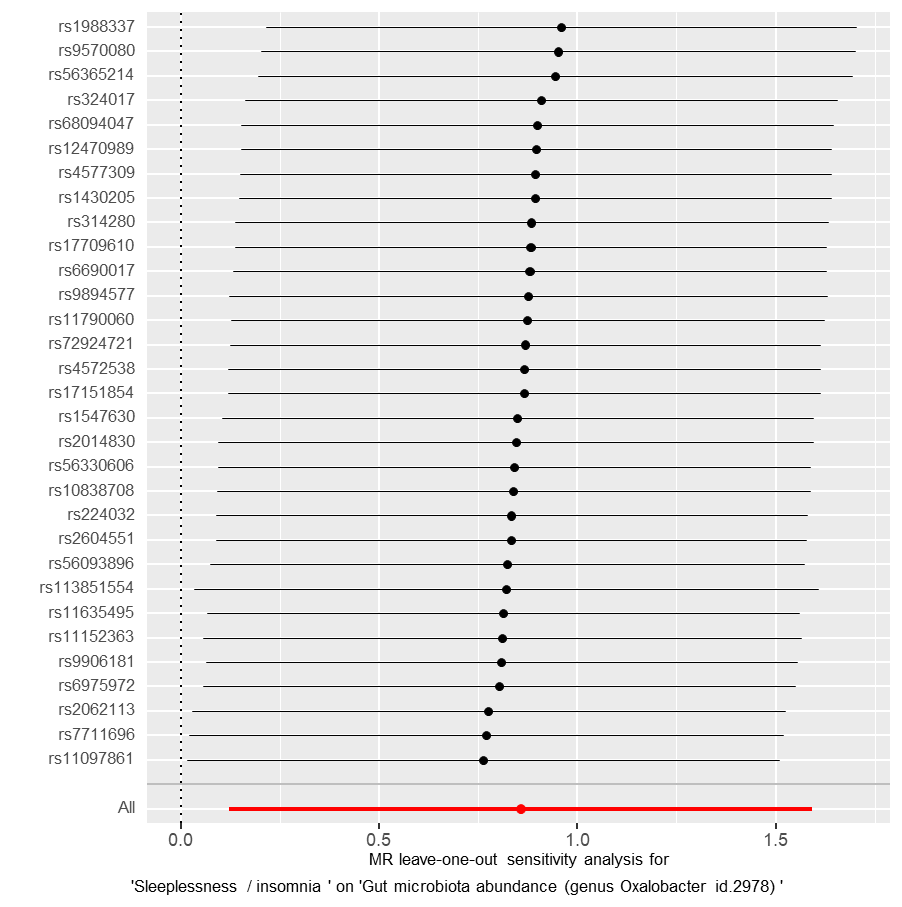

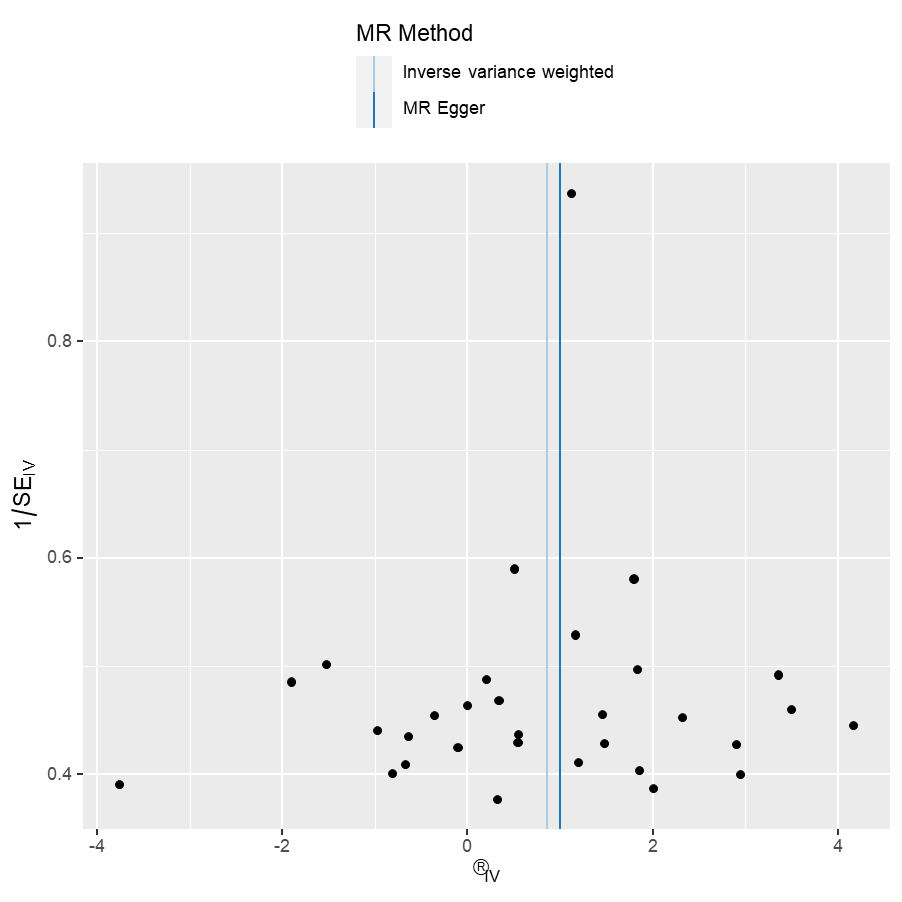
**

**Sup Fig. 26 Scatter plot,** **leave-one-out plot and funnel plot for the causal association between insomnia and *genus Ruminococcaceae*.**

**
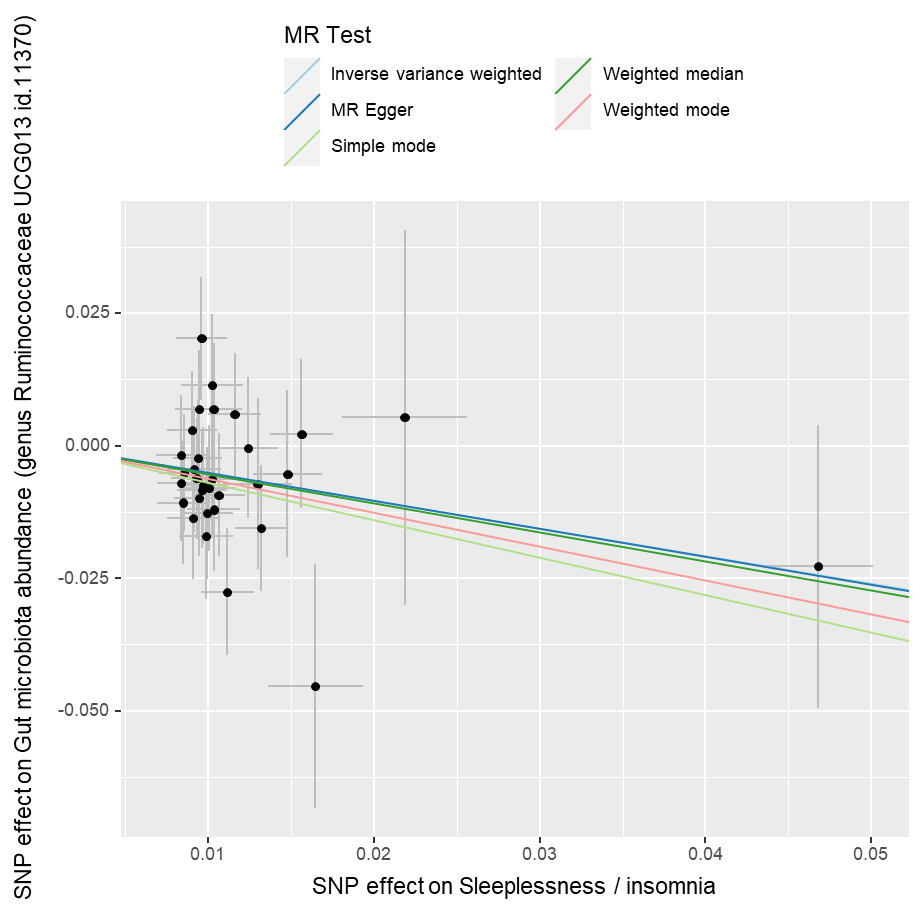

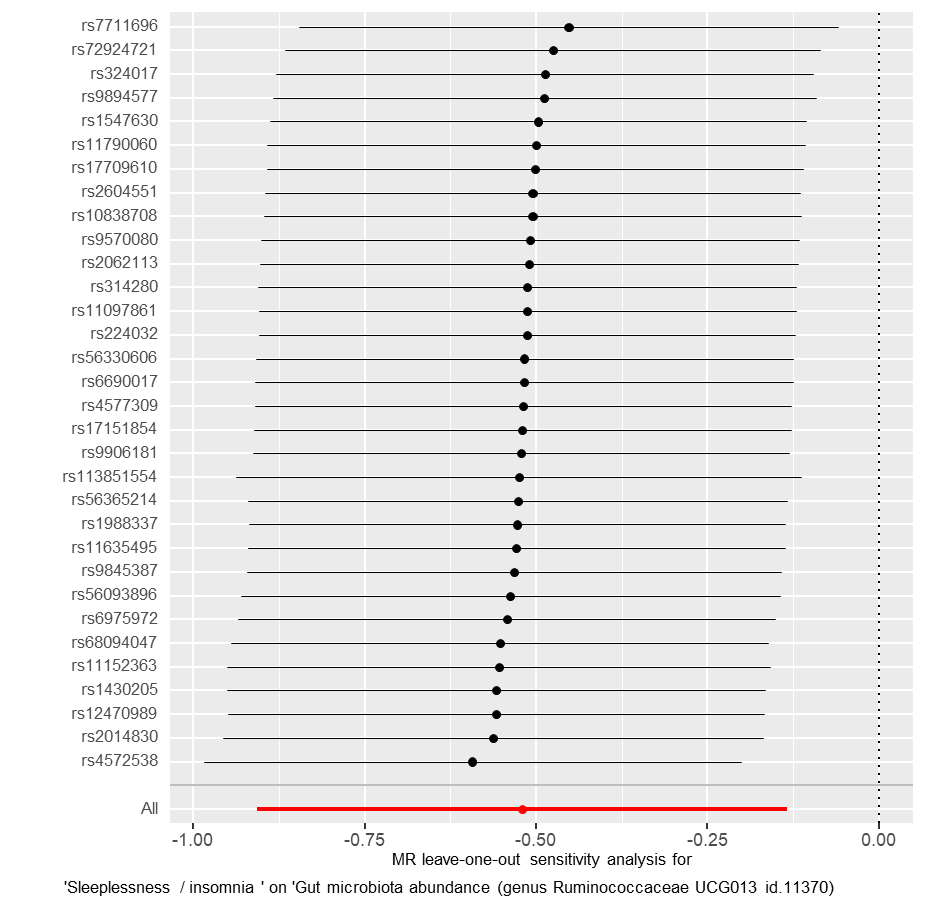

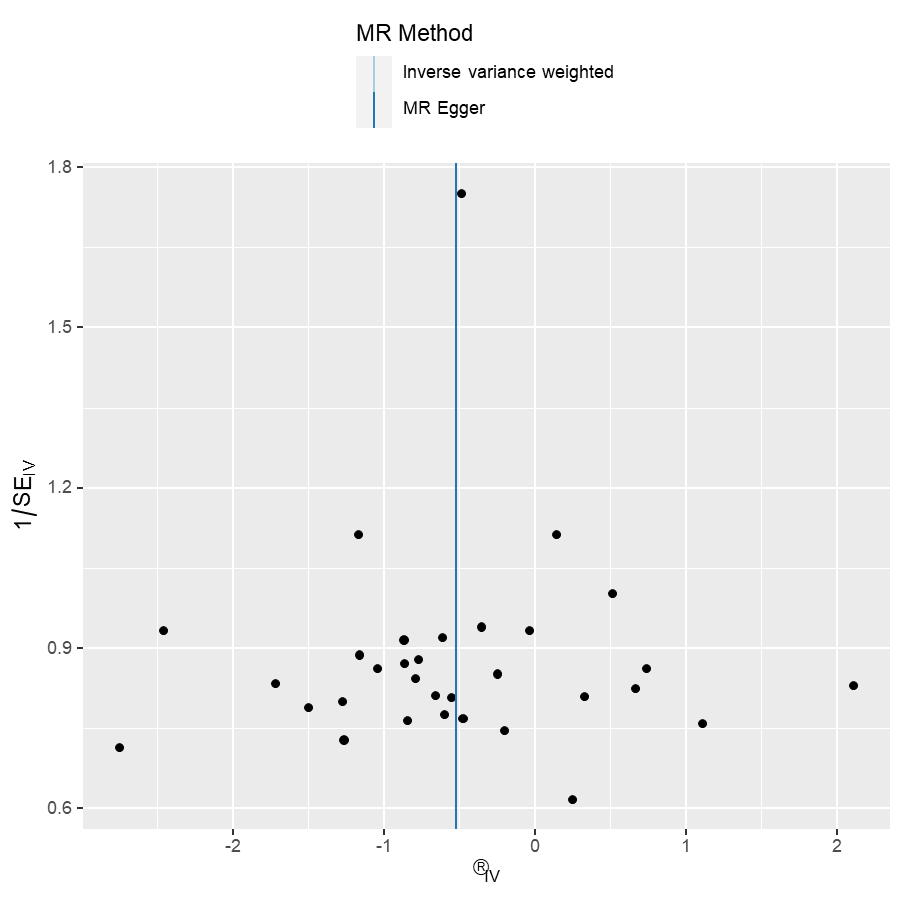
**

**Sup Fig. 27 Scatter plot,** **leave-one-out plot and funnel plot for the causal association between insomnia and *order Erysipelotrichales*.**


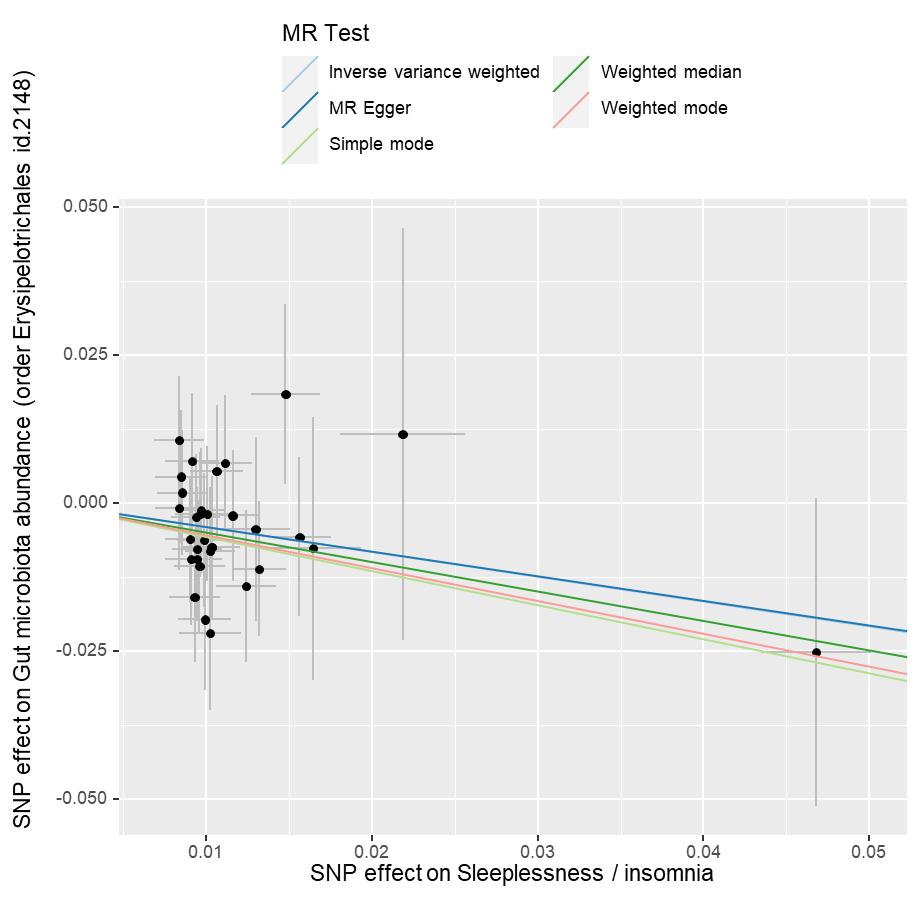

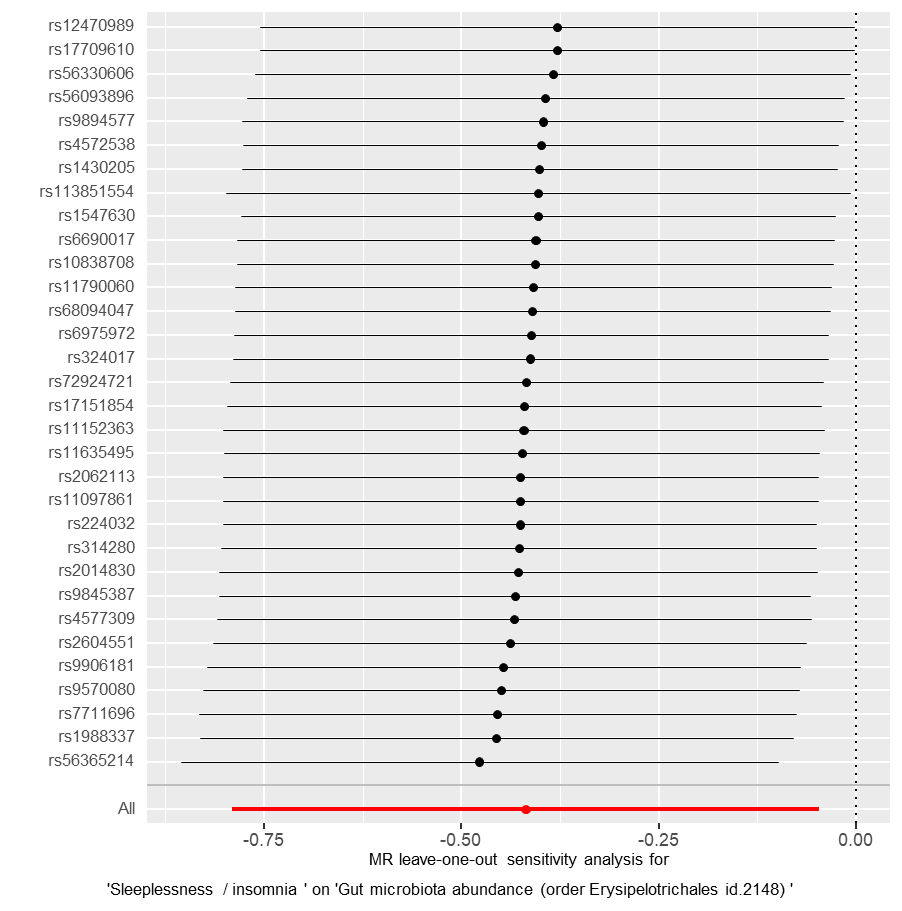

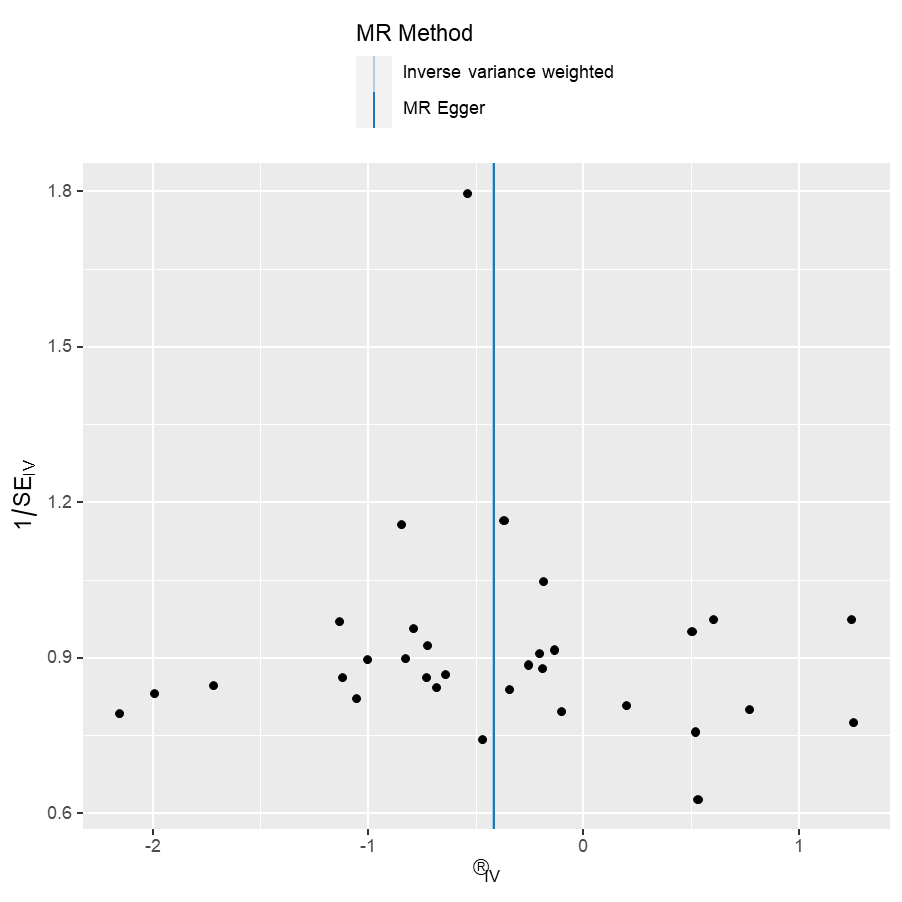


**Sup Fig. 28 Scatter plot,** **leave-one-out plot and funnel plot for the causal association between insomnia and *order Rhodospirillales*.**

**
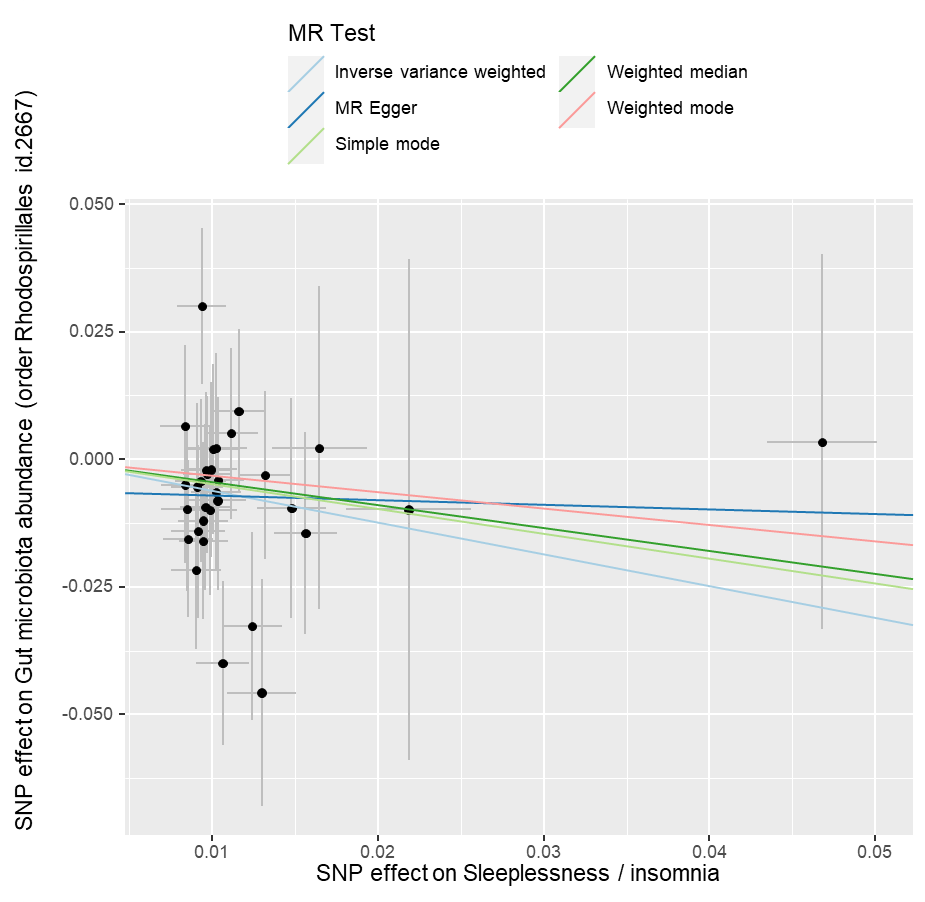

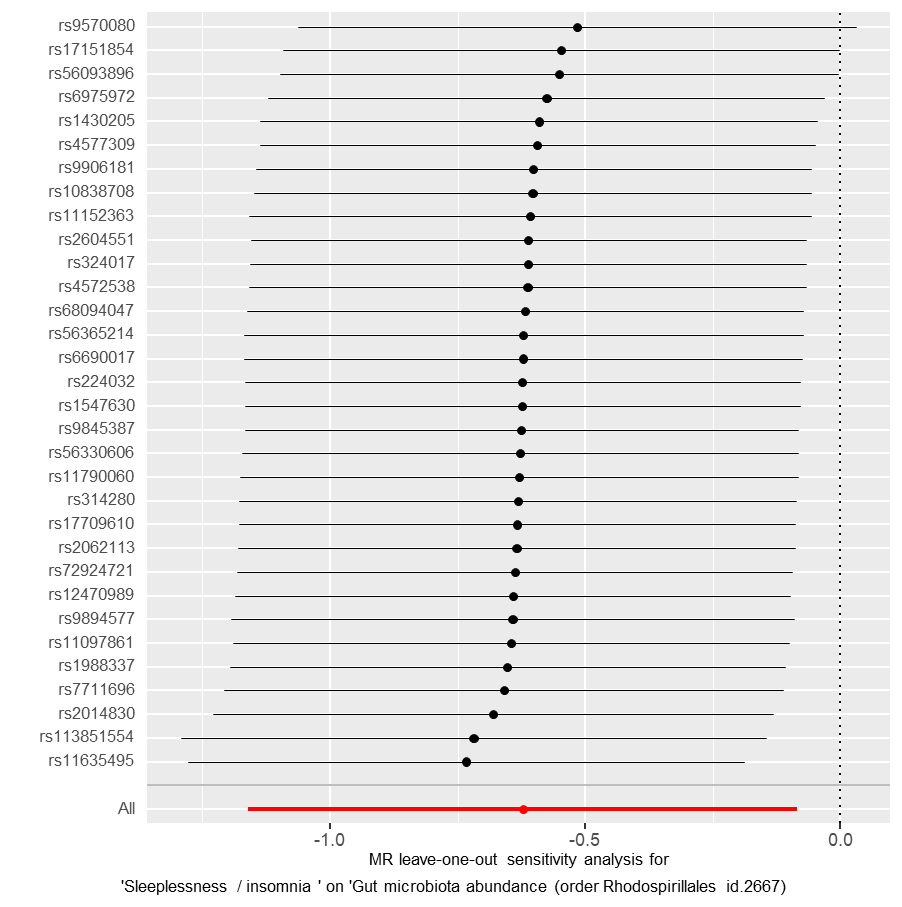

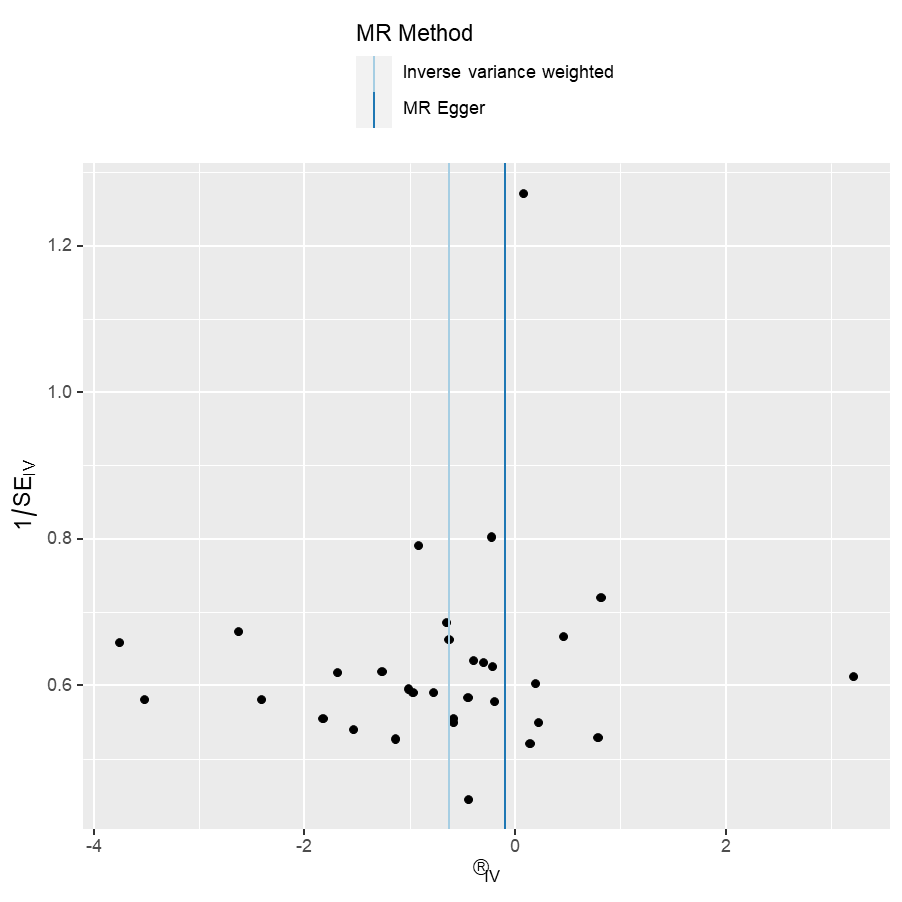
**

**Sup Fig. 29 Scatter plot,** **leave-one-out plot and funnel plot for the causal association between chronotype and *class Bacilli*.**


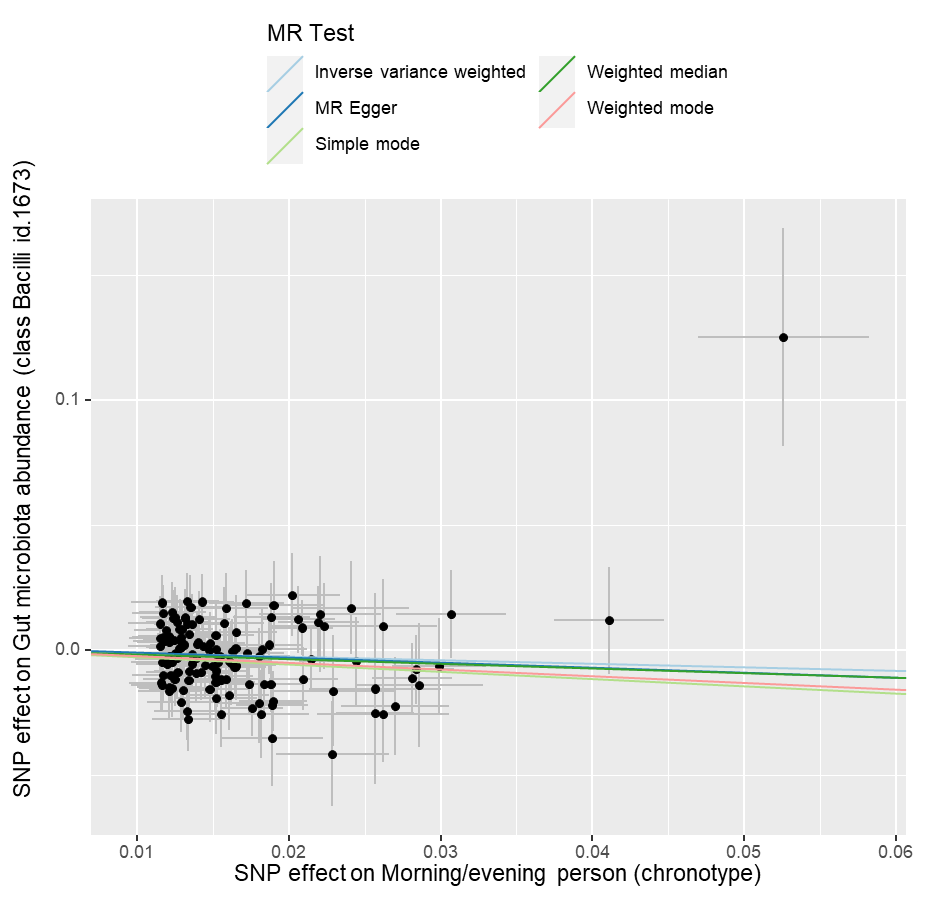

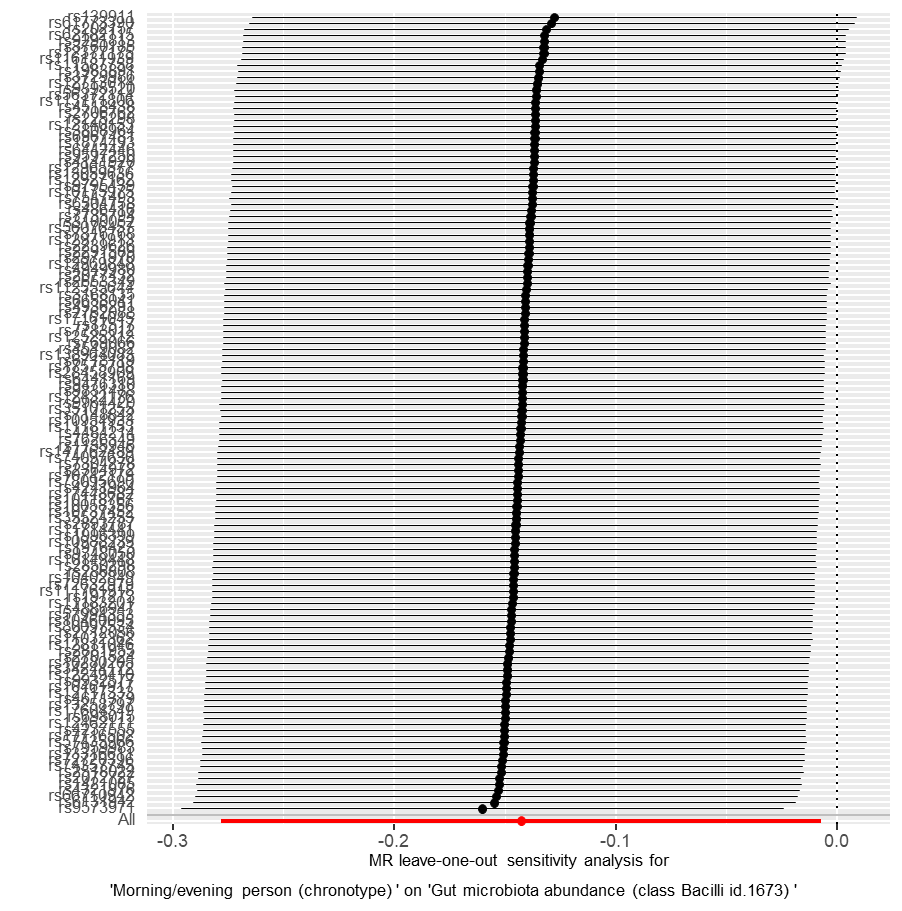

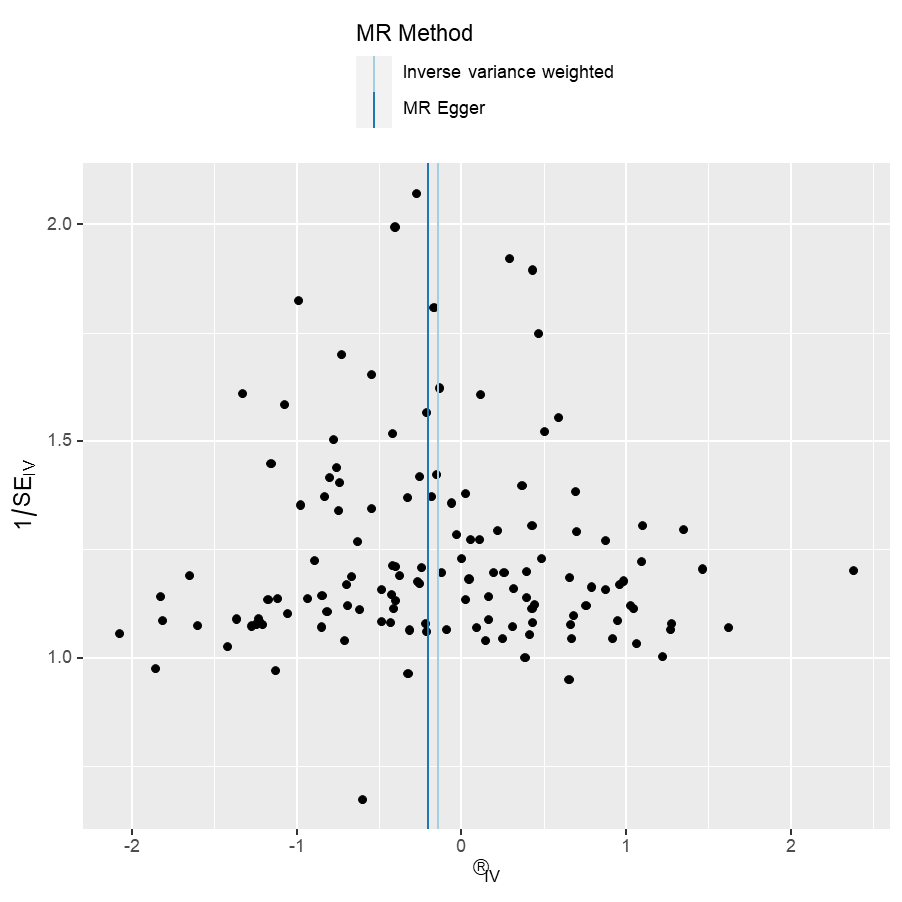


**Sup Fig. 30 Scatter plot,** **leave-one-out plot and funnel plot for the causal association between chronotype and *family Peptostreptococcaceae*.**

**
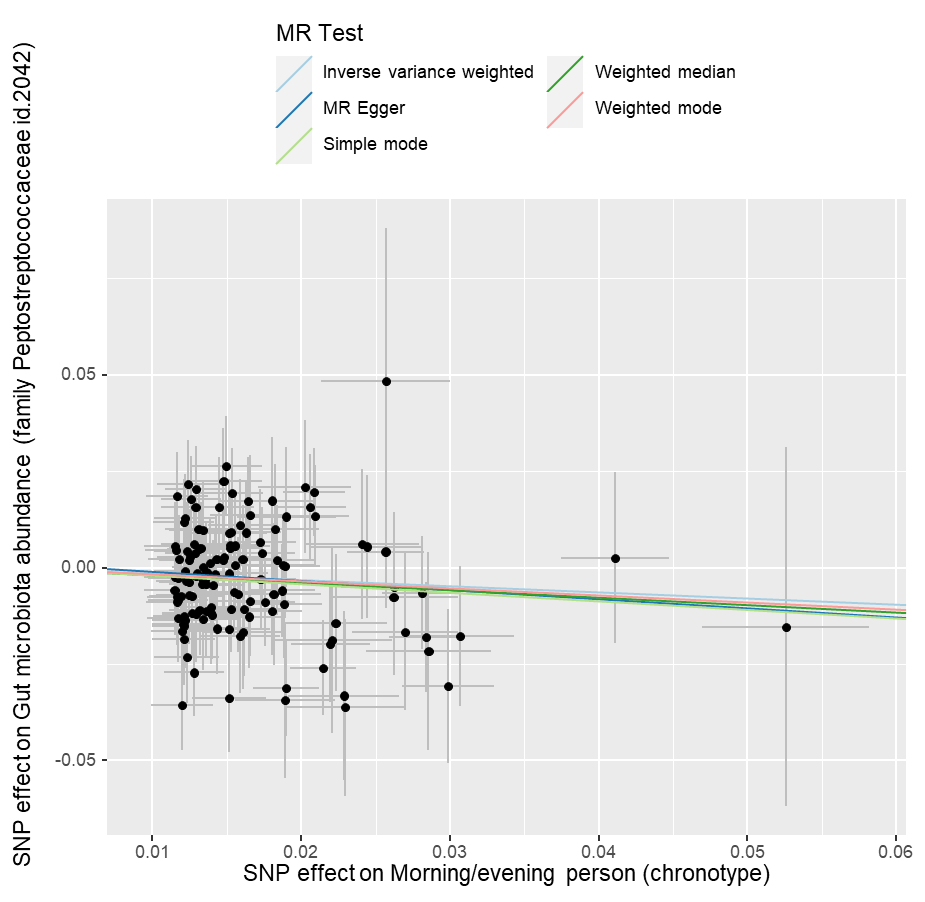

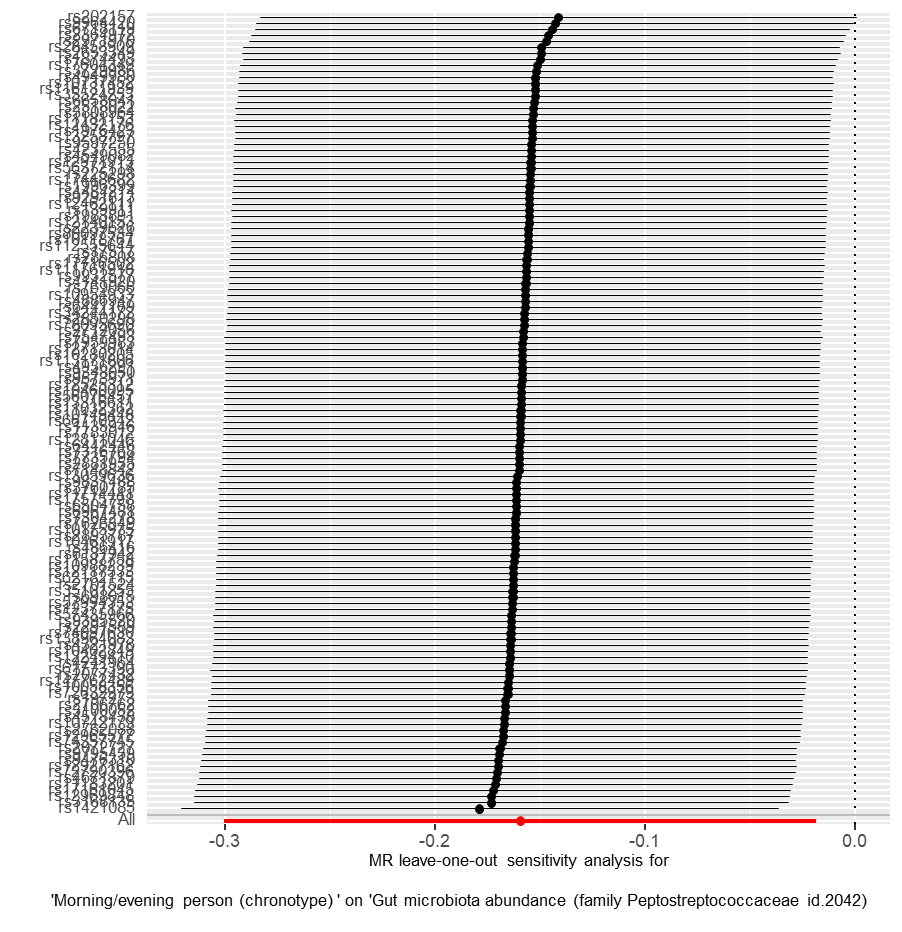

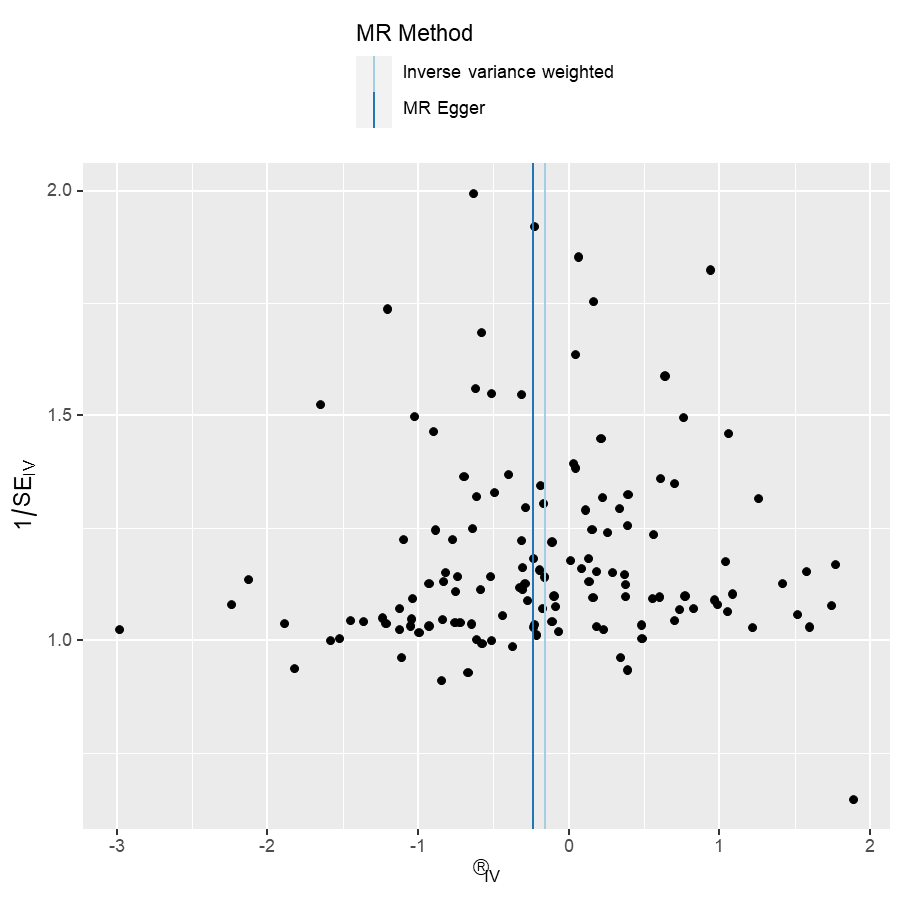
**

**Sup Fig. 31 Scatter plot,** **leave-one-out plot and funnel plot for the causal association between chronotype and *family Porphyromonadaceae*.**

**
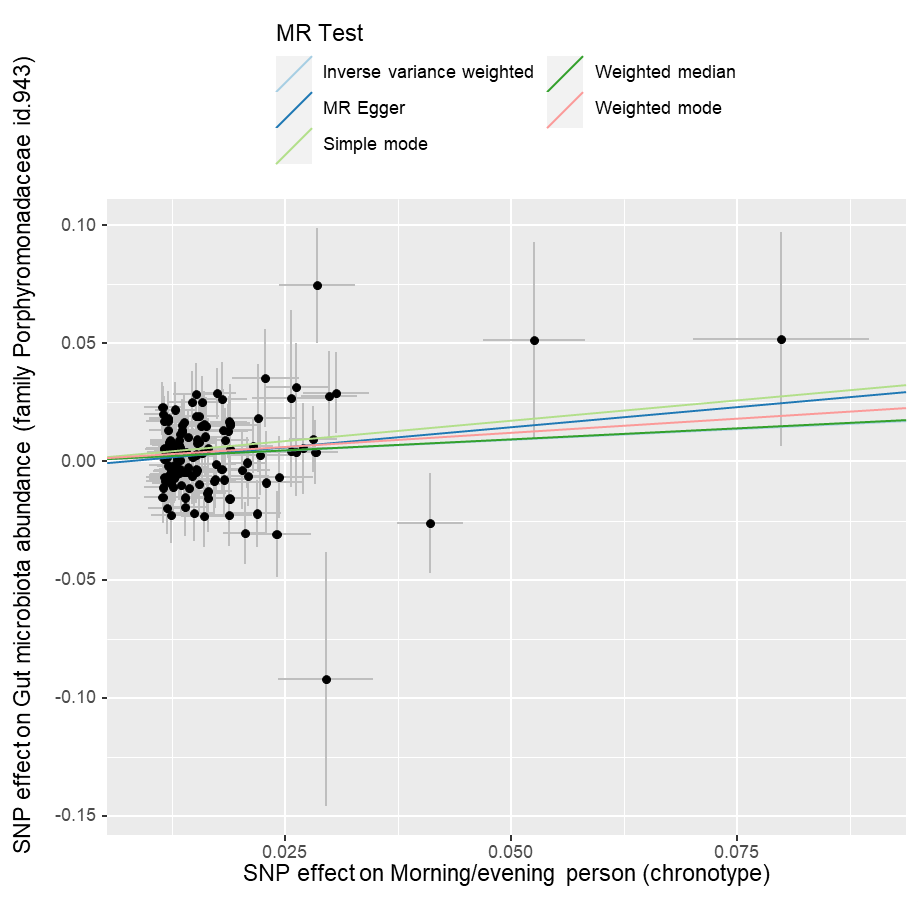

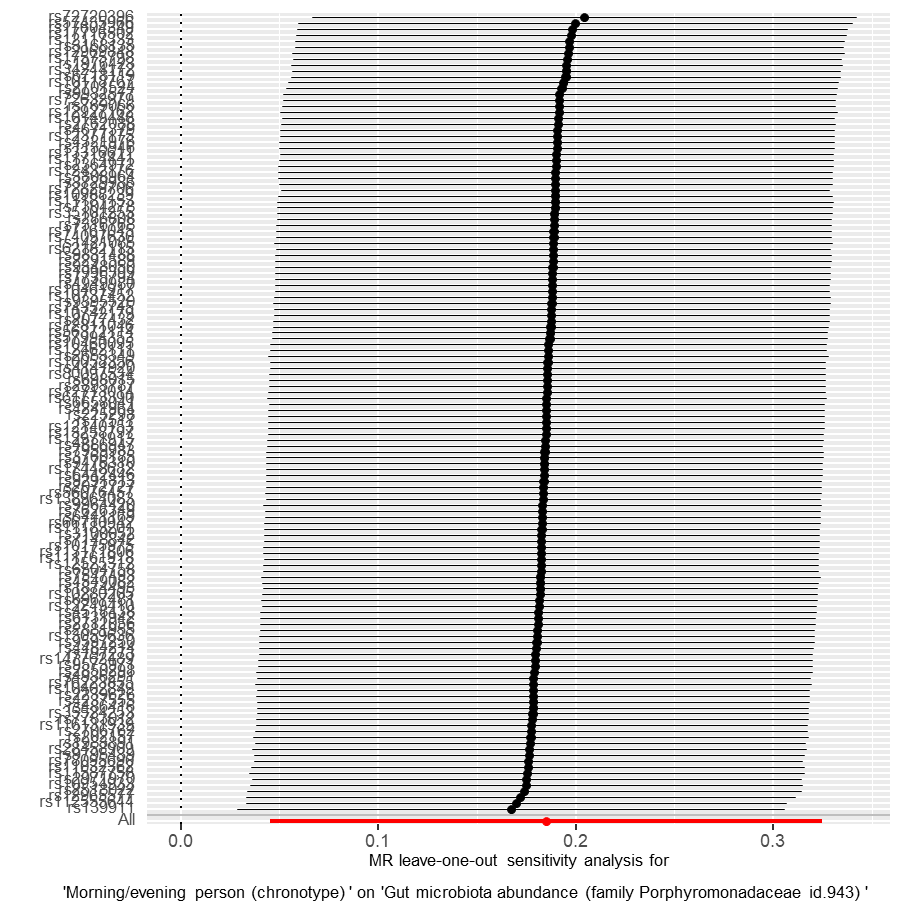

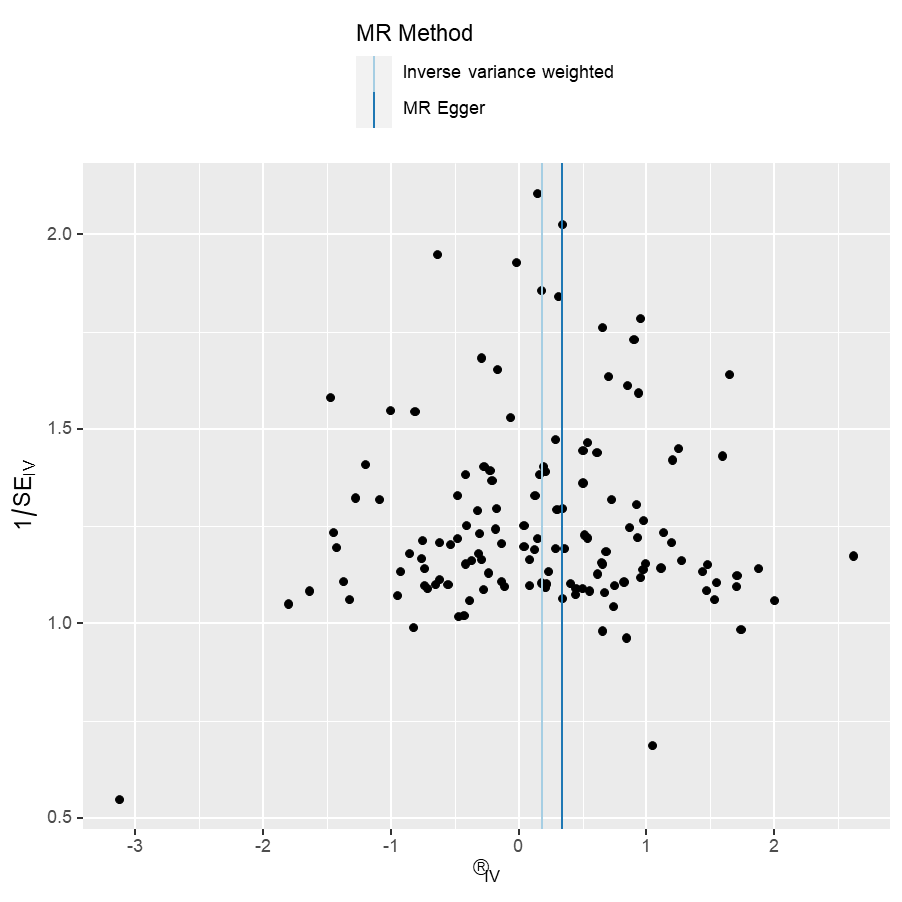
**

**Sup Fig. 32 Scatter plot,** **leave-one-out plot and funnel plot for the causal association between chronotype and *family Streptococcaceae*.**

**
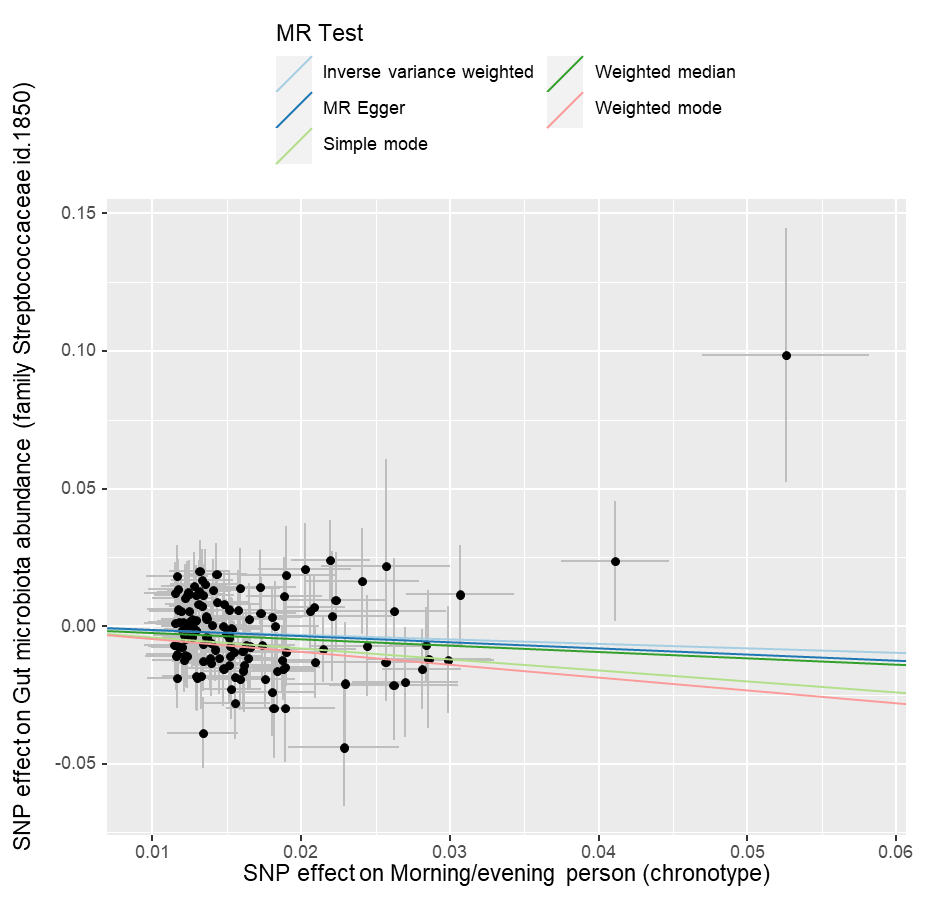

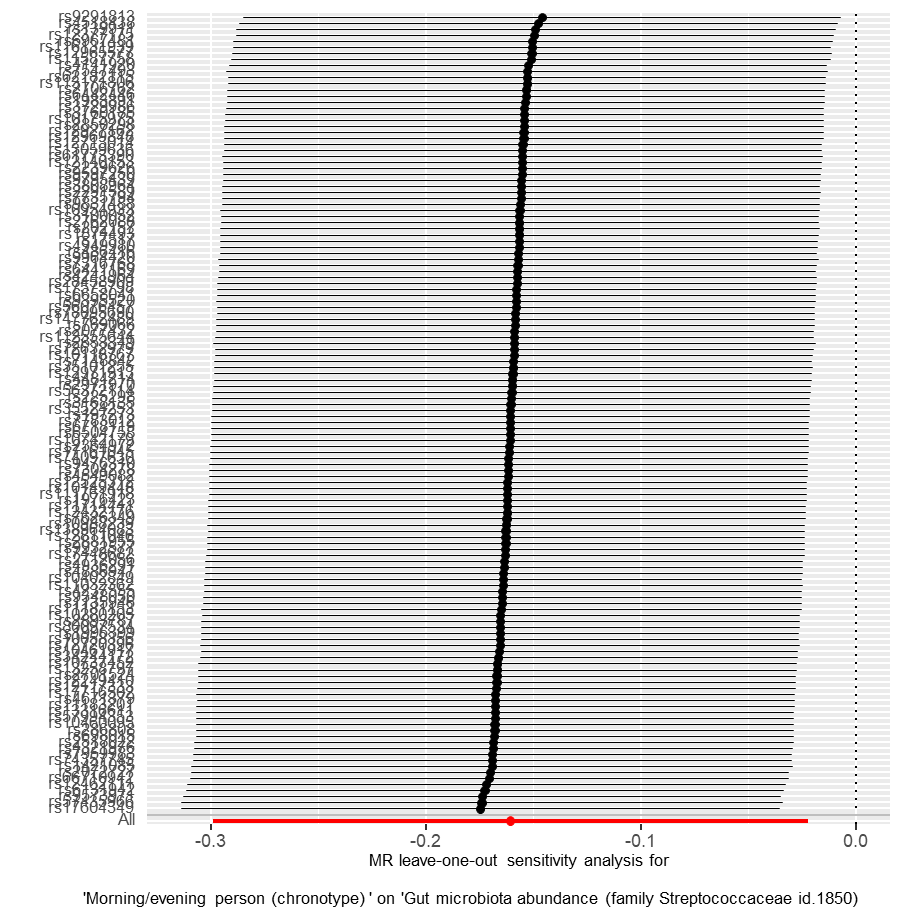

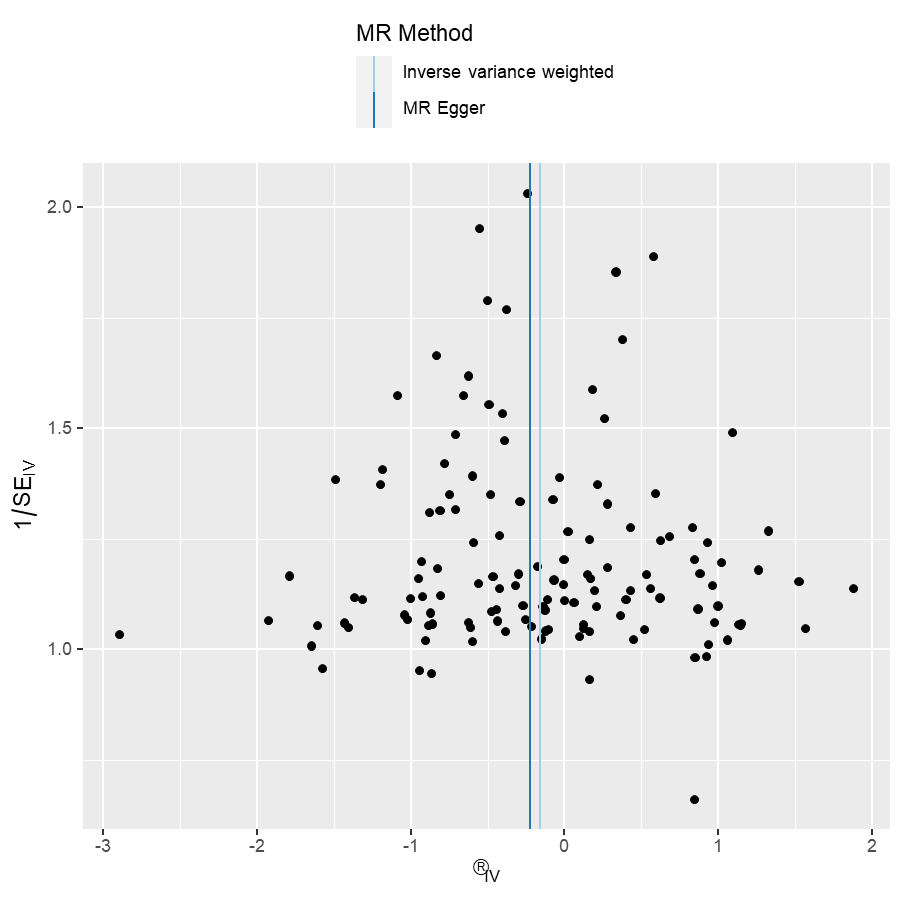
**

**Sup Fig. 33 Scatter plot,** **leave-one-out plot and funnel plot for the causal association between chronotype and *genus Butyricicoccus*.**

**
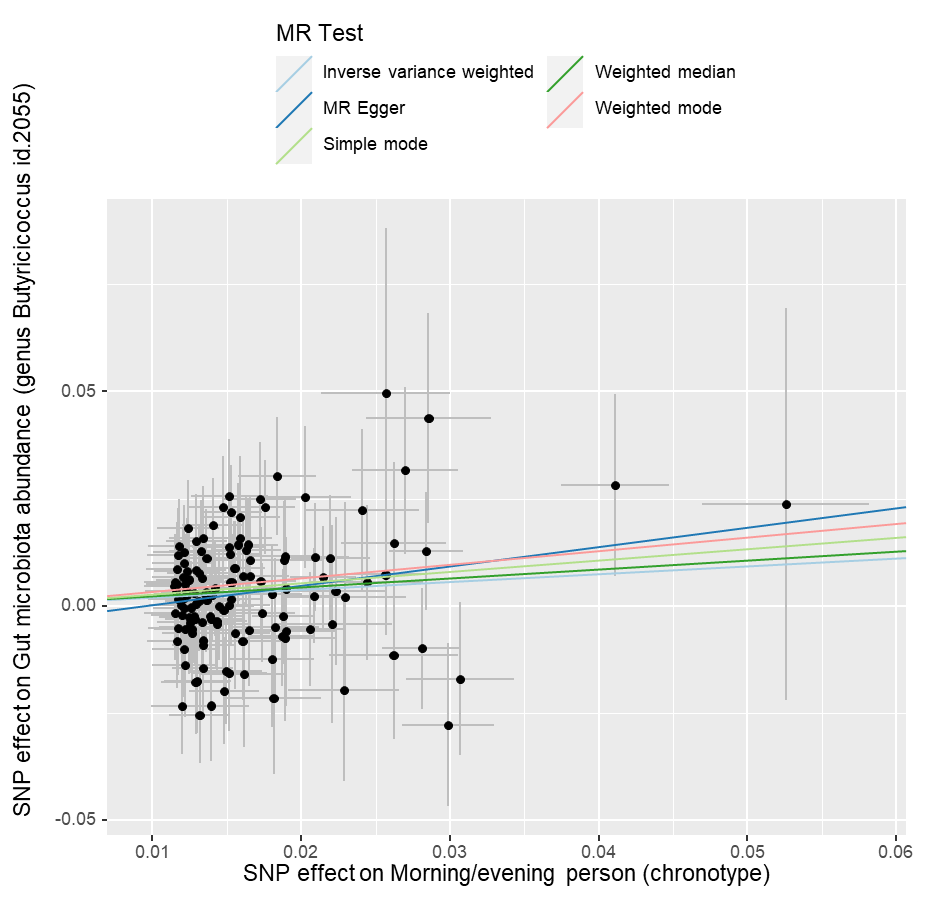

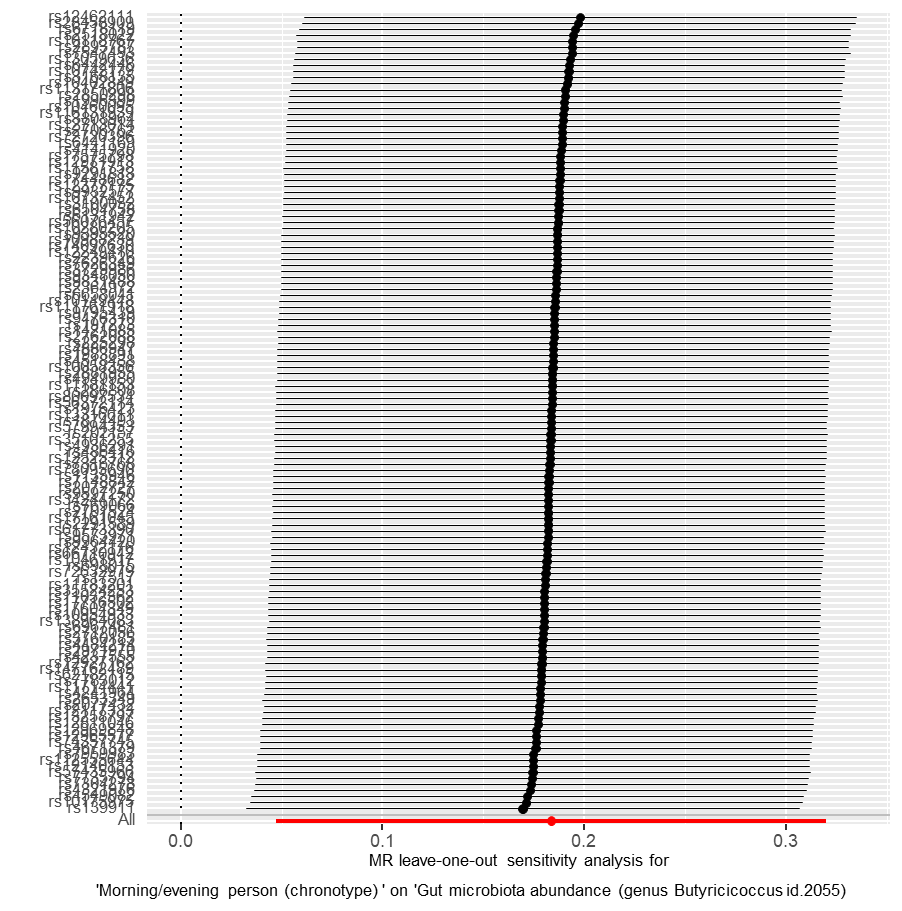

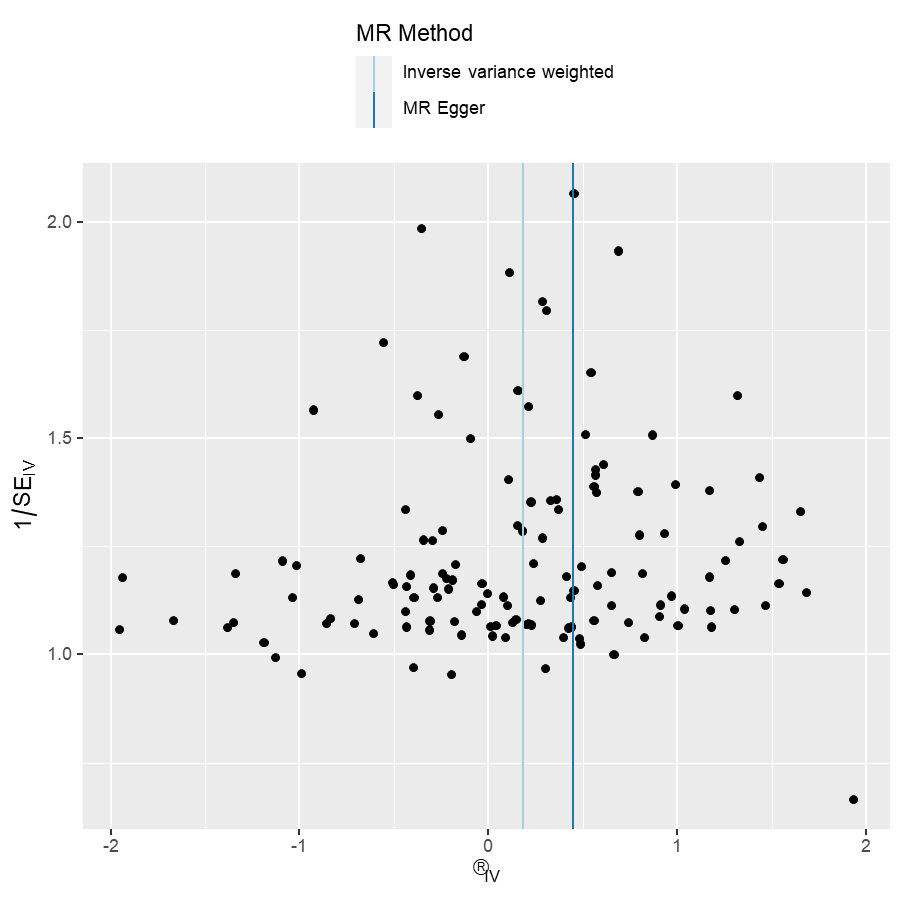
**

**Sup Fig. 34 Scatter plot,** **leave-one-out plot and funnel plot for the causal association between chronotype and *genus Ruminococcaceae*.**

**
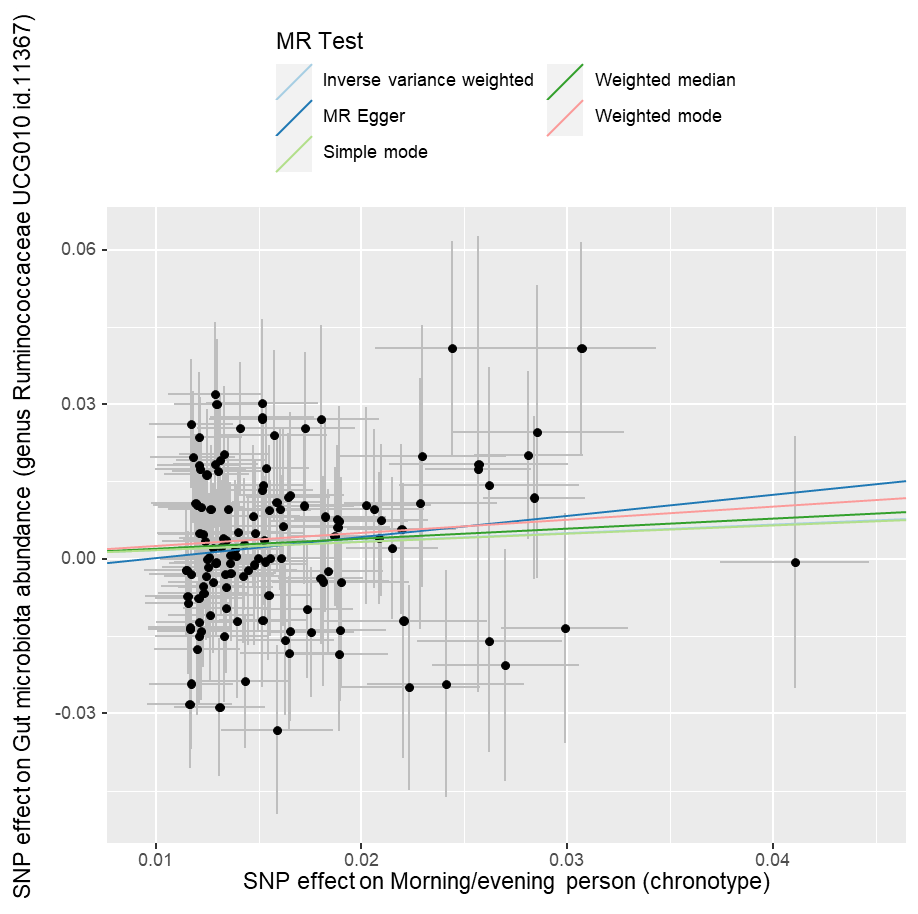
**

**Sup Fig. 35 Scatter plot,** **leave-one-out plot and funnel plot for the causal association between chronotype and *genus Streptococcus*.**

**Sup Fig. 36 Scatter plot,** **leave-one-out plot and funnel plot for the causal association between chronotype and *order Lactobacillales*.**

**Sup Fig. 37 Scatter plot,** **leave-one-out plot and funnel plot for the causal association between nap during day and *class Deltaproteobacteria*.**

**Sup Fig. 38 Scatter plot,** **leave-one-out plot and funnel plot for the causal association between nap during day and *family Christensenellaceae*.**

**Sup Fig. 39 Scatter plot,** **leave-one-out plot and funnel plot for the causal association between nap during day and *family Desulfovibrionaceae*.**

**Sup Fig. 40 Scatter plot,** **leave-one-out plot and funnel plot for the causal association between nap during day and *genus Butyricicoccus*.**

**Sup Fig. 41 Scatter plot,** **leave-one-out plot and funnel plot for the causal association between nap during day and *genus Desulfovibrio*.**

**Sup Fig. 42 Scatter plot,** **leave-one-out plot and funnel plot for the causal association between nap during day and *genus Enterorhabdus*.**

**Sup Fig. 43 Scatter plot,** **leave-one-out plot and funnel plot for the causal association between nap during day and *genus Fusicatenibacter*.**

**Sup Fig. 44 Scatter plot,** **leave-one-out plot and funnel plot for the causal association between nap during day and *genus Romboutsia*.**

**Sup Fig. 45 Scatter plot,** **leave-one-out plot and funnel plot for the causal association between nap during day and *order Desulfovibrionales*.**

**Sup Fig. 46 Scatter plot,** **leave-one-out plot and funnel plot for the causal association between sleep duration and *class Bacteroidia*.**

**Sup Fig. 47 Scatter plot,** **leave-one-out plot and funnel plot for the causal association between sleep duration and *class Coriobacteriia*.**

**Sup Fig. 48 Scatter plot,** **leave-one-out plot and funnel plot for the causal association between sleep duration and *family Bacteroidaceae*.**

**Sup Fig. 49 Scatter plot,** **leave-one-out plot and funnel plot for the causal association between sleep duration and *family Coriobacteriaceae*.**

**Sup Fig. 50 Scatter plot,** **leave-one-out plot and funnel plot for the causal association between sleep duration and *genus Alloprevotella*.**

**Sup Fig. 51 Scatter plot,** **leave-one-out plot and funnel plot for the causal association between sleep duration and *genus Bacteroides*.**

**Sup Fig. 52 Scatter plot,** **leave-one-out plot and funnel plot for the causal association between sleep duration and *genus Lachnospiraceae UCG008*.**

**Sup Fig. 53 Scatter plot,** **leave-one-out plot and funnel plot for the causal association between sleep duration and *genus Senegalimassilia*.**

**Sup Fig. 54 Scatter plot,** **leave-one-out plot and funnel plot for the causal association between sleep duration and *order Bacteroidales*.**

**Sup Fig. 55 Scatter plot,** **leave-one-out plot and funnel plot for the causal association between sleep duration and *order Coriobacteriales*.**

**Sup Fig. 56 Scatter plot,** **leave-one-out plot and funnel plot for the causal association between sleep duration and *phylum Bacteroidetes*.**

**Sup Fig. 57 Scatter plot,** **leave-one-out plot and funnel plot for the causal association between snoring and *genus Anaerostipes*.**

**Sup Fig. 58 Scatter plot,** **leave-one-out plot and funnel plot for the causal association between snoring and *genus Erysipelotrichaceae UCG003*.**

**Sup Fig. 59 Scatter plot,** **leave-one-out plot and funnel plot for the causal association between snoring and *genus Lactobacillus*.**

**Sup Fig. 60 Scatter plot,** **leave-one-out plot and funnel plot for the causal association between snoring and *genus Peptococcus*.**
