# Supplementary material for: Association between sleep-related phenotypes and gut microbiota: a two-sample bidirectional Mendelian randomization study
Source: Front Microbiol. 2024 Feb 2;15:1341643. doi: 10.3389/fmicb.2024.1341643 (PMC10869596; doi:10.3389/fmicb.2024.1341643)
Supplement: Supplementary file 2 [file Data_Sheet_2.docx]

**Supplementary figures 2**

**Sup Fig. 61 to Sup Fig. 67 Scatter plots,** **leave-one-out plots and funnel plots for the causal association between gut microbiota and daytime dozing.**

**Sup Fig. 68 to Sup Fig. 74 Scatter plots,** **leave-one-out plots and funnel plots for the causal association between gut microbiota and getting up in morning.**

**Sup Fig. 75 to Sup Fig. 80 Scatter plots,** **leave-one-out plots and funnel plots for the causal association between gut microbiota and insomnia.**

**Sup Fig. 81 to Sup Fig. 85 Scatter plots,** **leave-one-out plots and funnel plots for the causal association between gut microbiota and chronotype.**

**Sup Fig. 86 to Sup Fig. 91 Scatter plots,** **leave-one-out plots and funnel plots for the causal association between gut microbiota and nap during day.**

**Sup Fig. 92 to Sup Fig. 101 Scatter plots,** **leave-one-out plots and funnel plots for the causal association between gut microbiota and sleep duration.**

**Sup Fig. 102 to Sup Fig. 107 Scatter plots,** **leave-one-out plots and funnel plots for the causal association between gut microbiota and snoring.**

**Sup Fig. 61** **Scatter plot,** **leave-one-out plot and funnel plot for the causal association between *class Gammaproteobacteria* and daytime dozing.**

**
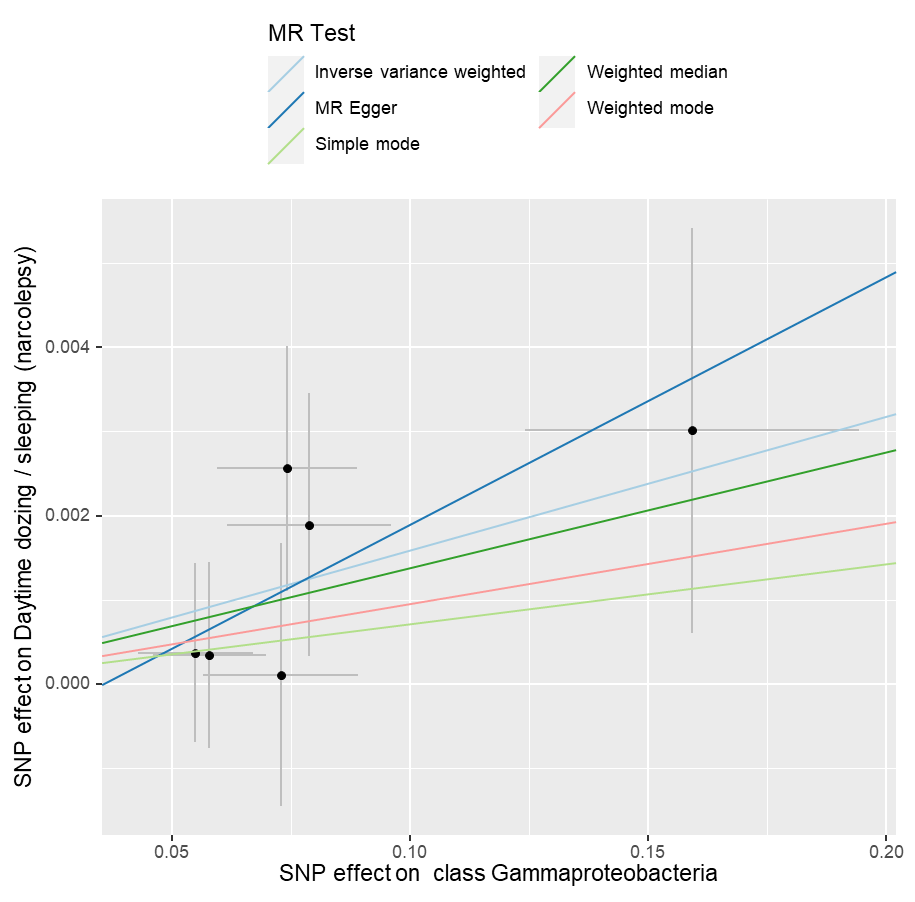

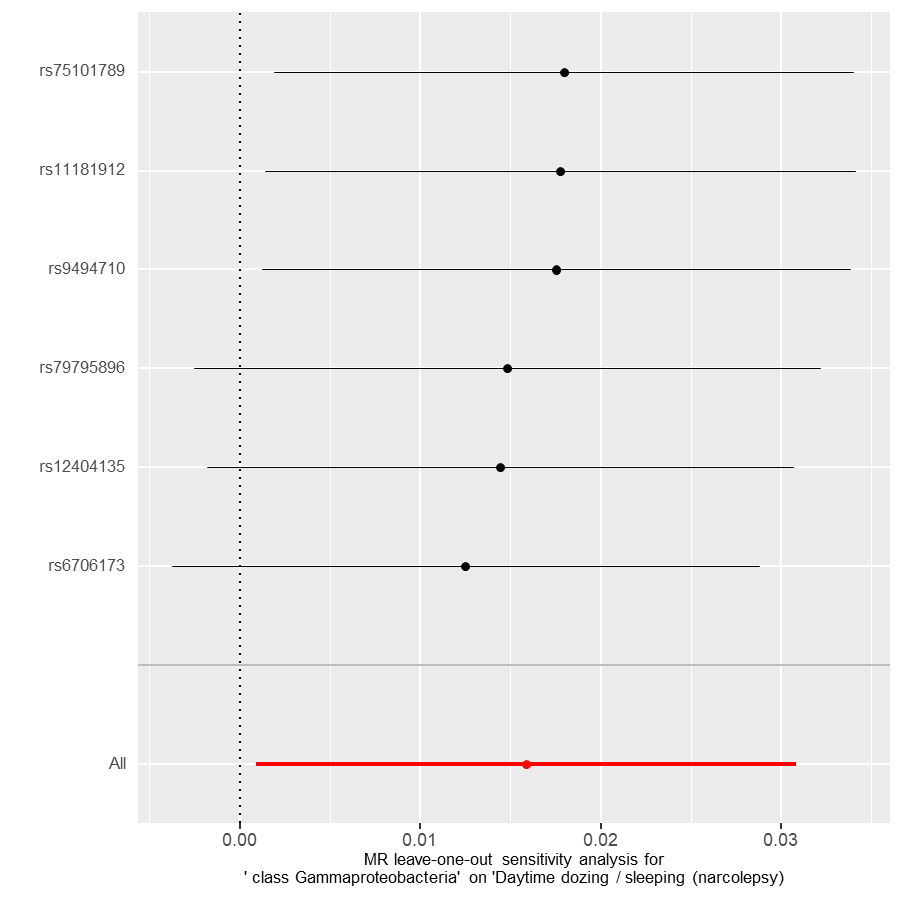

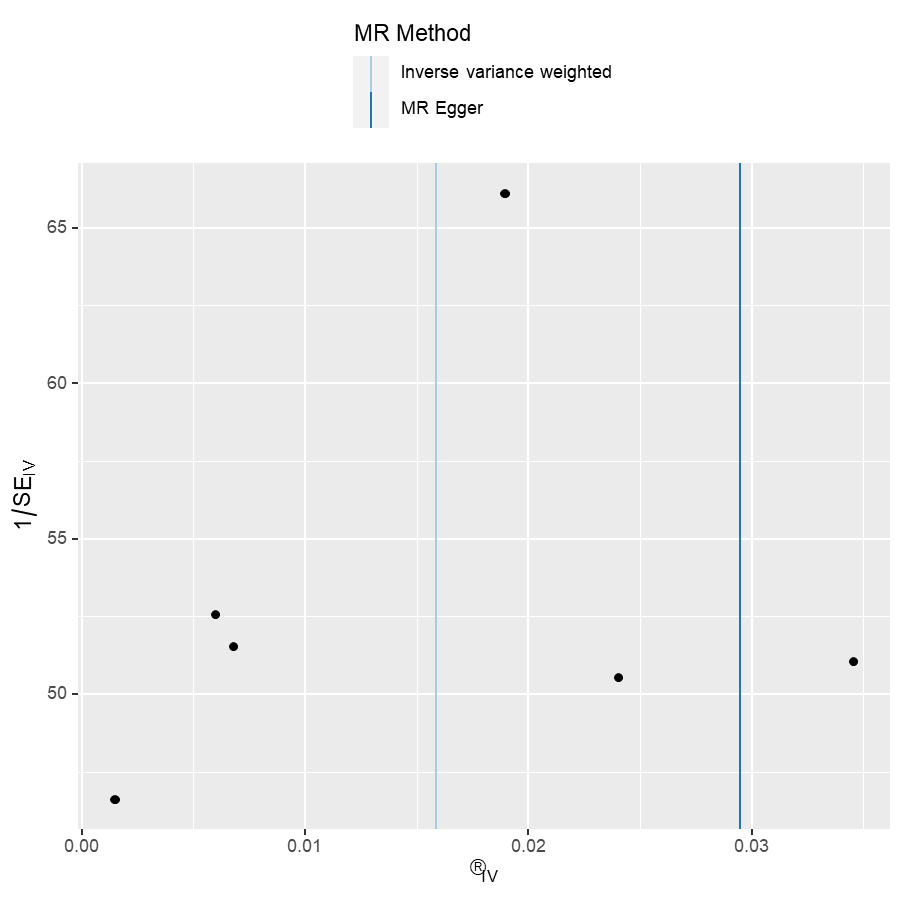
**

**Sup Fig. 62 Scatter plot,** **leave-one-out plot and funnel plot for the causal association between *genus Butyricimonas* and daytime dozing.**

**
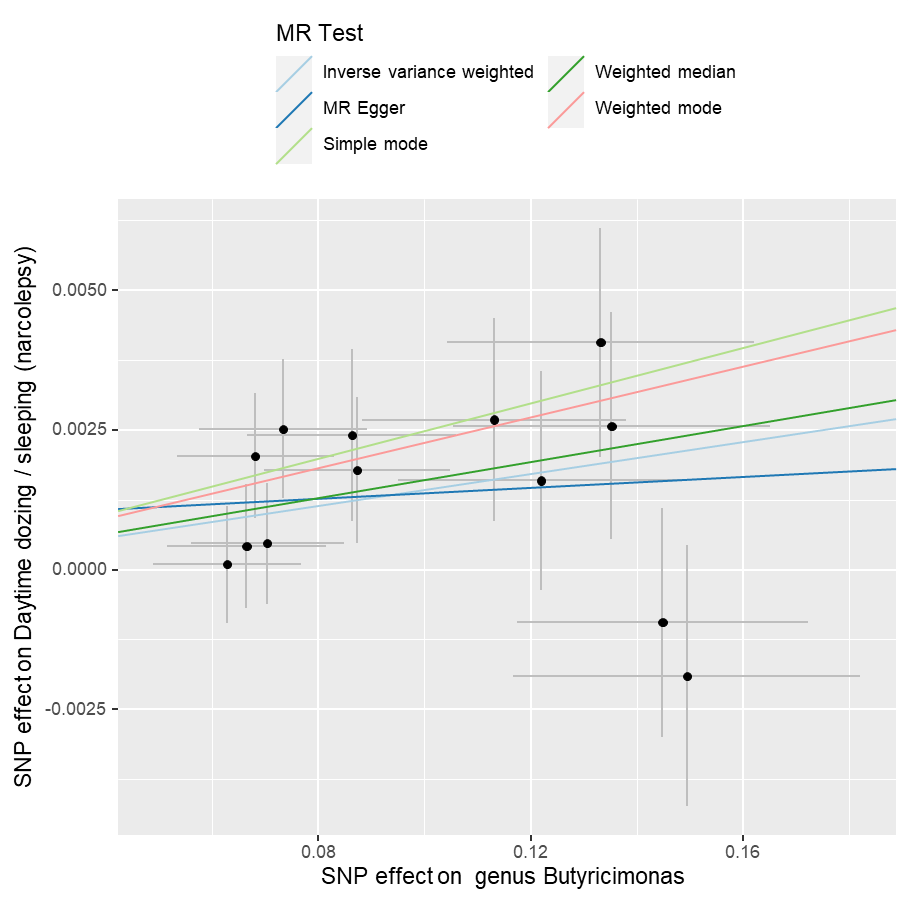

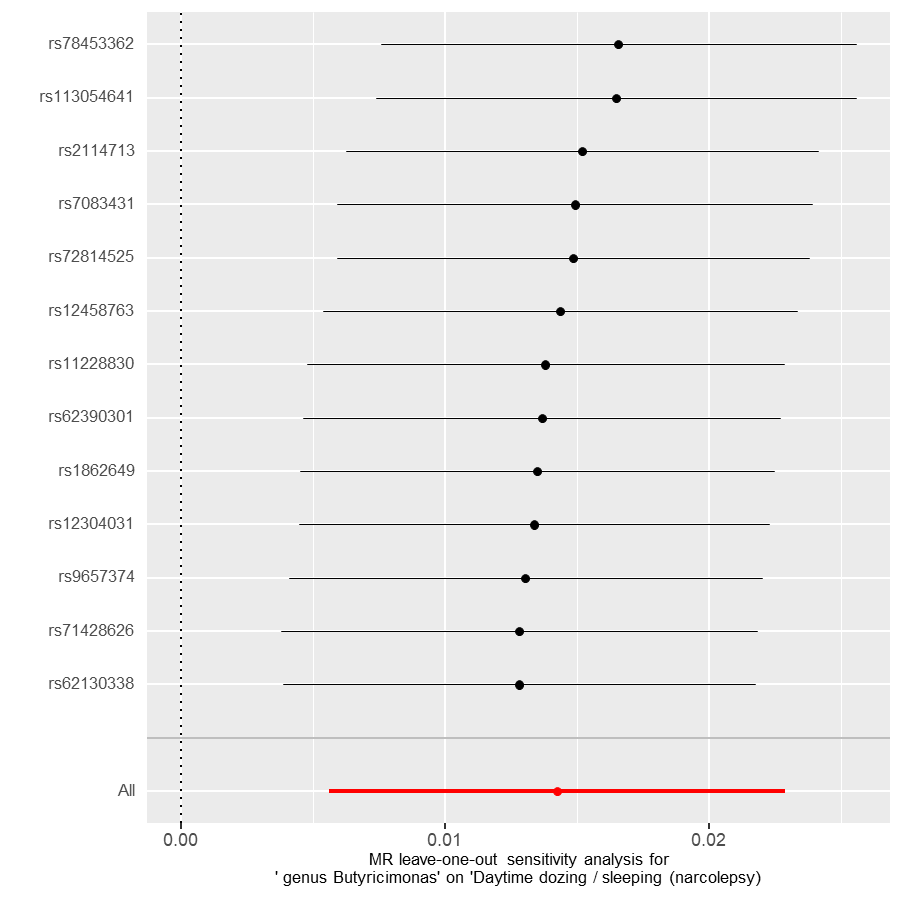

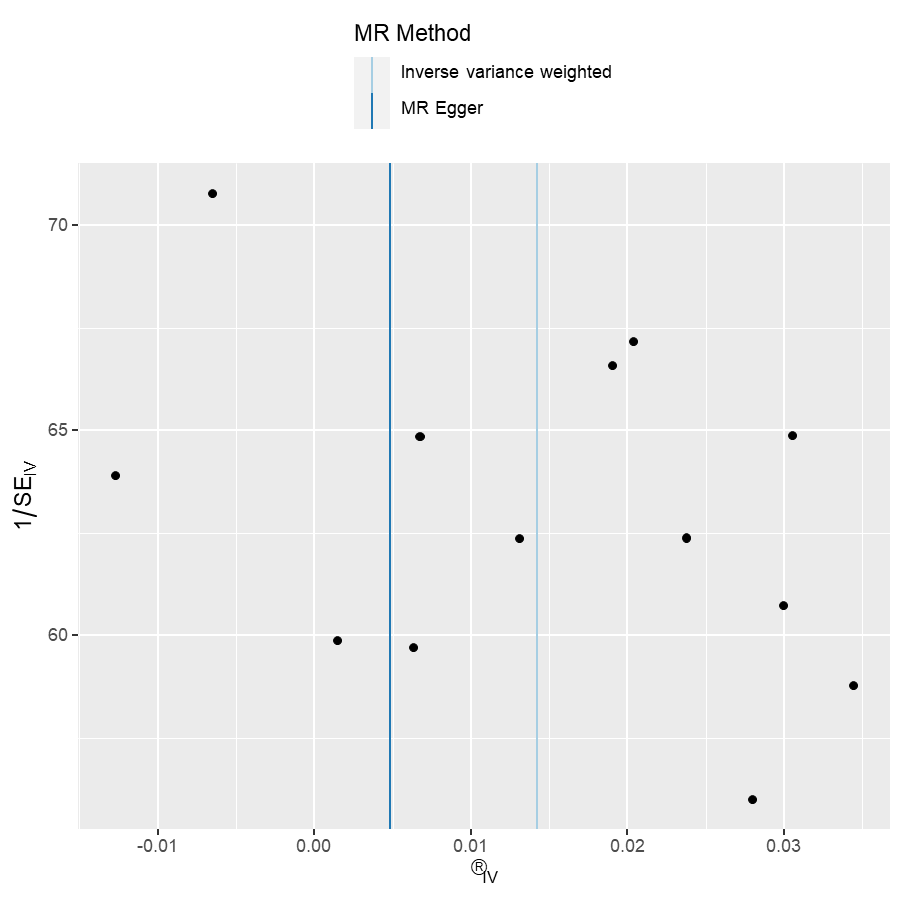
**

**Sup Fig. 63 Scatter plot,** **leave-one-out plot and funnel plot for the causal association between *Clostridium sensustricto1* and daytime dozing.**

**
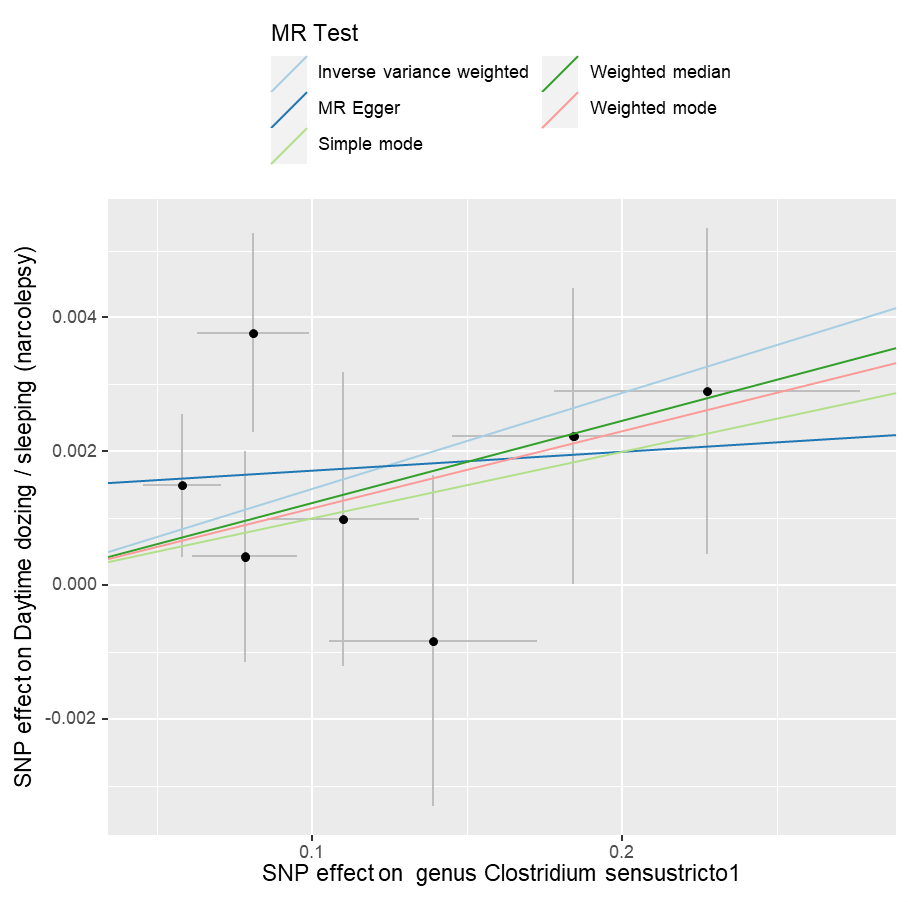

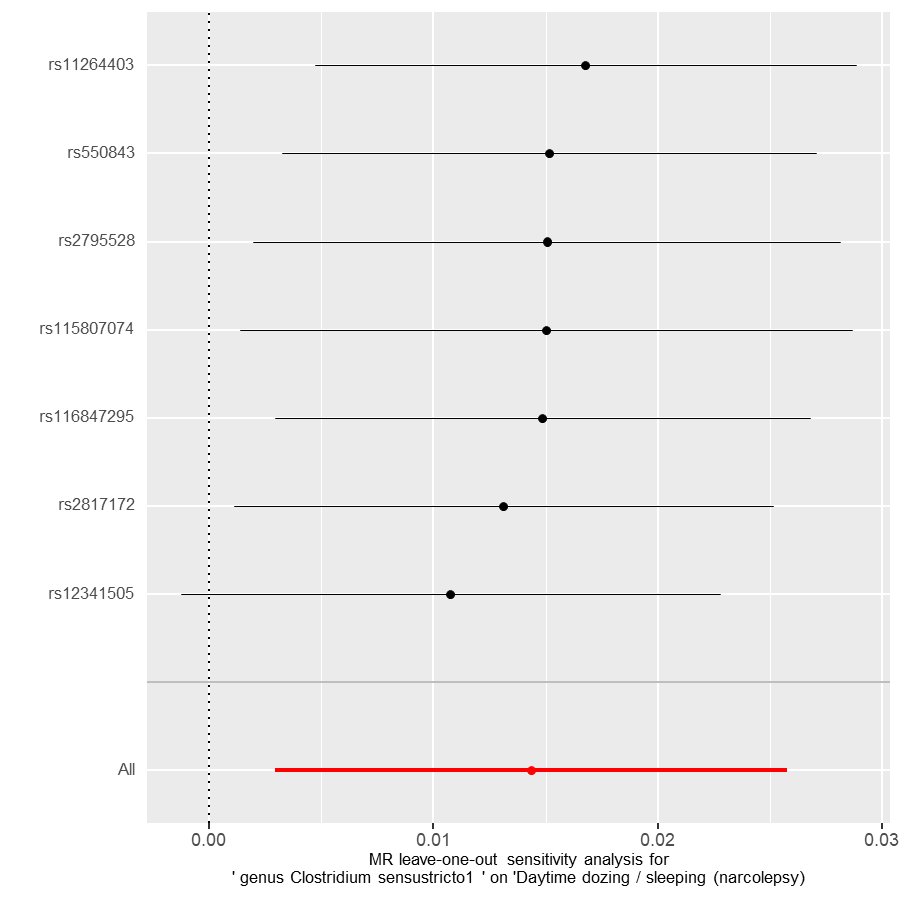

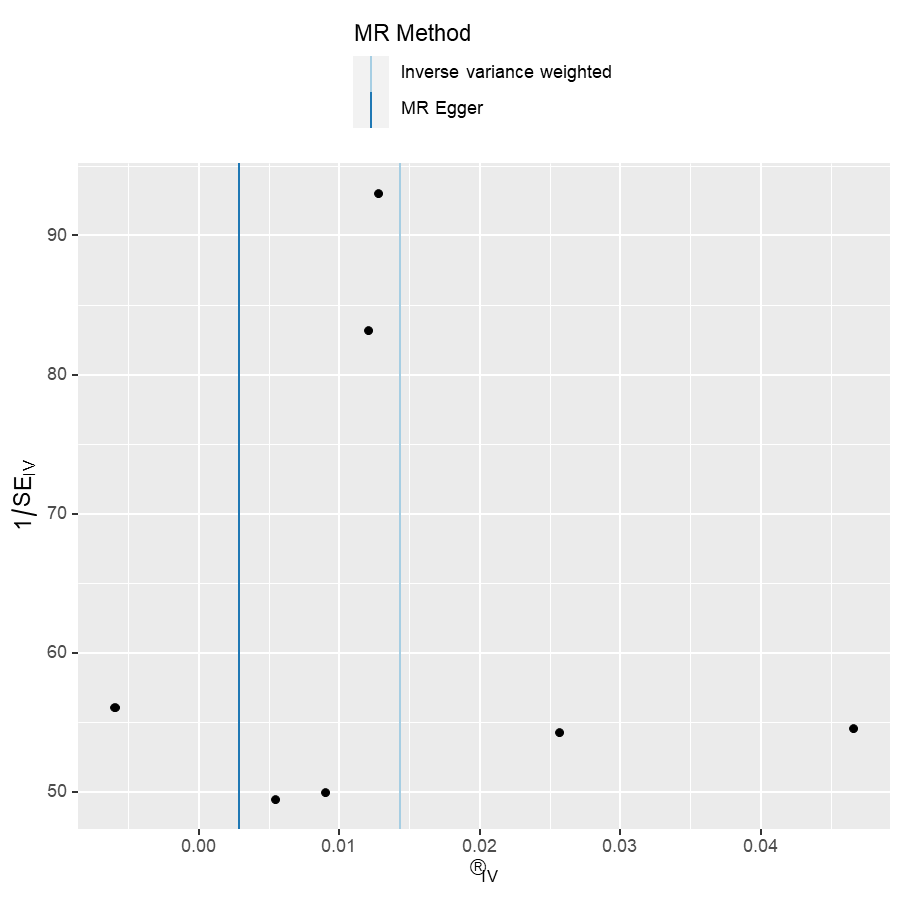
**

**Sup Fig. 64 Scatter plot,** **leave-one-out plot and funnel plot for the causal association between *genus Coprococcus2* and daytime dozing.**

**
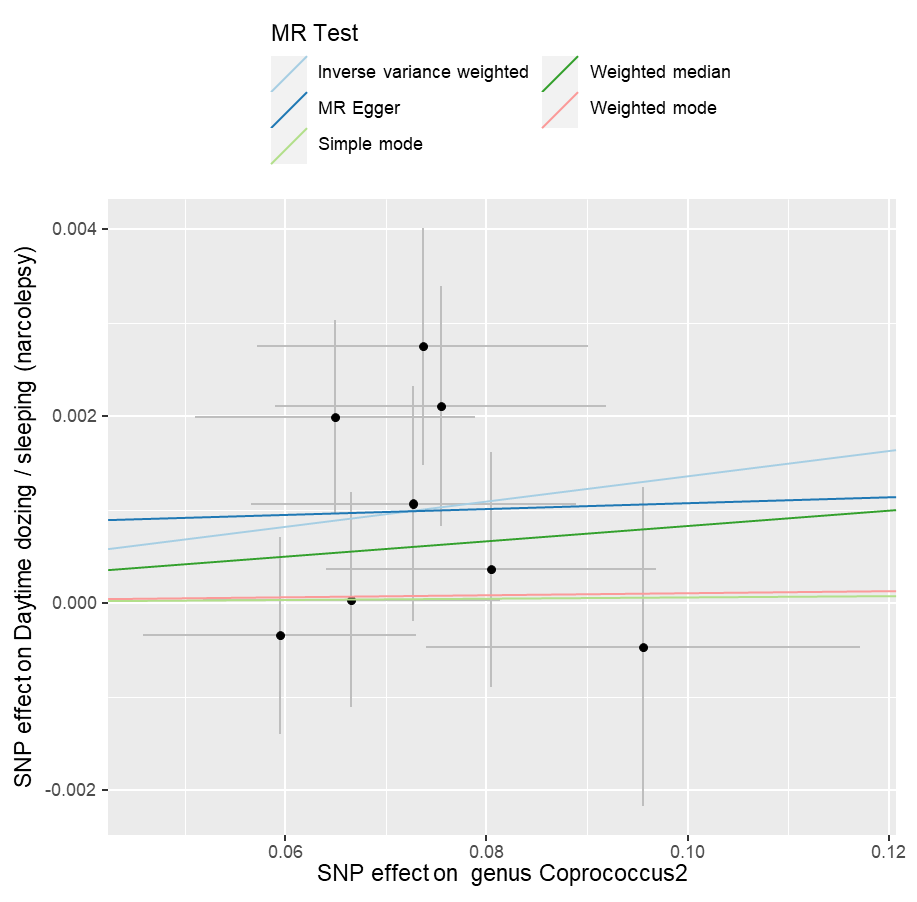

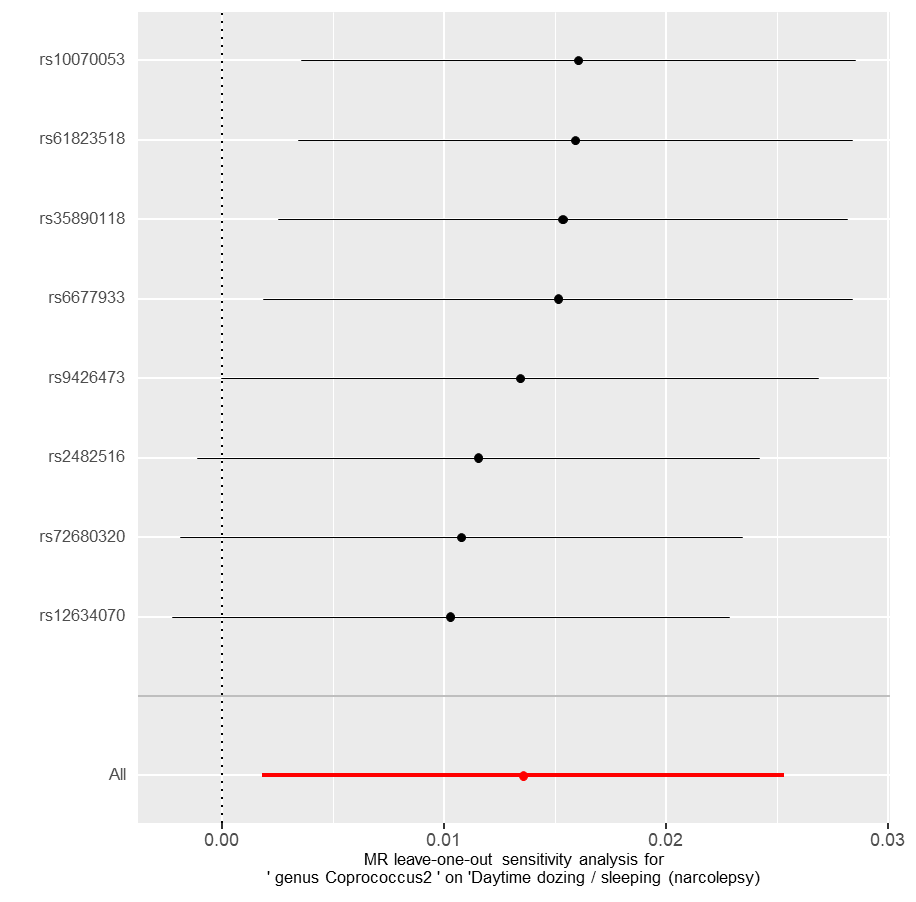

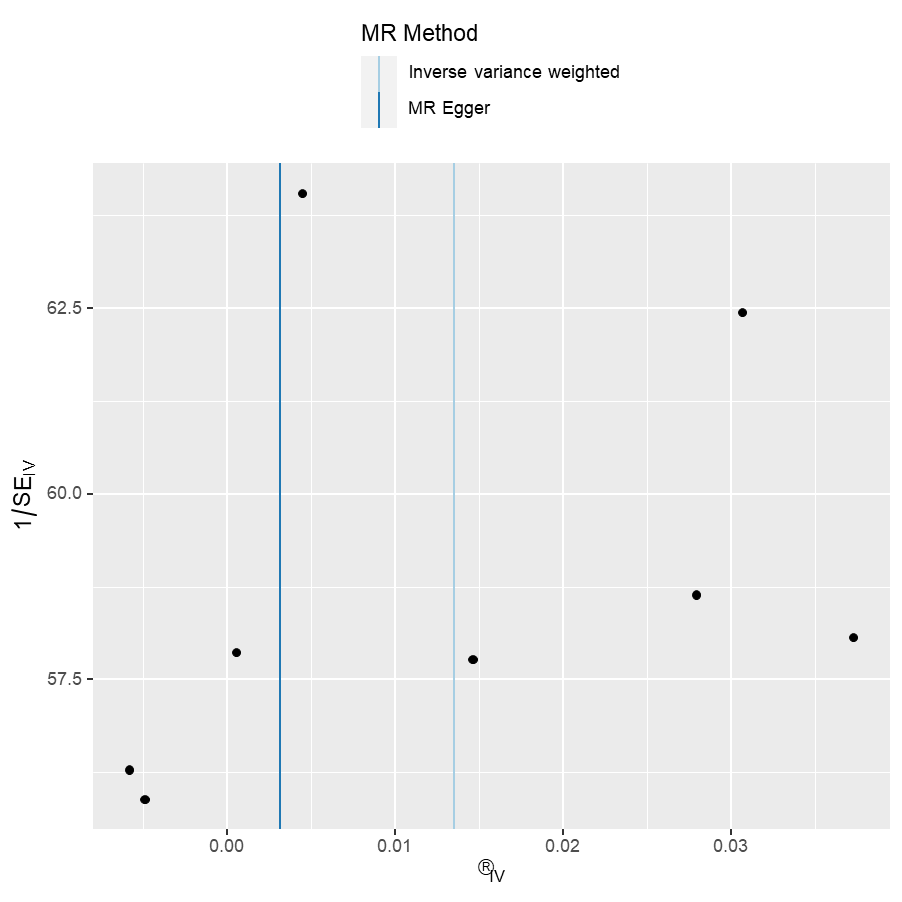
**

**Sup Fig. 65 Scatter plot,** **leave-one-out plot and funnel plot for the causal association between *genus Eubacterium eligens group* and daytime dozing.**

**
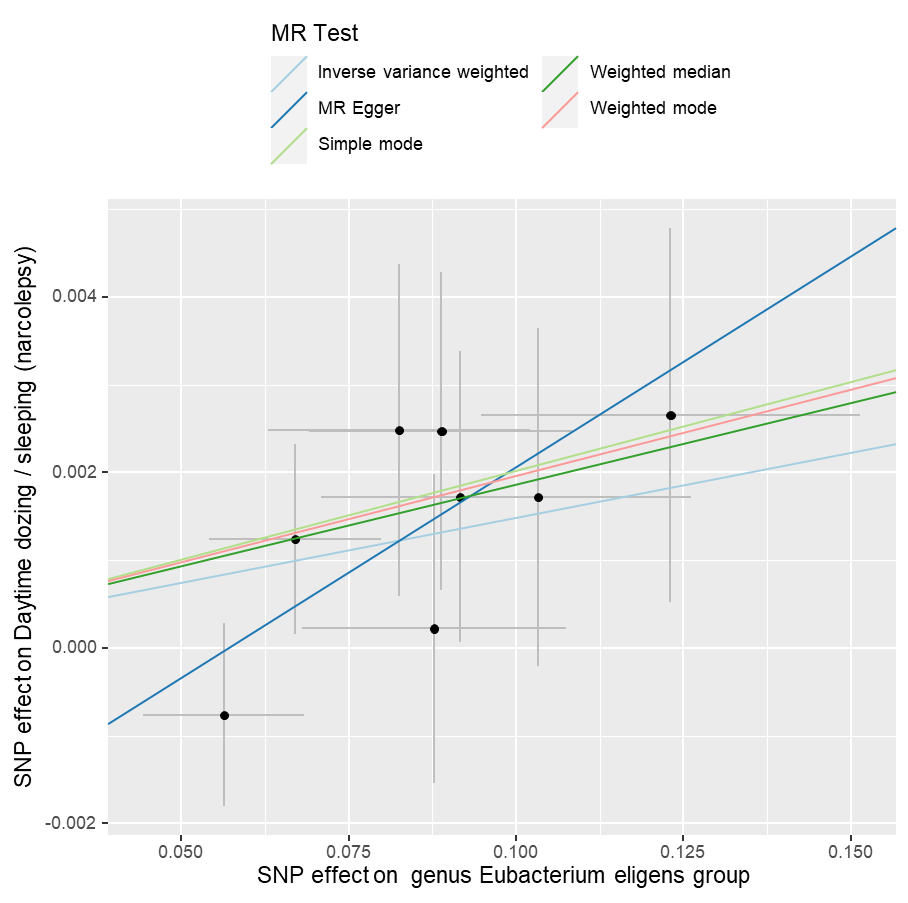

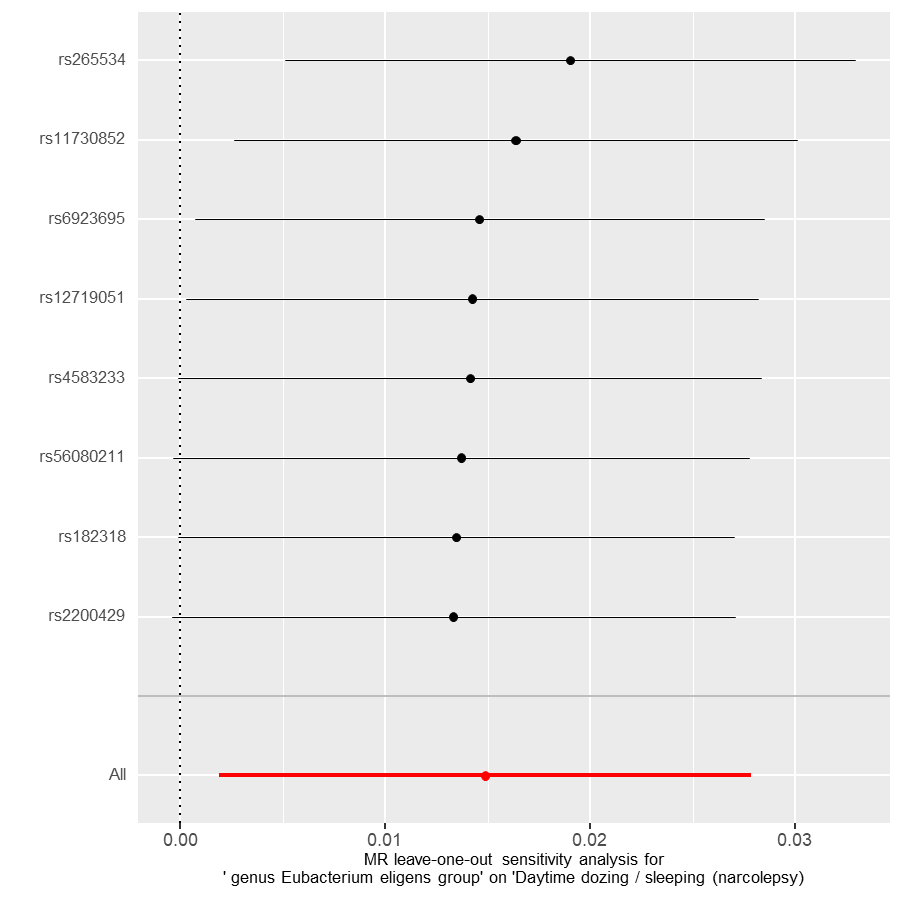

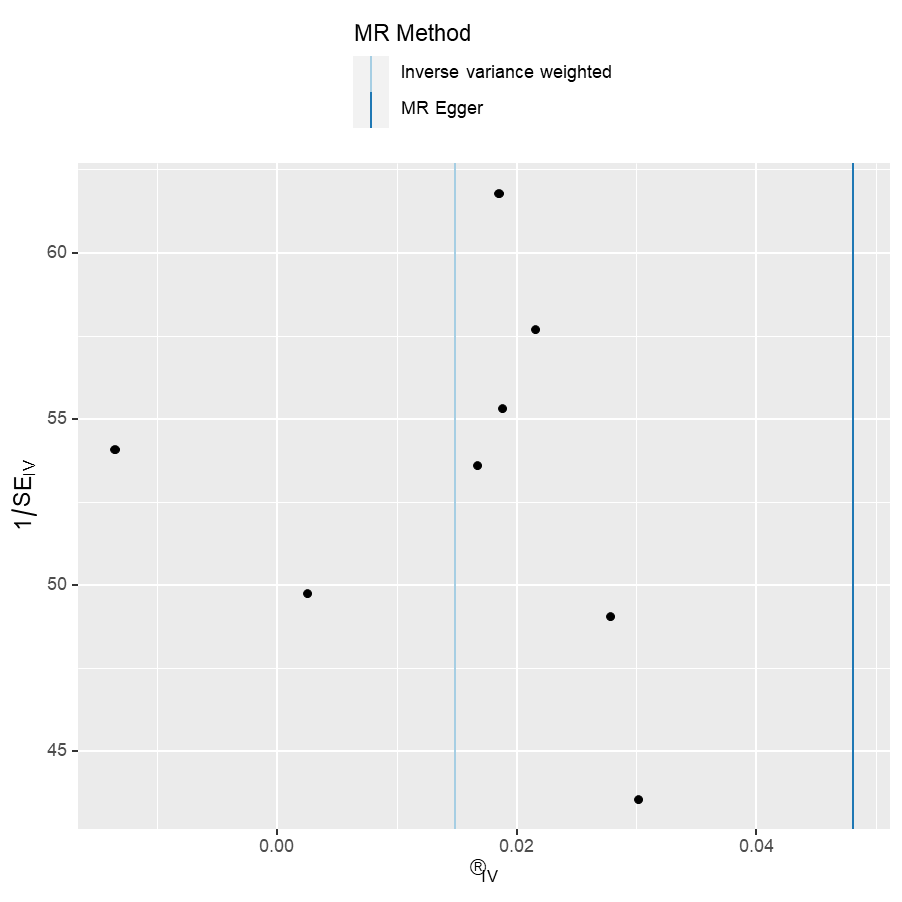
**

**Sup Fig. 66 Scatter plot,** **leave-one-out plot and funnel plot for the causal association between *genus Intestinibacter* and daytime dozing.**

**
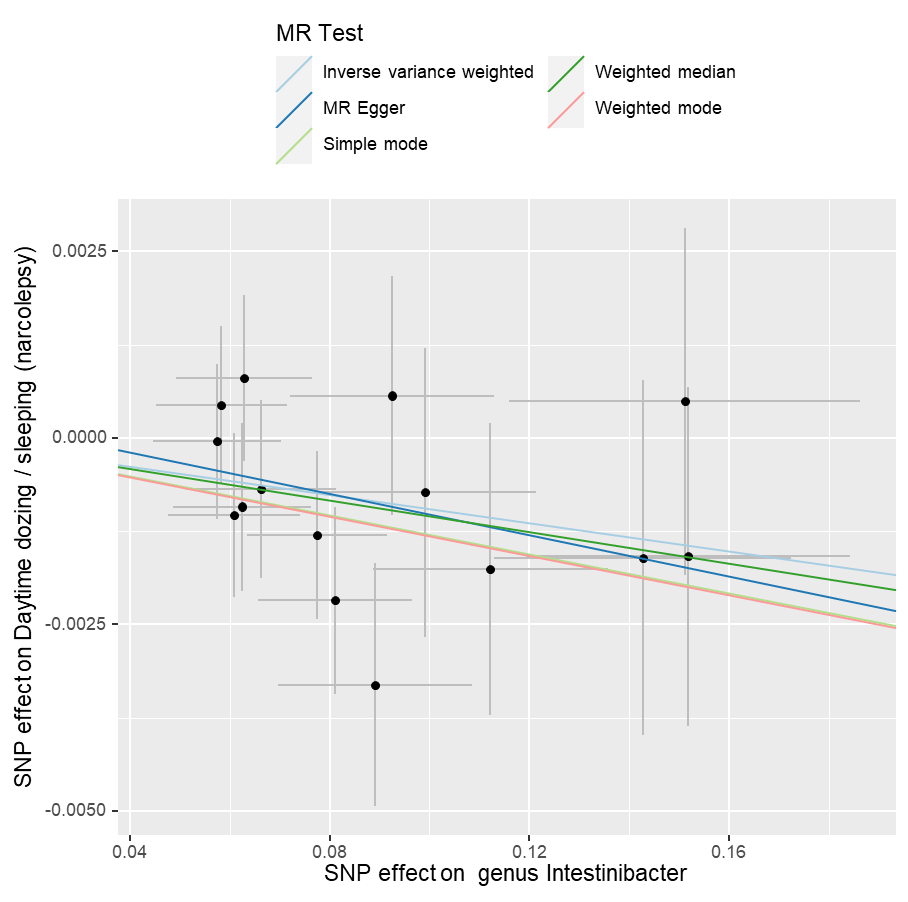

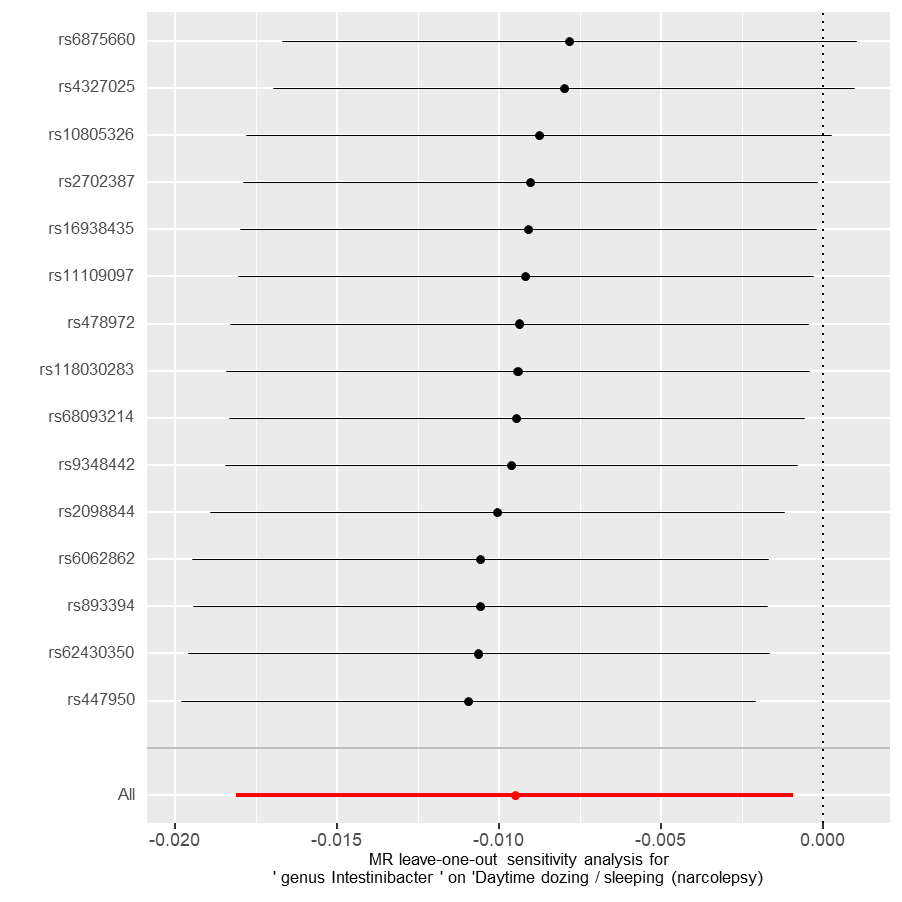

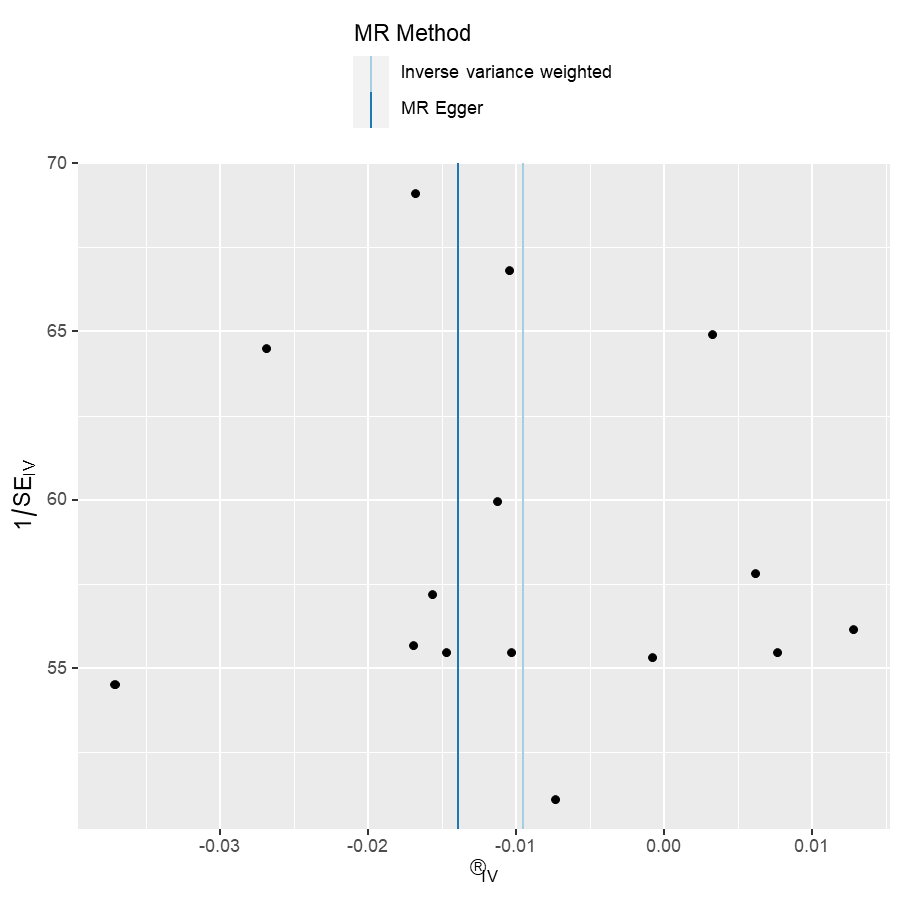
**

**Sup Fig. 67 Scatter plot,** **leave-one-out plot and funnel plot for the causal association between *phylum Bacteroidetes* and daytime dozing.**

**
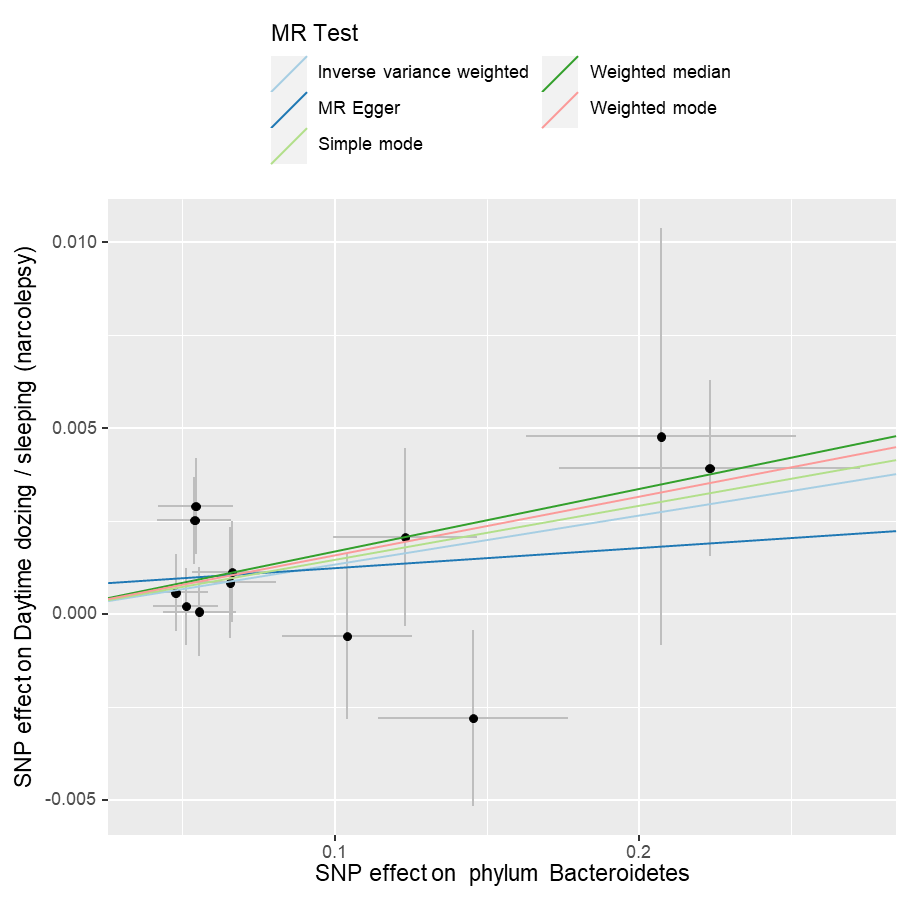

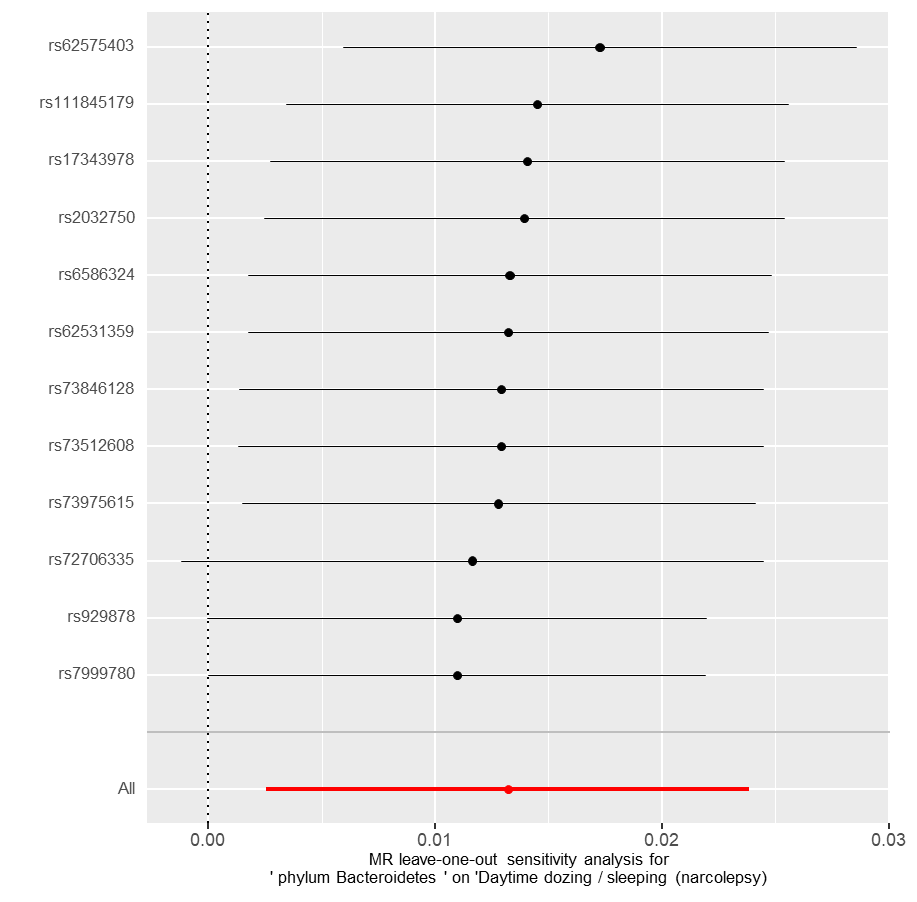

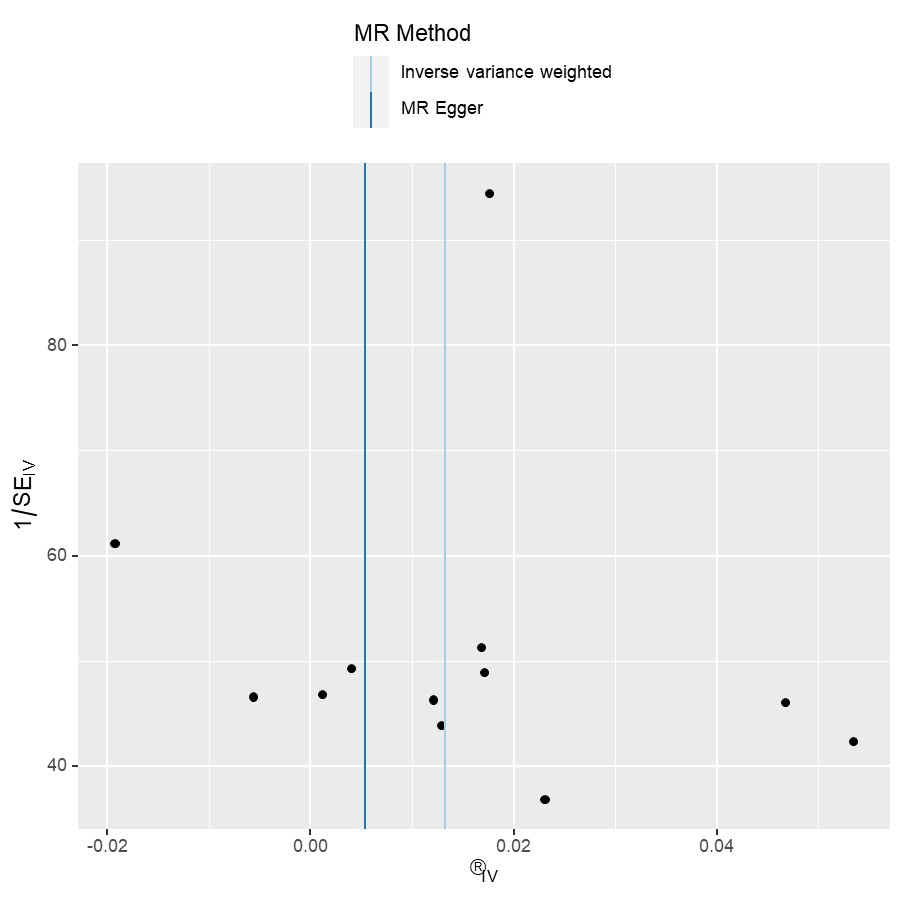
**

**Sup Fig. 68 Scatter plot,** **leave-one-out plot and funnel plot for the causal association between *class Clostridia* and getting up in morning.**

**
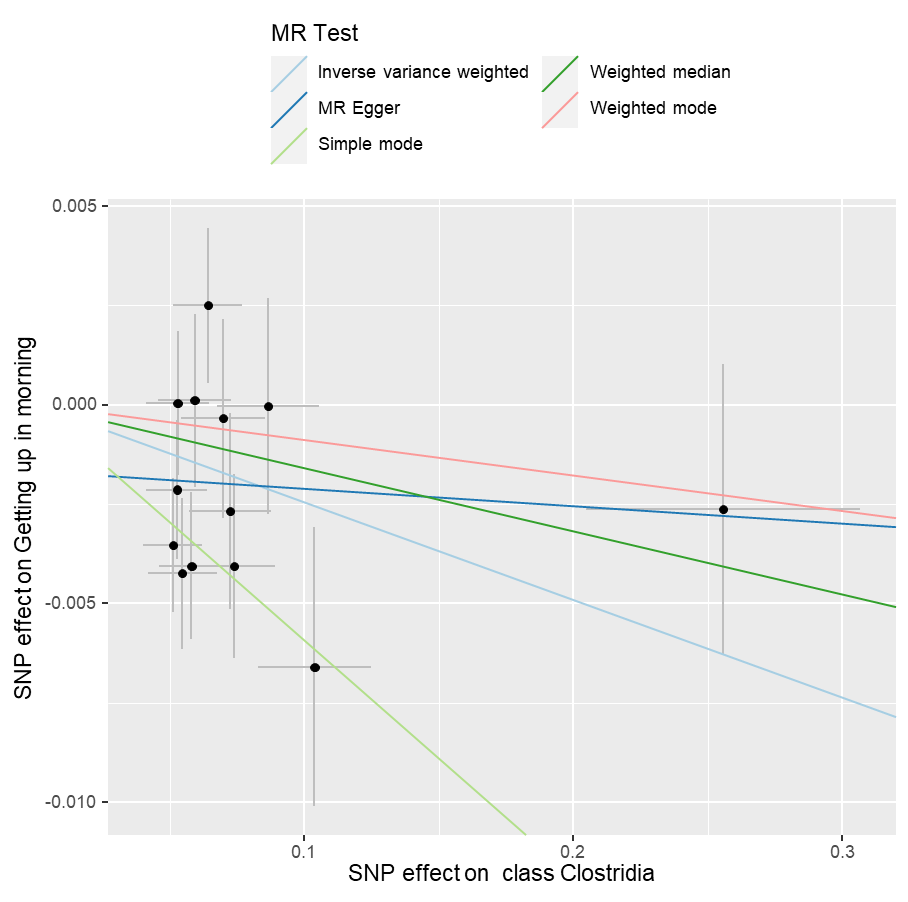

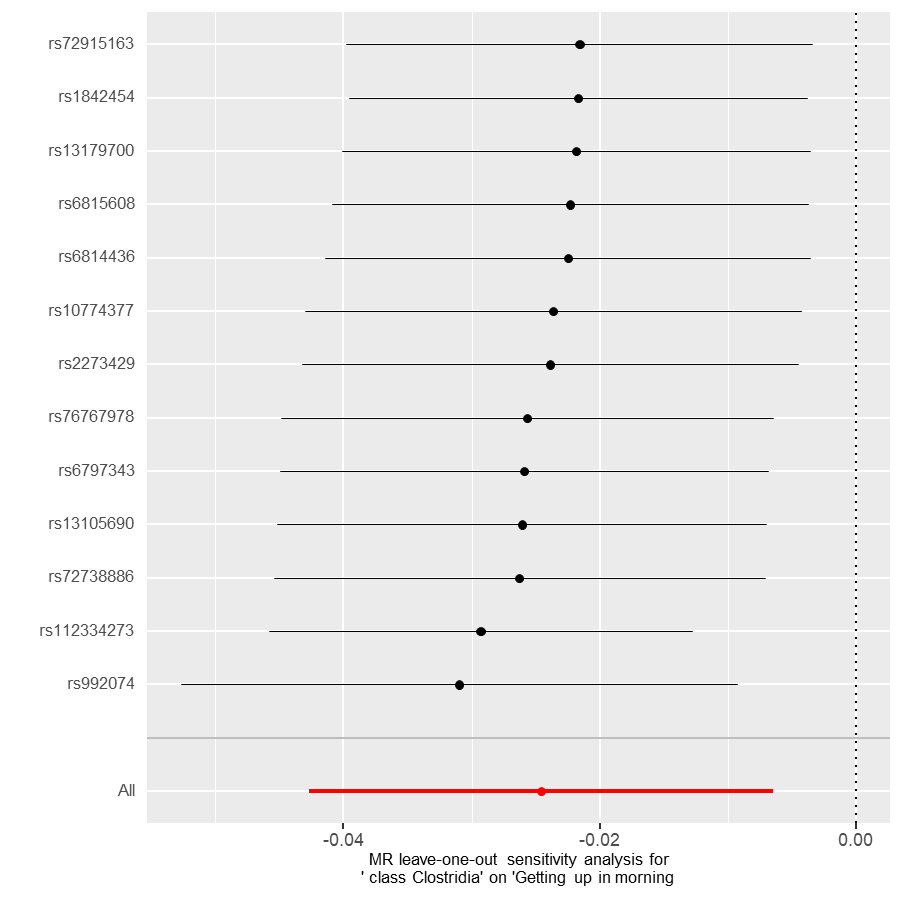

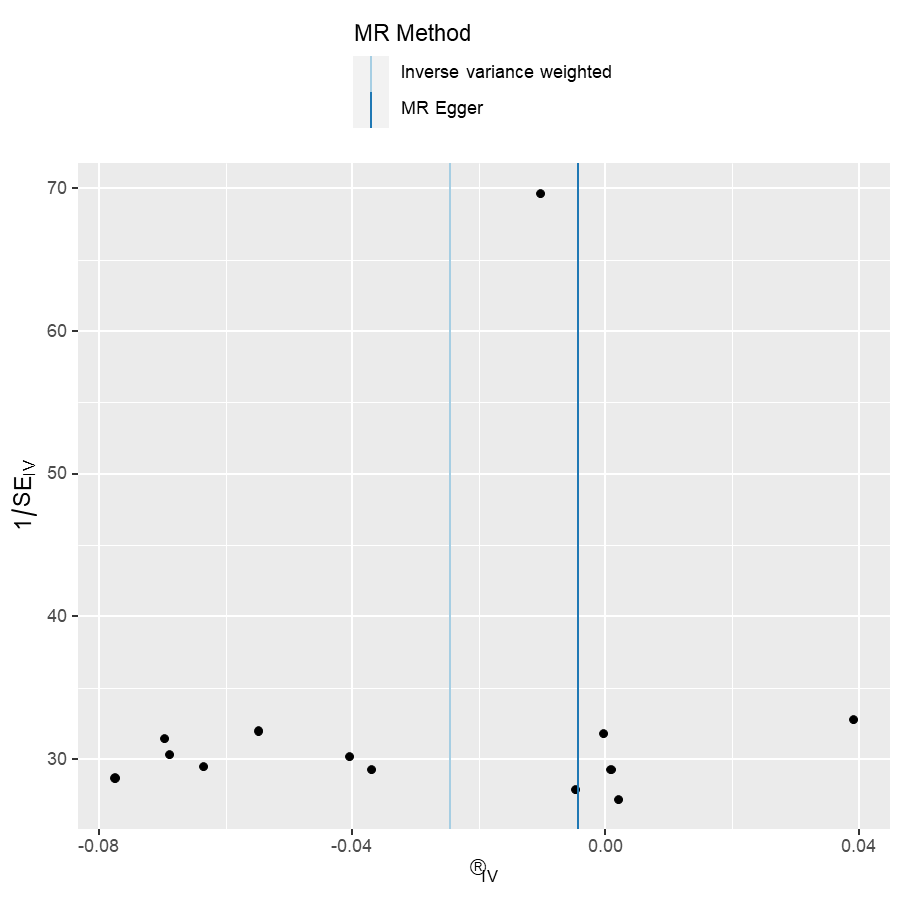
**

**Sup Fig. 69 Scatter plot,** **leave-one-out plot and funnel plot for the causal association between *order Bifidobacteriales* and getting up in morning.**

**
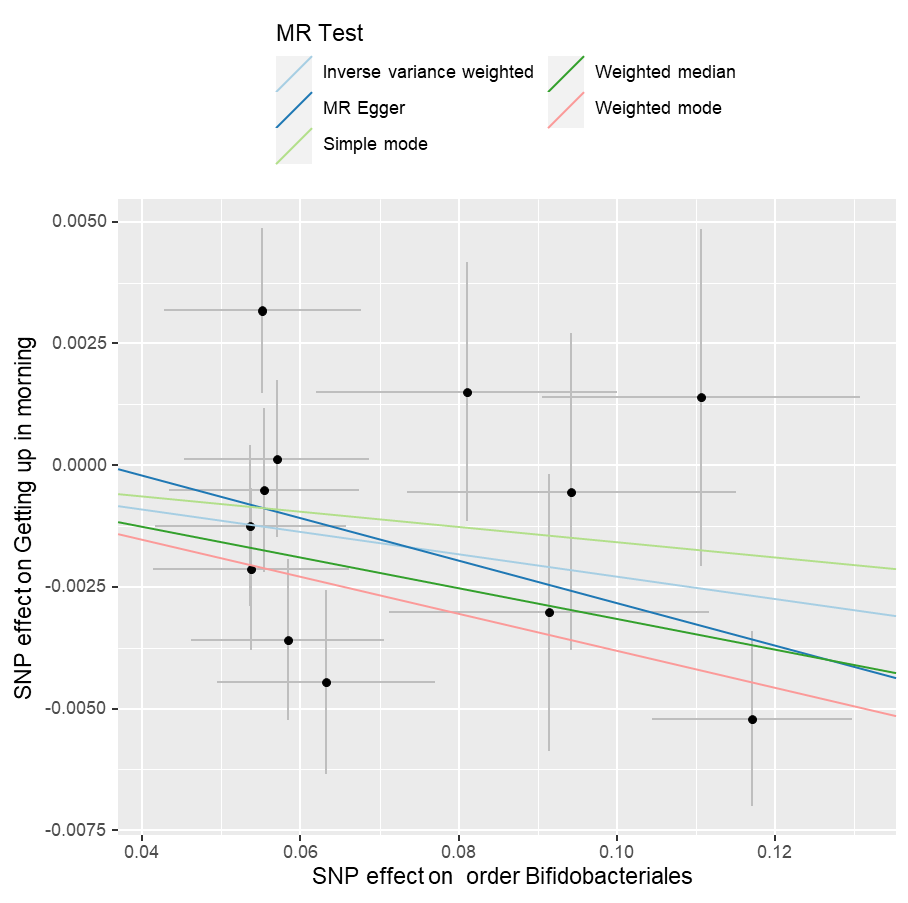

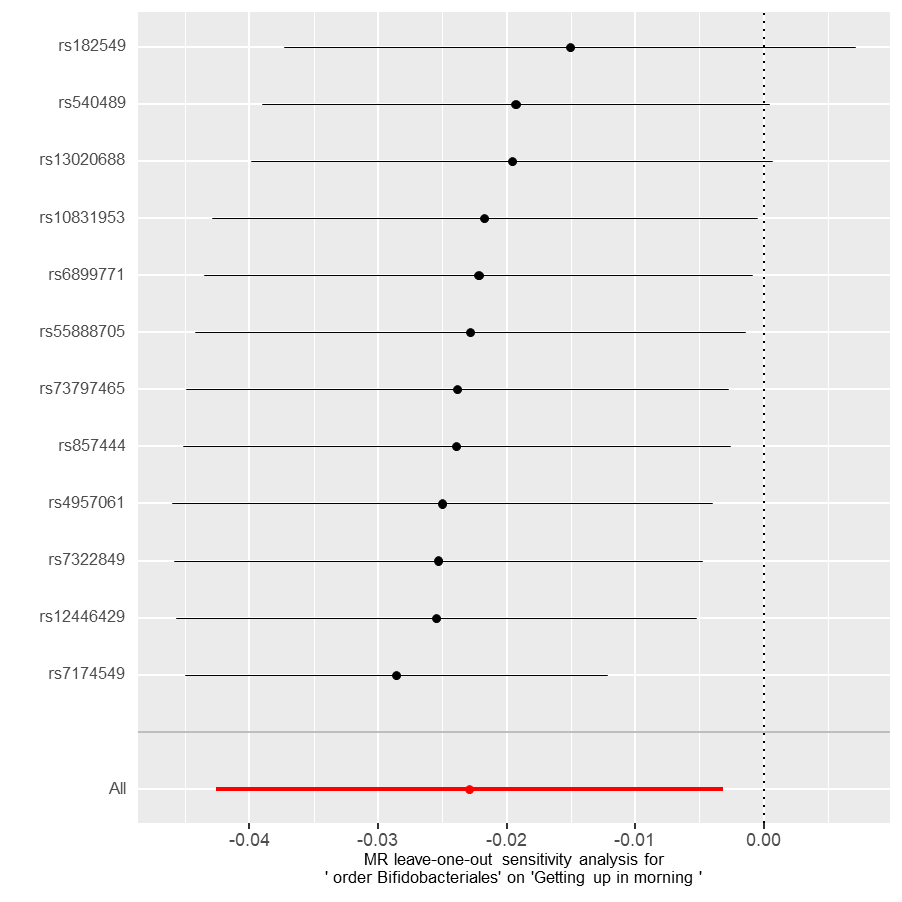

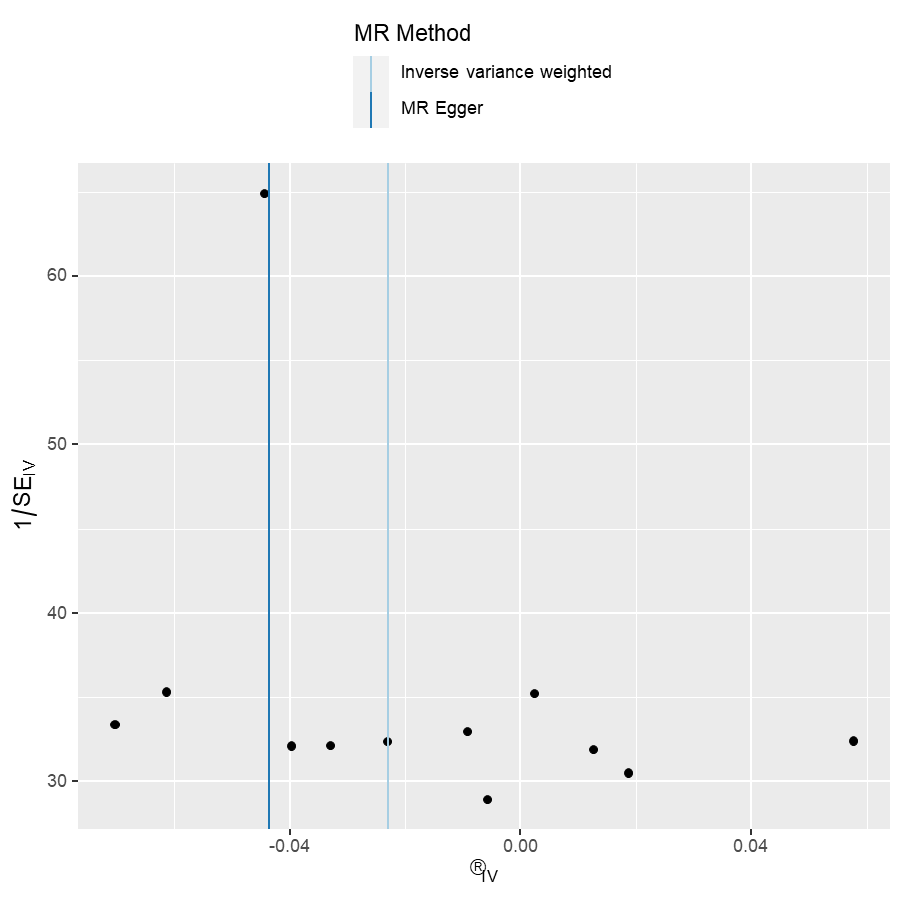
**

**Sup Fig. 70 Scatter plot,** **leave-one-out plot and funnel plot for the causal association between *family Peptococcaceae* and getting up in morning.**

**
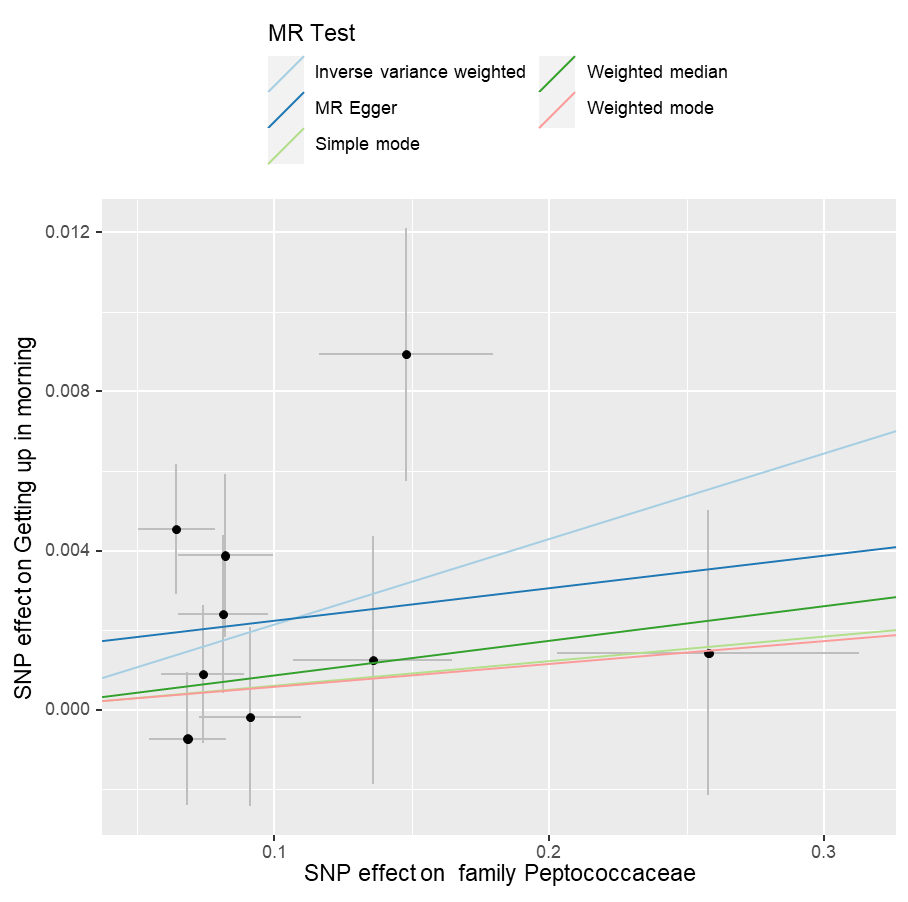

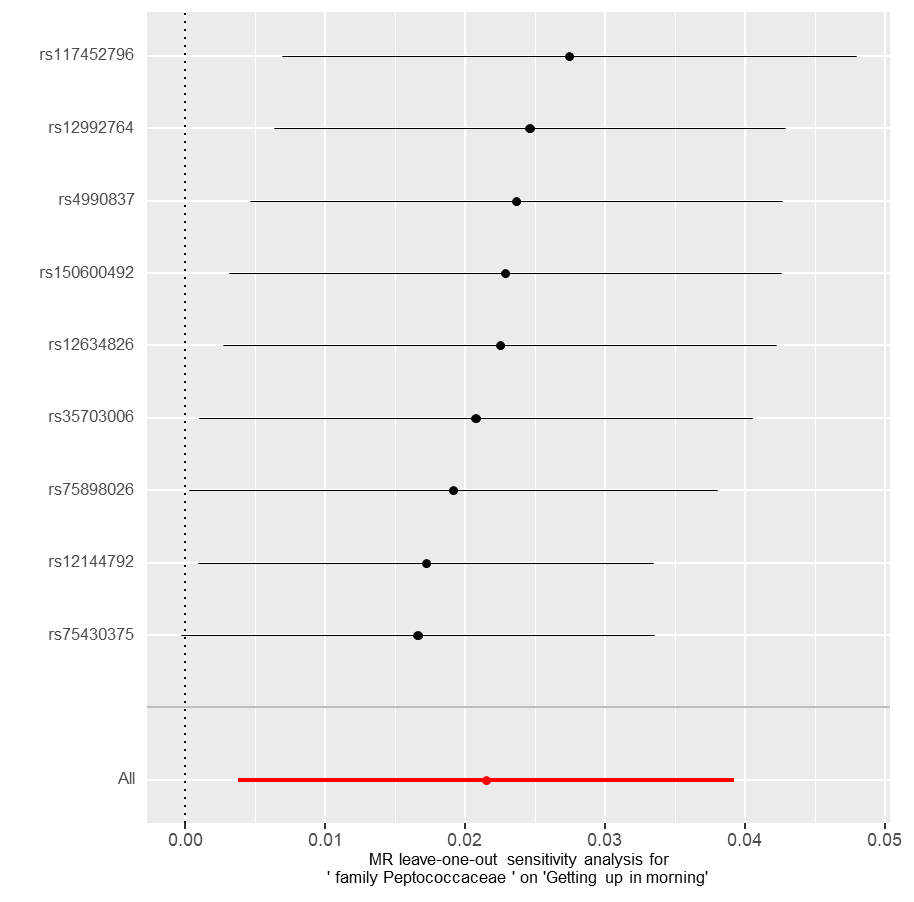

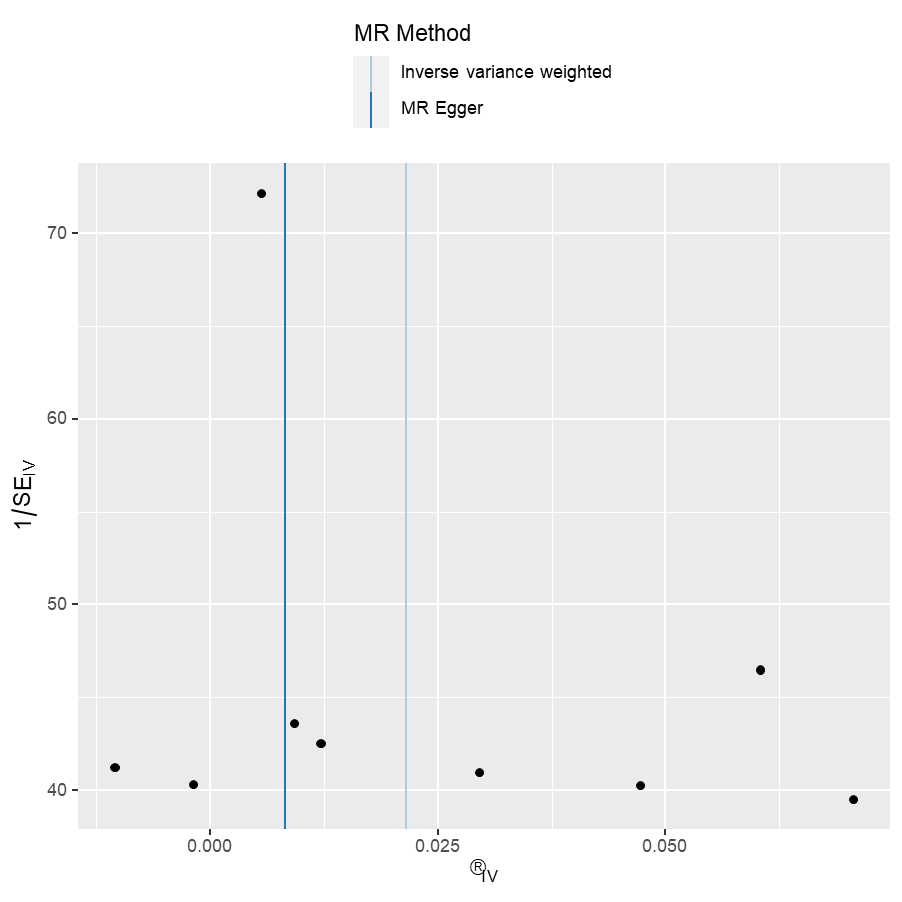
**

**Sup Fig. 71 Scatter plot,** **leave-one-out plot and funnel plot for the causal association between *genus Anaerofilum* and getting up in morning.**

**
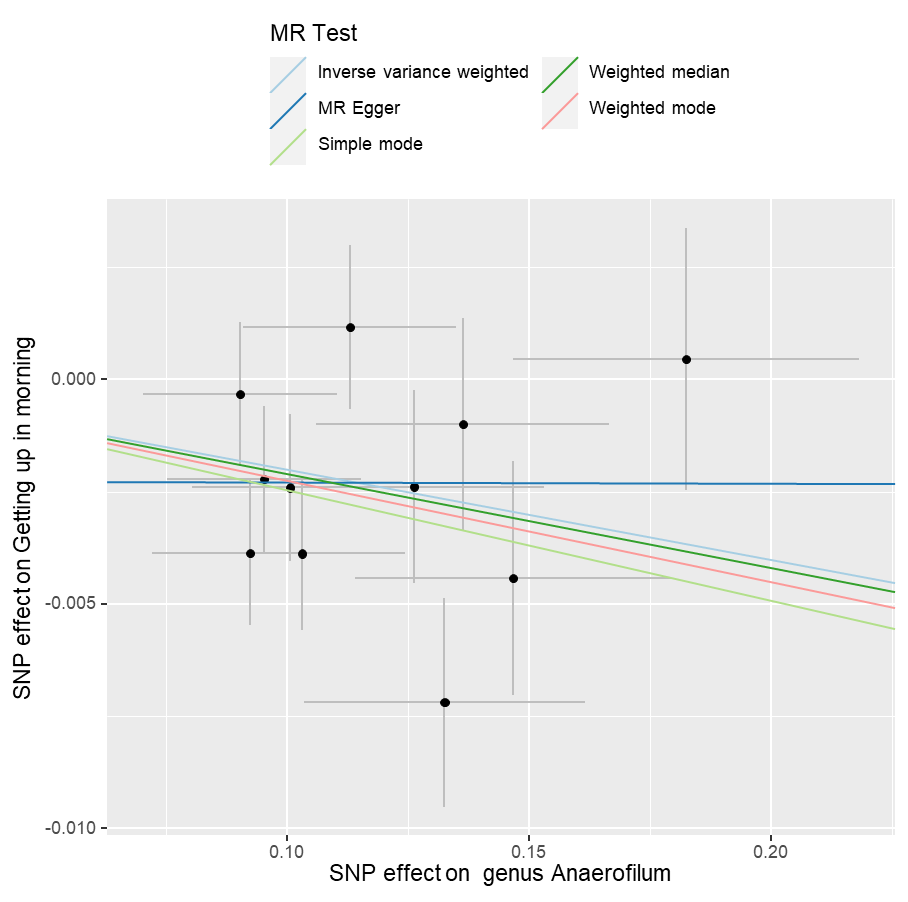

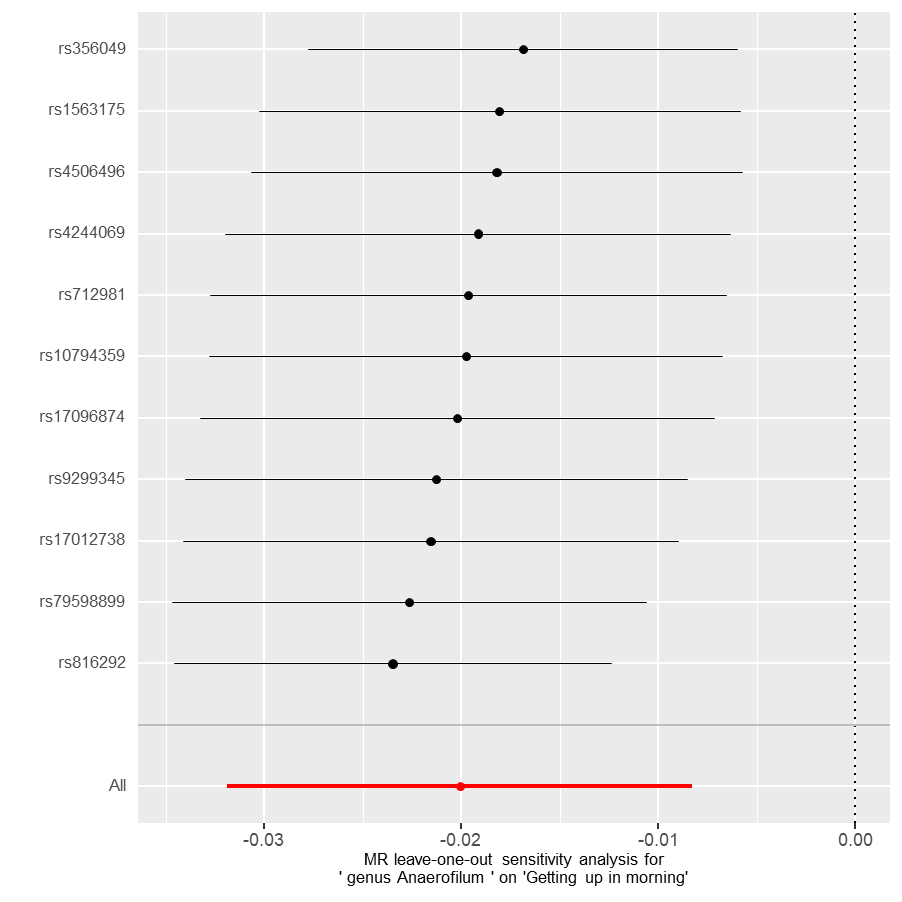

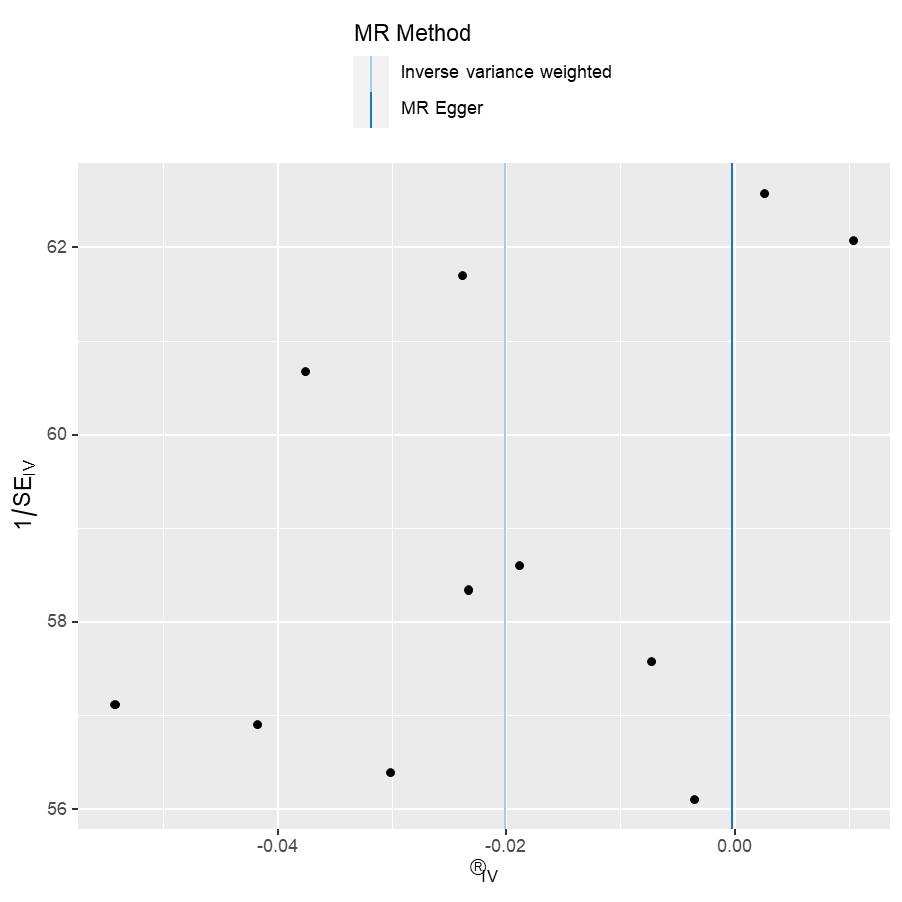
**

**Sup Fig. 72 Scatter plot,** **leave-one-out plot and funnel plot for the causal association between *genus Intestinimonas* and getting up in morning.**

**
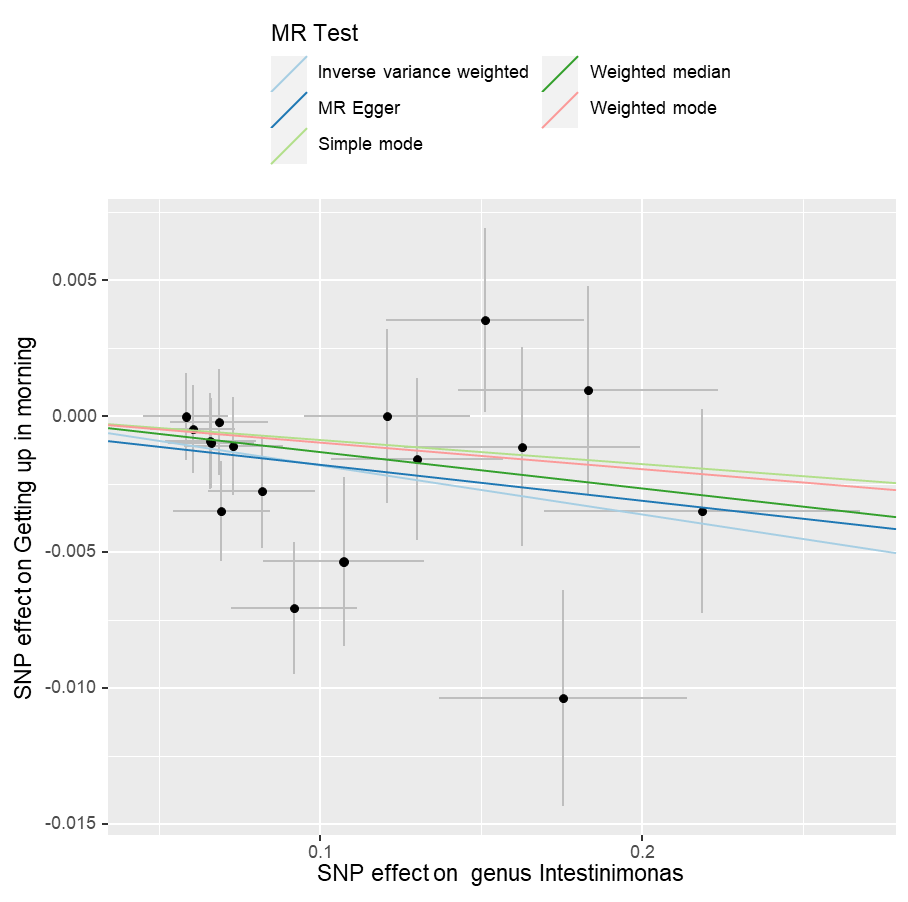

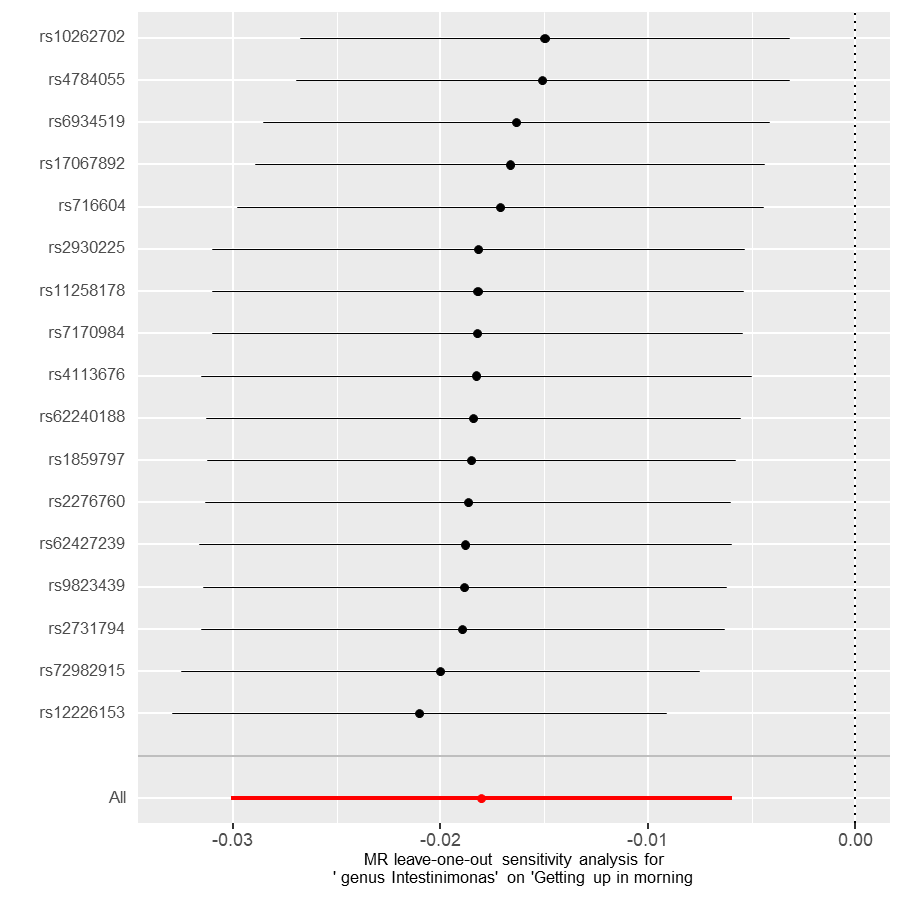

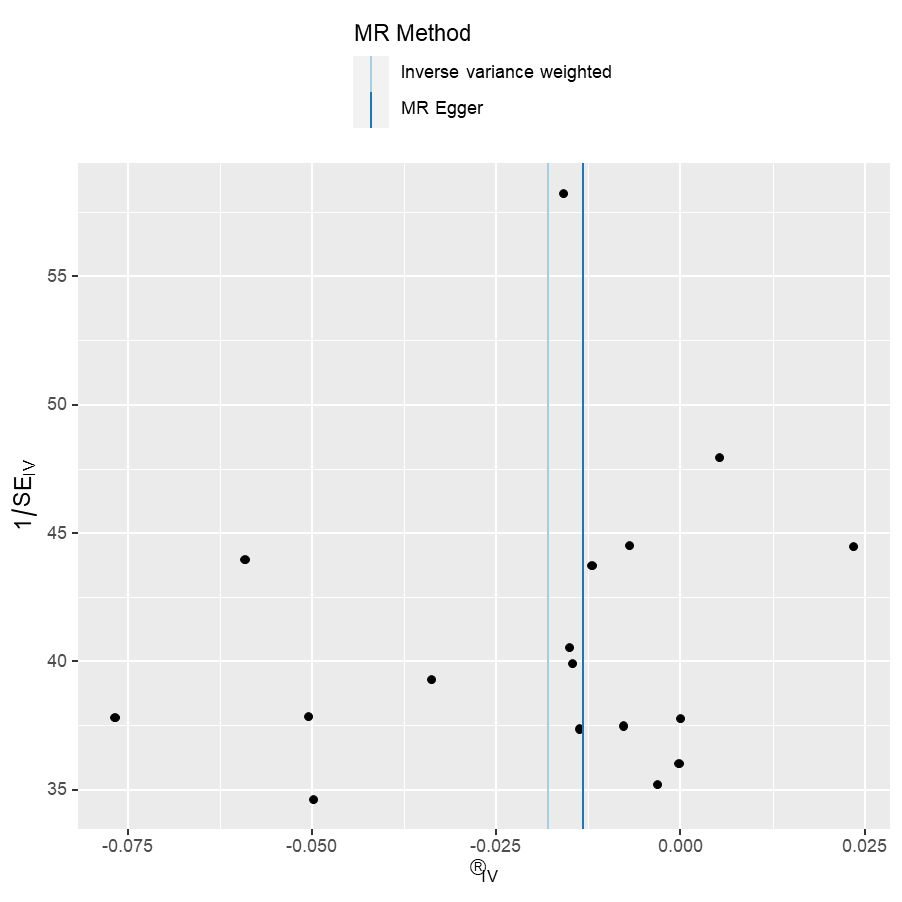
**

**Sup Fig. 73 Scatter plot,** **leave-one-out plot and funnel plot for the causal association between *family Bifidobacteriaceae* and getting up in morning.**

**
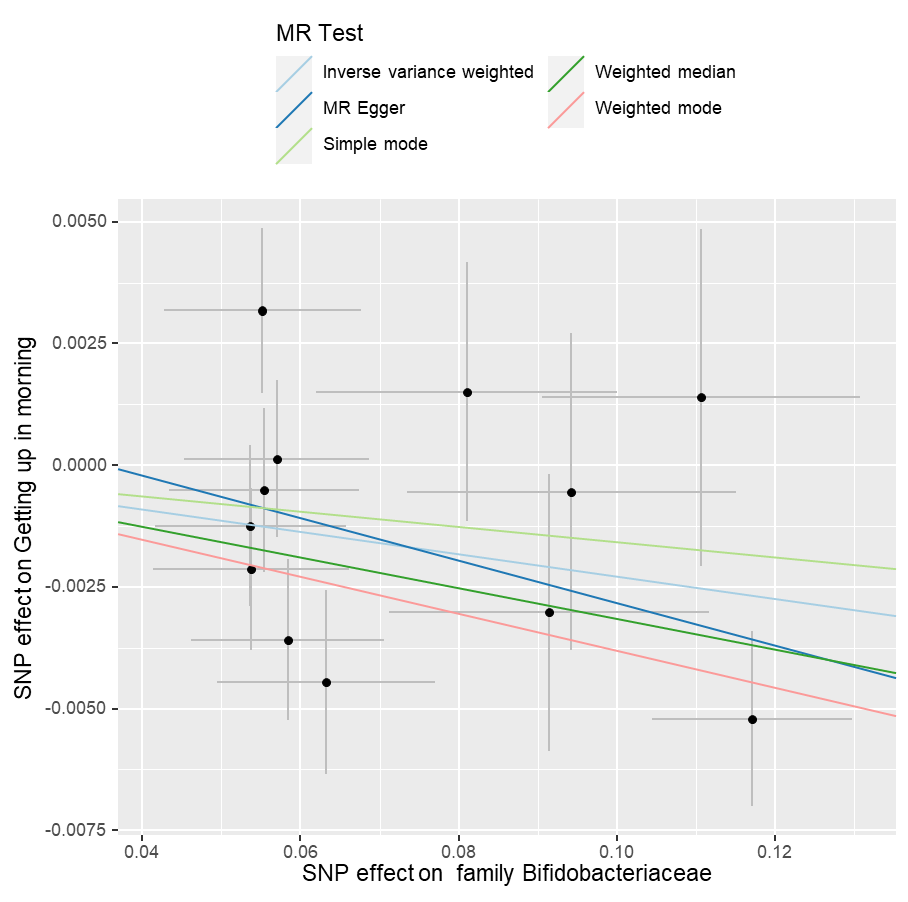

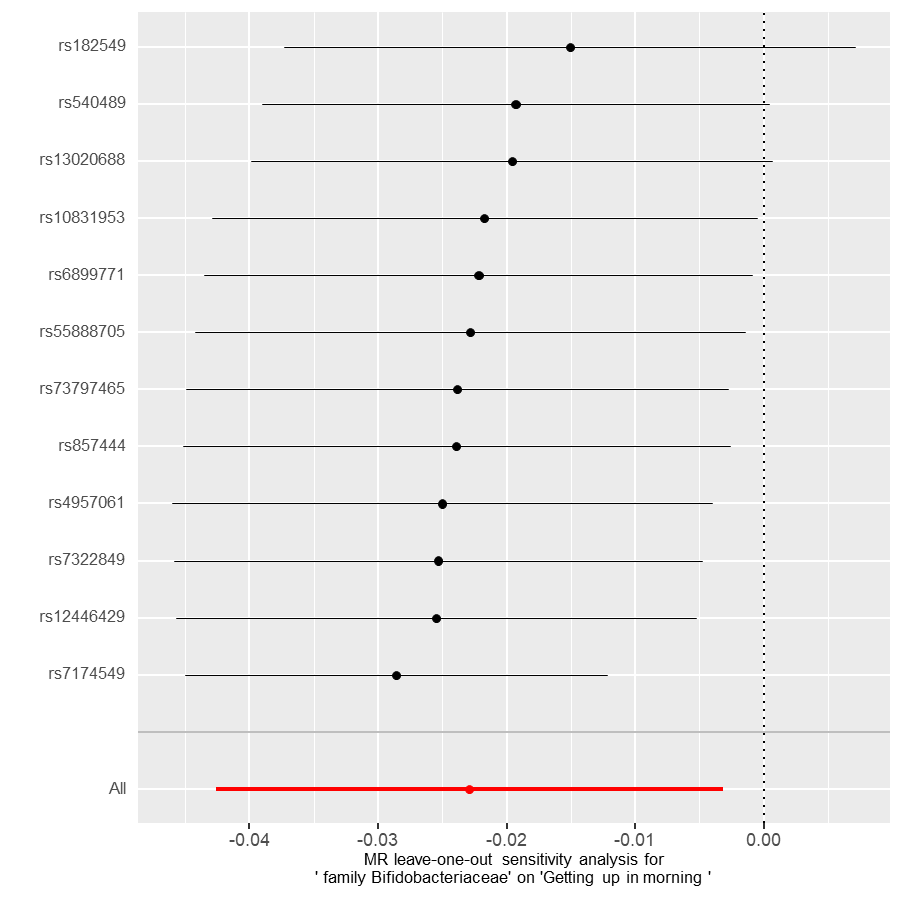

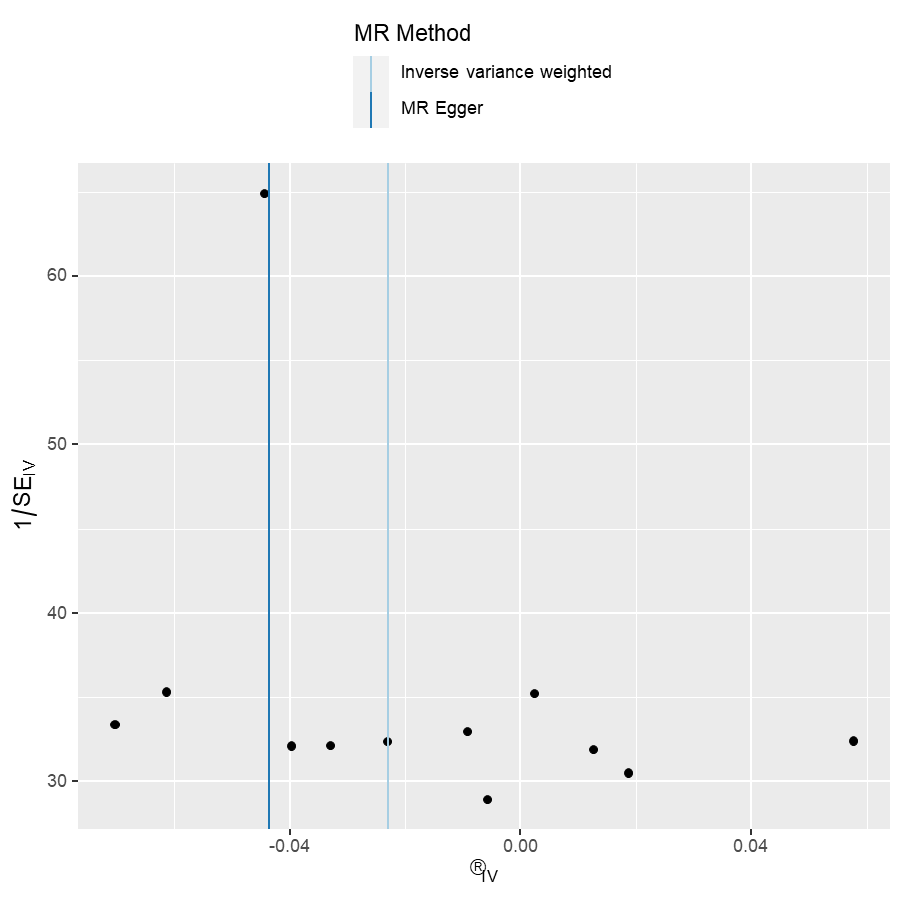
**

**Sup Fig. 74 Scatter plot,** **leave-one-out plot and funnel plot for the causal association between *order Clostridiales* and getting up in morning.**

**
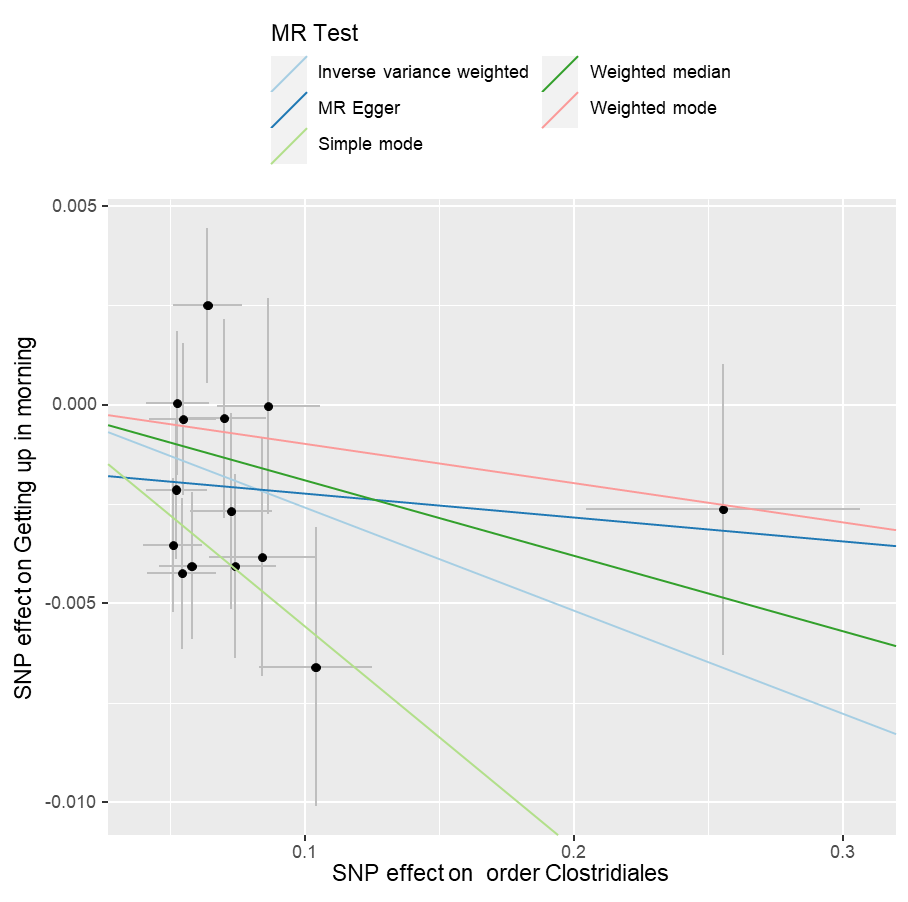

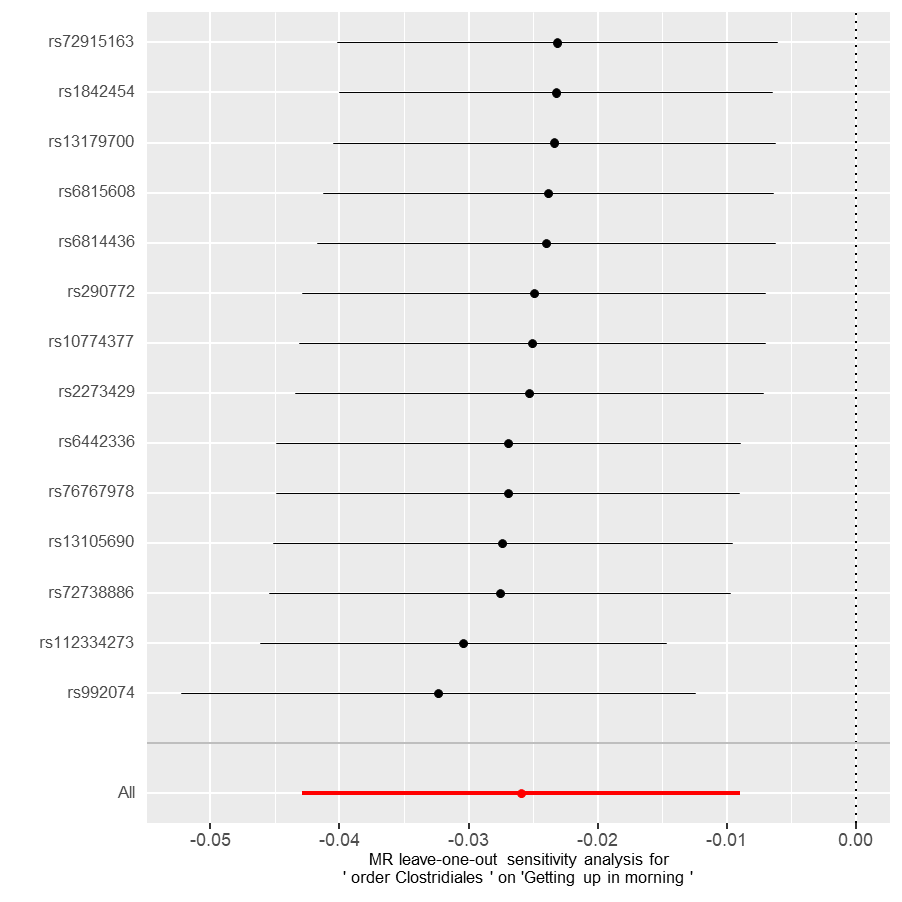

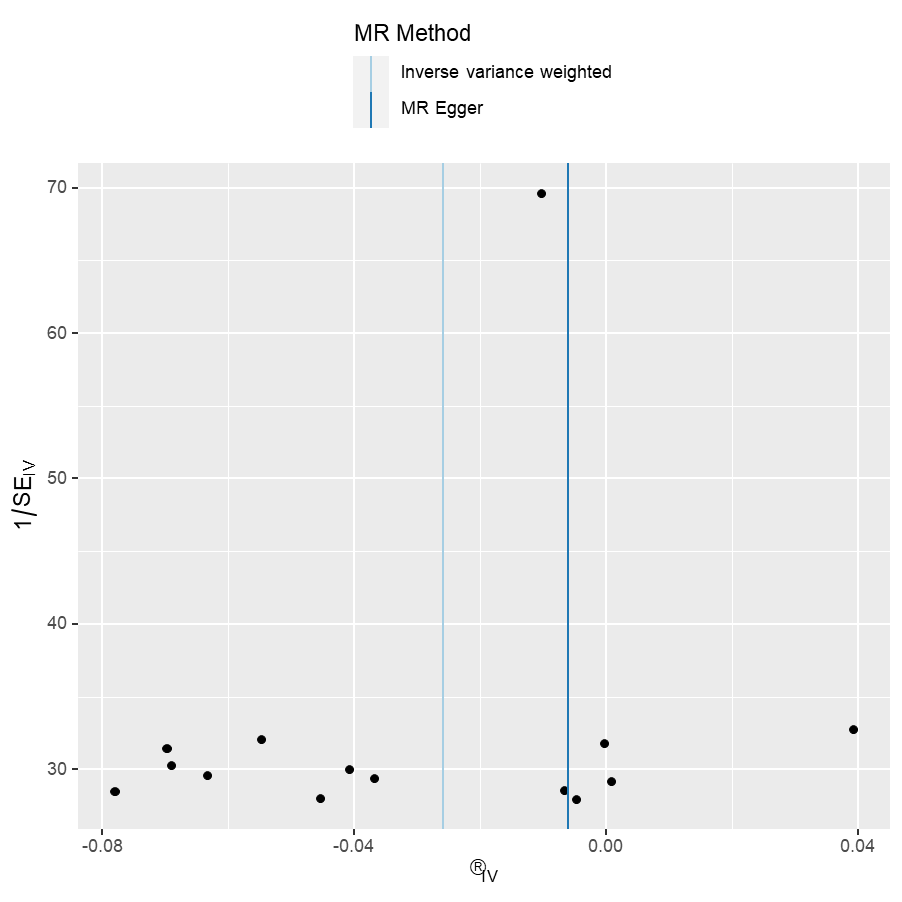
**

**Sup Fig. 75 Scatter plot,** **leave-one-out plot and funnel plot for the causal association between *class Negativicutes* and insomnia.**

**
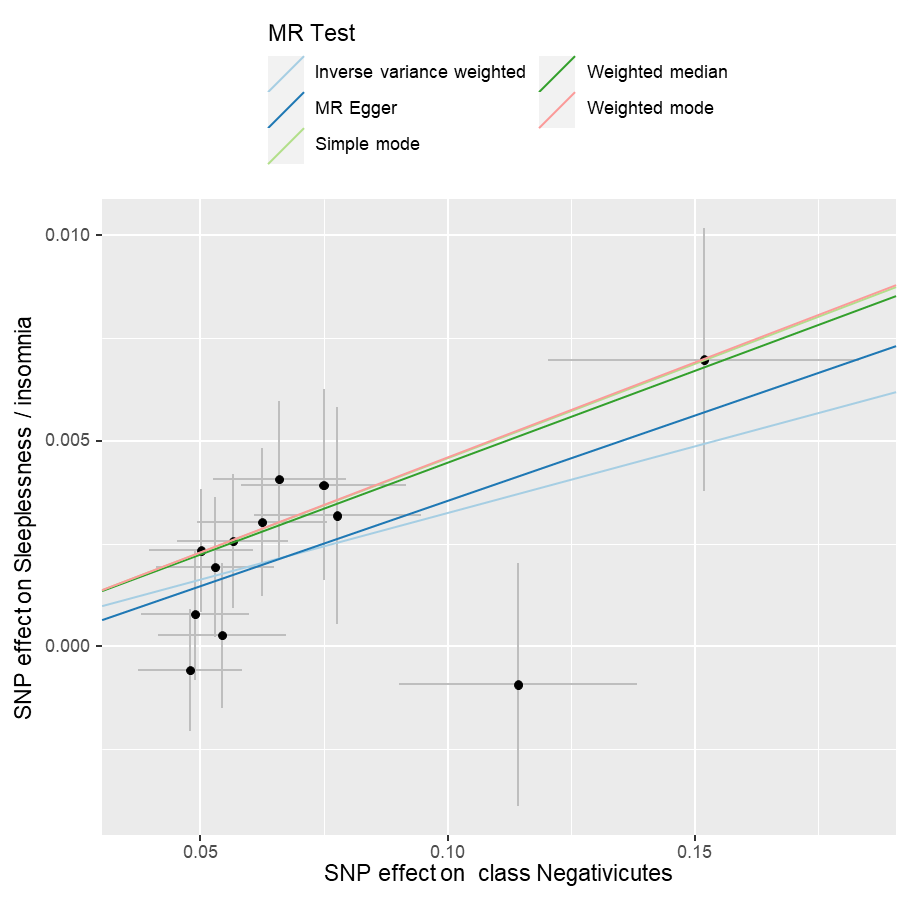

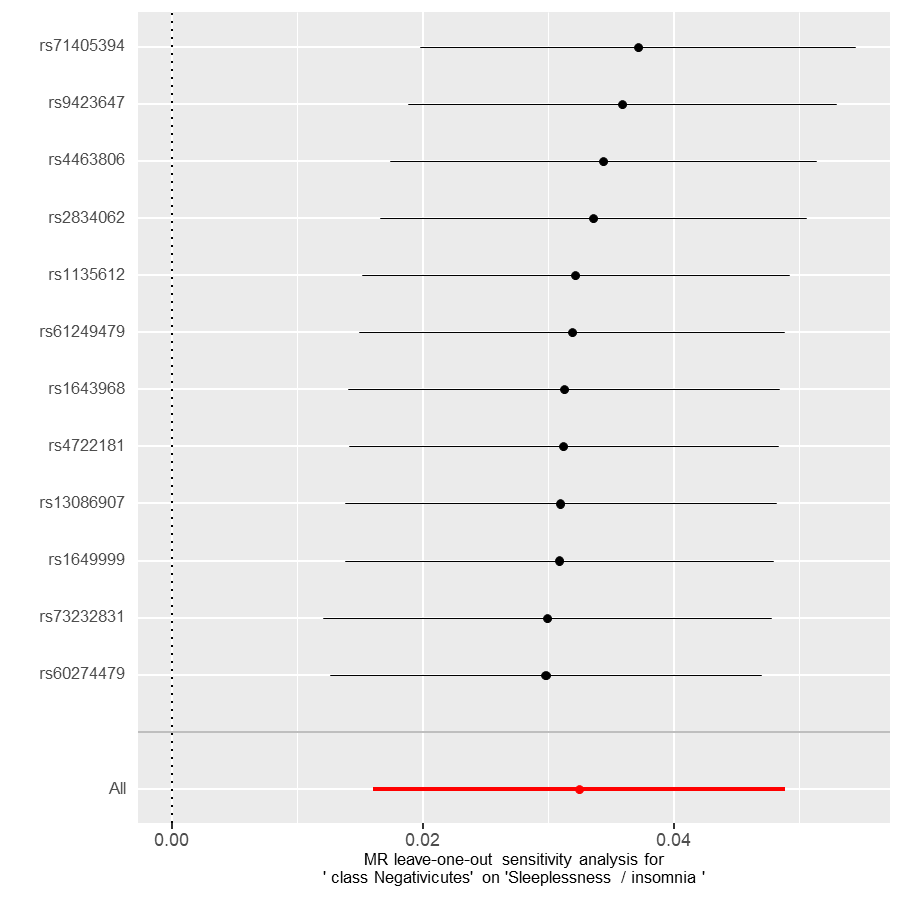

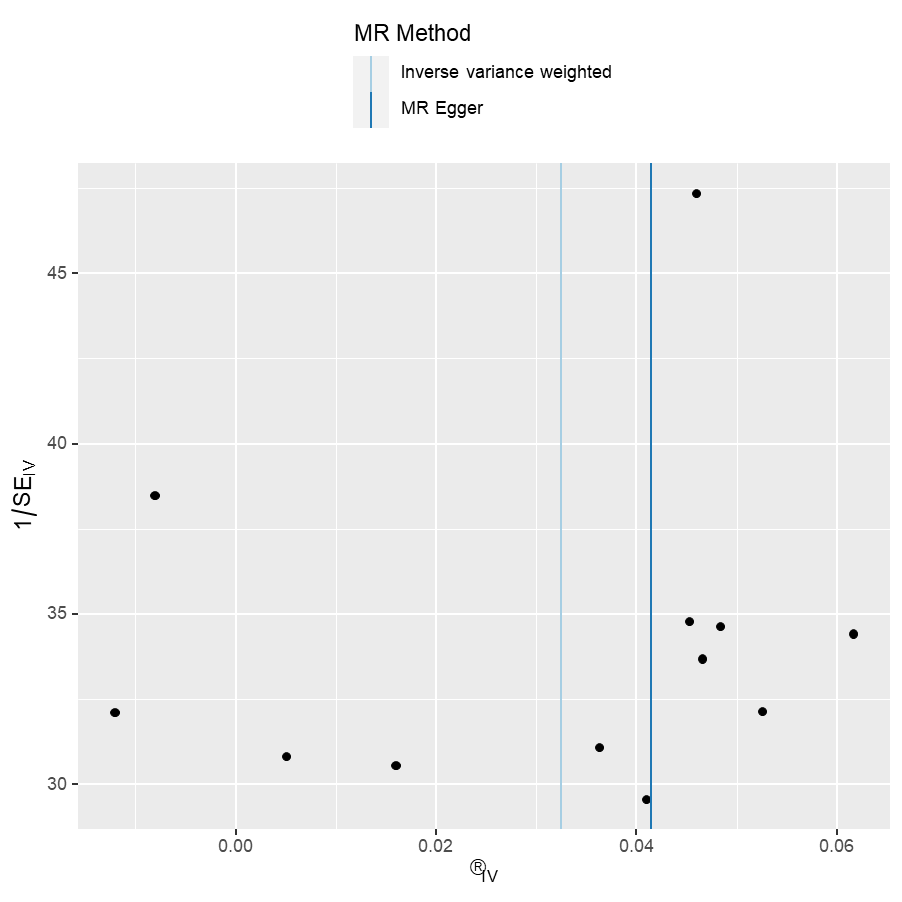
**

**Sup Fig. 76 Scatter plot,** **leave-one-out plot and funnel plot for the causal association between *genus Eubacterium eligens group* and insomnia.**

**
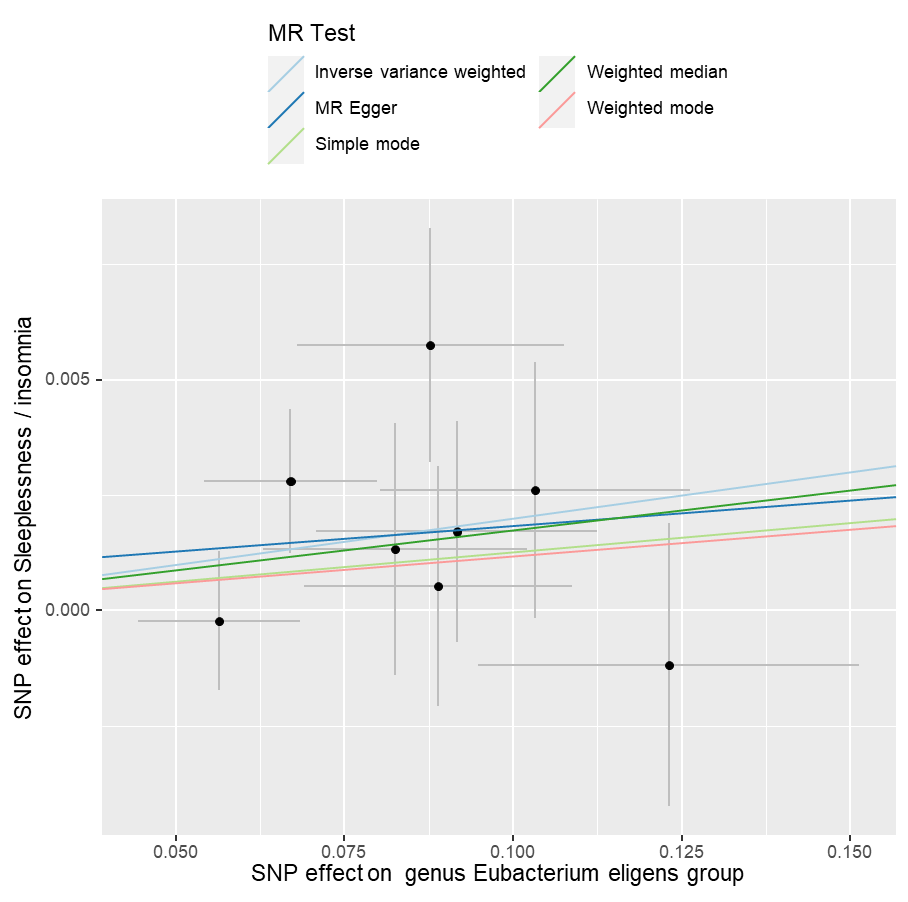

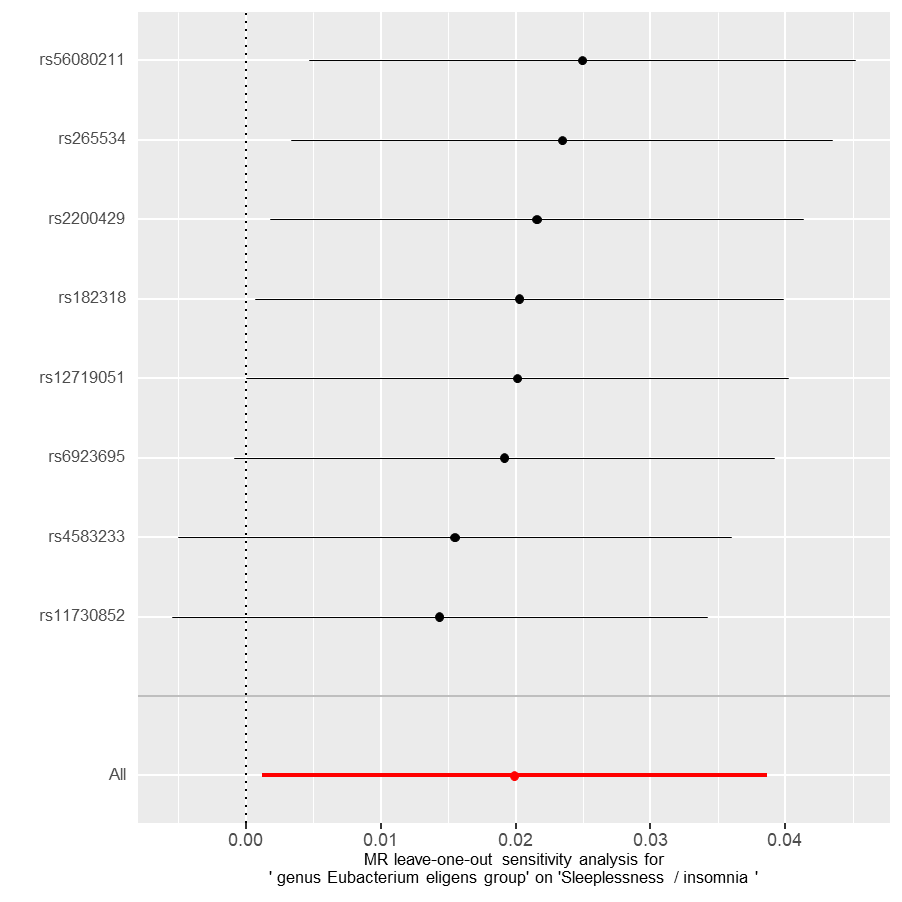

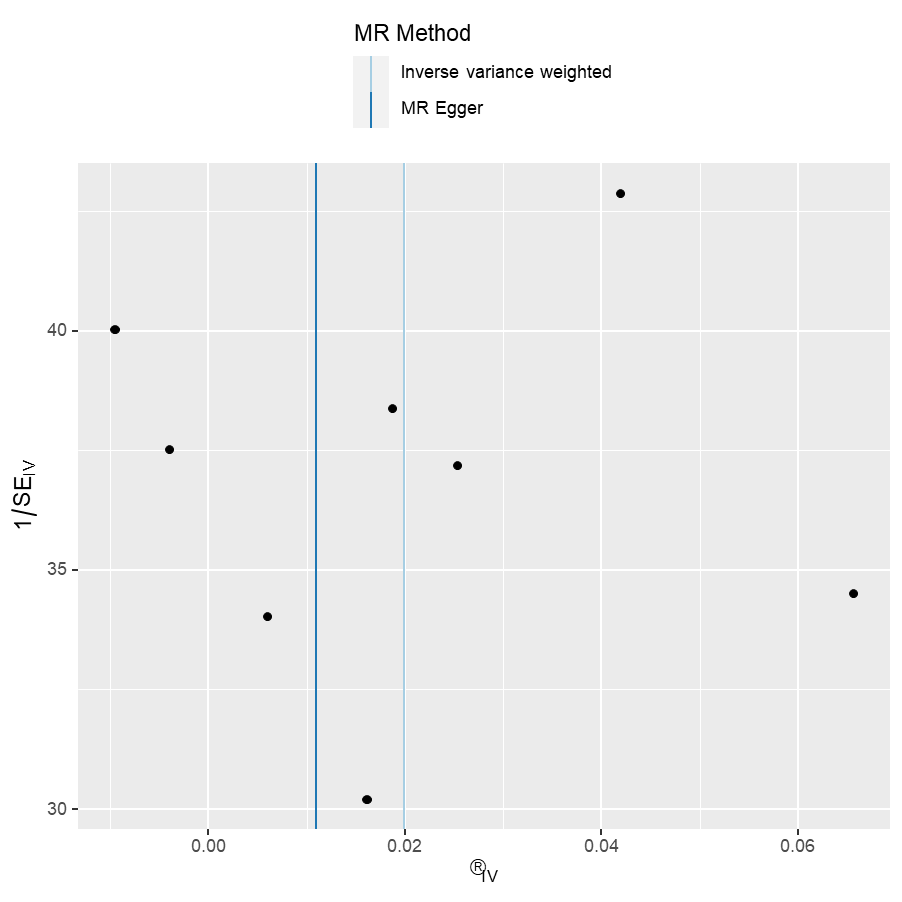
**

**Sup Fig. 77 Scatter plot,** **leave-one-out plot and funnel plot for the causal association between *genus Oxalobacter* and insomnia.**

**
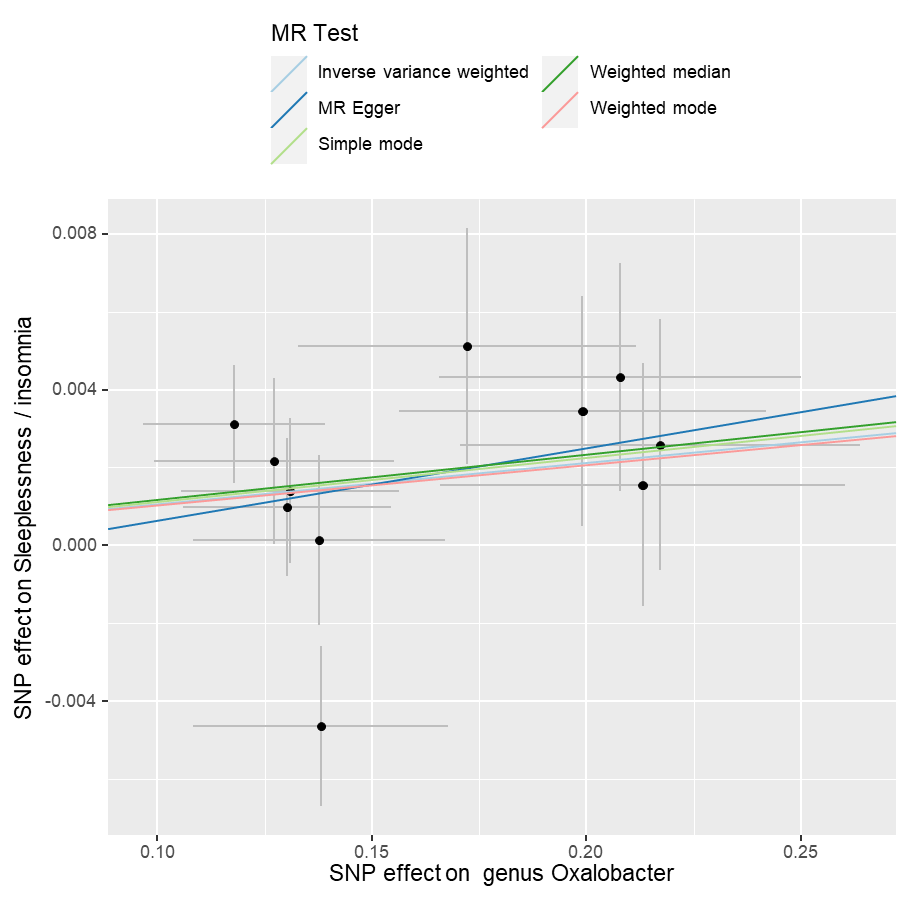

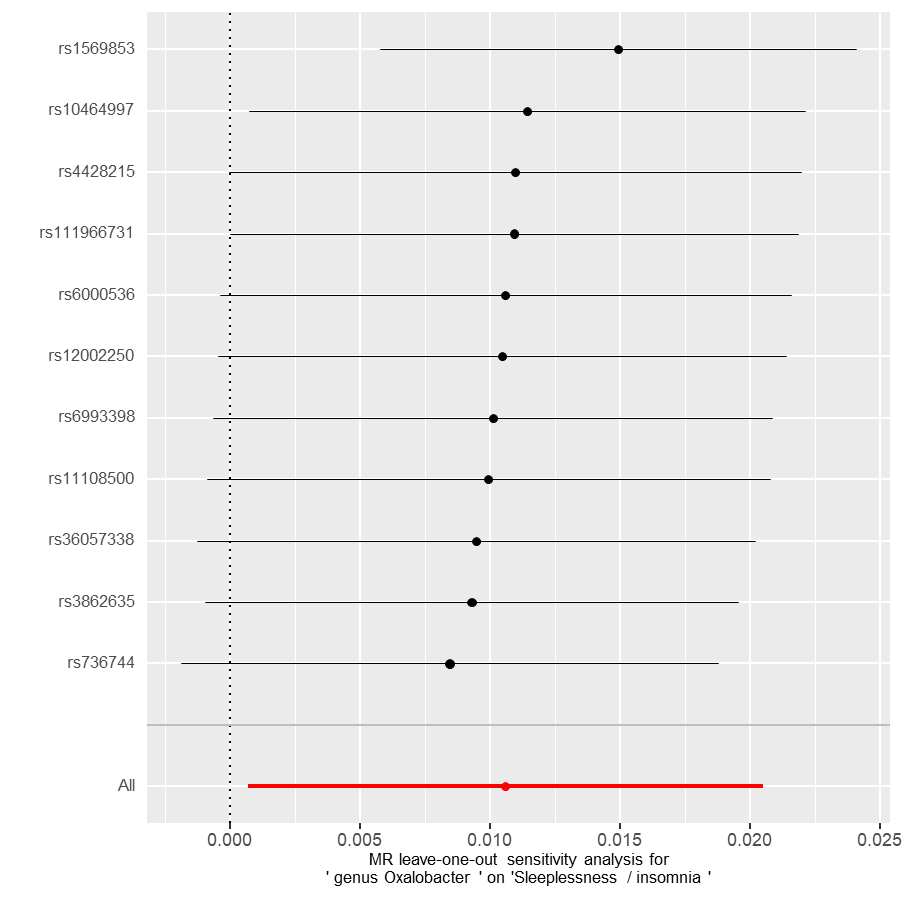

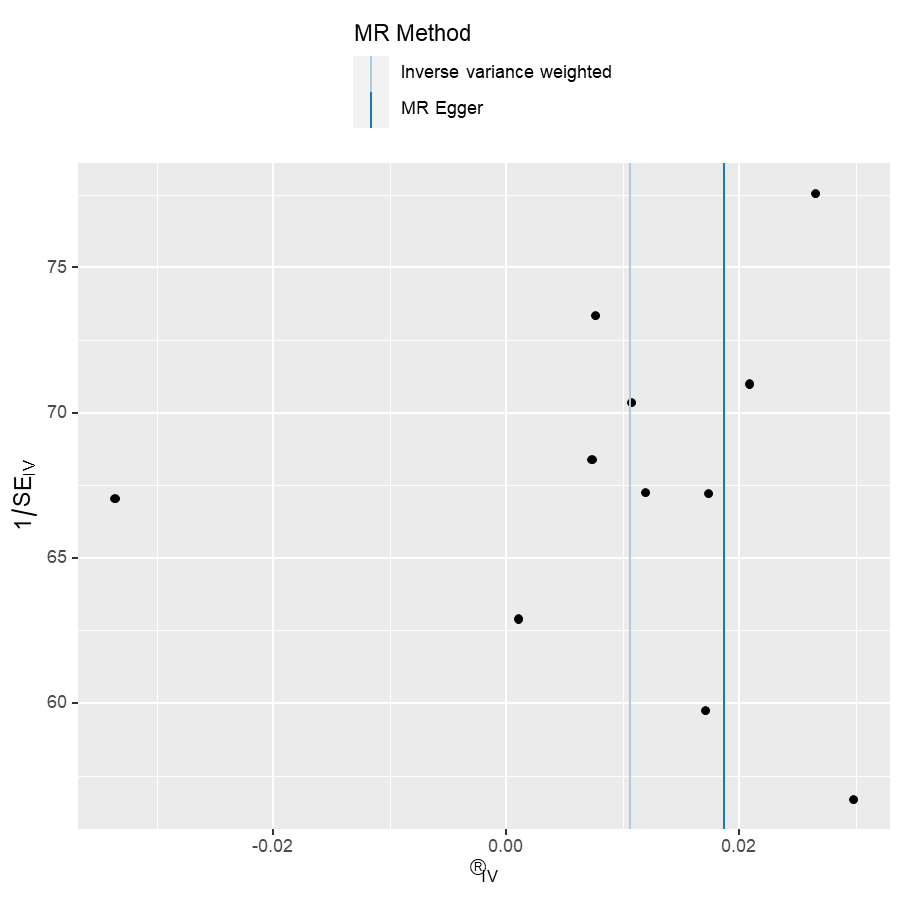
**

**Sup Fig. 78 Scatter plot,** **leave-one-out plot and funnel plot for the causal association between *order Selenomonadales* and insomnia.**

**
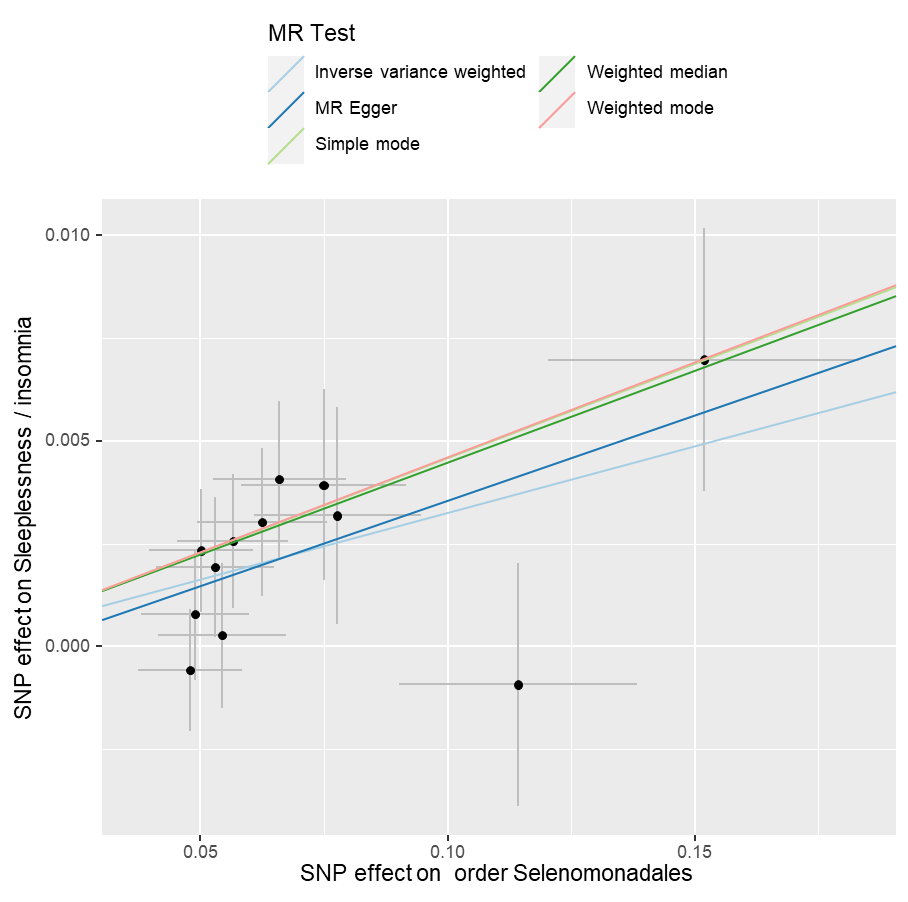

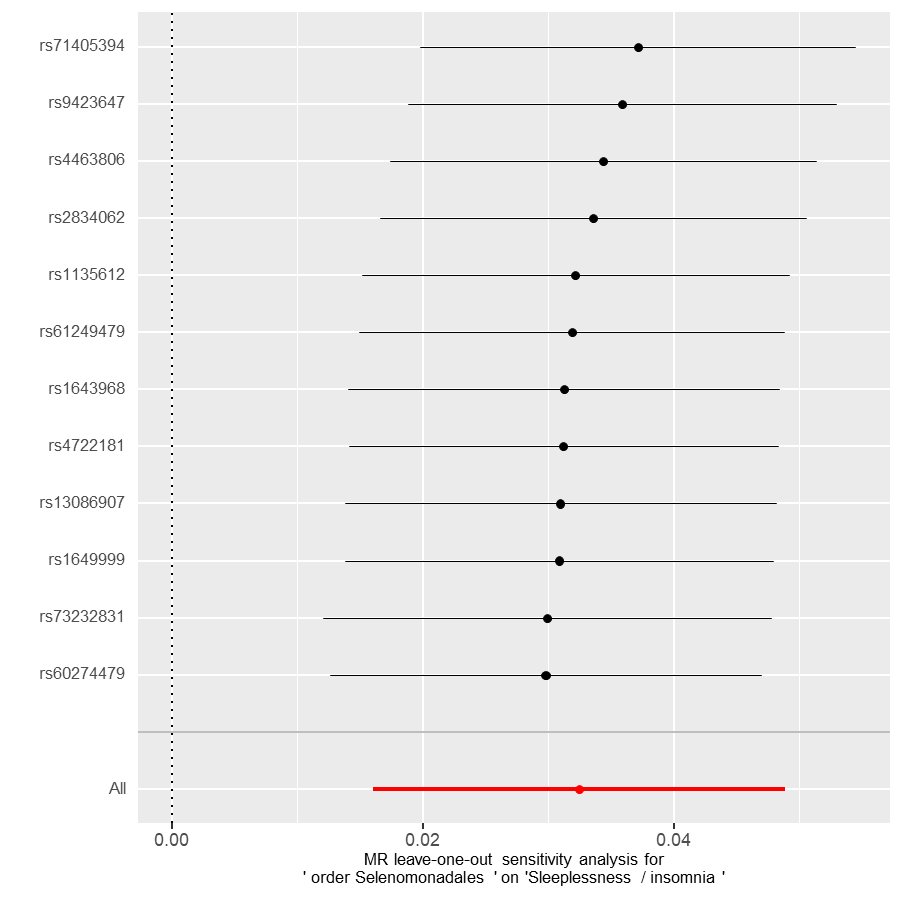

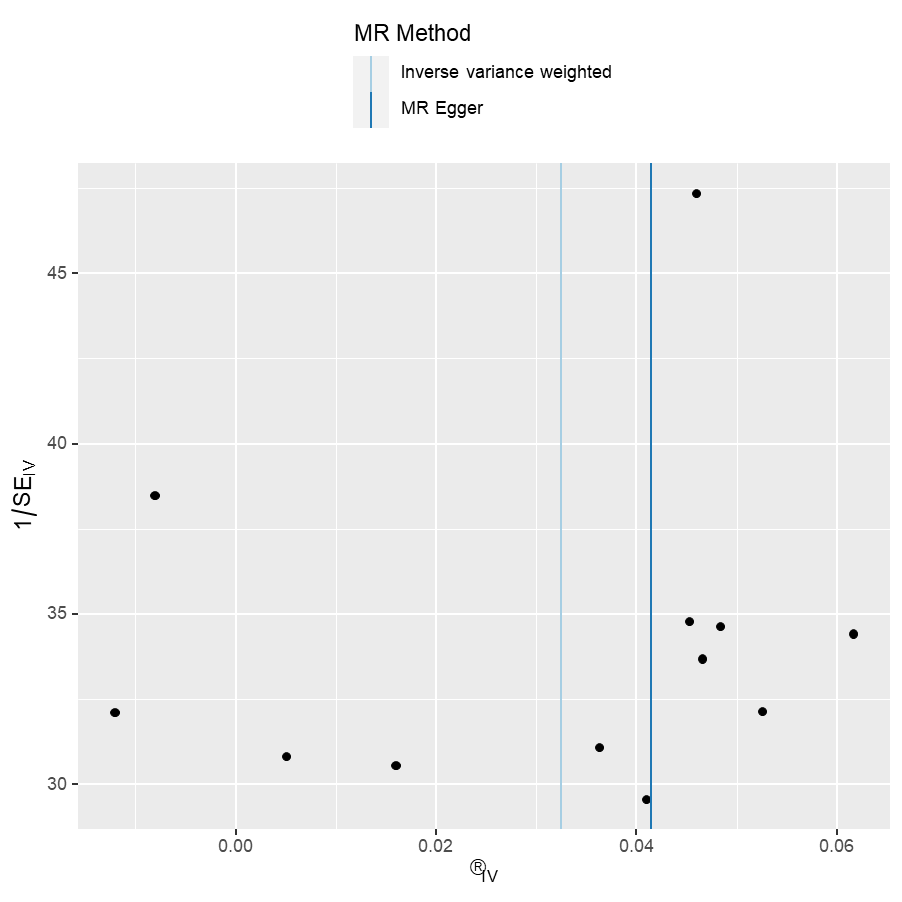
**

**Sup Fig. 79 Scatter plot,** **leave-one-out plot and funnel plot for the causal association between *phylum Firmicutes* and insomnia.**

**
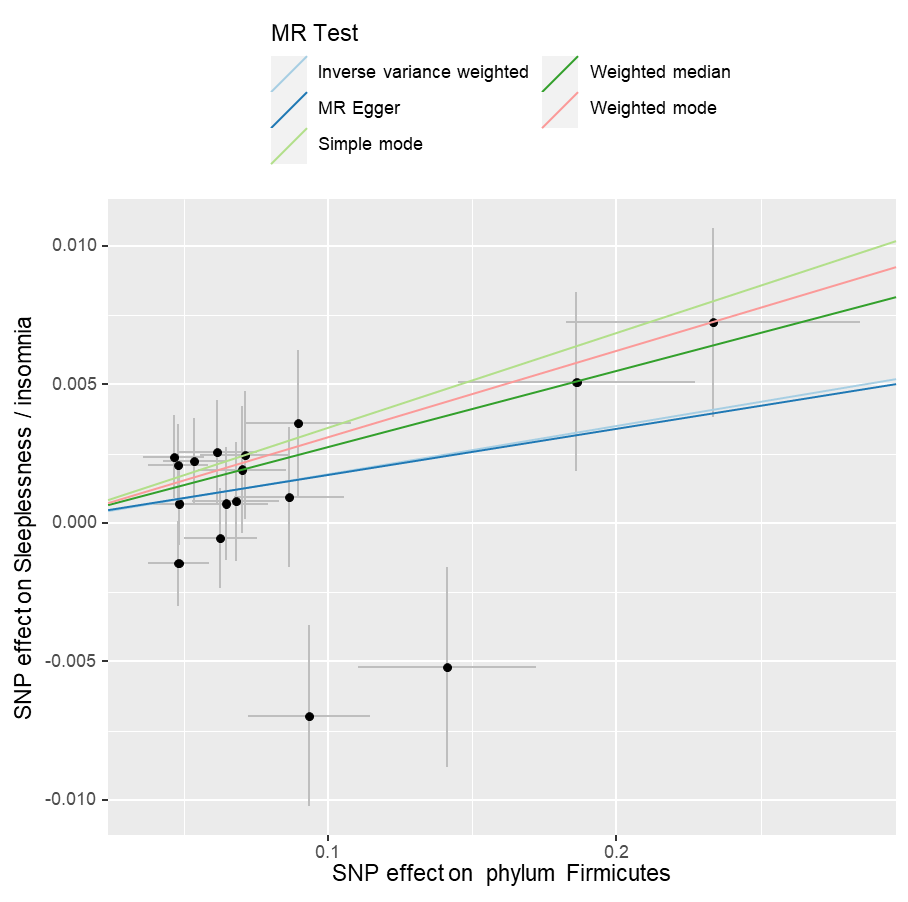

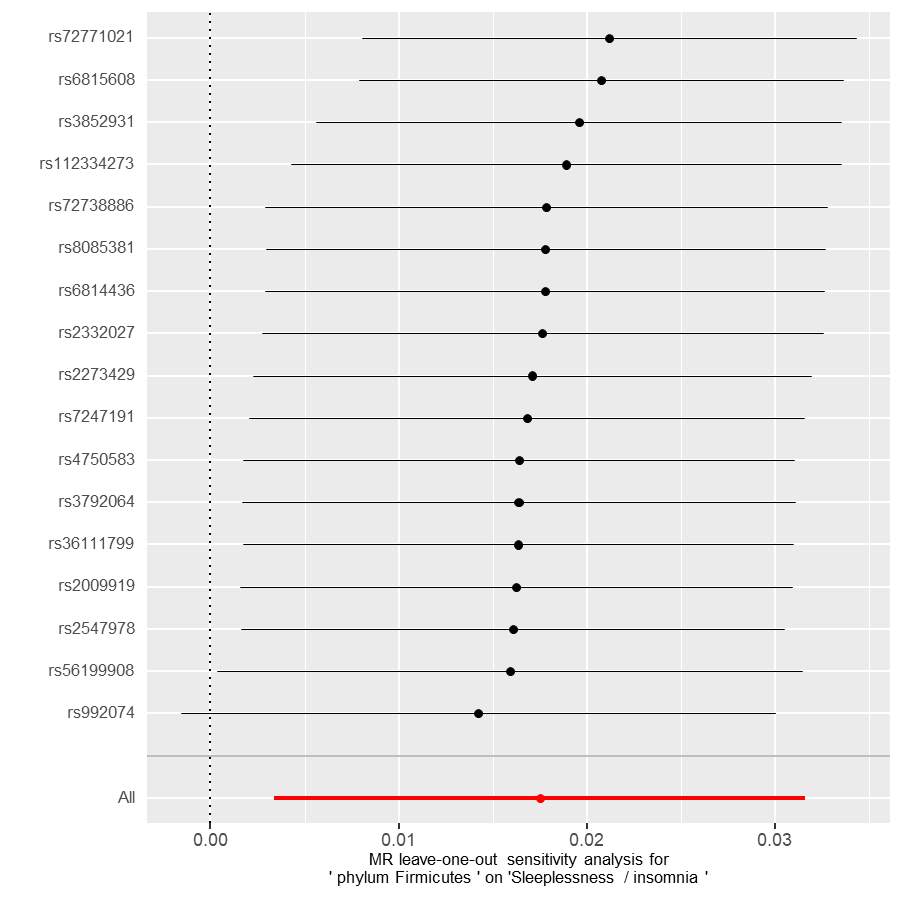

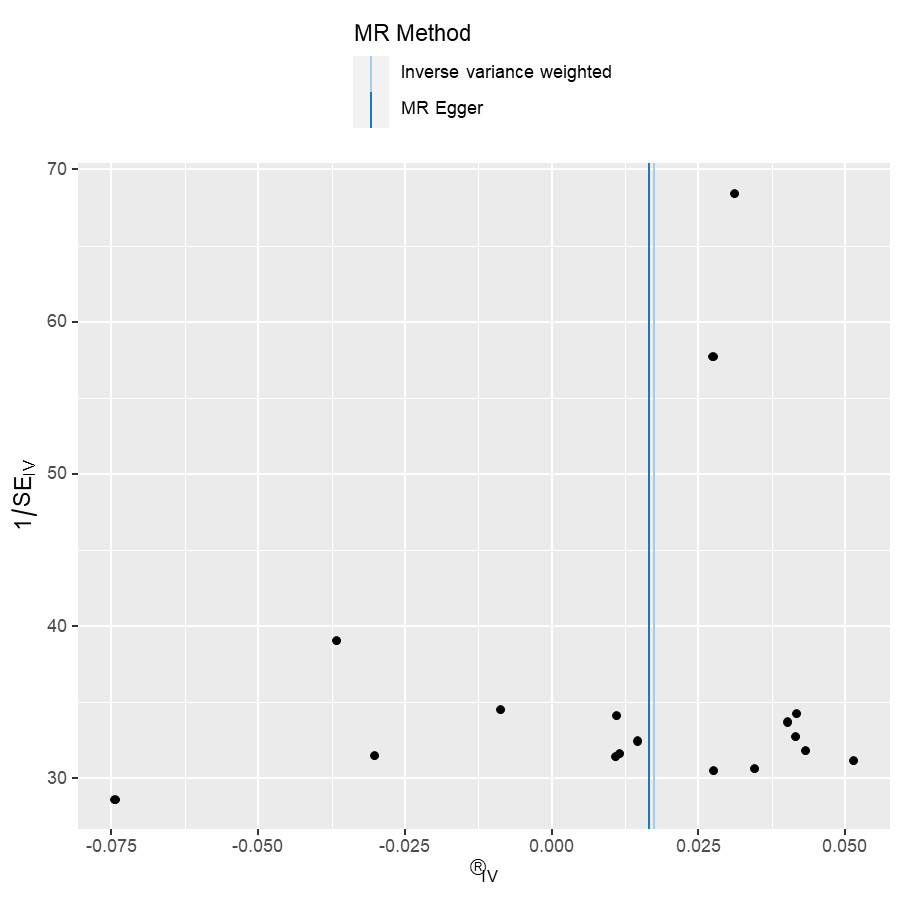
**

**Sup Fig. 80 Scatter plot,** **leave-one-out plot and funnel plot for the causal association between *phylum Verrucomicrobia* and insomnia.**

**
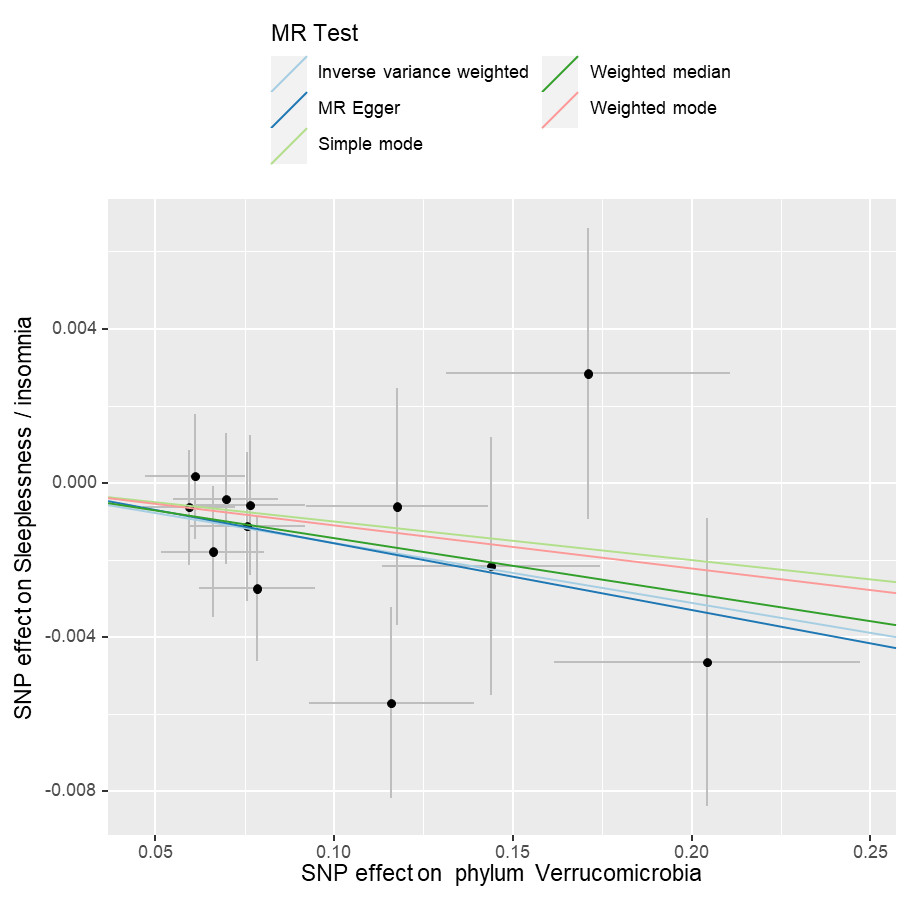

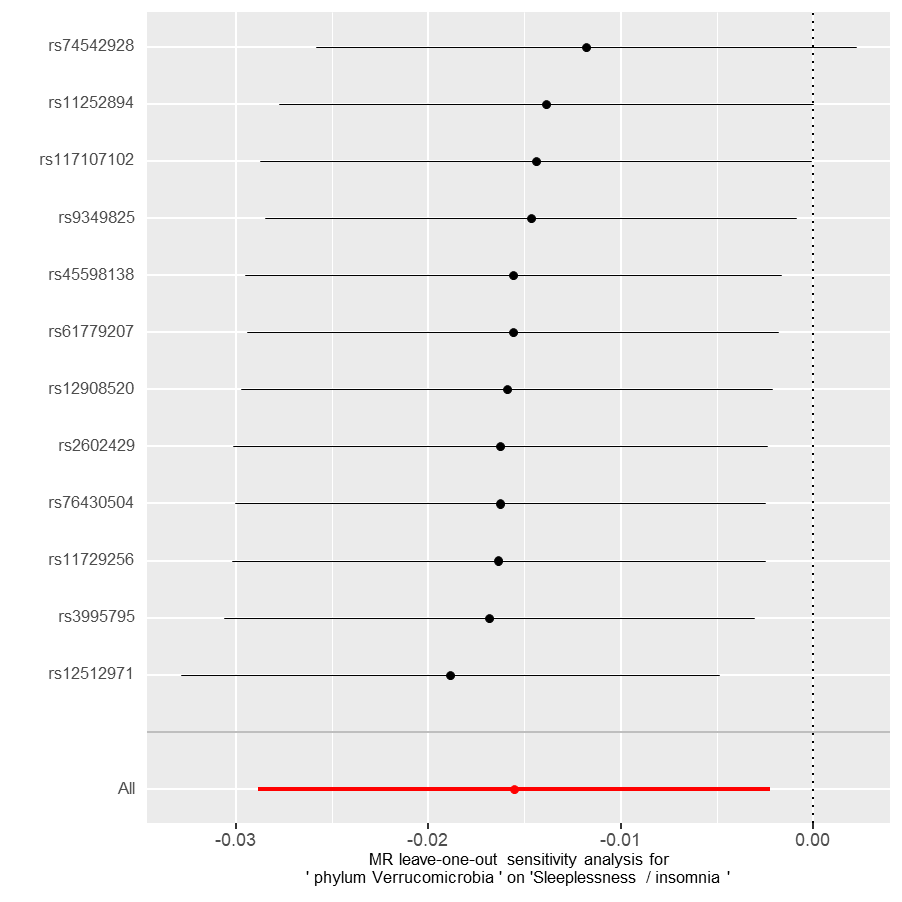

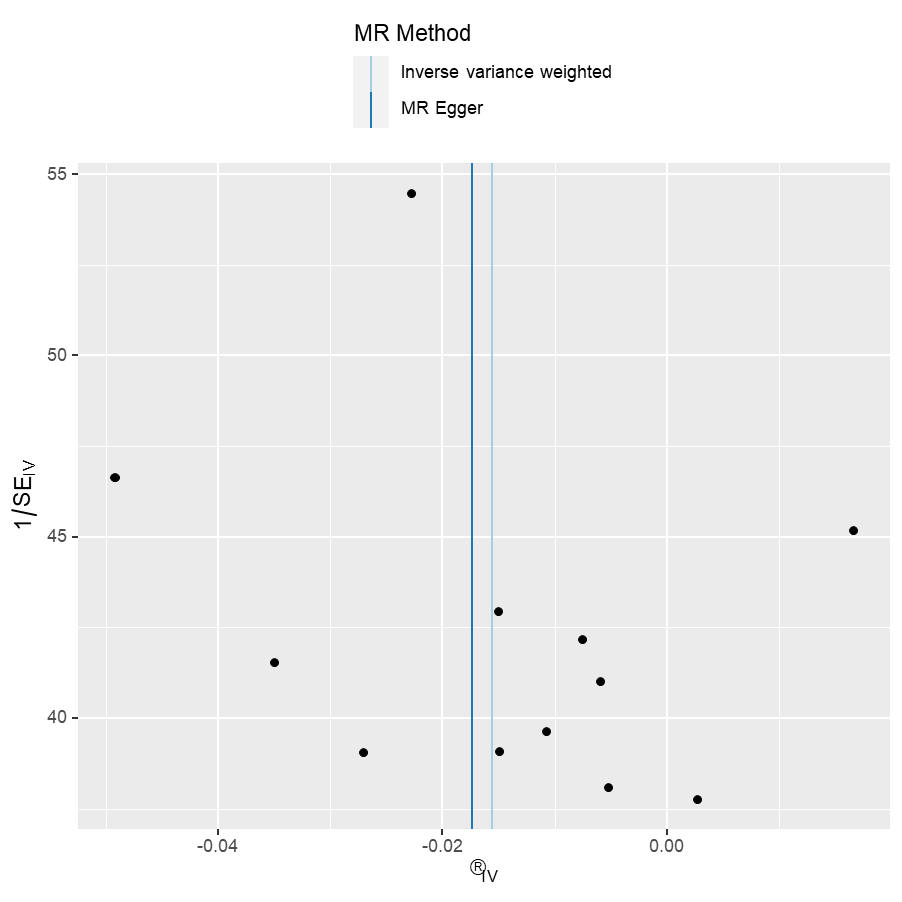
**

**Sup Fig. 81 Scatter plot,** **leave-one-out plot and funnel plot for the causal association between *family Peptococcaceae* and chronotype.**

**
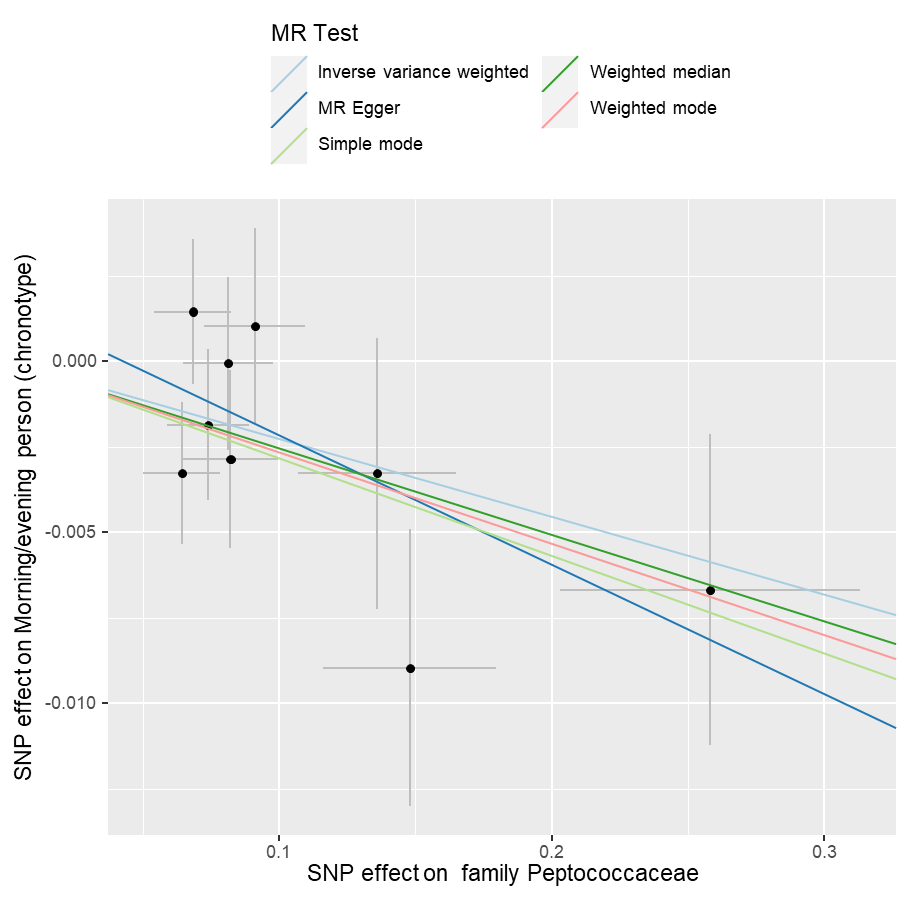

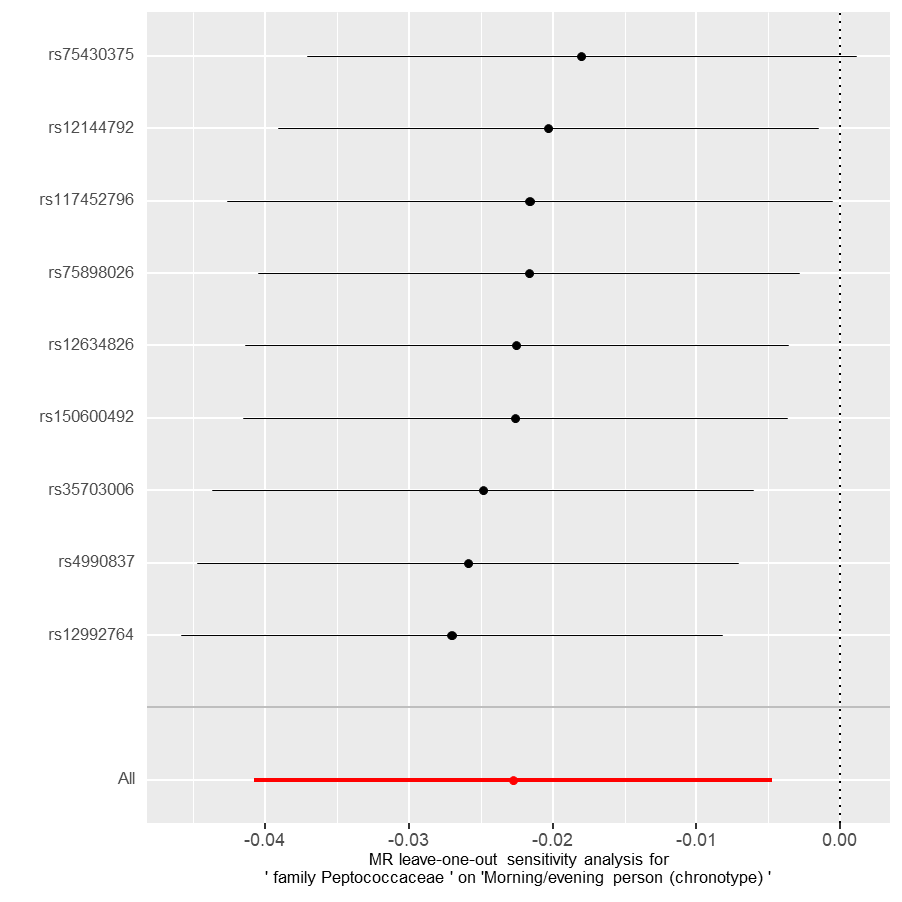

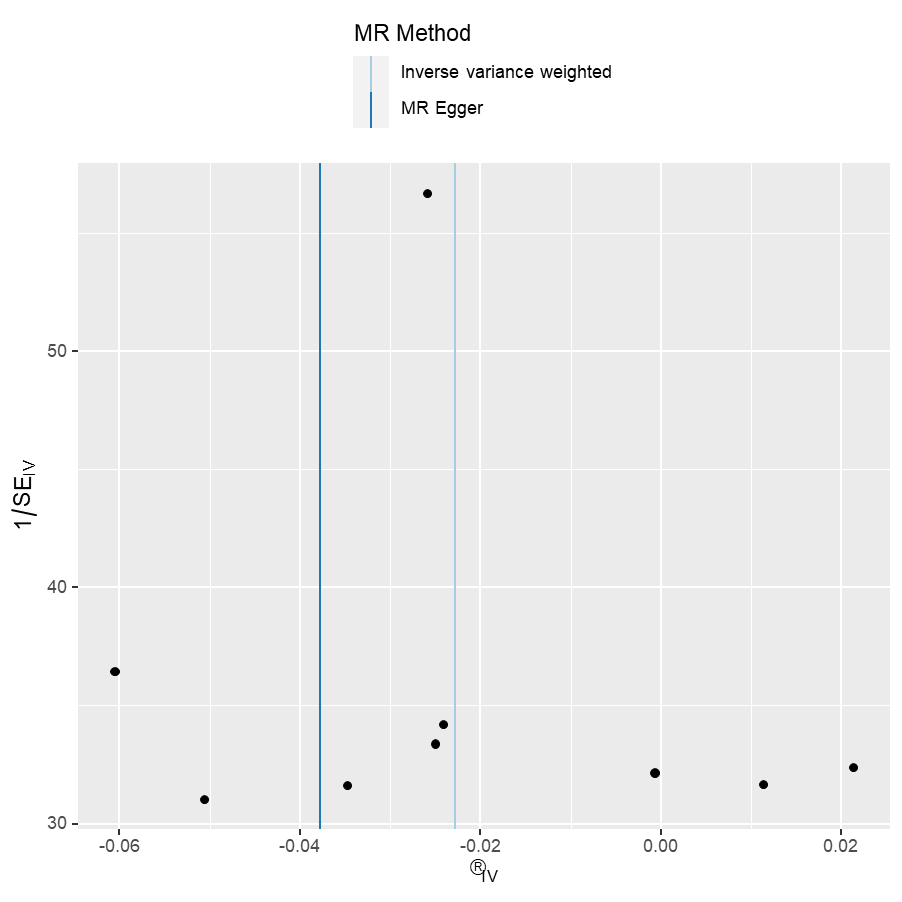
**

**Sup Fig. 82 Scatter plot,** **leave-one-out plot and funnel plot for the causal association between *genus Parabacteroides* and chronotype.**

**
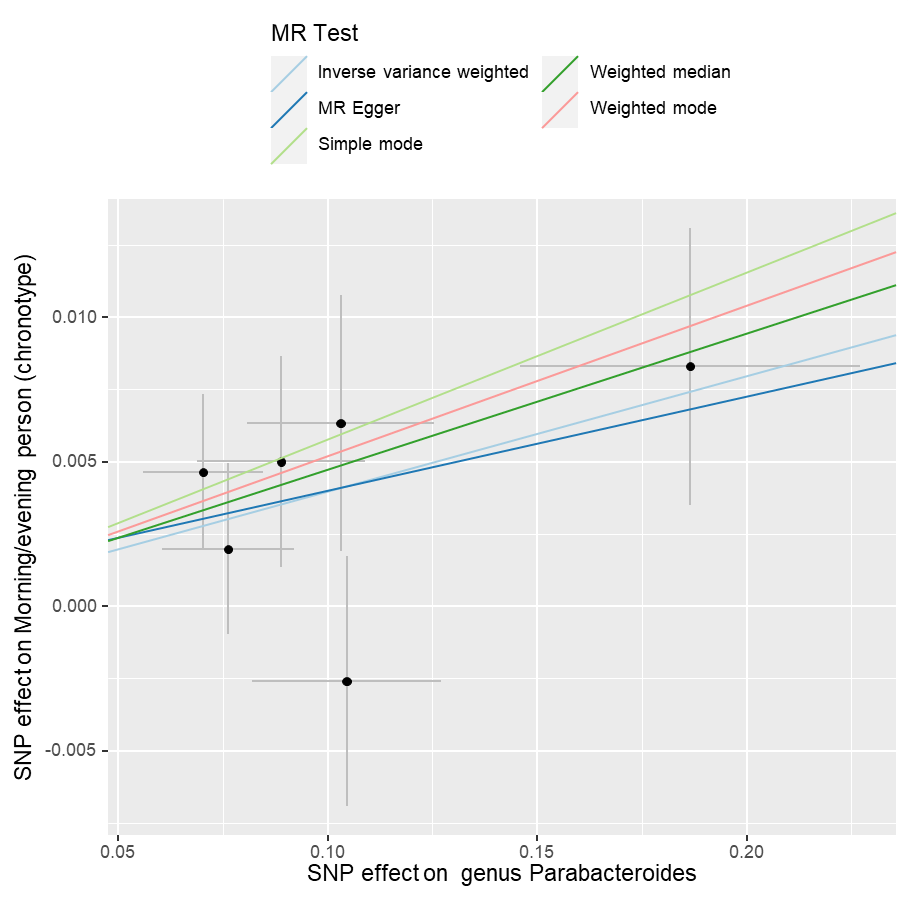

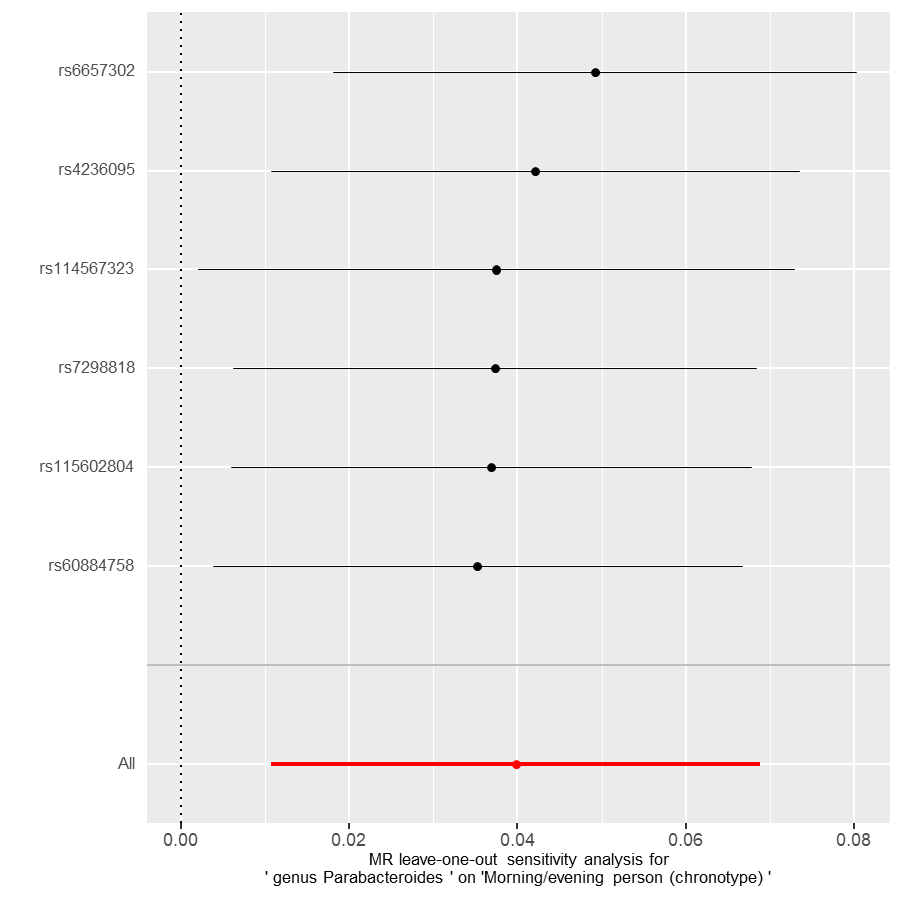

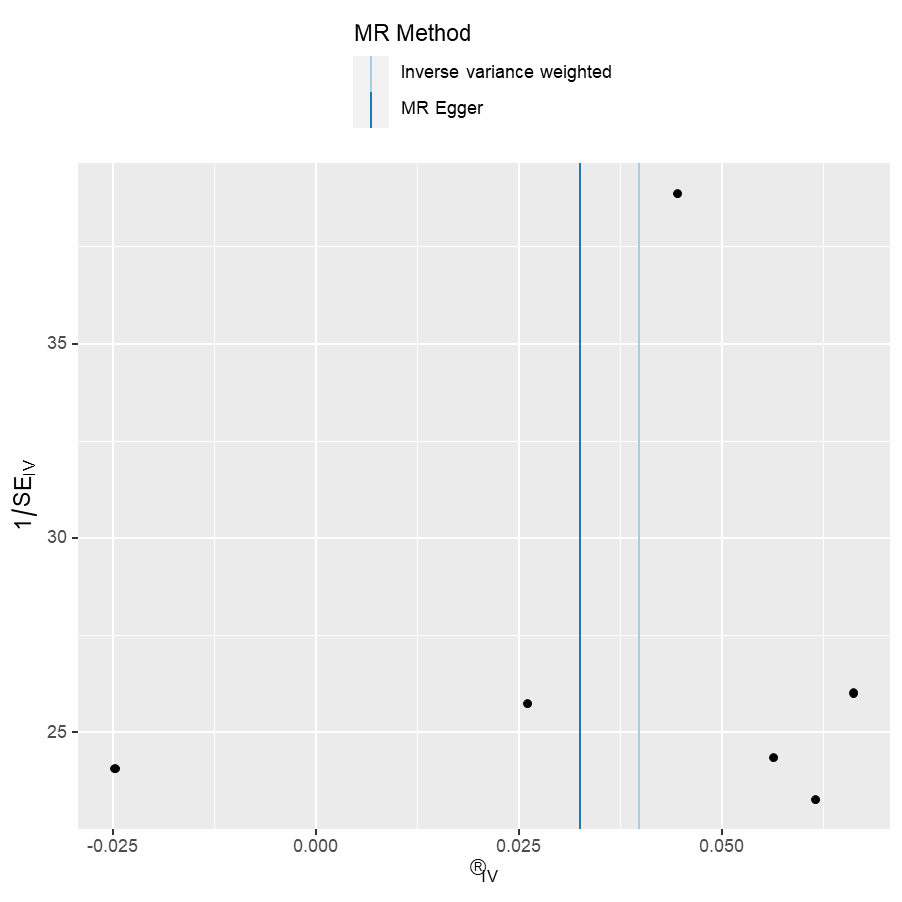
**

**Sup Fig. 83 Scatter plot,** **leave-one-out plot and funnel plot for the causal association between *genus Eubacterium coprostanoligenes group* and chronotype.**

**
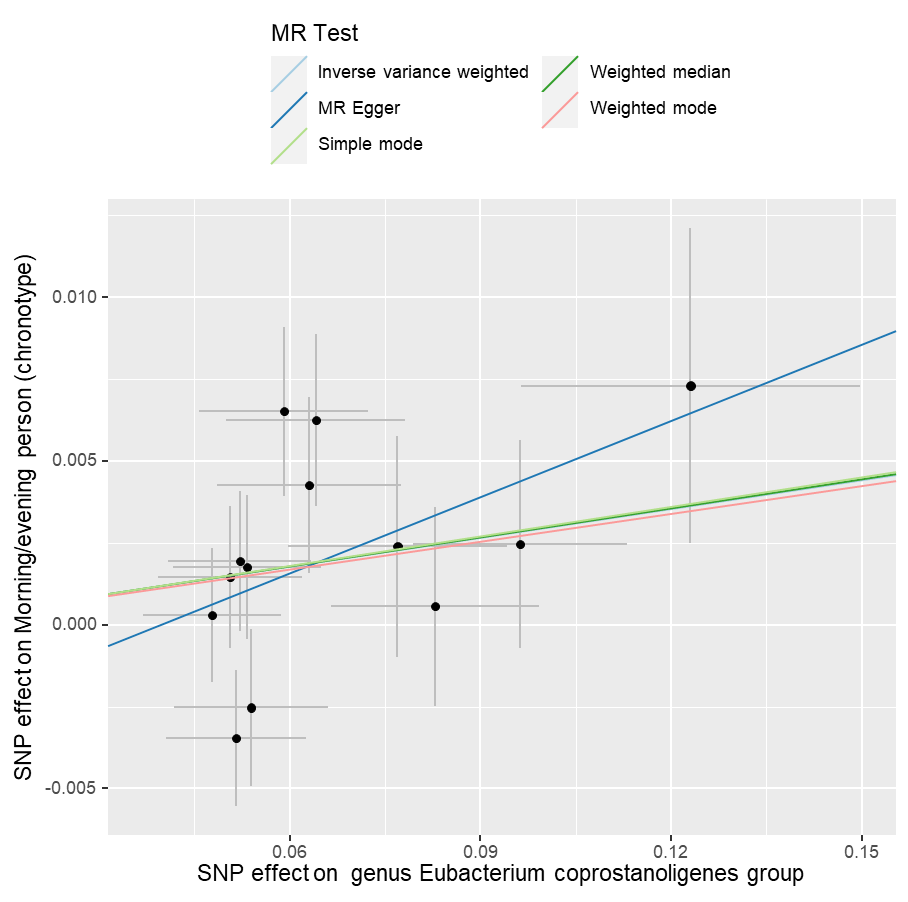

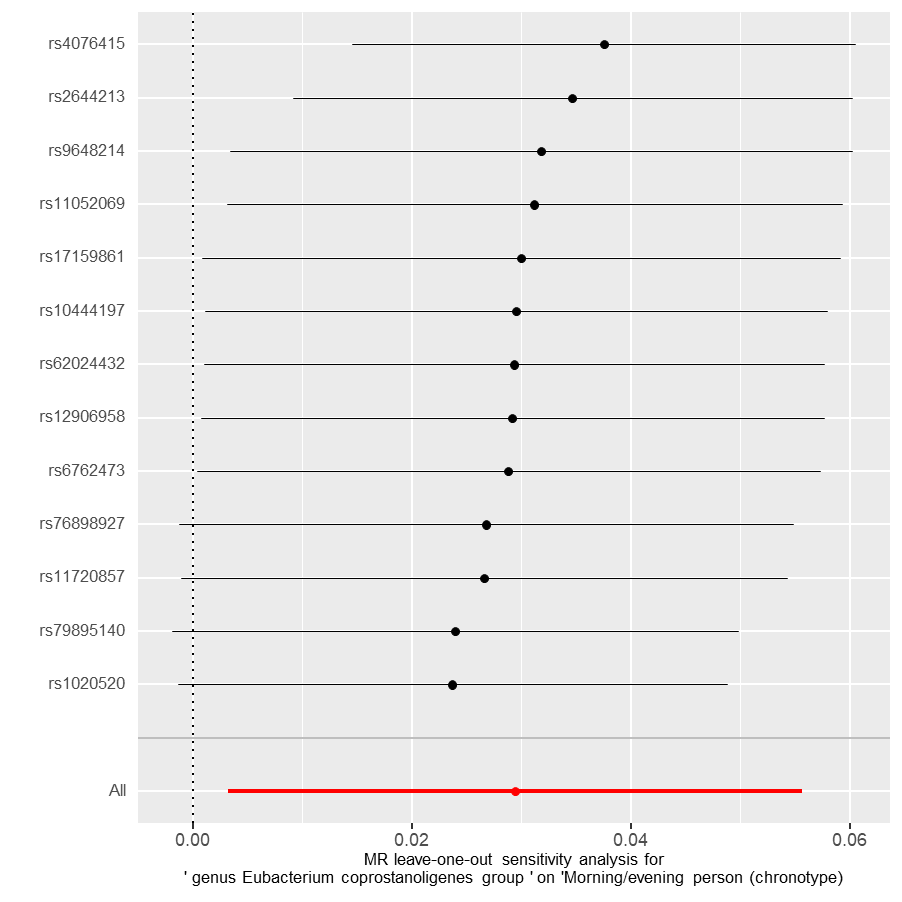

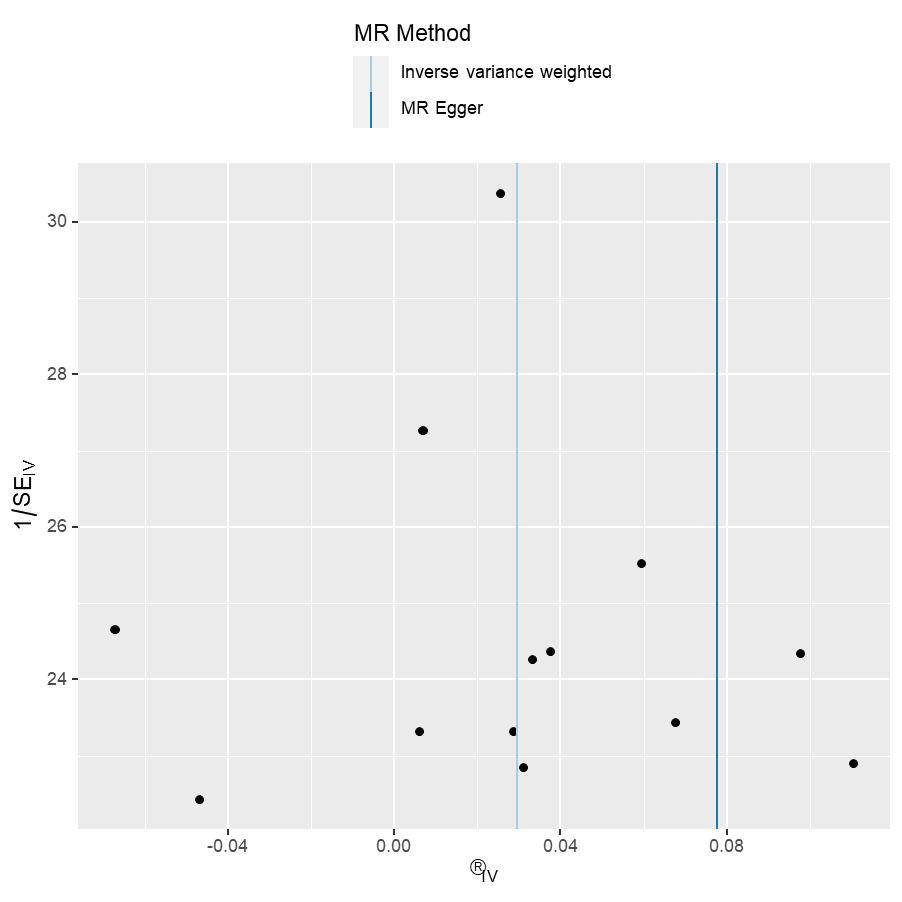
**

**Sup Fig. 84 Scatter plot,** **leave-one-out plot and funnel plot for the causal association between *genus Prevotella7* and chronotype.**

**
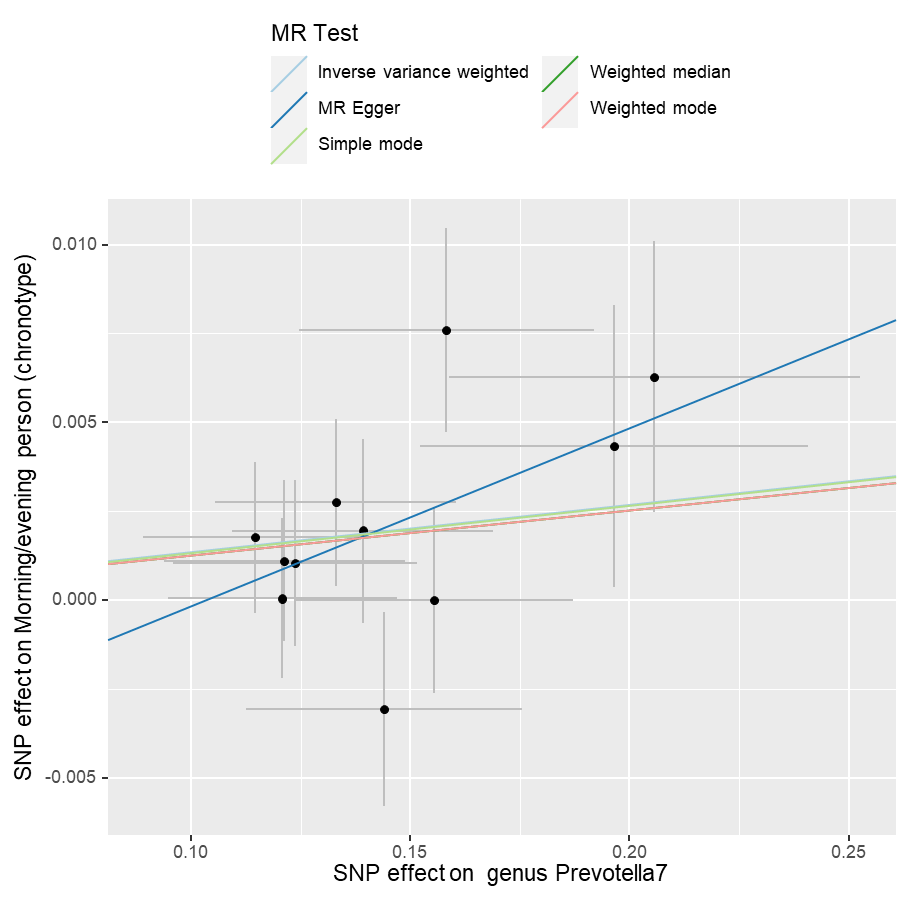

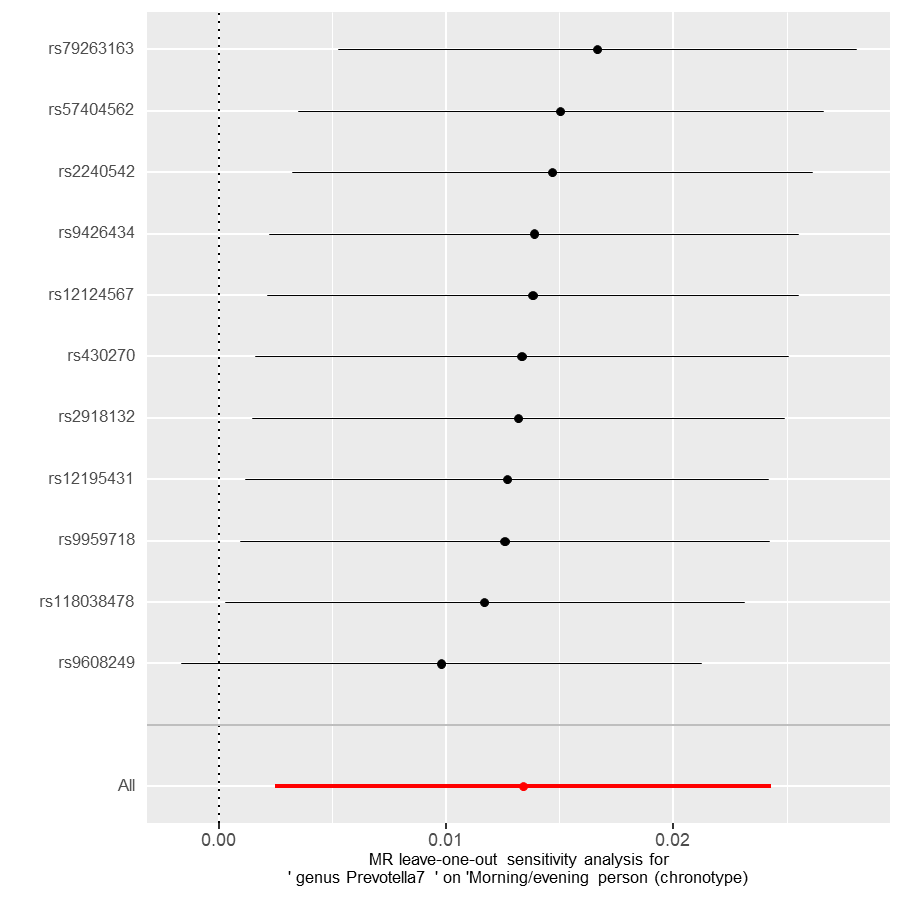

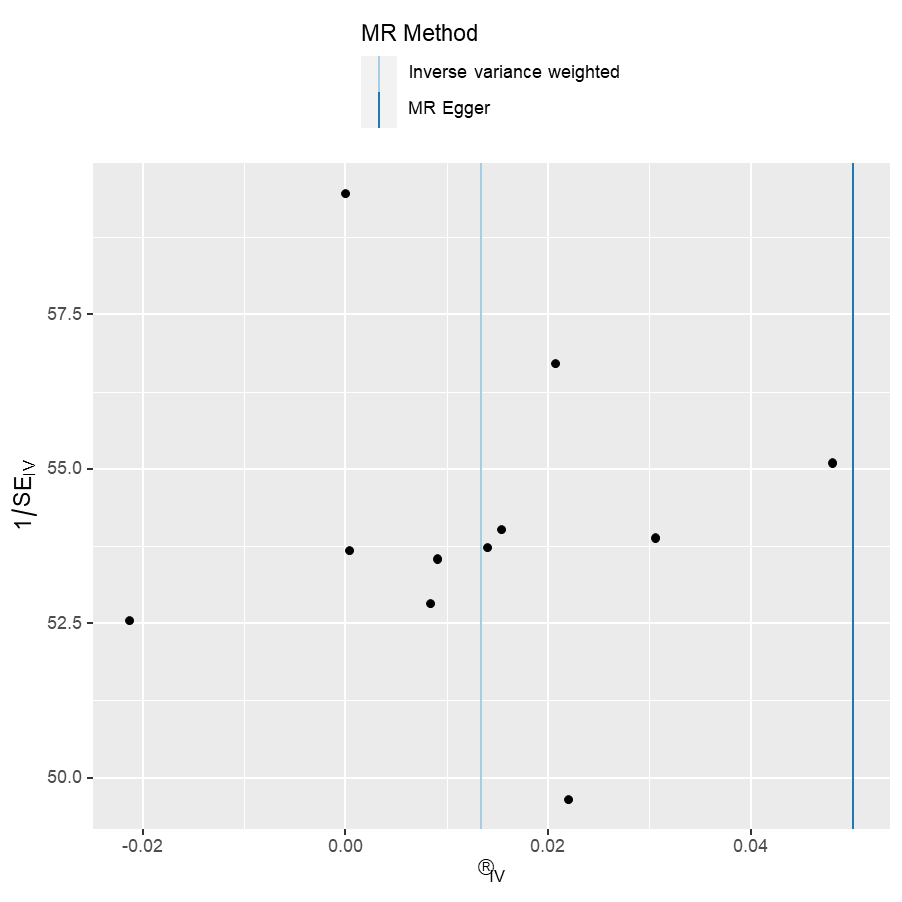
**

**Sup Fig. 85 Scatter plot,** **leave-one-out plot and funnel plot for the causal association between *genus Ruminococcus1* and chronotype.**

**
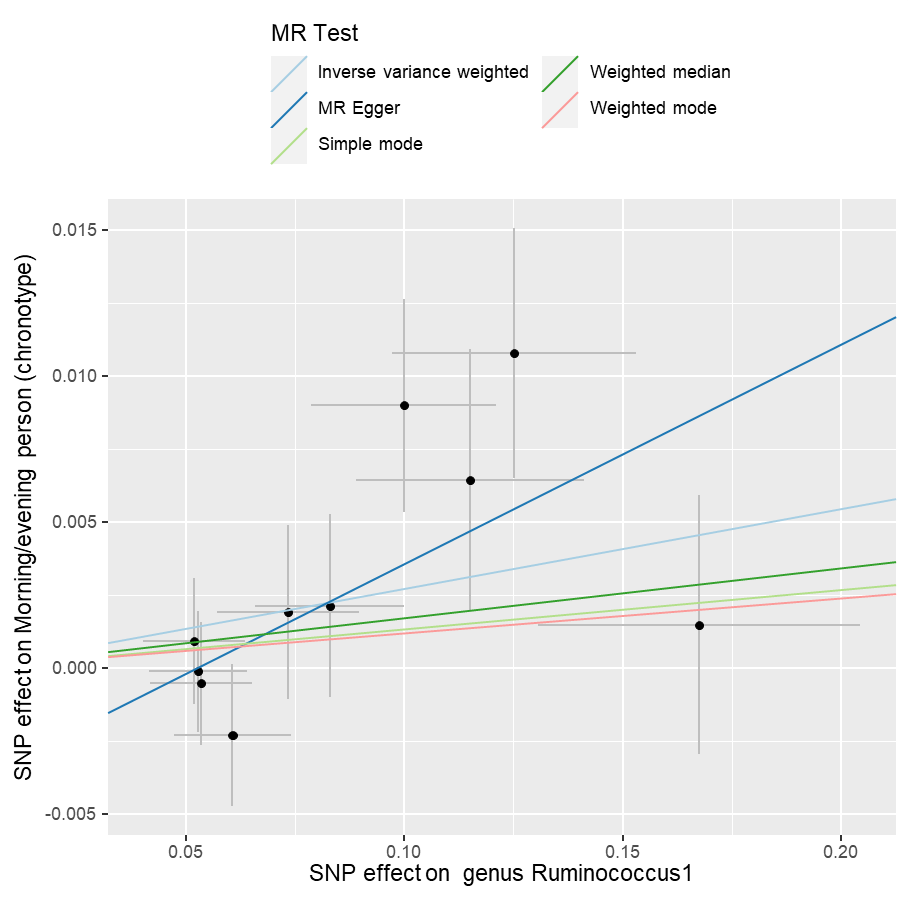

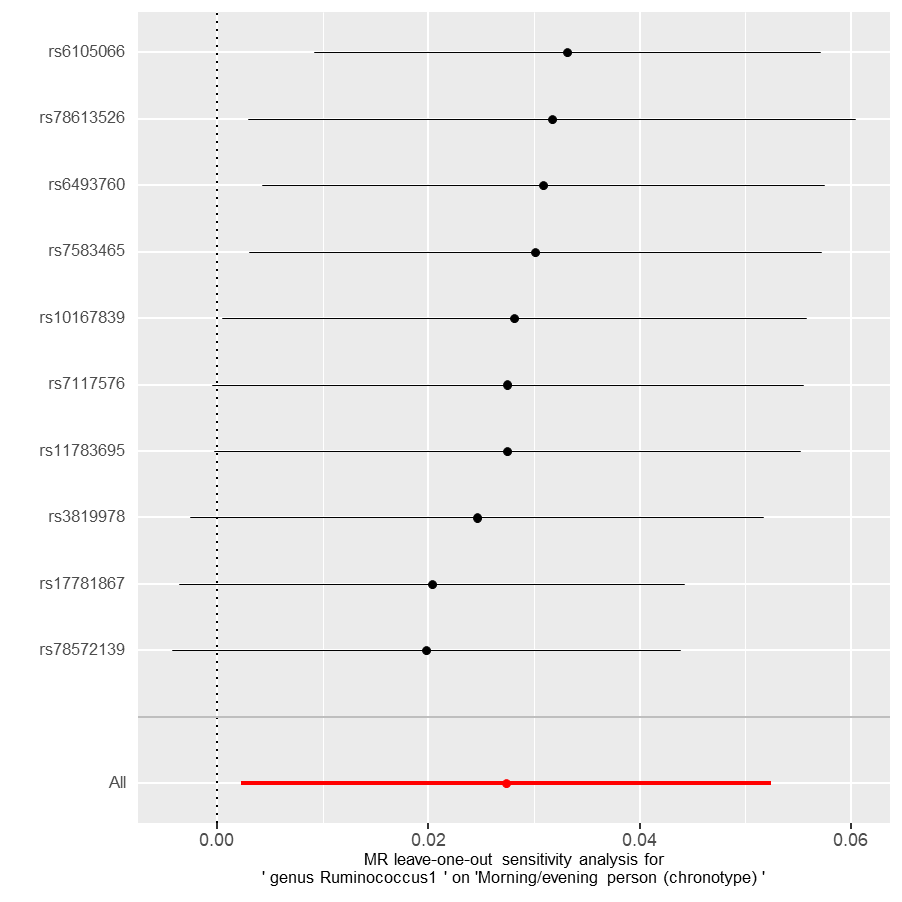

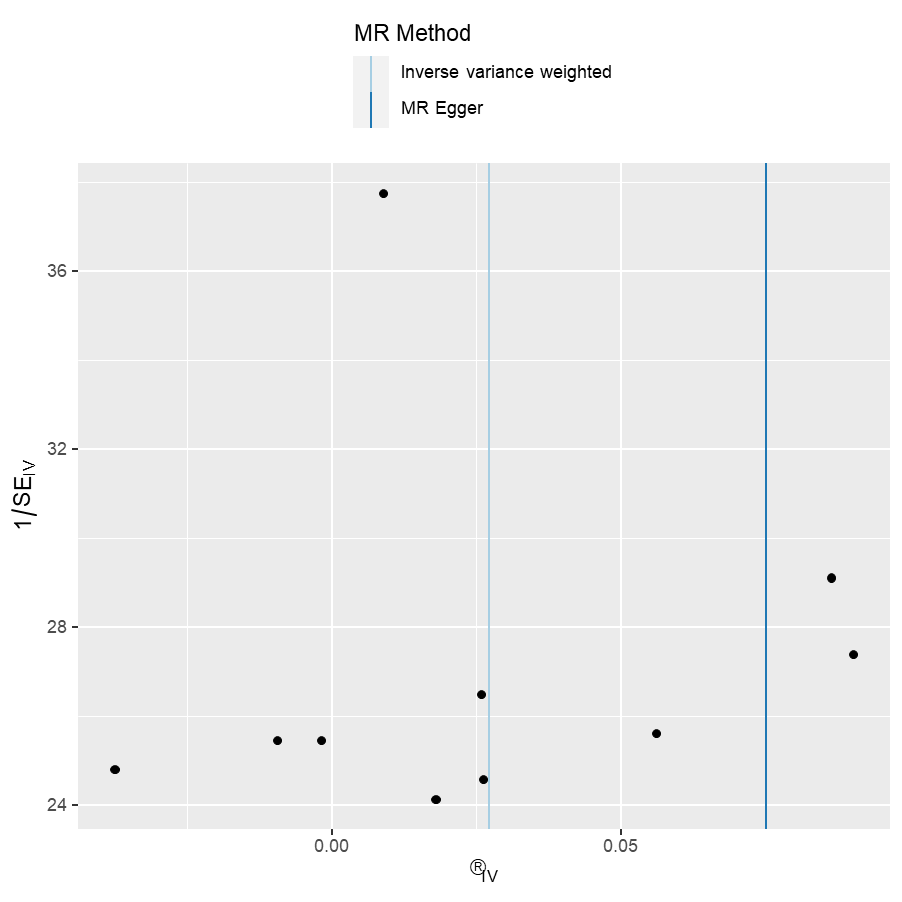
**

**Sup Fig. 86 Scatter plot,** **leave-one-out plot and funnel plot for the causal association between *genus Butyricimonas* and nap during day.**

**
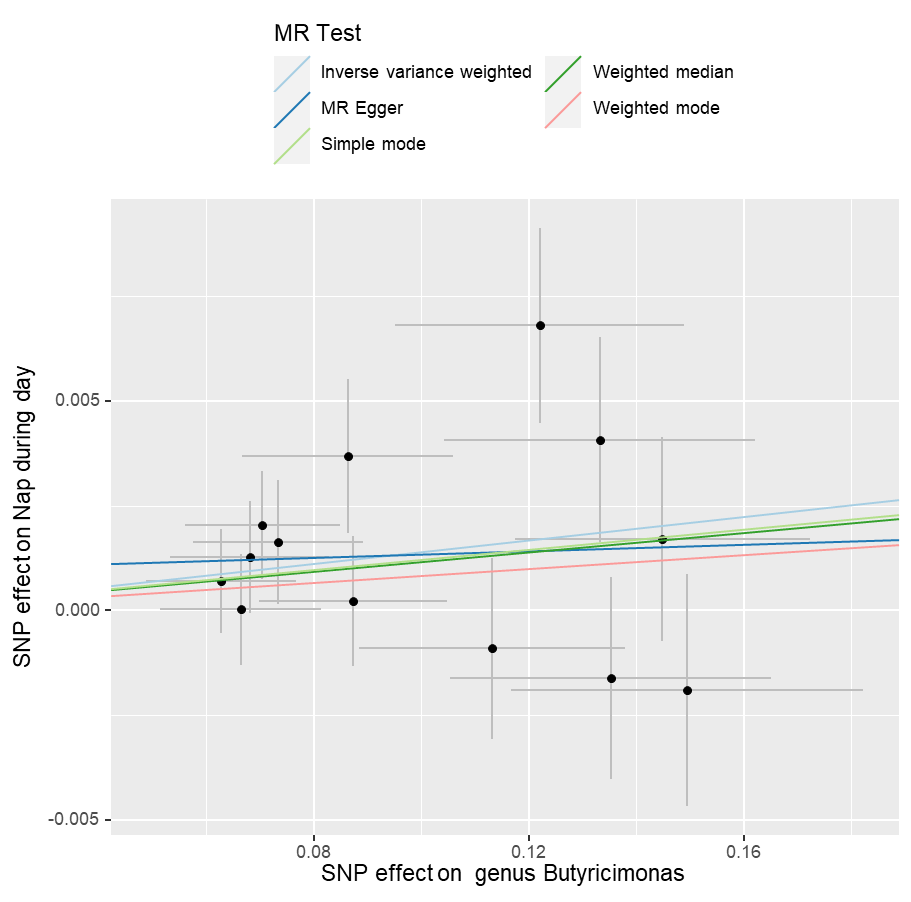

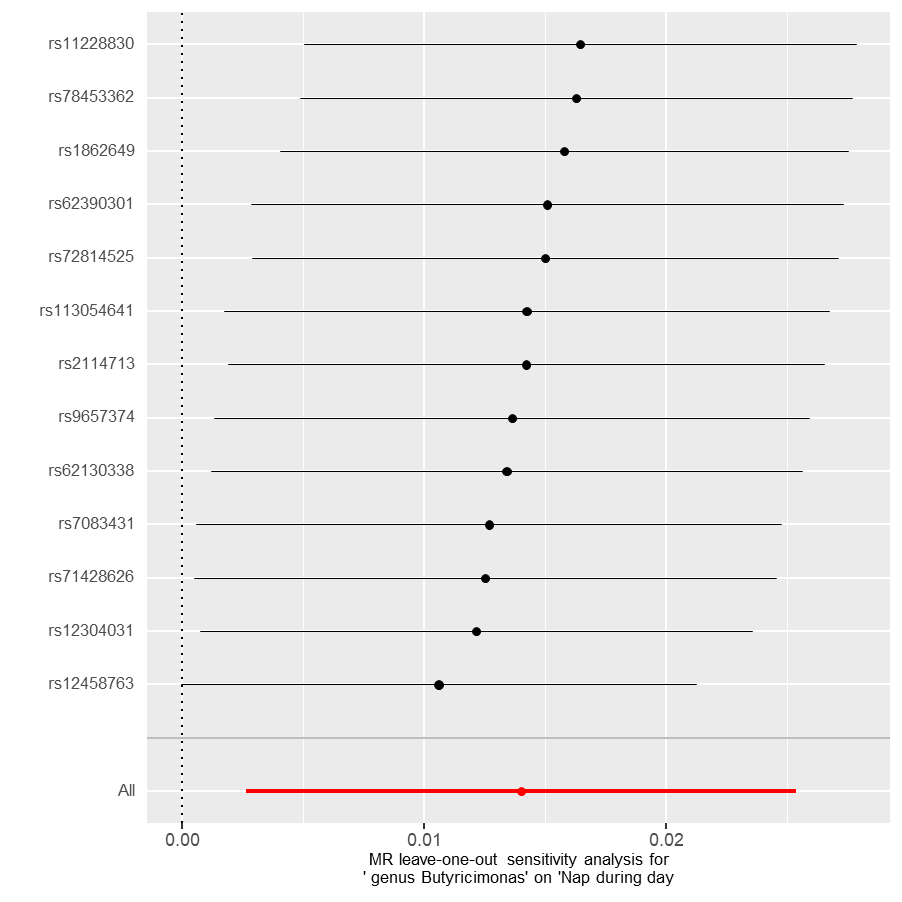

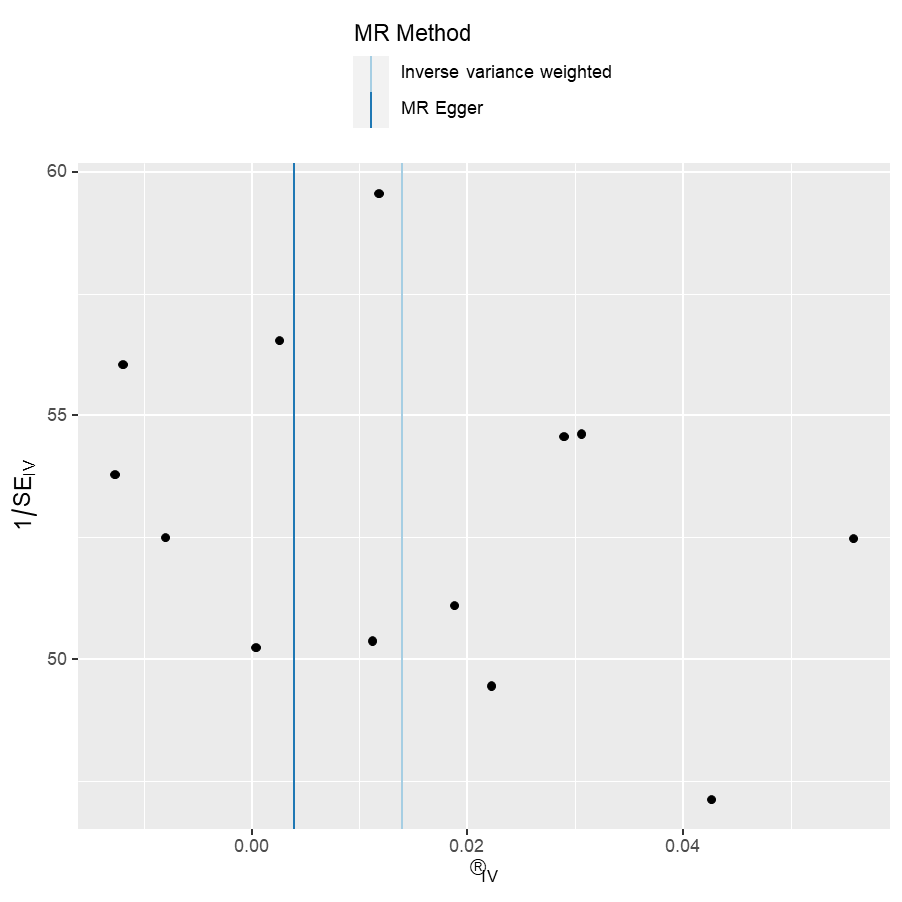
**

**Sup Fig. 87 Scatter plot,** **leave-one-out plot and funnel plot for the causal association between *genus Clostridium sensustricto1* and nap during day.**

**
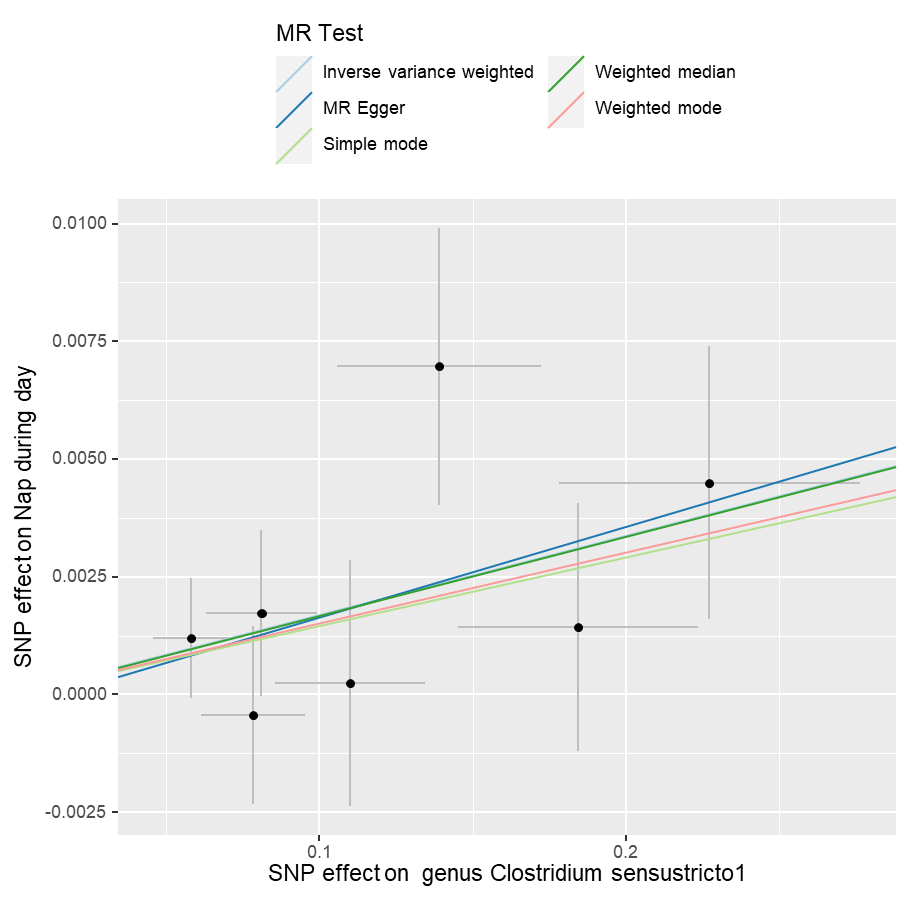

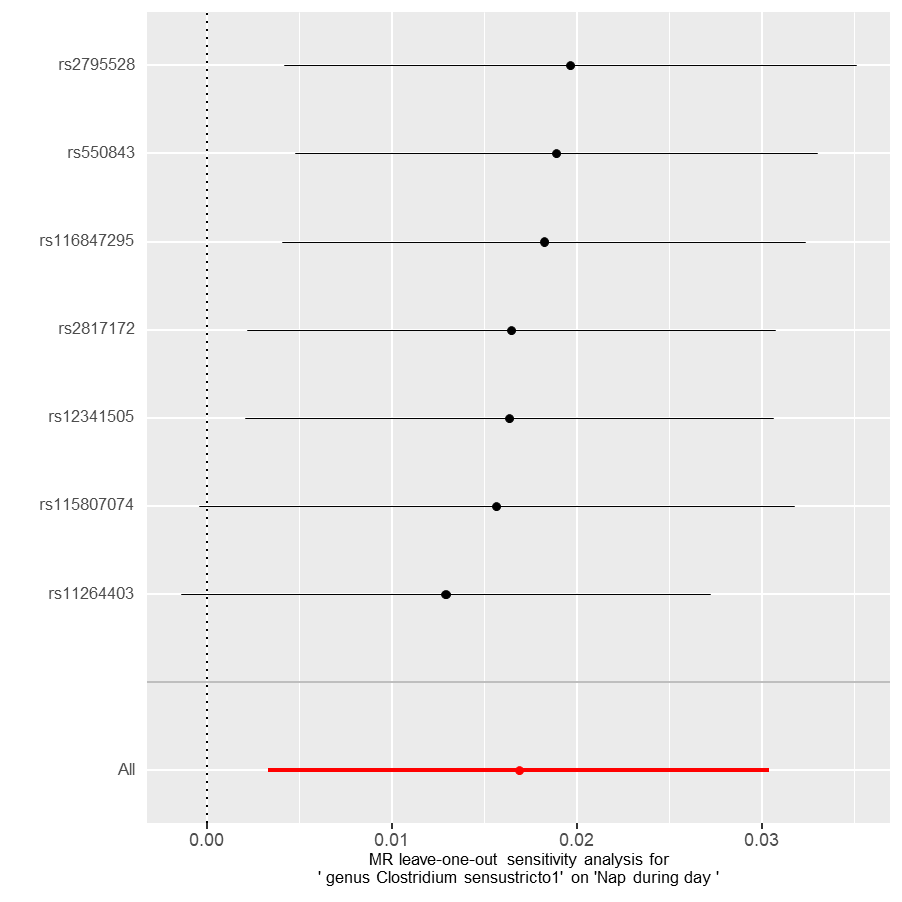

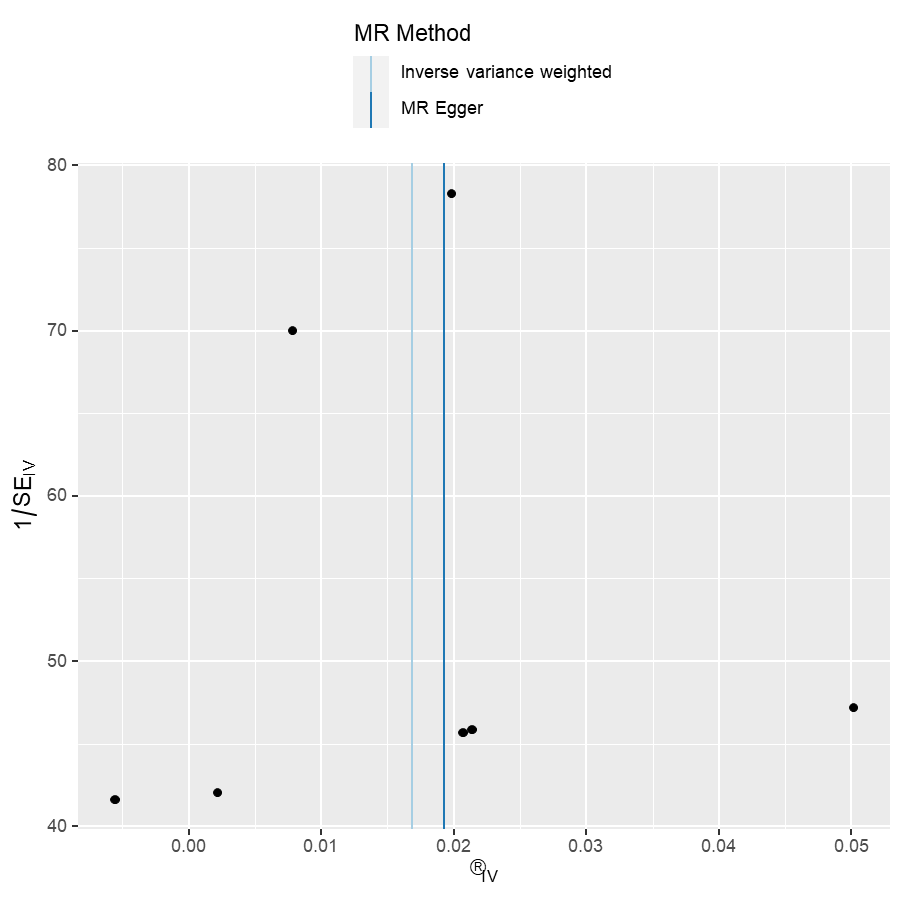
**

**Sup Fig. 88 Scatter plot,** **leave-one-out plot and funnel plot for the causal association between *genus Eubacterium fissicatena group* and nap during day.**

**
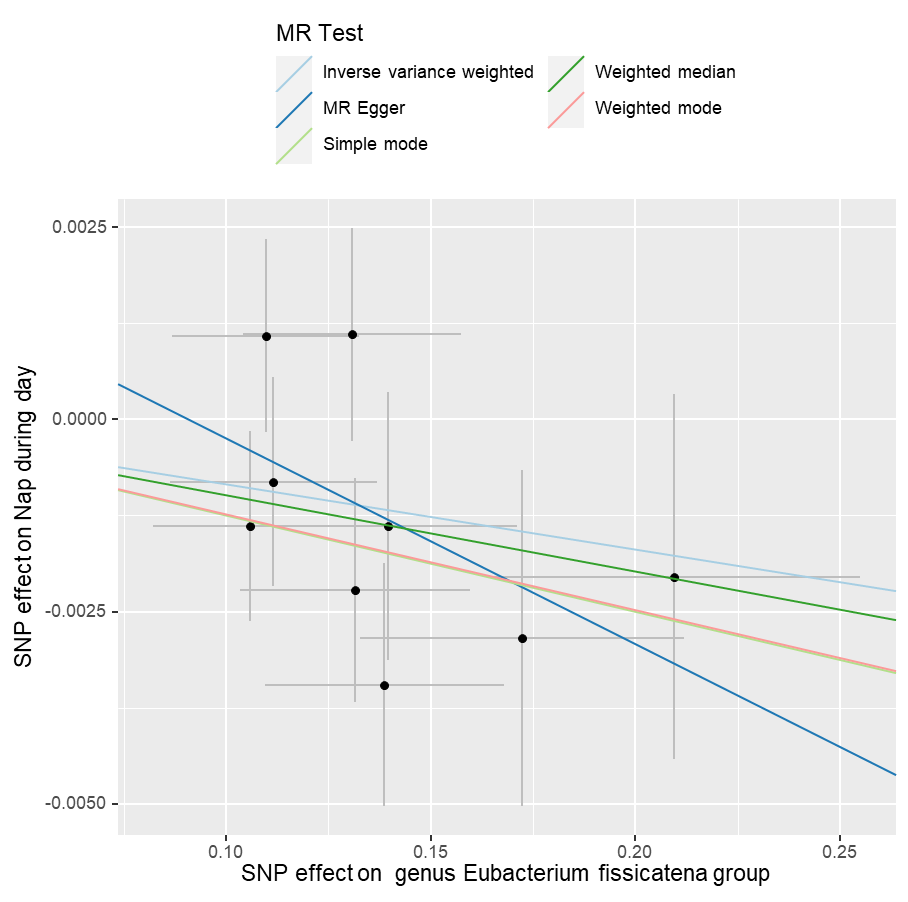

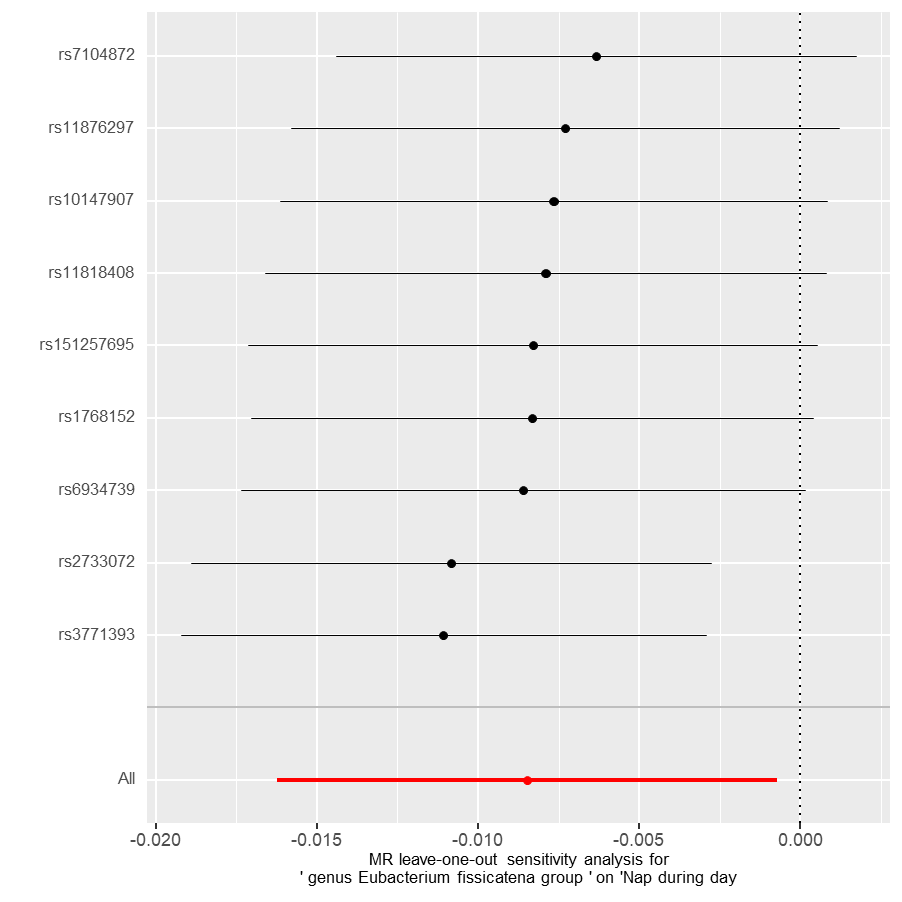

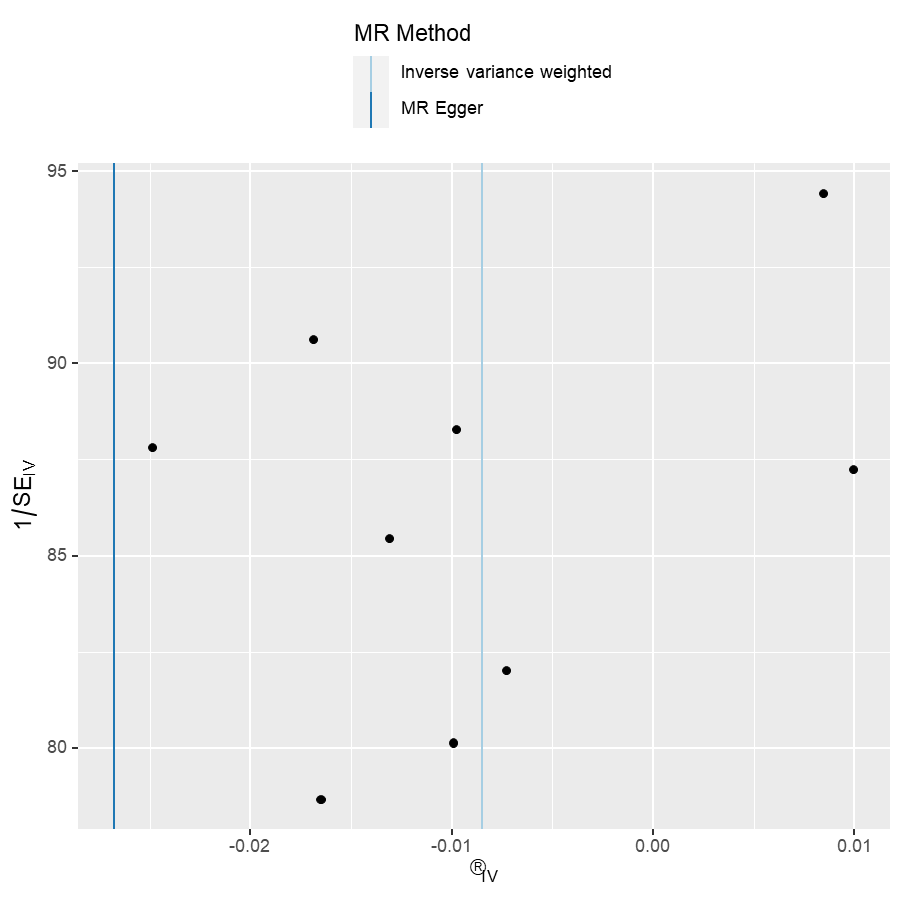
**

**Sup Fig. 89 Scatter plot,** **leave-one-out plot and funnel plot for the causal association between *order genus Holdemanella* and nap during day.**

**
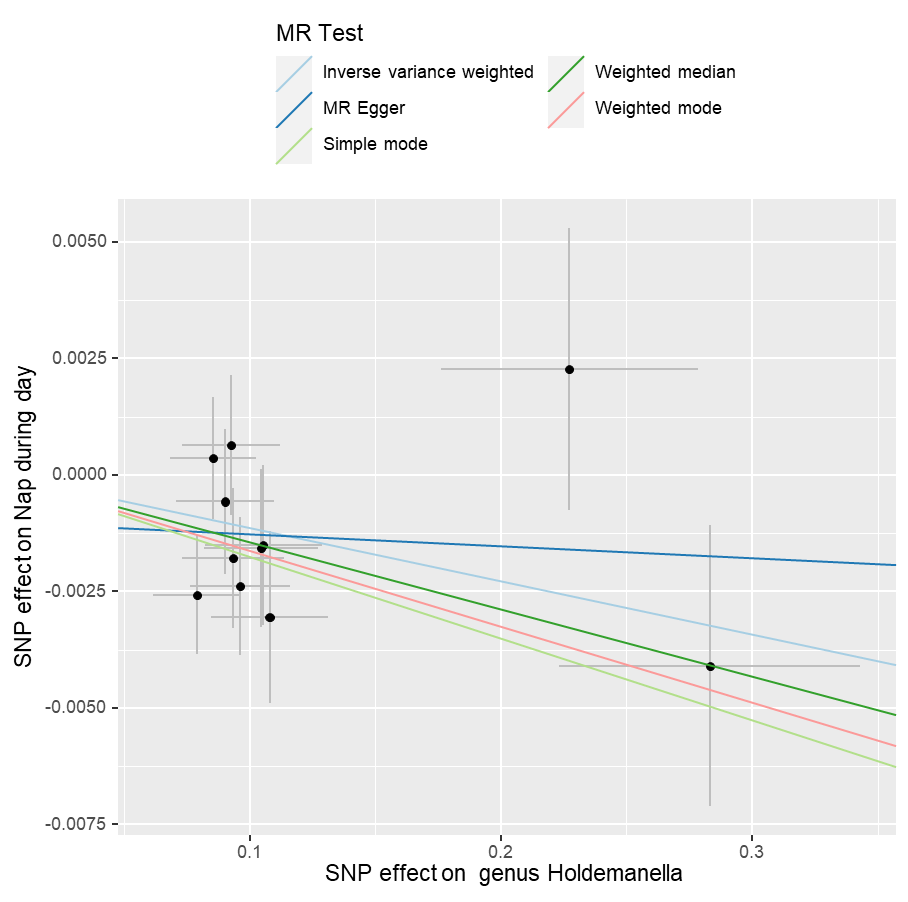

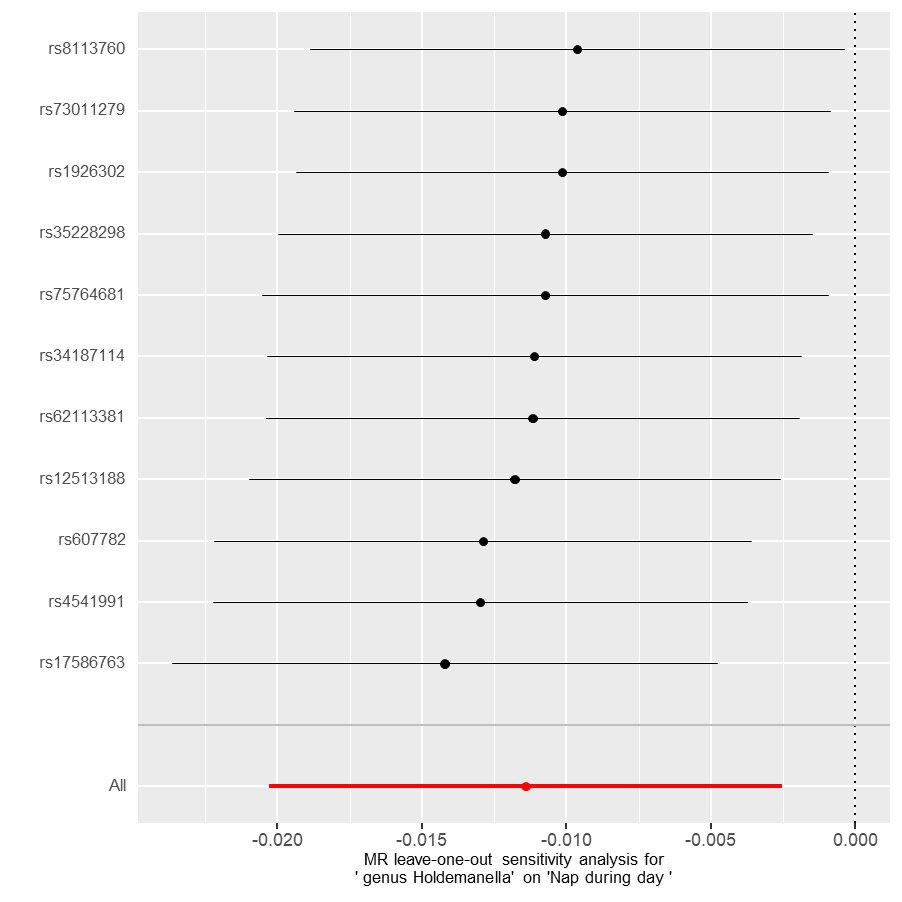

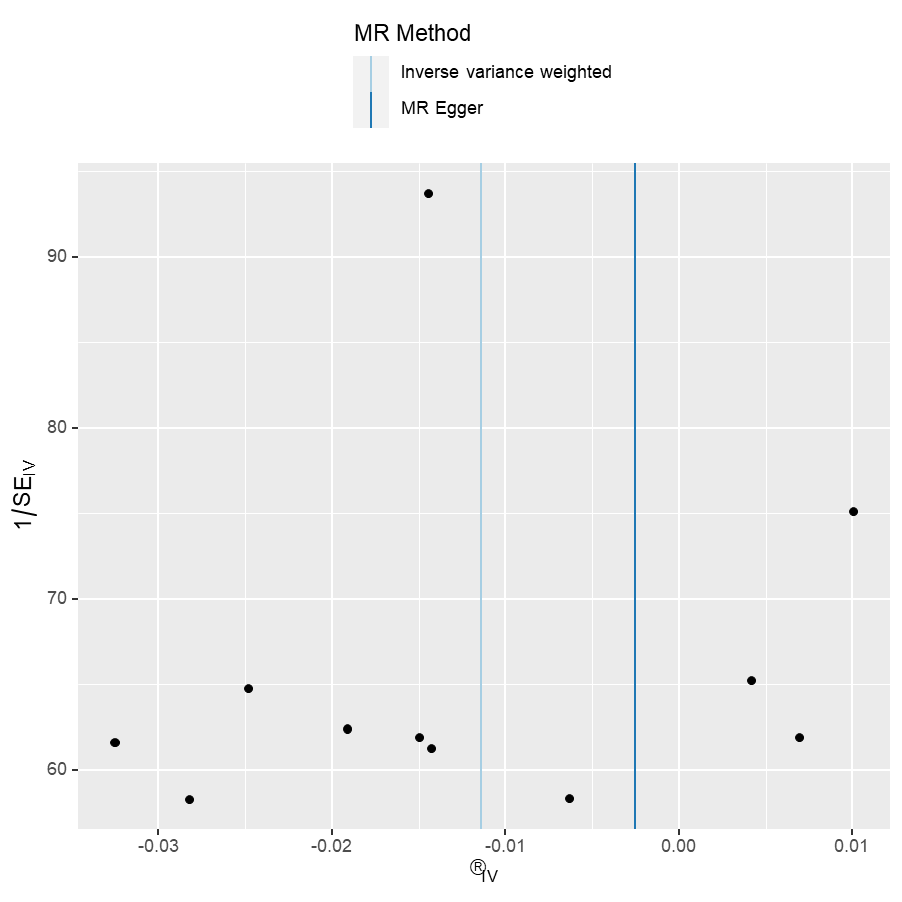
**

**Sup Fig. 90 Scatter plot,** **leave-one-out plot and funnel plot for the causal association between *genus Oxalobacter* and nap during day.**

**
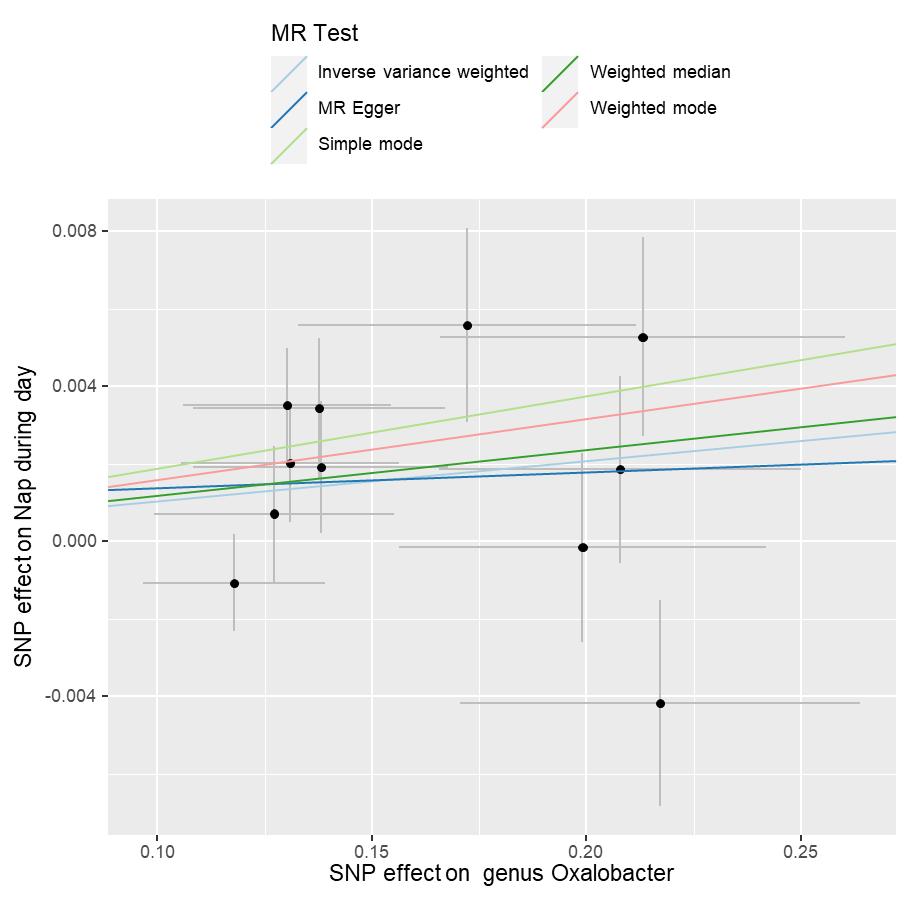

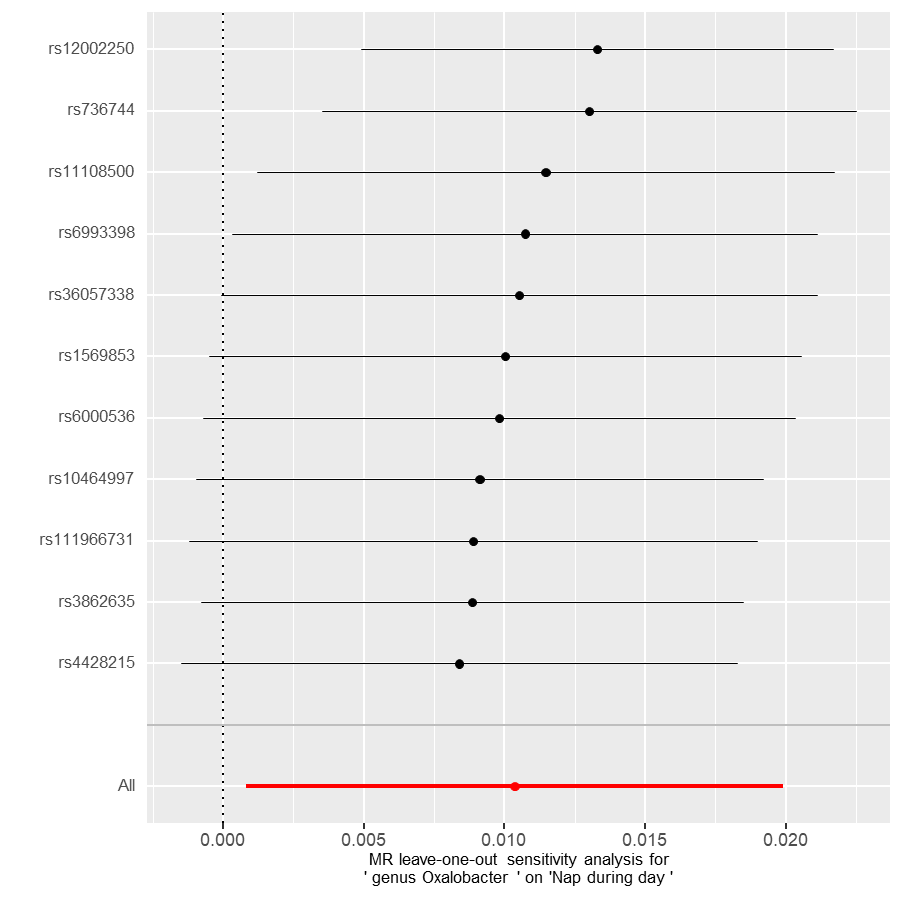

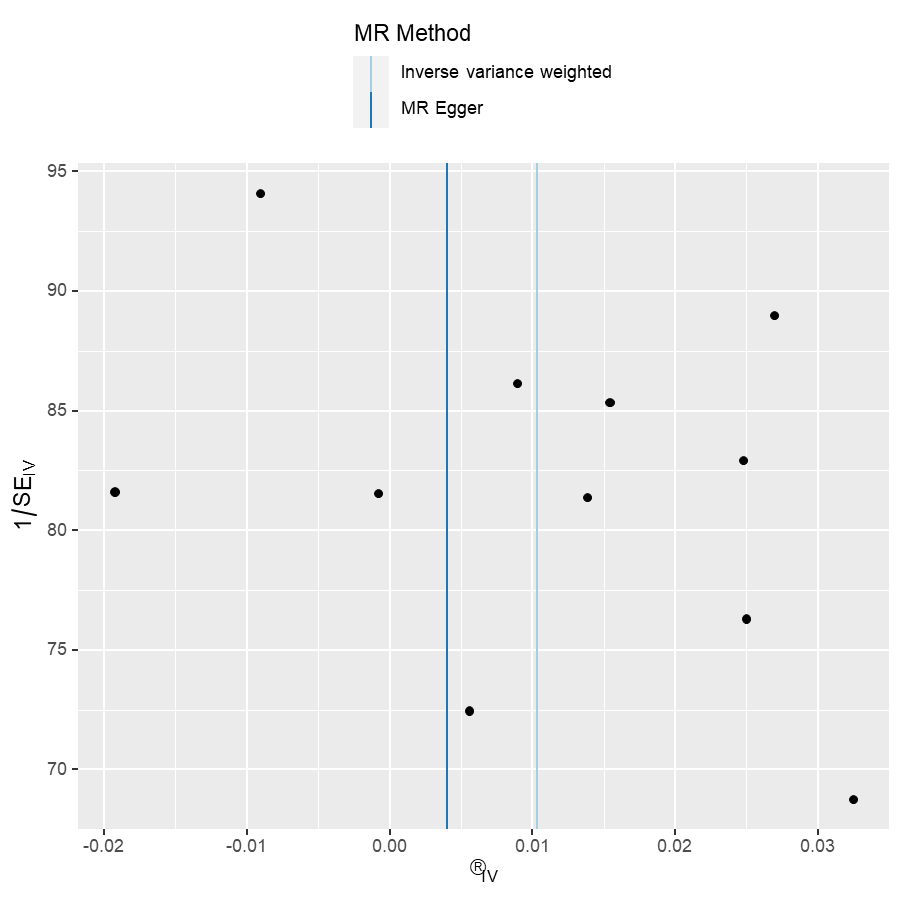
**

**Sup Fig. 91 Scatter plot,** **leave-one-out plot and funnel plot for the causal association between *order NB1n* and nap during day.**


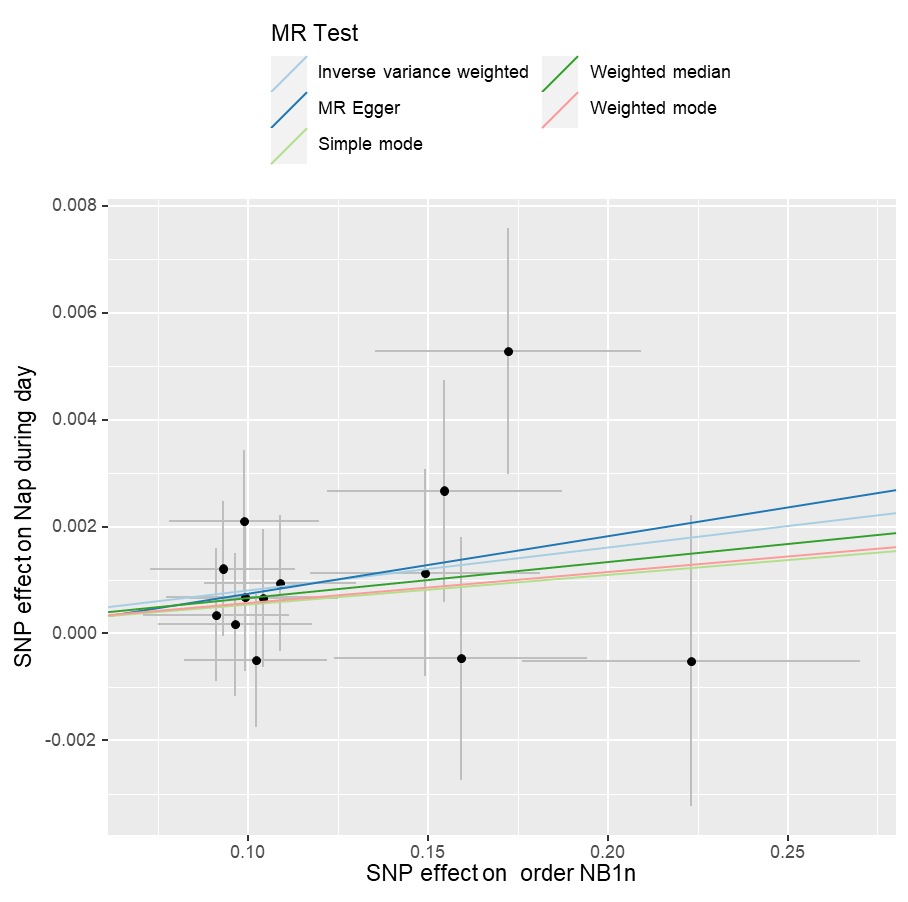

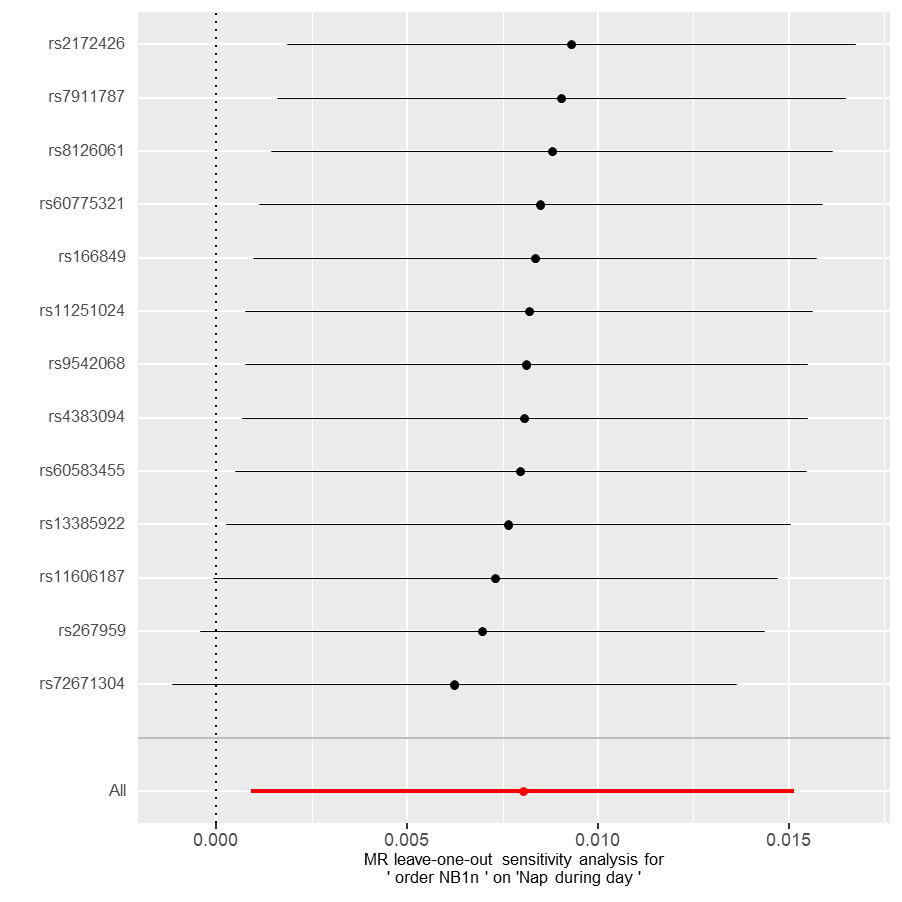

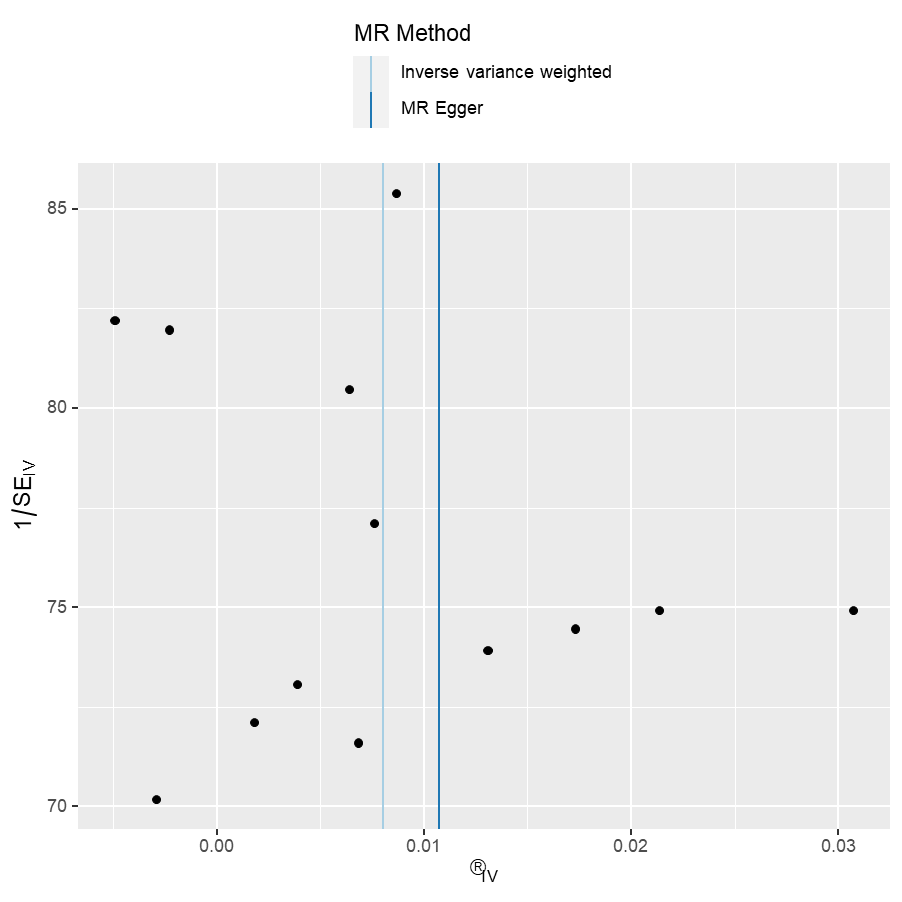


**Sup Fig. 92 Scatter plot,** **leave-one-out plot and funnel plot for the causal association between *class Coriobacteriia* and Sleep duration.**


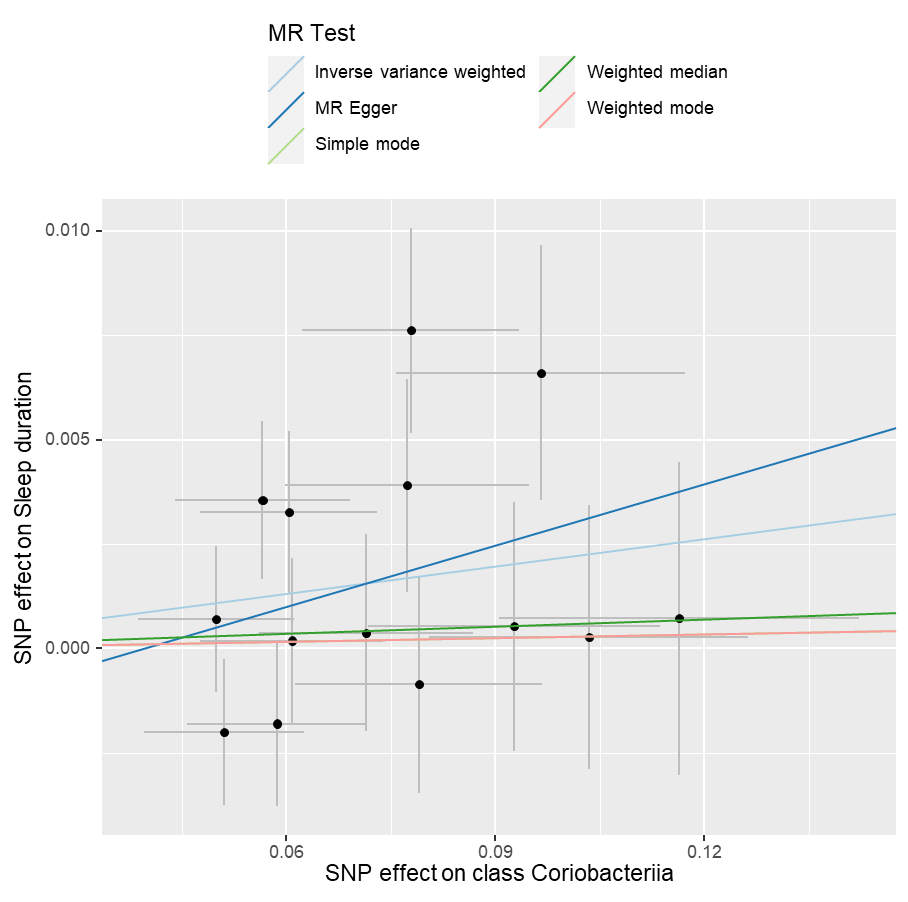

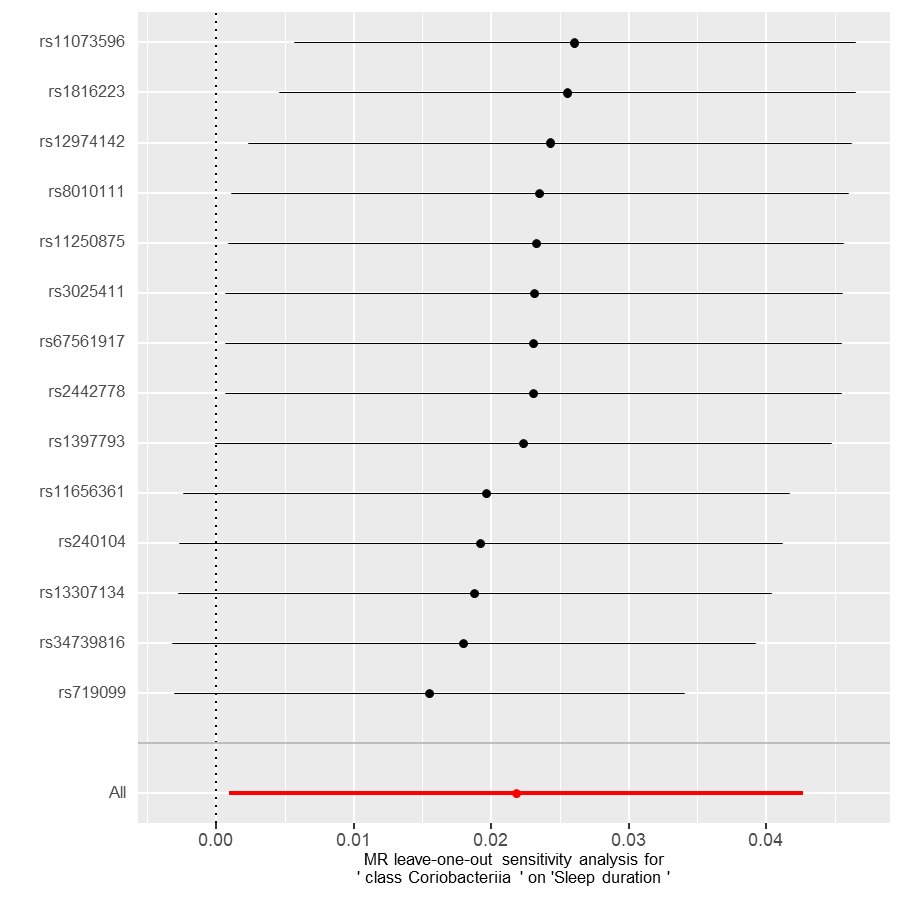

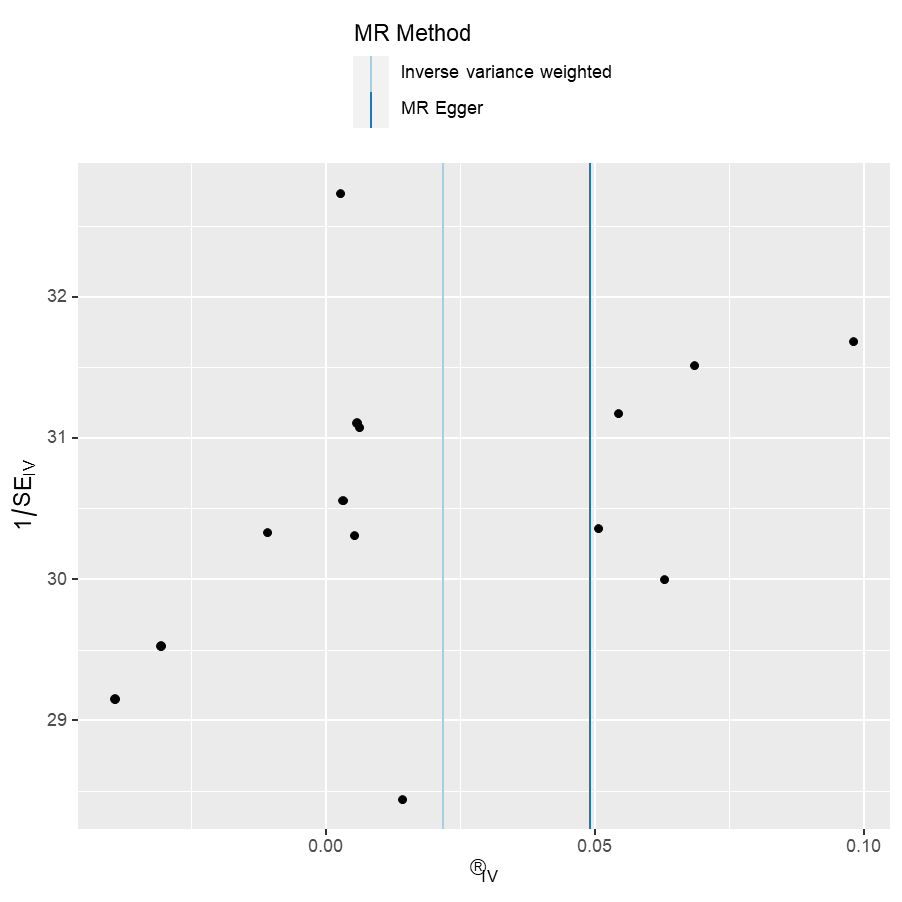


**Sup Fig. 93 Scatter plot,** **leave-one-out plot and funnel plot for the causal association between *family Coriobacteriaceae* and Sleep duration.**

**
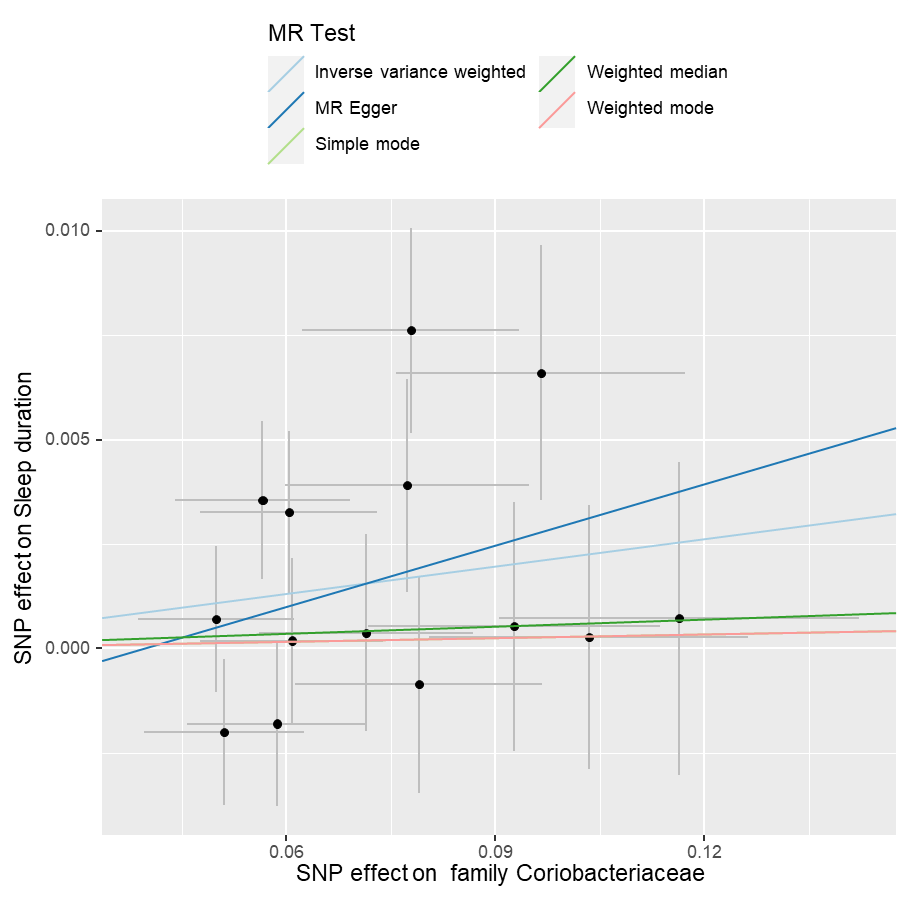

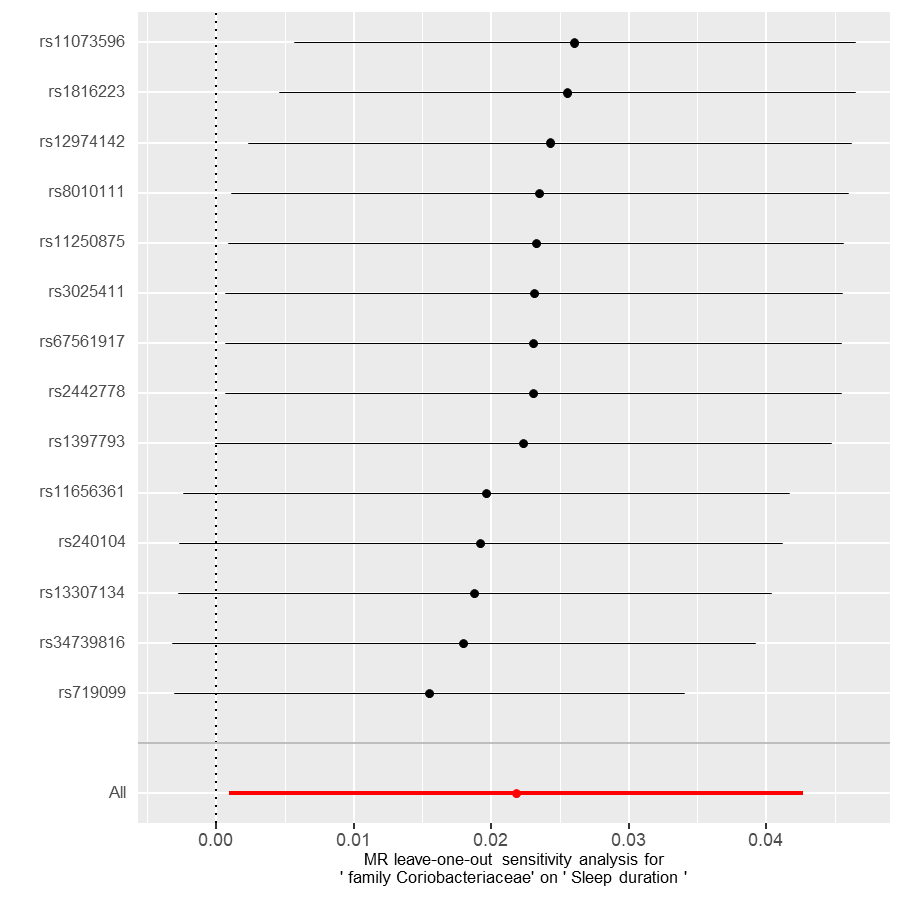

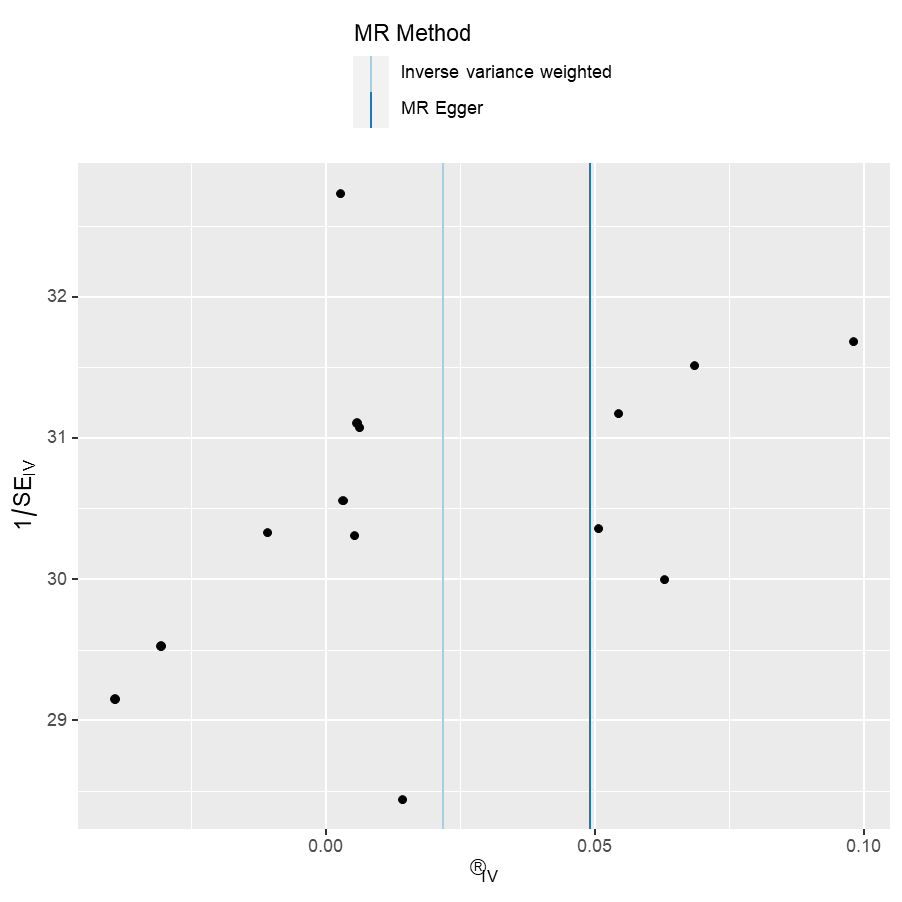
**

**Sup Fig. 94 Scatter plot,** **leave-one-out plot and funnel plot for the causal association between *genus Anaerofilum* and Sleep duration.**

**
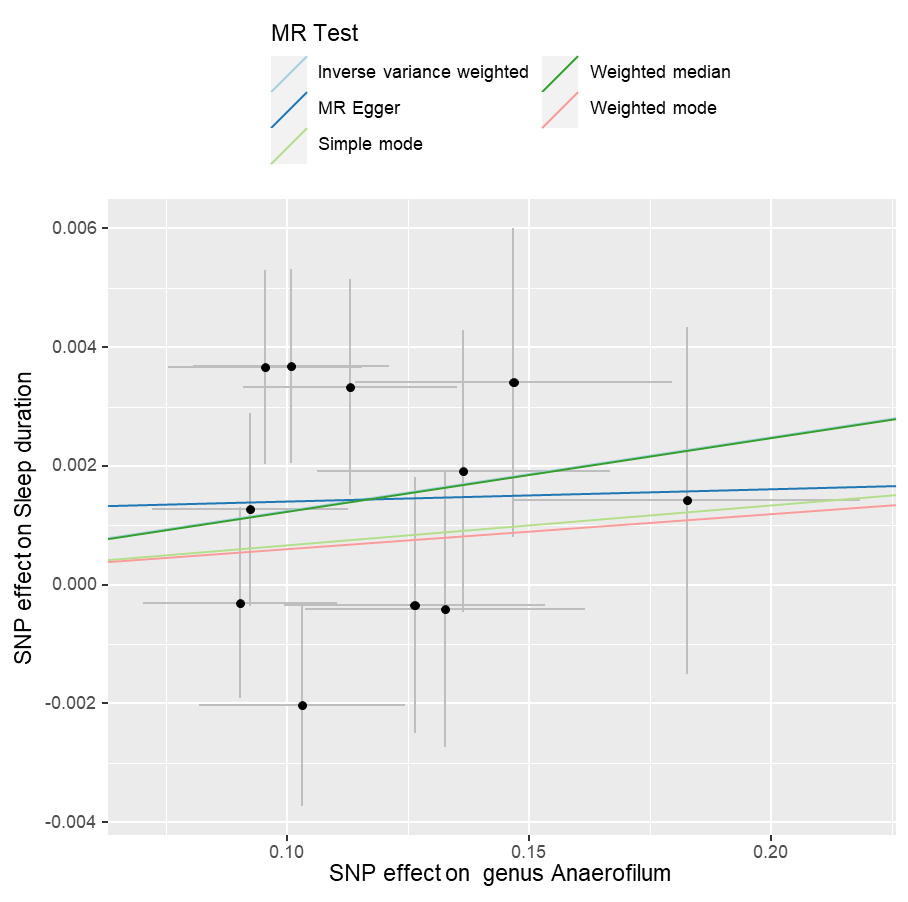
**

**Sup Fig. 95 Scatter plot,** **leave-one-out plot and funnel plot for the causal association between *genus Eubacterium fissicatena group* and Sleep duration.**

**Sup Fig. 96 Scatter plot,** **leave-one-out plot and funnel plot for the causal association between *genus Eubacterium hallii group* and Sleep duration.**

**Sup Fig. 97 Scatter plot,** **leave-one-out plot and funnel plot for the causal association between *genus Odoribacter* and Sleep duration.**

**Sup Fig. 98 Scatter plot,** **leave-one-out plot and funnel plot for the causal association between *genus Victivallis* and Sleep duration.**

**Sup Fig. 99 Scatter plot,** **leave-one-out plot and funnel plot for the causal association between *order Coriobacteriales* and Sleep duration.**

**Sup Fig. 100 Scatter plot,** **leave-one-out plot and funnel plot for the causal association between *order Desulfovibrionales* and Sleep duration.**

**Sup Fig. 101 Scatter plot,** **leave-one-out plot and funnel plot for the causal association between *phylum Lentisphaerae* and Sleep duration.**

**Sup Fig. 102 Scatter plot,** **leave-one-out plot and funnel plot for the causal association between *genus Eubacterium ruminantium group* and snoring.**

**Sup Fig. 103 Scatter plot,** **leave-one-out plot and funnel plot for the causal association between *genus Haemophilus* and snoring.**

**Sup Fig. 104 Scatter plot,** **leave-one-out plot and funnel plot for the causal association between *genus Ruminococcaceae UCG010* and snoring.**

**Sup Fig. 105 Scatter plot,** **leave-one-out plot and funnel plot for the causal association between *genus Ruminococcus torques group* and snoring.**

**Sup Fig. 106 Scatter plot,** **leave-one-out plot and funnel plot for the causal association between *genus Senegalimassilia* and snoring.**

**Sup Fig. 107 Scatter plot,** **leave-one-out plot and funnel plot for the causal association between *genus Terrisporobacter* and snoring.**
